# Halogenation of neolignan scaffolds reveals stage-specific anthelmintic activity against *Haemonchus contortus*

**DOI:** 10.1016/j.ijpddr.2026.100666

**Published:** 2026-08-10

**Authors:** Kanchana Wijesekera, Aya C. Taki, Joseph J. Byrne, Jonathan M. White, Anthony R. Carroll, Robin B. Gasser, Rohan A. Davis

**Affiliations:** a Institute for Biomedicine and Glycomics, Griffith University, Brisbane, QLD, 4111, Australia; b School of Environment and Science, Griffith University, Brisbane, QLD, 4111, Australia; c Department of Veterinary Biosciences, Melbourne Veterinary School, Faculty of Science, The University of Melbourne, Parkville, VIC, 3010, Australia; d School of Chemistry and Bio21 Institute, The University of Melbourne, Melbourne, VIC, 3010, Australia; e School of Environment and Science, Griffith University, Gold Coast, QLD, 4222, Australia; f NatureBank, Griffith University, Brisbane, QLD, 4111, Australia

**Keywords:** Anthelmintic activity, Halogenated neolignan derivatives, Natural products, *Styrax suberifolius*, Suberifolioside B, *Haemonchus contortus*

## Abstract

Gastrointestinal nematodes, including *Haemonchus contortus*, represent a major constraint to livestock production globally, and the increasing prevalence of anthelmintic resistance necessitates the discovery of new chemotypes. Natural products provide a rich and underexplored source of bioactive scaffolds for anthelmintic development. However, there remains a need to systematically optimise such scaffolds to define structure–activity relationships in whole-organism systems. Here, we investigated neolignan compounds from the fruits of *Styrax suberifolius* and assessed the impact of halogenation on their anthelmintic activity. Two known neolignans, suberifolioside A and equiselignan B, were isolated and used to generate a series of 11 halogenated derivatives using *N*-halosuccinimide chemistry. In addition, a new glycoside, suberifolioside B, together with two known metabolites, 7*R*,8*S*-dihydrodehydrodiconiferyl alcohol and 3′,4-*O*-dimethylcedrusin, were characterised by spectroscopic and spectrometric methods, and the absolute configuration of equiselignan B was confirmed by X-ray crystallography. All compounds were evaluated for activity against larval stages of *H. contortus* using an established in vitro phenotypic assay. While no compounds significantly affected exsheathed third-stage larvae, multiple derivatives displayed activity against fourth-stage larvae, indicating pronounced stage-specific susceptibility. Notably, halogenation of the neolignan scaffold modulated biological activity, with several derivatives inducing substantial reductions in larval motility and distinct abnormal phenotypes. This modulation was non-linear, with both mono- and poly-halogenated derivatives displaying activity. A monobrominated ether analogue exhibited maximal motility inhibition approaching that observed for moxidectin under the assay conditions, highlighting the potential of this scaffold for optimisation. These findings demonstrate that halogenated neolignans represent a tractable chemical class for anthelmintic discovery and reveal stage-specific vulnerabilities in *H. contortus*. This work provides a foundation for further optimisation and prioritisation of neolignan derivatives for downstream studies of mechanism of action and translational potential.

## Highlights

• Neolignans from *Styrax suberifolius* were halogenated using *N*-halosuccinimides.
• Eleven halogenated neolignan derivatives were synthesised and structurally characterised.
• A new neolignan glycoside, suberifolioside B, was isolated from the crude extract of the fruits.
• Halogenation enhanced stage-specific anthelmintic activity against *Haemonchus contortus.*
• A monobrominated analogue showed activity comparable to moxidectin.

## Graphical abstract

**Figure:**
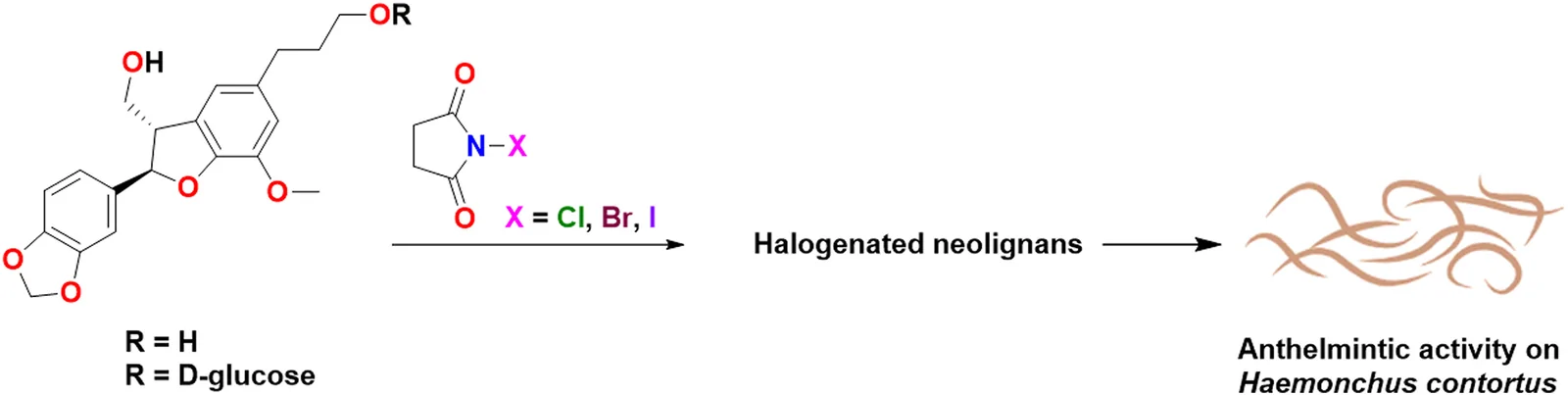

## 1 Introduction

Parasitic infections caused by gastrointestinal nematodes (GINs) continue to impose substantial health and economic burdens on livestock and human populations worldwide, contributing to reduced productivity, chronic disease and major financial losses (Beveridge and Emery, 2015; Bowman, 2020). Among these parasites, the blood-feeding nematode *Haemonchus contortus* is one of the most pathogenic species affecting ruminants, with widespread distribution and a well-documented capacity to cause severe production losses in sheep, goats and cattle (Besier et al., 2016). Control of GIN infections relies predominantly on a limited number of anthelmintic drug classes, and resistance to these compounds has emerged globally, often within a relatively short period following their introduction (Kotze and Prichard, 2016; Charlier et al., 2020, 2022). The increasing prevalence of resistance, including multidrug resistance in livestock systems, highlights the need to identify new chemical scaffolds and to explore strategies that extend beyond the optimisation of existing drug classes (Nixon et al., 2020; Partridge et al., 2020; Packianathan et al., 2023).

Natural products represent a major source of chemically diverse scaffolds for antiparasitic discovery (Hayes et al., 2022a; 2022b; Wijesekera et al., 2025a), occupying regions of chemical space that are not well represented in synthetic libraries. Rather than relying solely on large-scale screening, targeted modification of bioactive natural-product scaffolds provides a complementary approach to discovery, enabling systematic exploration of structure–activity relationships and identification of features that enhance biological activity. In anthelmintic discovery, there remains a particular need to identify chemically tractable scaffolds that can be systematically modified and evaluated in whole-organism assays. Within this context, halogenation represents a simple yet powerful chemical modification strategy. The introduction of halogen atoms, including chlorine, fluorine, bromine and iodine, can alter electronic properties, increase lipophilicity and influence molecular interactions with biological targets, thereby modulating biological activity (Neumann et al., 2008; Wagner et al., 2009). Although halogenated natural products are relatively rare in higher plants (Engvild, 1986), halogenation has been associated with enhanced activity across a wide range of compound classes, including antiparasitic, antibacterial and anticancer agents (Gribble, 1992; Vairappan et al., 2004; Dias et al., 2013). These effects are reflected in drug discovery more broadly, where halogen atoms are frequently incorporated to optimise potency and physicochemical properties (Wang et al., 2022).

Recent work has identified neolignans from *Styrax suberifolius* as a chemically tractable scaffold with measurable anthelmintic activity. In particular, suberifolioside A (**4**), equiselignan B (**5**) and egonol (**6**) have been shown to exhibit biological activity, and semisynthetic modification of these compounds has yielded derivatives with enhanced effects on larval stages of *H. contortus* (see Wijesekera et al., 2025b). A trimethylated ether analogue displayed substantial activity against both exsheathed third-stage larvae (xL3s) and fourth-stage larvae (L4s), producing marked reductions in motility and micromolar potency (Wijesekera et al., 2025b). These findings indicate that the neolignan scaffold is responsive to chemical modification and provides a suitable framework for systematic optimisation.

In this study, we investigated whether targeted halogenation of the neolignan scaffold could modulate anthelmintic activity and provide insight into structure–activity relationships. During large-scale extraction and purification to obtain sufficient quantities of suberifolioside A (**4**) and equiselignan B (**5**) for halogenation studies, we isolated a new neolignan glycoside, suberifolioside B (**1**), together with two known minor metabolites, 7*R*,8*S*-dihydrodehydrodiconiferyl alcohol (**2**) (Liu et al., 2018) and 3′,4-*O*-dimethylcedrusin (**3**) (Anantachoke et al., 2020). Subsequent semisynthetic modification using commercially available *N*-halosuccinimide reagents enabled the generation of a series of halogenated derivatives, which were structurally characterised and evaluated in phenotypic assays against xL3 and L4 stages of *H. contortus*. By focusing on targeted chemical modification rather than library-scale screening (i.e. HTS), this work defines how halogenation influences neolignan bioactivity and provides initial structure–activity insights to guide further optimisation of this scaffold for anthelmintic discovery.

## 2 Materials and methods

### 2.1 Procurement of *Haemonchus contortus*

The Haecon-5 strain of *Haemonchus contortus* was maintained in experimental sheep according to established protocols (Taki et al., 2021), in compliance with institutional animal ethics guidelines (permit no. 23983; University of Melbourne) and Australian regulations. Helminth-free Merino sheep (six months old, male) were orally inoculated with 7000 third-stage larvae (L3s) of *H. contortus*. Faecal samples containing parasite eggs were collected daily from sheep with patent infection, commencing four weeks post-infection. Eggs were incubated at 27 °C and >90% relative humidity for seven days to develop into third-stage larvae (L3s).

Subsequently, L3s were collected, suspended in tap water and filtered through two layers of nylon mesh (20 μm pore size; Sefar, Australia) to remove debris and non-viable material. Filtered larvae were stored in 0.9% NaCl at a density of 2000 L3s per mL at 11 °C for up to six months (Preston et al., 2015). Larval viability was assessed prior to use based on motility and morphological integrity. Immediately prior to use, L3s were exsheathed and sterilised by incubation in 0.15% (v/v) sodium hypochlorite at 38 °C for 20 min with gentle agitation (Taki et al., 2021), followed by five washes in sterile saline by centrifugation (800 × g, 5 min, 22–24 °C). After the final wash, exsheathed L3s (xL3s) were suspended in sterile lysogeny broth (LB) (Bertani, 1951) supplemented with 100 IU/mL penicillin, 100 μg/mL streptomycin and 0.25 μg/mL amphotericin B (Fungizone®, Thermo Fisher Scientific, USA), referred to as LB*. To generate L4s, xL3s were incubated at 38 °C, 10% (v/v) CO_2_ and >90% relative humidity for 168 h prior to compound exposure. L4s were identified by completion of the L3-to-L4 moult and the presence of the characteristic buccal capsule (Veglia, 1915).

### 2.2 Bioassay for evaluation of anthelmintic activity of pure compounds

Compounds **7**–**15** and **17**–**18** were individually tested at 100 μM for anthelmintic activity against xL3s and in vitro-raised L4s of *H. contortus* using an established phenotypic assay (Taki et al., 2021). Dose–response assays were conducted to estimate half-maximal inhibitory concentrations (IC_50_ values), with monepantel (Zolvix™; Elanco, USA) and moxidectin (Cydectin®^;^ Virbac, France) used as positive reference controls.

Larvae were dispensed into 96-well plates (Corning, USA) at a density of 300 xL3s or L4s per well in a final volume of 100 μL LB*. Compounds were applied as nine-point, two-fold serial dilutions (100 μM to 0.39 μM). Plates were incubated at 38 °C, 10% (v/v) CO_2_ and >90% relative humidity. All compounds were tested in triplicate wells across three independent experiments to ensure reproducibility. Larval motility was measured following optimised incubation periods of 168 h for xL3s and 90 h for L4s. Motility measurements were normalised to untreated controls and expressed as percentage inhibition. Following motility measurements, larvae were fixed with 1% (v/v) iodine and examined using a Leica M80 light microscope to assess morphological changes relative to untreated controls. Compound concentrations were log_10_-transformed and fitted using a four-parameter variable-slope concentration-response model in GraphPad Prism (v10.2.2; GraphPad Software, San Diego, CA, USA), with the upper constraint fixed at 100%. IC_50_ values were estimated from at least three independent experiments. Statistical differences in larval motility were assessed using one-way ANOVA followed by Tukey's multiple comparison test. Differences were considered statistically significant at *P* < 0.05.

### 2.3 Chemistry procedures

Melting points were measured using a Cole–Parmer melting point apparatus and are uncorrected. Specific rotations were recorded using a JASCO P-2000 polarimeter. Electronic circular dichroism (ECD) spectra were recorded on a JASCO J-1500 CD spectrophotometer. NMR spectra were recorded at 25 °C on a Bruker AVANCE III HDX 800 MHz spectrometer equipped with a cryoprobe, with ^1^H and ^13^C chemical shifts referenced to solvent peaks for DMSO-*d*_6_ at *δ*_H_ 2.50 and *δ*_C_ 39.52. LRESIMS data were recorded using an Ultimate 3000 RS UHPLC system fitted with a Thermo Scientific Accucore C_18_-bonded column (2.6 μm, 80 Å, 150 × 2.1 mm) coupled to a Thermo Fisher Scientific ISQ EC single quadrupole ESI mass spectrometer. HRESIMS data were acquired on a Bruker maXis II ETD ESI-qTOF mass spectrometer. Silica flash chromatography was performed using Isolute® cartridges (53 μm, 54 Å, 70 × 30 mm), and GRACE Davisil C_18_-bonded silica (35–70 μm, 60 Å) was used for pre-adsorption prior to reversed-phase HPLC (RP-HPLC) separations. RP-HPLC separations were performed using a Waters 2535 pump with a Waters 2998 photodiode array detector and a Gilson GX-281 autosampler, employing Thermo Betasil or Phenomenex Luna C_18_-bonded columns. Solvents were removed using a Büchi R-144 rotary evaporator or a GeneVac XL4 centrifugal evaporator. All solvents used for chromatography, UV, MS, ECD and optical rotation were HPLC grade (Honeywell Burdick & Jackson or Lab-Scan), and H_2_O was purified using a Sartorius Arium® Pro VF system. Merck silica gel 60 F254 pre-coated aluminium plates were used for thin-layer chromatography (TLC) and analysed under UV light at 254 nm. Chemical reagents (e.g. *N*-halosuccinimides, L-glucose and D-glucose) were all purchased from Sigma-Aldrich. NMR spectra were processed using MestReNova v14.3.0, and chemical structures were drawn using ChemDraw v23.1.1.3.

### 2.4 Plant material, extraction and isolation

Fruits of *Styrax suberifolius* (Styracaceae) were collected in Guilin, Guangxi Province, China (110°21′890″E, 25°54′344″N) in May 1998, and a voucher specimen has been deposited at Zi Yuan Medical Company, Guangxi, China. A dried fruit sample (20 g) of *S. suberifolius* was extracted using CH_2_Cl_2_:MeOH (4:1, 500 mL, 2 h) followed by MeOH (500 mL, 2 h; 500 mL, 16 h) and filtered under gravity, yielding 2.6 g and 1.3 g of extract, respectively.

Suberifolioside A (**4**), equiselignan B (**5**) and egonol (**6**) were isolated from the CH_2_Cl_2_:MeOH extract (Wijesekera et al., 2025a). The MeOH extract was examined to obtain sufficient quantities of compounds **4** and **5** for halogenation studies. The extract was pre-adsorbed onto silica (∼1 g) and subjected to flash chromatography using a stepwise gradient from 100% CH_2_Cl_2_ to 100% MeOH (100 mL flushes), yielding 25 fractions (F1–F25), which were analysed by silica TLC (10:90 MeOH:CH_2_Cl_2_).

Fractions F8–F14 yielded equiselignan B (**5**, 332.0 mg, 1.66% dry wt) together with 7*R*,8*S*-dihydrodehydrodiconiferyl alcohol (**2**, 9.0 mg, 0.04% dry wt) and 3′,4-*O*-dimethylcedrusin (**3**, 38.3 mg, 0.19% dry wt). Based on ^1^H NMR and LC–MS data, polar fractions F15–F20 were combined and subjected to semipreparative RP-HPLC, yielding a new natural product suberifolioside B (**1**, 18.9 mg, 0.09% dry wt) and additional quantities of suberifolioside A (**4**, 120.0 mg, 0.6% dry wt), equiselignan B (**5**, 13.0 mg, 0.06% dry wt) and egonol (**6**, 13.6 mg, 0.07% dry wt).

Suberifolioside B (**1**): stable brown gum; ${\left[\alpha \right]}_{D}^{24}$ −10.0 (*c* 0.06, MeOH); UV (MeOH) *λ*_max_ (log *ε*) 211 (0.39), 234 (0.33), 272 (0.26) nm; ECD (MeOH) *λ*_ext_ 210 nm (Δε −14.9), 225 nm (Δε +0.5), 243 nm (Δε −2.2), 297 nm (Δε −1.5); ^1^H and ^13^C NMR data, see Table 1; LRESIMS *m/z* 705 [M+Na]^+^, 727 [M + HCHO]^−^; HRESIMS *m/z* 705.2357 [M+Na]^+^ (calcd for C_32_H_42_O_16_Na, 705.2365).

**Table 1:** NMR data for suberifolioside B (**1**) in DMSO-*d*_6_^a^.

Position	*δ*_H_, (mult., *J* in Hz)	*δ*_C_, type	COSY	HMBC	ROESY
1	-	135.5, C	-	-	-
2	6.86 (d, 1.7)	106.5, CH	-	1, 4, 6	7
3	-	147.8, C	-	-	-
4	-	147.2, C	-	-	-
5	6.88 (d, 8.0)	108.7, CH	6	1, 3, 4	-
6	6.83 (dd, 8.0, 1.7)	119.6, CH	5	2, 4, 7	8
7	5.46 (d, 6.0)	86.9, CH	8	1, 2, 6, 4′, 5′	2, 9a, 9b
8	3.34 (m)	54.0, CH	7, 9a, 9b	1, 4′, 5′	6
9a	3.97 (d, 9.8)	68.7, CH_2_	8, 9b	7, 8, 5′, 1'''	7, 1'''
9b	3.56 (m)		8, 9a	7, 8, 5′, 1'''	7, 1'''
1′	-	136.3, C	-	-	-
2′	6.70 (brs)	112.9, CH	-	-	3′- OCH_3_, 7′
3′	-	143.8, C	-	-	-
4′	-	145.8, C	-	-	-
5′	-	128.8, C	-	-	-
6′	6.72 (brs)	117.0, CH	-	-	-
7′a	2.61 (m)	31.8, CH_2_	8′a, 8′b	1′, 2′, 6′, 9′	2′, 9′a, 9′b
7′b	2.56 (m)		8′a, 8′b	1′, 2′, 6′, 9′	2′, 9′a, 9′b
8′a	1.81 (m)	31.8, CH_2_	7′, 9′a, 9′b	1′, 7′, 9′	-
8′b	1.76 (m)		7′, 9′a, 9′b	1′, 7′, 9′	-
9′a	3.73 (m)	68.5, CH_2_	8′, 9′b	7′, 8′, 1''	7′a, 7′b, 1''
9′b	3.41 (m)		8′, 9′a	7′, 8′, 1''	7′a, 7′b, 1''
3′-OCH_3_	3.77 (s)	56.1, CH_3_	-	3′	2′
3,4-OCH_2_O	6.00 (d, 0.9)	101.5, CH_2_	-	3, 4	-
	5.99 (d, 0.9)				
1''	4.11 (d, 7.9)	103.1, CH	2"	9′, 2", 5"	9′a, 9′b, 3", 5"
2''	2.98 (m)	73.9, CH	1", 3"	1", 4"	4"
3''	3.14 (m)	76.9, CH	2", 4"	4", 5"	1", 5"
4''	3.07 (m)	70.4, CH	3", 5"	3", 5", 6"	2"
5''	3.12 (m)	77.2, CH	4", 6"	3", 6"	1", 3"
6"a	3.70 (m)	63.6, CH_2_	5", 6"b	4"	-
6"b	3.42 (m)		5", 6"a	4"	-
1‴	4.26 (d, 7.9)	103.6, CH	2‴	9, 2‴, 5‴	9a, 9b, 3‴, 5‴
2‴	2.97 (m)	73.8, CH	1‴, 3‴	1‴, 4‴	4‴
3‴	3.29 (m)	76.0, CH	2‴, 4‴	4‴, 5‴	1‴, 5‴
4‴	3.04 (m)	70.3, CH	3‴, 5‴	3‴, 5‴, 6‴	2‴
5‴	3.06 (m)	77.0, CH	4‴, 6‴	3‴, 6‴	1‴, 3‴
6‴a	3.73 (m)	61.5, CH_2_	5‴, 6‴b	4‴	-
6‴b	3.57 (m)		5‴, 6‴a	4‴	-

a Spectra were recorded at 25 °C (800 MHz for ^1^H NMR and 200 MHz for ^13^C NMR).

7*R*,8*S*-Dihydrodehydrodiconiferyl alcohol (**2**): orange gum; ${\left[\alpha \right]}_{D}^{25}$ −8.6 (*c* 0.09, MeOH), lit. ${\left[\alpha \right]}_{D}^{25}$ −6.1 (*c* 0.99, MeOH) (Liu et al., 2018); LRESIMS *m/z* 383 [M+Na]^+^, 331 [M−CH_2_OH]^+^, 359 [M−H]^−^.

3′,4-*O*-Dimethylcedrusin (**3**): orange gum; ${\left[\alpha \right]}_{D}^{25}$ −4.9 (*c* 0.06, CHCl_3_), lit. ${\left[\alpha \right]}_{D}^{28}$, −8.52 (*c* 0.1, CHCl_3_) (Anantachoke et al., 2020); LRESIMS *m/z* 345 [M−CH_2_OH]^+^.

Suberifolioside A (**4**): pale-orange needles; mp 86–88 °C; ${\left[\alpha \right]}_{D}^{24}$ −62.5 (*c* 0.05, MeOH) (Wijesekera et al., 2025b); LRESIMS *m/z* 543 [M+Na]^+^, 565 [M + HCHO]^−^.

Equiselignan B (**5**): pale-orange needles; mp 80–83 °C; ${\left[\alpha \right]}_{D}^{24}$ −32.6 (*c* 0.03, MeOH), lit. ${\left[\alpha \right]}_{D}^{25}$ −12 (*c* 0.25, MeOH) (Zhu et al., 2022); LRESIMS *m/z* 329 [M−CH_2_OH]^+^, 381 [M+Na]^+^.

Egonol (**6**): white powder; LRESIMS *m/z* 327 [M+H]^+^.

### 2.5 Hydrolysis of suberifolioside B (**1**) and determination of absolute stereochemistry of sugars

Suberifolioside B (**1**, 2 mg) was hydrolysed with 2 M HCl in H_2_O–dioxane (1:1, 2 mL) at 80 °C for 6 h. The reaction mixture was diluted with H_2_O (10 mL) and extracted with CHCl_3_ (2 × 10 mL). The aqueous layer was neutralised with Ag_2_CO_3_ and analysed by optical rotation using D-glucose and L-glucose as reference standards. The aqueous hydrolysate exhibited ${\left[\alpha \right]}_{D}^{25}$ +18.4 (*c* 0.05, H_2_O), consistent with D-glucose (${\left[\alpha \right]}_{D}^{20}$ +40.4, *c* 0.1, H_2_O) and distinct from L-glucose (${\left[\alpha \right]}_{D}^{20}$ −37.2, *c* 0.05, H_2_O), indicating that both sugar units in **1** possessed D-configurations.

### 2.6 General preparation and purification of halogenated neolignans (**7**–**15**, **17**–**18**)

Halogenated derivatives were synthesised from scaffolds **4**, **5** and **16** using *N*-halosuccinimide reagents [*N*-chlorosuccinimide (NCS), *N*-bromosuccinimide (NBS) and *N*-iodosuccinimide (NIS)] in CH_2_Cl_2_ at room temperature for 1–4 h (Egbewande et al., 2023), with reaction progress monitored by LC–MS. To enable systematic comparison of halogen substitution patterns, chlorination, bromination and iodination reactions were applied under comparable conditions. Reaction mixtures were pre-adsorbed onto C_18_-bonded silica and purified by RP-HPLC using established conditions (Wijesekera et al., 2025b). Chlorination afforded compounds **7** and **12**, bromination yielded compounds **8**–**10**, **13**–**15**, **17** and **18**, and iodination afforded compounds **11** and **15**, with yields ranging from 3% to 54% and purities >95%.

All compounds were characterised by 1D and 2D NMR spectroscopy, UV and ECD analyses, and LRESIMS and HRESIMS measurements. Absolute configurations were assigned based on comparison with parent scaffolds and ECD data and confirmed for selected compounds by X-ray crystallography. Complete spectroscopic data for compounds **7**–**18** are provided in the Supplementary Information.

### 2.7 X-ray crystallography analysis of **5** and **15**

Intensity data were collected on a Rigaku XtalLAB Synergy diffractometer using Cu-Kα radiation at 100 K with an Oxford Cryostream cooling device. Structures were solved by direct methods and refined by difference Fourier synthesis (Sheldrick, 2015). Crystallographic data for compounds **5** and **15** have been deposited with the Cambridge Crystallographic Data Centre (CCDC 2473768 and 2473767) and can be obtained free of charge via http://www.ccdc.cam.ac.uk/data_request/cif.

## 3 Results

**Fig. 1:**
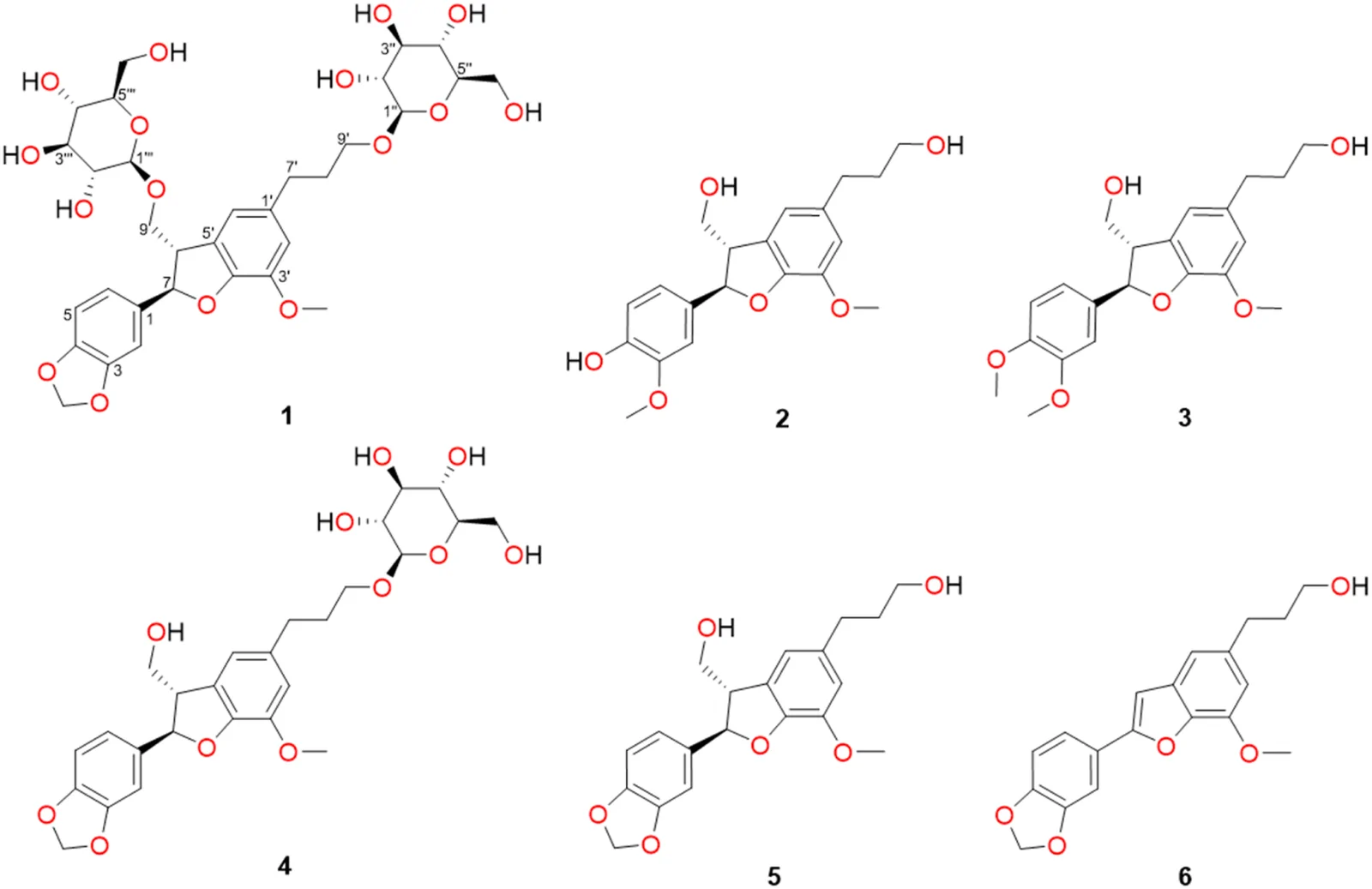
Chemical structures of natural compounds suberifolioside B (**1**), 7*R*,8*S*-dihydrodehydrodiconiferyl alcohol (**2**), 3′,4-*O*-dimethylcedrusin (**3**), suberifolioside A (**4**), equiselignan B (**5**) and egonol (**6**).

A dried fruit sample (20 g) of *S. suberifolius* was extracted using CH_2_Cl_2_:MeOH (4:1) followed by MeOH. Previously, we reported the purification and isolation of suberifolioside A (**4**), equiselignan B (**5**) and egonol (**6**) from the CH_2_Cl_2_:MeOH extract (Wijesekera et al., 2025b). To obtain sufficient quantities of **5** and additional amounts of **4** for halogenation studies, the MeOH extract was subjected to silica flash column chromatography to yield equiselignan B (**5**) together with two known compounds, 7*R*,8*S*-dihydrodehydrodiconiferyl alcohol (**2**) and 3′,4-*O*-dimethylcedrusin (**3**) (Liu et al., 2018; Anantachoke et al., 2020). A polar fraction from the silica flash column was further purified by RP-HPLC to afford the new neolignan glycoside, suberifolioside B (**1**), together with additional amounts of **4**, **5** and **6** (Fig. 1).

Suberifolioside B (**1**) was isolated as a stable brown gum. HRESIMS revealed an ion at *m/z* 705.2357 [M + Na]^+^ (calcd for C_32_H_42_O_16_Na, 705.2365), establishing the molecular formula. The ^1^H NMR spectrum (Table 1) in DMSO-*d*_6_ exhibited five aromatic proton resonances, signals for a methylenedioxy unit and an oxygenated methine proton at *δ*_H_ 5.46 (H-7). Additional resonances included two anomeric protons, a methoxy group, eight oxygenated methines, four oxygenated methylenes, one methine and two aliphatic methylenes, consistent with a glycosylated neolignan framework. The ^13^C NMR spectrum (Table 1) showed 32 carbon signals, including two phenyl rings, a methylenedioxy group, four oxygenated carbons and two hexose units.

**Fig. 2:**
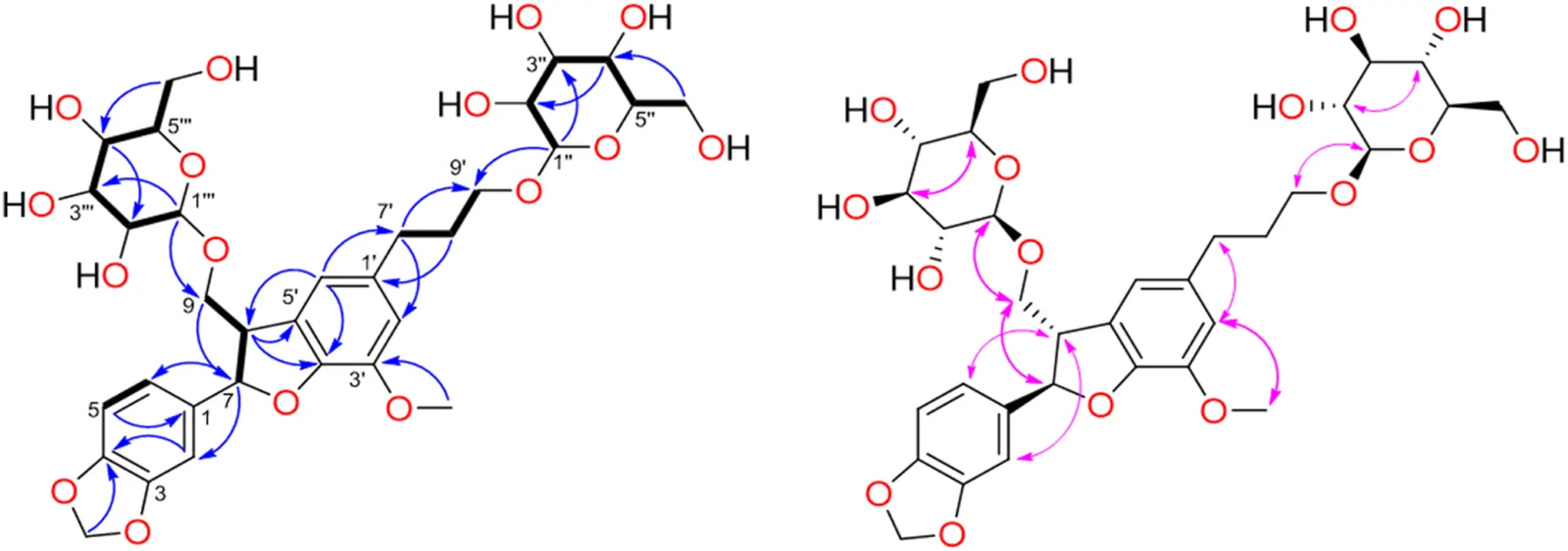
Key COSY (), HMBC () and ROESY () correlations for suberifolioside B (**1**).

^1^H–^1^H COSY data (Fig. 2) established five spin systems, including aromatic, aliphatic and sugar fragments. Key HMBC correlations from H-7 to C-2, C-6 and C-4′, from H-8 to C-4′ and C-6′, and from H_2_-7′ and H_2_-8′ to aromatic carbons defined the neolignan core structure. Additional HMBC correlations confirmed the attachment of two sugar units via C-9 and C-9′. HSQC and HMBC data further supported a structure identical to suberifolioside A (**4**) at the aglycone level. The coupling constant between H-7 and H-8 (*J* = 6.0 Hz) supported a trans configuration, consistent with literature values for related neolignans, and this assignment was reinforced by ROESY correlations. Following hydrolysis and comparison of optical rotation values with standards, the sugars of **1** were identified as *β*-D-glucopyranose units (Ohtani et al., 1992).

**Fig. 3:**
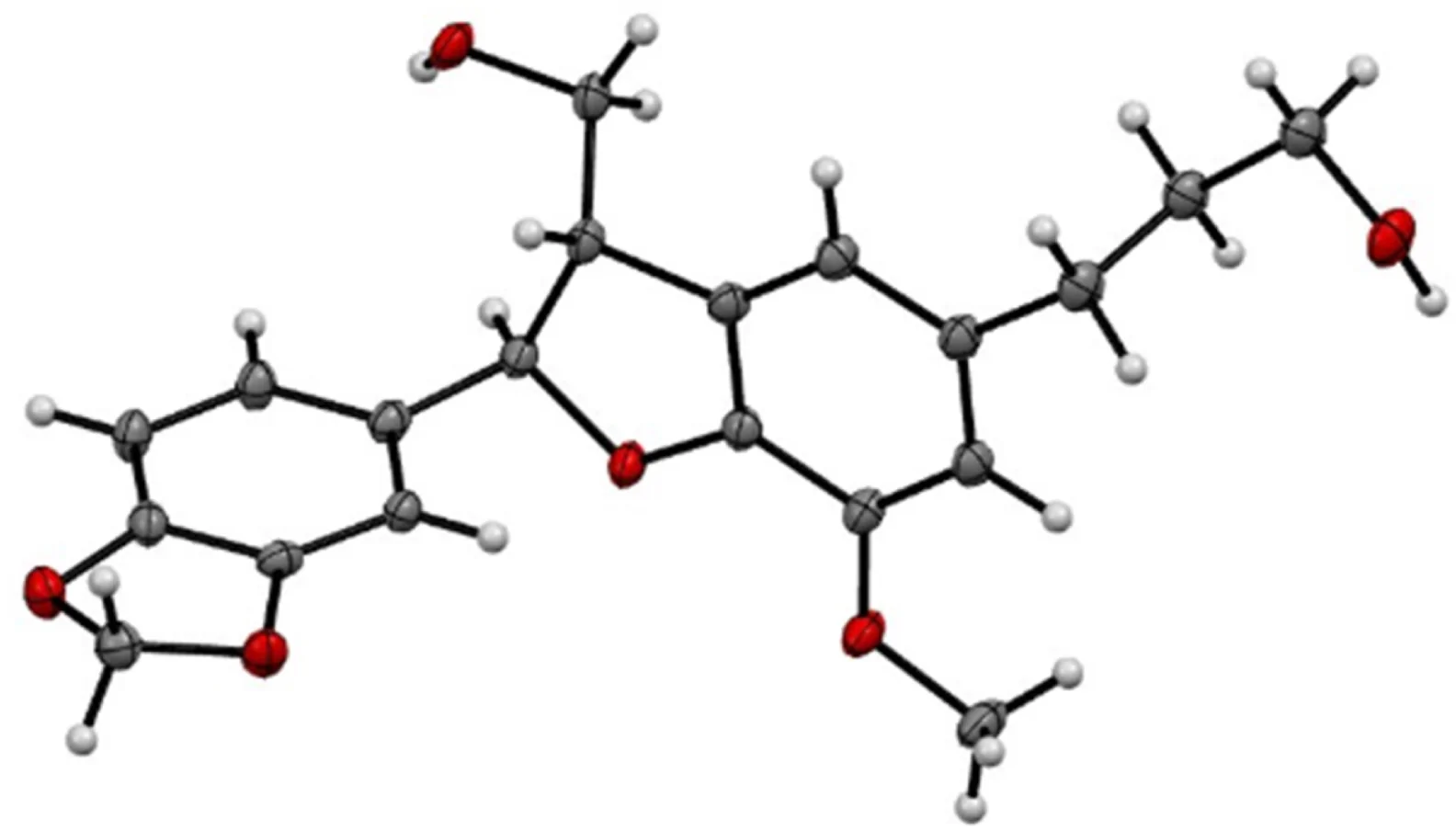
ORTEP of equiselignan B (**5**).

Slow evaporation of a methanolic solution of equiselignan B (**5**) yielded crystals suitable for X-ray diffraction analysis. The crystallographic data, acquired using Cu-Kα radiation, established the absolute configuration as 7*R* and 8*S* and the Oak Ridge Thermal Ellipsoid Plot (ORTEP) for compound **5** is shown in Fig. 3; these data confirmed the previous ECD assignment (Zhu et al., 2022). To our knowledge, X-ray crystallographic data for equiselignan B have not been reported previously.

**Fig. 4:**
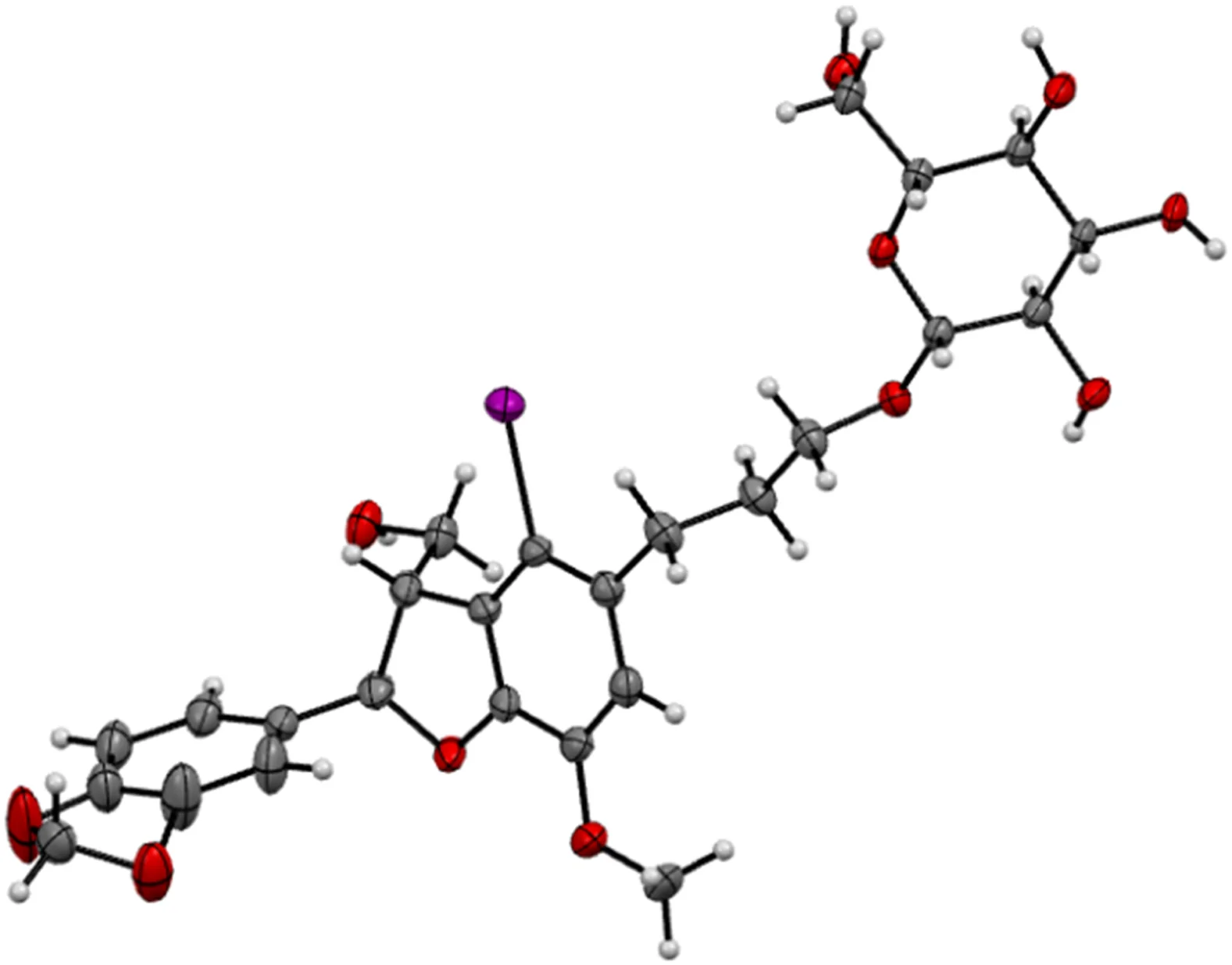
ORTEP of compound **15** with minor components of the disordered methylenedioxy moiety and the disordered water solvent molecules omitted for clarity.

**Scheme 1:**
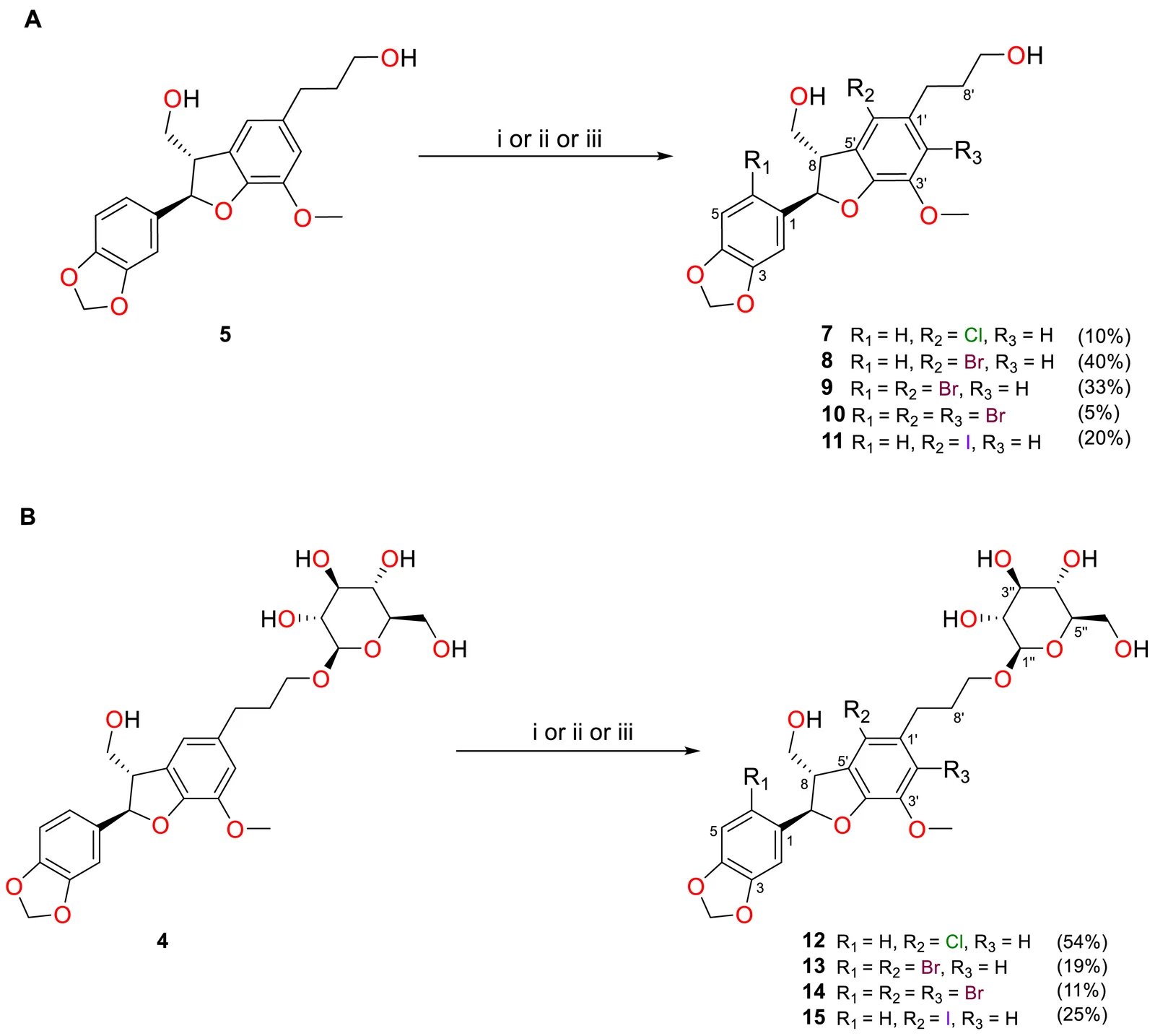
Chemical structures, reagents and conditions used to generate halogenated neolignans (**7***–***15**). (i) NCS, CH_2_Cl_2_, rt, 4 h; (ii) NBS, CH_2_Cl_2_, rt, 1 h; (iii) NIS, CH_2_Cl_2_, rt, 4 h; **A**: halogenated neolignans synthesised using compound **5**, **B**: halogenated neolignans synthesised using compound **4**.

**Scheme 2:**
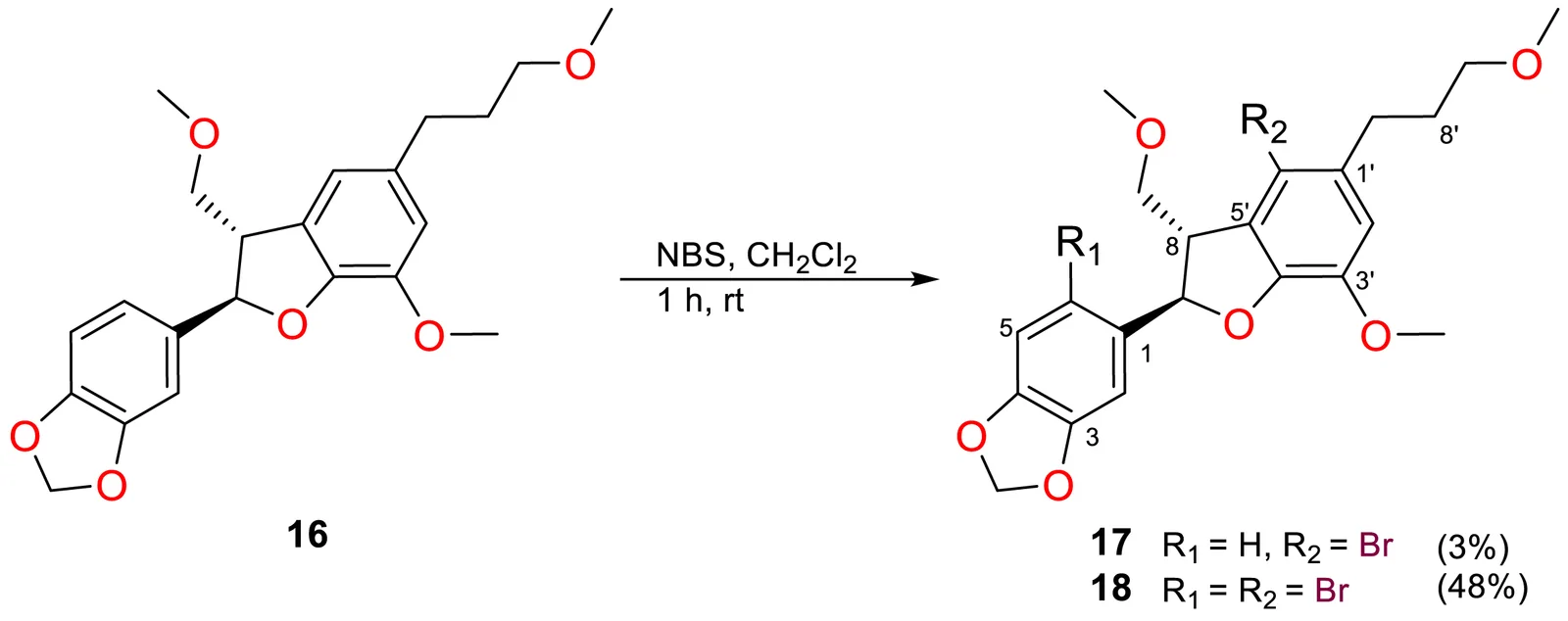
Conversion of compound **16** to the new brominated analogues **17** and **18**.

With sufficient quantities of **4** and **5** available, 11 new halogenated analogues were synthesised using aromatic halogenation with *N*-halosuccinimides (i.e. NBS, NCS and NIS) (Scheme 1). Reaction of **4** and **5** with NBS yielded five brominated derivatives (**8**–**10**, **13**–**14**) in yields of 5–40%, while NCS afforded compounds **7** and **12** in yields of 10% and 48%, respectively. Iodination using NIS produced compounds **11** and **15** in yields of 20% and 25%, respectively. In addition, the previously identified active ether analogue (**16**) was reacted with NBS to generate two new brominated analogues, **17** and **18**, in yields of 3% and 54%, respectively (Scheme 2). Overall, halogenation of **4**, **5** and **16** yielded 11 new derivatives (**7**–**15**, **17**–**18**) in yields of 3–54% and purities >95%, with structures confirmed by NMR, MS, ECD and, for selected compounds, X-ray crystallography. For example, HRESIMS analysis of compound **15** revealed an ion at *m/z* 669.0795 [M + Na]^+^ corresponding to C_26_H_31_IO_11_. Analysis of the ^1^H NMR spectrum of **15** in DMSO-*d*_6_ indicated that one aromatic broad singlet was missing compared to **4**, indicating the addition of one iodine atom at either C-2′ or C-6′. The positioning of the iodine in **15** was confirmed by strong HMBC correlations from *δ*_H_ 6.87 (H-2′) and 2.63 (H_2_-7′) to the shielded ^13^C NMR resonance at *δ*_C_ 86.5 (C-6′). Moreover, a methanolic solution of **15** yielded crystals appropriate for X-ray crystallography that enabled the absolute configuration of the molecule to be assigned as 7*R* and 8*S*, which was consistent with all our studies on this series of neolignans. See Fig. 4 for the ORTEP of compound **15**.

**Fig. 5:**
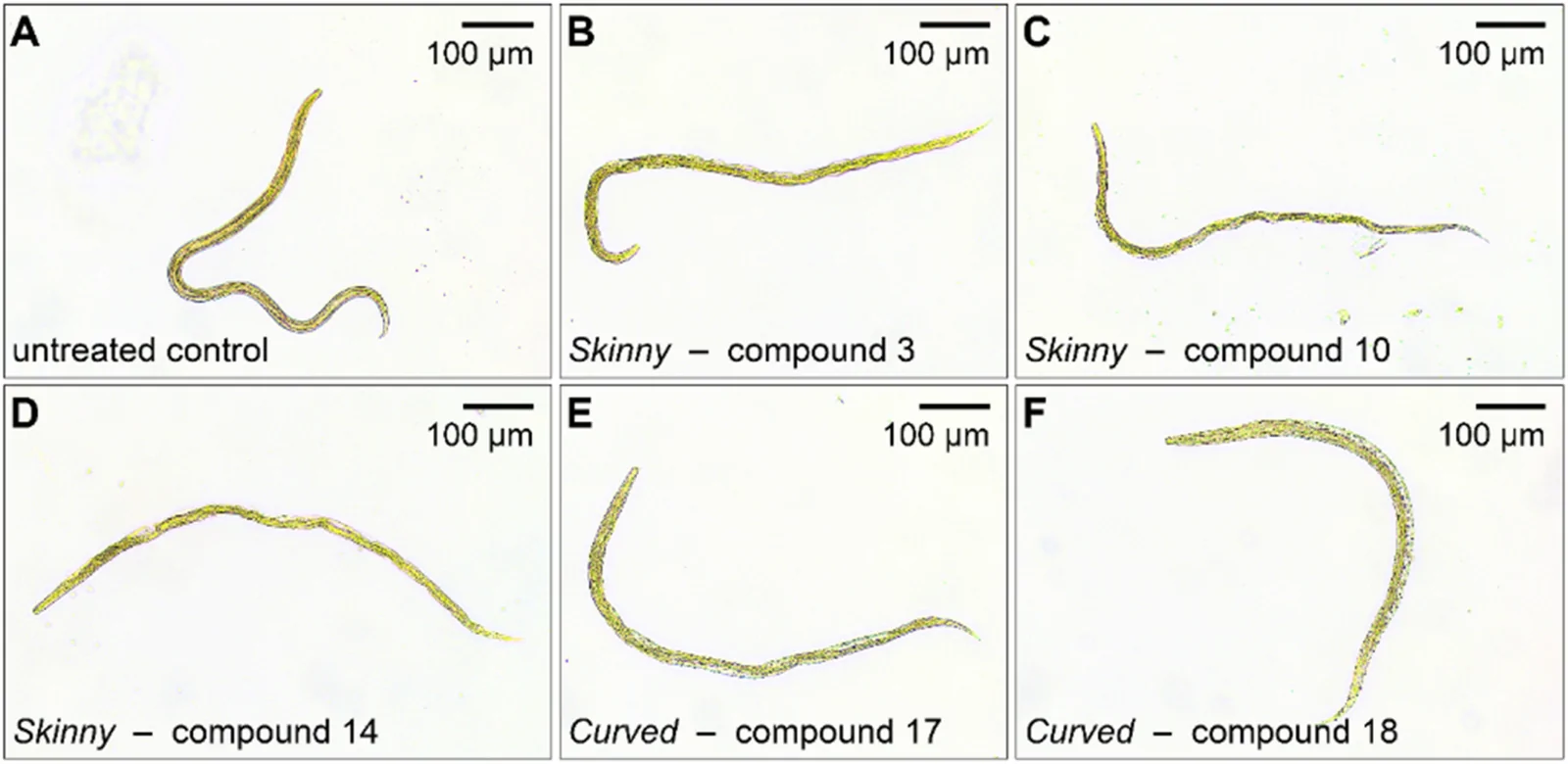
Representative light micrographs of *Haemonchus contortus* fourth-stage larvae (L4s) after incubation for 90 h in the absence of compound (untreated control; A) or with compounds **3** (B), **10** (C), **14** (D), **17** (E) or **18** (F) at 100 μM. The untreated control exhibits the wild-type (WT) phenotype; compounds **3**, **10** and **14** induced a skinny (*Ski*) phenotype, whereas compounds **17** and **18** induced a curved (*Cur*) phenotype.

All compounds were evaluated for anthelmintic activity against *H. contortus*. None of the compounds reduced xL3 motility at 100 μM after 168 h; however, compounds **17** and **18** induced curved phenotypes (10–21%), indicative of impaired development. In contrast, activity was observed against in vitro-raised L4s, with 10 of the 14 compounds inducing abnormal phenotypes (skinny or curved) at 90 h (Table 2; Fig. 5). Among the natural products, 3′,4-*O*-dimethylcedrusin (**3**) induced a skinny phenotype. Notably, tribrominated derivatives **10** and **14** achieved ≥50% reduction in L4 motility after 90 h, while monochlorinated compound **7** induced skinny phenotypes with 50.0 ± 15.6% maximal motility inhibition. The previously identified ether analogue **16** exhibited 73 ± 13.9% inhibition at 50 μM (Wijesekera et al., 2025b). The monobrominated ether analogue **17** showed the highest activity, with 79.6 ± 8.4% inhibition, comparable to moxidectin.

**Table 2:** Activity of neolignan natural products and halogenated analogues against L4s (90 h incubation) of *Haemonchus contortus*, with reference to two positive control compounds.

Compound	L4 Motility Relative IC_50_ (μM)^a^	L4 Motility MMI^b^ (%)	Abnormal phenotype^c^^,^^d^ (%)^e^
**1**	> 100	20.7 ± 18.3	Nil
**2**	> 100	0.0 ± 32.2	Nil
**3**	13.2 ± 3.8	48.1 ± 13.4	*Ski*/*Str* (50)
**4**	27.6 f	52.89 ± 14.0 f	Nil f
**5**	> 50 f	35.95 ± 21.1 f	Nil f
**6**	> 50 f	34.70 ± 9.2 f	Nil f
**7**	84.3 ± 16.8	50.0 ± 15.6	*Ski* (15)
**8**	0.7 ± 3.7	0.0 ± 14.1	Nil
**9**	0.4 ± 0.4	37.5 ± 17.6	*Ski* (35)
**10**	26.2 ± 3.0	46.9 ± 15.8	*Ski* (20)
**11**	> 100	27.1 ± 13.3	*Ski*/*Str* (14)
**12**	0.9 ± 10.9	33.7 ± 11.5	Nil
**13**	> 100	33.5 ± 10.3	Nil
**14**	12.6 ± 6.0	50.9 ± 15.8	*Ski* (21)
**15**	3.5 ± 2.7	29.2 ± 23.9	*Cur* (21)
**16**	24.43 f	72.62 ± 13.9 f	*Ski*/*Str *(80) f
**17**	6.7 ± 7.4	79.6 ± 8.4	*Cur* (60)
**18**	1.3 ± 3.4	29.3 ± 18.3	*Cur* (50)
Monepantel	0.3 ± 0.3	88.3 ± 14.0	Nil
Moxidectin	2.8 ± 4.7	76.0 ± 10.4	Nil

a IC_50_ calculated from three independent assay in triplicate.

b MMI = maximum motility inhibition (the highest inhibition level observed).

c Abnormal phenotypes described as *Cur* (curved)*, Str* (straight) and *Ski* (skinny) phenotypes.

d Nil indicates no abnormal phenotype.

e Population of affected larvae exhibiting abnormal phenotype(s) at 100 μM, %.

f Anthelmintic activity of compounds **4**–**6** and **16** were previously reported (Wijesekera et al., 2025b).

## 4 Discussion

Halogenation of neolignan scaffolds resulted in marked changes in anthelmintic activity against *H. contortus*, with clear stage-specific effects. None of the compounds significantly reduced xL3 motility, whereas multiple derivatives were active against L4s, underscoring the value of assessing compounds across developmental stages in phenotypic anthelmintic screening (Taki et al., 2021; Herath et al., 2022). This differential susceptibility may reflect physiological and developmental changes accompanying the in vitro transition from xL3s to L4s (Ma et al., 2019). Stage-dependent differences in compound uptake, metabolism, target abundance or target accessibility could therefore contribute to the greater activity observed against L4s. These possibilities cannot be resolved from the present data, but the results demonstrate that activity against one larval stage does not necessarily predict activity against another. Notably, compounds **17** and **18** induced curved phenotypes in a proportion of xL3s despite producing no significant reduction in xL3 motility, indicating that the absence of measurable motility inhibition does not exclude morphological or physiological effects that are not captured by motility alone. Distinct abnormal morphologies have previously been used as phenotypic indicators of compound-induced perturbation in *H. contortus* (see Jiao et al., 2019; Shanley et al., 2022). Here, curved phenotype may reflect altered neuromuscular or body-wall function (Nguyen et al., 2019), whereas the skinny phenotype may indicate impaired growth or an inability to maintain normal body dimensions (Zhang et al., 2021). These interpretations remain provisional because similar phenotypes can arise from different biological perturbations. Motility and morphology were assessed independently and did not correspond uniformly across compounds: some derivatives caused marked morphological abnormalities with modest motility inhibition, whereas others strongly reduced motility with limited morphological change. These phenotypic parameters therefore provide complementary information, and their incomplete correspondence may reflect differences in the timing, magnitude or biological basis of the compound-induced responses.

Structure–activity relationships revealed that halogenation modulated anthelmintic activity in a non-linear and scaffold-dependent manner. Although selected brominated equiselignan B derivatives were active, the monochlorinated analogue **7** achieved maximum motility inhibition comparable to that of the more heavily brominated compounds **10** and **14**, and iodination conferred no clear advantage. Activity therefore depended on the identity, position and extent of substitution rather than halogen number or size alone. These findings are consistent with the context-dependent effects of halogenation on molecular properties and biological interactions (Neumann et al., 2008; Wagner et al., 2009; Summerfield and Pattison, 2026). The observed differences may reflect changes in polarity, lipophilicity, conformation, membrane permeability or molecular interactions, although these properties were not examined directly. The modest activity of several glycosylated derivatives further suggests that the sugar moiety may influence bioactivity by altering polarity, permeability or intracellular availability. Mono-halogenation of the glycosylated scaffold was generally insufficient to produce strong motility inhibition, although some more extensively substituted analogues retained activity. Direct measurements of solubility, permeability and compound accumulation in larvae would be required to determine whether differential exposure contributed to this pattern (cf. Lanusse et al., 2016). Overall, the non-linear structure–activity relationship favours systematic exploration of substitution position and scaffold context over the simple addition of larger or more numerous halogens. Importantly, monobromination of the previously reported active ether analogue **16** (Wijesekera et al., 2025b) yielded compound **17**, which produced the greatest maximum motility inhibition among the derivatives examined. Relative to compound **16**, compound **17** had a lower IC_50_, consistent with improved concentration-dependent activity. In contrast, the more extensively brominated analogue **18** produced substantially lower maximum motility inhibition despite also inducing a curved phenotype, indicating that additional bromination was not inherently beneficial. Compound **17** achieved maximum motility inhibition numerically similar to that observed for moxidectin under the same in vitro assay conditions; however, its higher mean IC_50_ limits this comparison to maximal effect and does not establish equivalent potency. This activity profile identifies the ether-containing scaffold as a rational starting point for further optimisation and supports the use of whole-organism phenotypic screening to guide targeted modification of natural-product frameworks (Herath et al., 2022; Taki et al., 2021).

The combined motility and morphological responses can guide the prioritisation of compounds for mechanistic studies but do not identify a specific molecular target or mechanism of action. The phenotypes are compatible with perturbation of neuromuscular function, growth and development, or body-wall and cuticular integrity, although they may also reflect secondary effects of broader physiological impairment. These possibilities remain hypotheses, as phenotype alone cannot distinguish a primary effect on these processes from a secondary consequence of general physiological disruption. Resolving them will require complementary approaches, including time-resolved phenotyping, ultrastructural examination, molecular profiling and target-engagement or target-deconvolution studies (Shanley et al., 2025). Evaluation against additional developmental stages, including adult worms, would also help determine whether the activity observed against L4s extends to later parasitic stages. Such assays require distinct ex vivo or in vivo approaches and were beyond the scope of this initial structure–activity study. The paired xL3 and in vitro-raised L4 assays nevertheless provide a tractable platform for detecting stage-specific activity while limiting the need for additional animal use (Taki et al., 2020; Thilakarathne et al., 2025). The contrasting susceptibilities observed here reinforce the value of evaluating compounds across multiple developmental stages during phenotypic anthelmintic discovery.

Collectively, these findings demonstrate that halogenation can modulate and, for selected derivatives, enhance the anthelmintic activity of neolignan scaffolds. In particular, the activity profile of compound **17** identifies the ether-containing scaffold and its monobrominated substitution pattern as a rational starting point for further optimisation. Integrating systematic medicinal chemistry with stage-resolved phenotypic analysis, physicochemical characterisation and mechanism-of-action studies will be necessary to assess the translational potential of this compound class.

## Ethics approval

This study was conducted in accordance with the institutional animal ethics guidelines (permit no. 23983; The University of Melbourne).

## CRediT authorship contribution statement

**Kanchana Wijesekera:** Data curation, Formal analysis, Investigation, Software, Validation, Writing – original draft, Writing – review & editing. **Aya C. Taki:** Data curation, Investigation, Validation, Writing – review & editing. **Joseph J. Byrne:** Data curation, Validation, Visualization. **Jonathan M. White:** Formal analysis, Validation, Visualization. **Anthony R. Carroll:** Supervision, Writing – review & editing. **Robin B. Gasser:** Conceptualization, Data curation, Funding acquisition, Project administration, Resources, Validation, Writing – review & editing. **Rohan A. Davis:** Conceptualization, Data curation, Formal analysis, Funding acquisition, Project administration, Resources, Supervision, Validation, Writing – review & editing.

## Declaration of competing interest

The authors declare that they have no known competing financial interests or personal relationships that could have appeared to influence the work reported in this paper.
