# Supplementary material for: Halogenation of neolignan scaffolds reveals stage-specific anthelmintic activity against Haemonchus contortus
Source: Int J Parasitol Drugs Drug Resist. 2026 Aug 10;32:100666. doi: 10.1016/j.ijpddr.2026.100666 (PMC13545404; doi:10.1016/j.ijpddr.2026.100666)
Supplement: Supplementary file 1 [file mmc1.pdf]

Supporting Information

Halogenation of neolignan scaffolds reveals stage-specific anthelmintic activity against *Haemonchus contortus*

Kanchana Wijesekera,[a, b] Aya C. Taki,[c] Joseph J. Byrne,[c] Jonathan M. White,[d] Anthony R. Carroll,[e] Robin B. Gasser,[c, *] Rohan A. Davis,[a, b, f, *]

[a] Institute for Biomedicine and Glycomics, Griffith University, Brisbane, QLD 4111, Australia

[b] School of Environment and Science, Griffith University, Brisbane, QLD 4111, Australia

[c] Department of Veterinary Biosciences, Melbourne Veterinary School, Faculty of Science, The University of Melbourne, Parkville, VIC 3010, Australia

[d] School of Chemistry and Bio21 Institute, The University of Melbourne, Melbourne, VIC 3010, Australia

[e] School of Environment and Science, Griffith University, Gold Coast, QLD 4222, Australia

[f] NatureBank, Griffith University, Brisbane, QLD 4111, Australia

**Corresponding authors*. robinbg@unimelb.edu.au (R.B. Gasser); r.davis@griffith.edu.au (R.A. Davis)

Table of Contents

X-ray crystallography analysis of **5** and **15** 5

NMR and MS data for compounds **7** – **18** 5

Figure S1 ^{1}H NMR (800 MHz) spectrum of suberifolioside B (**1**) in DMSO-d_6 12
Figure S2 ^{13}C NMR (200 MHz) spectrum of suberifolioside B (**1**) in DMSO-d_6 12
Figure S3 COSY spectrum of suberifolioside B (**1**) in DMSO-d_6 13
Figure S4 HSQC spectrum of suberifolioside B (**1**) in DMSO-d_6 13
Figure S5 HMBC spectrum of suberifolioside B (**1**) in DMSO-d_6 14
Figure S6 ROESY spectrum of suberifolioside B (**1**) in DMSO-d_6 14
Figure S7 ^{1}H NMR (800 MHz) spectrum of 7*R*,8*S*-dihydrodehydrodiconiferyl alcohol (**2**) in DMSO-d_6 15
Figure S8 ^{13}C NMR (200 MHz) spectrum of 7*R*,8*S*-dihydrodehydrodiconiferyl alcohol (**2**) in DMSO-d_6 15
Figure S9 ^{1}H NMR (800 MHz) spectrum of 3',4-*O*-dimethylcedrusin (**3**) in DMSO-d_6 16
Figure S10 ^{13}C NMR (200 MHz) spectrum of 3',4-*O*-dimethylcedrusin (**3**) in DMSO-d_6 16
Figure S11 ^{1}H NMR (800 MHz) spectrum of suberifolioside A (**4**) in DMSO-d_6 17
Figure S12 ^{13}C NMR (200 MHz) spectrum of suberifolioside A (**4**) in DMSO-d_6 17
Figure S13 ^{1}H NMR (800 MHz) spectrum of equiselignan B (**5**) in DMSO-d_6 18
Figure S14 ^{13}C NMR (200 MHz) spectrum of equiselignan B (**5**) in DMSO-d_6 18
Figure S15 ^{1}H NMR (800 MHz) spectrum of egonol (**6**) in DMSO-d_6 19
Figure S16 ^{13}C NMR (200 MHz) spectrum of egonol (**6**) in DMSO-d_6 19
Figure S17 ^{1}H NMR (800 MHz) spectrum of compound **7** in DMSO-d_6 20
Figure S18 ^{13}C NMR (200 MHz) spectrum of compound **7** in DMSO-d_6 20
Figure S19 COSY spectrum of compound **7** in DMSO-d_6 21
Figure S20 HSQC spectrum of compound **7** in DMSO-d_6 21
Figure S21 HMBC spectrum of compound **7** in DMSO-d_6 22
Figure S22 ROESY spectrum of compound **7** in DMSO-d_6 22
Figure S23 ^{1}H NMR (800 MHz) spectrum of compound **8** in DMSO-d_6 23
Figure S24 ^{13}C NMR (200 MHz) spectrum of compound **8** in DMSO-d_6 23
Figure S25 COSY spectrum of compound **8** in DMSO-d_6 24
Figure S26 HSQC spectrum of compound **8** in DMSO-d_6 24
Figure S27 HMBC spectrum of compound **8** in DMSO-d_6 25
Figure S28 ROESY spectrum of compound **8** in DMSO-d_6 25
Figure S29 ^{1}H NMR (800 MHz) spectrum of compound **9** in DMSO-d_6 26
Figure S30 ^{13}C NMR (200 MHz) spectrum of compound **9** in DMSO-d_6 26
Figure S31 COSY spectrum of compound **9** in DMSO-d_6 27
Figure S32 HSQC spectrum of compound **9** in DMSO-d_6 27
Figure S33 HMBC spectrum of compound **9** in DMSO-d_6 28
Figure S34 ROESY spectrum of compound **9** in DMSO-d_6 28
Figure S35 ^{1}H NMR (800 MHz) spectrum of compound **10** in DMSO-d_6 29
Figure S36 ^{13}C NMR (200 MHz) spectrum of compound **10** in DMSO-d_6 29
Figure S37 COSY spectrum of compound **10** in DMSO-d_6 30
Figure S38 HSQC spectrum of compound **10** in DMSO-d_6 30
Figure S39 HMBC spectrum of compound **10** in DMSO-d_6 31
Figure S40 ROESY spectrum of compound **10** in DMSO-d_6 31
Figure S41 ^{1}H NMR (800 MHz) spectrum of compound **11** in DMSO-d_6 32
Figure S42 ^{13}C NMR (200 MHz) spectrum of compound **11** in DMSO-d_6 32
Figure S43 COSY spectrum of compound **11** in DMSO-d_6 33

Figure S44 HSQC spectrum of compound **11** in DMSO-d_6 33
Figure S45 HMBC spectrum of compound **11** in DMSO-d_6 34
Figure S46 ROESY spectrum of compound **11** in DMSO-d_6 34
Figure S47 ^{1}H NMR (800 MHz) spectrum of compound **12** in DMSO-d_6 35
Figure S48 ^{13}C NMR (200 MHz) spectrum of compound **12** in DMSO-d_6 35
Figure S49 COSY spectrum of compound **12** in DMSO-d_6 36
Figure S50 HSQC spectrum of compound **12** in DMSO-d_6 36
Figure S51 HMBC spectrum of compound **12** in DMSO-d_6 37
Figure S52 ROESY spectrum of compound **12** in DMSO-d_6 37
Figure S53 ^{1}H NMR (800 MHz) spectrum of compound **13** in DMSO-d_6 38
Figure S54 ^{13}C NMR (200 MHz) spectrum of compound **13** in DMSO-d_6 38
Figure S55 COSY spectrum of compound **13** in DMSO-d_6 39
Figure S56 HSQC spectrum of compound **13** in DMSO-d_6 39
Figure S57 HMBC spectrum of compound **13** in DMSO-d_6 40
Figure S58 ROESY spectrum of compound **13** in DMSO-d_6 40
Figure S59 ^{1}H NMR (800 MHz) spectrum of compound **14** in DMSO-d_6 41
Figure S60 ^{13}C NMR (200 MHz) spectrum of compound **14** in DMSO-d_6 41
Figure S61 COSY spectrum of compound **14** in DMSO-d_6 42
Figure S62 HSQC spectrum of compound **14** in DMSO-d_6 42
Figure S63 HMBC spectrum of compound **14** in DMSO-d_6 43
Figure S64 ROESY spectrum of compound **14** in DMSO-d_6 43
Figure S65 ^{1}H NMR (800 MHz) spectrum of compound **15** in DMSO-d_6 44
Figure S66 ^{13}C NMR (200 MHz) spectrum of compound **15** in DMSO-d_6 44
Figure S67 COSY spectrum of compound **15** in DMSO-d_6 45
Figure S68 HSQC spectrum of compound **15** in DMSO-d_6 45
Figure S69 HMBC spectrum of compound **15** in DMSO-d_6 46
Figure S70 ROESY spectrum of compound **15** in DMSO-d_6 46
Figure S71 ^{1}H NMR (800 MHz) spectrum of compound **16** in DMSO-d_6 47
Figure S72 ^{13}C NMR (200 MHz) spectrum of compound **16** in DMSO-d_6 47
Figure S73 ^{1}H NMR (800 MHz) spectrum of compound **17** in DMSO-d_6 48
Figure S74 ^{13}C NMR (200 MHz) spectrum of compound **17** in DMSO-d_6 48
Figure S75 COSY spectrum of compound **17** in DMSO-d_6 49
Figure S76 HSQC spectrum of compound **17** in DMSO-d_6 49
Figure S77 HMBC spectrum of compound **17** in DMSO-d_6 50
Figure S78 ROESY spectrum of compound **17** in DMSO-d_6 50
Figure S79 ^{1}H NMR (800 MHz) spectrum of compound **18** in DMSO-d_6 51
Figure S80 ^{13}C NMR (200 MHz) spectrum of compound **18** in DMSO-d_6 51
Figure S81 COSY spectrum of compound **18** in DMSO-d_6 52
Figure S82 HSQC spectrum of compound **18** in DMSO-d_6 52
Figure S83 HMBC spectrum of compound **18** in DMSO-d_6 53
Figure S84 ROESY spectrum of compound **18** in DMSO-d_6 53
Figure S85 ECD data for suberifolioside B (**1**) 54
Figure S86 ECD data for 7*R*,8*S*-dihydrodehydrodiconiferyl alcohol (**2**), 3',4-*O*-dimethylcedrusin (**3**), suberifolioside A (**4**), equiselignan B (**5**) 54
Figure S87 ECD data for the compounds **7** and **12** 54
Figure S88 ECD data for the compounds **8–10**, **13** and **14** 55
Figure S89 ECD data for the compounds **11** and **15** 55
Figure S90 ECD data for the compounds **16–18** 55
Figure S91 LC-MS data of suberifolioside B (**1**) 56
Figure S92 HRESIMS data of suberifolioside B (**1**) 57

Figure S93 LC-MS data of 7*R*,8*S*-dihydrodehydrodiconiferyl alcohol (**2**) 58
Figure S94 LC-MS data of 3',4-*O*-dimethylcedrusin (**3**) 59
Figure S95 LC-MS data of suberifolioside A (**4**) 60
Figure S96 LC-MS data of equiselignan B (**5**) 61
Figure S97 LC-MS data of egonol (**6**) 62
Figure S98 LC-MS data of compound **7** 63
Figure S99 HRESIMS data of compound **7** 64
Figure S100 LC-MS data of compound **8** 65
Figure S101 HRESIMS data of compound **8** 66
Figure S102 LC-MS data of compound **9** 67
Figure S103 HRESIMS data of compound **9** 68
Figure S104 LC-MS data of compound **10** 69
Figure S105 HRESIMS data of compound **10** 70
Figure S106 LC-MS data of compound **11** 71
Figure S107 HRESIMS data of compound **11** 72
Figure S108 LC-MS data of compound **12** 73
Figure S109 HRESIMS data of compound **12** 74
Figure S110 LC-MS data of compound **13** 75
Figure S111 HRESIMS data of compound **13** 76
Figure S112 LC-MS data of compound **14** 77
Figure S113 HRESIMS data of compound **14** 78
Figure S114 LC-MS data of compound **15** 79
Figure S115 HRESIMS data of compound **15** 80
Figure S116 LC-MS data of compound **16** 81
Figure S117 LC-MS data of compound **17** 82
Figure S118 HRESIMS data of compound **17** 83
Figure S119 LC-MS data of compound **18** 84
Figure S120 HRESIMS data of compound **18** 85

X-ray crystallography analysis of 5 and 15

Crystal data for equiselignan B (**5**): $C_{20}H_{22}O_6$, M = 358.37, *T* = 100.0 K, □□= *1.54184 Å,* monoclinic, space group *P* 2_1, *a* = 9.4796(3) Å, *b* = 4.8886(2) Å, *c* = 18.4577(6) Å, □ = 93.862(3)°, *V* = 853.42(5), *Å*^{3}, *Z* = 2, D_c = 1.395 mg M^{-3} μ (Cu-Kα) 0.852 *mm*$^{-1}$, F(000) = 380 crystal size 0.15×0.06×0.01 mm^3; 8699 reflections measured θ_{max} = 79.485°, 5265 independent reflections [R_{int} = 0.061]; final R = 0.0439, [$I > 2\sigma(I)$, 3261 data] and wR(F^2) = 0.1208 [all data]; GOOF 1.070, absolute structure parameter 0.12(13).

Crystal data for compound **15**: $C_{26}H_{33}IO_{12}$, *M* = 664.42, *T* = 100.0 K, □ =1.54184 Å, orthorhombic, space group *P* 2_12_12, *a* = 8.41290(10) Å, *b* = 22.4443(2) Å, *c* = 14.07490(10) Å, *V* = 2657.65(4) Å^3, *Z* = 4, D_c = 1.661 mg M^{-3} μ (Cu-Kα) 10.040 mm^{-1}, F(000) = 1352 crystal size 0.47×0.07×0.07 mm^3, 83824 reflections measured θ_{max} = 79.586°, 5748 independent reflections [R_{int} = 0.097], the final R was 0.0335 [$I > 2s(I)$, 5669 data] and *w*R(F^2) = 0.0900 (all data); GOOF 1.070, absolute structure parameter -0.014(2).

NMR and MS data for compounds 7 – 18

Compound 7: Colourless gum (2.0 mg, 10%); $[\alpha]_D^{25}$ –94 (*c* 0.05, MeOH); UV (MeOH) λ_{max} (log ε) 211 (0.51), 234 (0.42), 272 (0.31) nm; ECD (MeOH) λ_{ext} 210 nm ($\Delta\varepsilon$ +19.9), 227 nm ($\Delta\varepsilon$ –10.4), 243 nm ($\Delta\varepsilon$ +24.1), 295 nm ($\Delta\varepsilon$ –41.0); ^{1}H NMR (DMSO-d_6, 800 MHz) δ_H 6.88 (1H, d, *J* = 8.0 Hz, H-5), 6.86 (1H, s, H-2'), 6.76 (1H, brs, H-2), 6.75 (1H, dd, *J* = 8.0 Hz, 0.6 Hz, H-6), 6.00 (1H, d, *J* = 1.0 Hz, OCH_2O), 5.99 (1H, d, *J* = 1.0 Hz, OCH_2O), 5.70 (1H, d, *J* = 3.4 Hz, H-7), 3.88 (1H, dd, *J* = 10.7, 3.6 Hz, H-9a), 3.80 (3H, s, OCH_3-3'), 3.55 (1H, dd, *J* = 10.7, 3.6 Hz, H-9b), 3.43 (2H, t, *J* = 6.5 Hz, H-9'a, H-9'b), 3.43 (1H, m, H-8), 2.63 (1H, m, H-7'a), 2.61 (1H, m, H-7'b), 1.68 (1H, m, H-8'a), 1.66 (1H, m, H-8'b). ^{13}C NMR (DMSO-d_6, 200 MHz) δ_C 147.5 (C-3), 146.8 (C-4), 146.5 (C-4'), 142.6 (C-3'), 135.7 (C-1) 132.1 (C-1'), 125.3 (C-5'), 120.2 (C-6'), 118.3 (C-6), 114.6 (C-2'), 108.3 (C-5), 105.4 (C-2), 101.1 (OCH_2O), 86.1

(C-7), 61.0 (C-9'), 60.2 (C-9), 56.0 (OCH_3-3'), 54.7 (C-8), 33.1 (C-8'), 29.1 (C-7'); LRESIMS *m/z* 415 $[M + Na]^+$; HRESIMS *m/z* 415.0919 $[M + Na]^+$ (calcd for $C_{20}H_{21}{}^{35}ClNaO_6$, 415.0919).

Compound 8: Yellow gum (8.0 mg, 40%); $[\alpha]_D^{25}$ –33 (*c* 0.15, MeOH); UV (MeOH) λ_{max} (log ε) 209 (1.9), 242 (0.5), 293 (0.2) nm; ECD (MeOH) λ_{ext} 210 nm ($\Delta\varepsilon$ +19.9), 227 nm ($\Delta\varepsilon$ –10.4), 243 nm ($\Delta\varepsilon$ +24.1), 295 nm ($\Delta\varepsilon$ –41.0); ^1H NMR (DMSO-d_6, 800 MHz) δ_H 6.92 (1H, s, H-2'), 6.88 (1H, d, J = 8.5 Hz, H-5), 6.75 (1H, dd, J = 8.5 Hz, 1.2 Hz, H-6), 6.74 (1H, d, J = 1.2 Hz, H-2), 5.99 (1H, d, J = 1.0 Hz, OCH_2O), 5.98 (1H, d, J = 1.0 Hz, OCH_2O), 5.72 (1H, d, J = 3.1 Hz, H-7), 4.41 (2H, t, J = 6.4 Hz, H-9'a, H-9'b), 3.91 (1H, m, H-9a), 3.80 (3H, s, OCH_3-3'), 3.52 (1H, m, H-9b), 3.36 (1H, m, H-8), 2.71 (1H, m, H-7'a), 2.70 (1H, m, H-7'b), 1.98 (1H, m, H-8'a), 1.96 (1H, m, H-8b'). ^{13}C NMR (DMSO-d_6, 200 MHz) δ_C 147.5 (C-3), 146.9 (C-4), 146.6 (C-4'), 143.4 (C-3'), 135.7 (C-1) 132.4 (C-1'), 127.5 (C-5'), 118.2 (C-6), 114.6 (C-2'), 110.3 (C-6'), 108.3 (C-5), 105.3 (C-2), 101.1 (OCH_2O), 85.8 (C-7), 67.9 (C-9'), 61.1 (C-9), 56.0 (OCH_3-3'), 56.0 (C-8), 30.9 (C-7'), 28.2 (C-8'); LRESIMS *m/z* 459 $[M(^{79}Br)+Na]^+$, 461 $[M(^{81}Br)+Na]^+$; HRESIMS *m/z* 459.0403 $[M+Na]^+$ (calcd for $C_{20}H_{21}{}^{79}BrNaO_6$, 459.0414).

Compound 9: Brown gum (6.6 mg, 33%); $[\alpha]_D^{25}$ –64.3 (*c* 0.09, MeOH); UV (MeOH) λ_{max} (log ε) 211 (0.33), 233 (0.54), 270 (0.20) nm; ECD (MeOH) λ_{ext} 215 nm ($\Delta\varepsilon$ –19.5), 297 nm ($\Delta\varepsilon$ –8.9); ^1H NMR (DMSO-d_6, 800 MHz) δ_H 7.26 (1H, s, H-5), 6.90 (1H, s, H-2'), 6.54 (1H, s, H-2), 6.05 (1H, d, J = 0.9 Hz, OCH_2O), 6.00 (1H, d, J = 0.9 Hz, OCH_2O), 5.91 (1H, d, J = 2.9 Hz, H-7), 3.94 (1H, dd, J = 10.8, 3.4 Hz, H-9a), 3.82 (1H, m, H-9b), 3.81 (3H, s, OCH_3-3'), 3.44 (2H, m, H-9'a, H-9'b), 3.30 (1H, m, H-8), 2.65 (1H, m, H-7'a), 2.63 (1H, m, H-7'b), 1.69 (1H, m, H-8'a), 1.67 (1H, m, H-8'b). ^{13}C NMR (DMSO-d_6, 200 MHz) δ_C 148.1 (C-3), 147.6 (C-4), 146.4 (C-4'), 143.1 (C-3'), 133.5 (C-1), 134.2 (C-1'), 127.7 (C-5'), 114.5 (C-2'), 113.0 (C-5), 111.4 (C-6), 110.3 (C-6'), 106.1 (C-2), 102.3 (OCH_2O), 84.9 (C-7), 61.1 (C-9'), 60.3 (C-9), 56.0 (OCH_3-3'), 54.9 (C-8), 33.3 (C-8'), 31.6 (C-7'); LRESIMS *m/z* 537 $[M(^{79}Br)+Na]^+$, 539 $[M(^{81}Br)+Na]^+$; HRESIMS *m/z* 536.9513 $[M+Na]^+$ (calcd for $C_{20}H_{20}{}^{79}Br_2NaO_6$, 536.9519).

Compound 10: Brown gum (1.0 mg, 5%); $[\alpha]_D^{25}$ –33.1 (*c* 0.1, MeOH); UV (MeOH) λ_{max} (log ε) 208 (17.5), 239 (4.2), 293 (0.08) nm; ECD (MeOH) λ_{ext} 217 nm ($\Delta\varepsilon$ +24.2), 246 nm ($\Delta\varepsilon$ +7.1), 273 nm ($\Delta\varepsilon$ +2.8), 299 nm ($\Delta\varepsilon$ –17.6); ^{1}H NMR (DMSO-d_6, 800 MHz) δ_H 7.29 (1H, s, H-5), 6.63 (1H, s, H-2), 6.06 (1H, d, *J* = 1.0 Hz, OCH_2O), 6.02 (1H, d, *J* = 1.0 Hz, OCH_2O), 6.01 (1H, d, *J* = 3.4 Hz, H-7), 3.91 (2H, m, H-9a, H-9b), 3.84 (3H, s, OCH_3-3'), 3.47 (2H, t, *J* = 6.4 Hz, H-9'a, H-9'b), 3.35 (1H, m, H-8), 2.89 (1H, m, H-7'a), 2.87 (1H, m, H-7'b), 1.61 (1H, m, H-8'a), 1.59 (1H, m, H-8'b). ^{13}C NMR (DMSO-d_6, 200 MHz) δ_C149.5 (C-3), 148.3 (C-4), 147.6 (C-4'), 139.9 (C-3'), 133.2 (C-1'), 132.9 (C-1), 129.0 (C-5'), 118.0 (C-6'), 114.0 (C-2'), 113.0 (C-6), 111.5 (C-5), 106.3 (C-2), 102.3 (OCH_2O), 85.9 (C-7), 60.7 (C-9'), 60.5 (C-9), 59.9 (OCH_3-3'), 54.8 (C-8) 33.0 (C-7'), 31.7 (C-8'); LRESIMS *m/z* 615 [M(^{79}Br)+Na]$^+$, 617 [M(^{81}Br)+Na]$^+$; HRESIMS *m/z* 614.8597 [M+Na]$^+$ (calcd for $C_{20}H_{19}{}^{79}Br_3NaO_6$, 614.8624).

Compound 11: Colourless gum (4.0 mg, 20%); $[\alpha]_D^{25}$ –152.5 (*c* 0.08, MeOH); UV (MeOH) λ_{max} (log ε) 211 (0.35), 233 (0.52), 272 (0.23) nm; ECD (MeOH) λ_{ext} 201 nm ($\Delta\varepsilon$ +13.6), 219 nm ($\Delta\varepsilon$ –80.0), 247 nm ($\Delta\varepsilon$ +8.1); ^{1}H NMR (DMSO-d_6, 800 MHz) δ_H 6.88 (1H, d, *J* = 7.4 Hz, H-5), 6.87 (1H, s, H-2'), 6.73 (1H, dd, *J* = 7.4 Hz,, 0.5 Hz, H-6), 6.72 (1H, d, *J* = 0.5 Hz, H-2), 5.99 (1H, d, *J* = 1.0 Hz, OCH_2O), 5.98 (1H, d, *J* = 1.0 Hz, OCH_2O), 5.73 (1H, d, *J* = 2.8 Hz, H-7), 3.93 (1H, dd, *J* = 10.7, 3.0 Hz, H-9a), 3.80 (3H, s, OCH_3-3'), 3.44 (2H, m, H-9'a, H-9'b), 3.43 (1H, m, H-9b), 3.22 (1H, m, H-8), 2.64 (1H, m, H-7'a), 2.61 (1H, m, H-7'b), 1.65 (1H, m, H-8'a), 1.63 (1H, m, H-8'b). ^{13}C NMR (DMSO-d_6, 200 MHz) δ_C 147.5 (C-3), 146.8 (C-4), 145.2 (C-4'), 144.0 (C-3'), 137.3 (C-1'), 135.8 (C-1), 131.3 (C-5'), 118.0 (C-6), 113.7 (C-2'), 108.4 (C-5), 105.2 (C-2), 101.1 (OCH_2O), 86.5 (C-6'), 84.3 (C-7), 61.4 (C-9), 60.2 (C-9'), 58.5 (C-8), 55.9 (OCH_3-3'), 36.0 (C-7'), 33.6 (C-8'); LRESIMS *m/z* 507 [M+Na]$^+$; HRESIMS *m/z* 507.0269 [M+Na]$^+$ (calcd for $C_{20}H_{21}INaO_6$, 507.0275).

Compound 12: Pale orange powder (11.0 mg, 55%); $[\alpha]_D^{25}$ – 18.2 (*c* 0.1, MeOH); UV (MeOH) λ_{max} (log ε) 211 (0.31), 233 (0.27), 272 (0.21) nm; ECD (MeOH) λ_{ext} 247 nm ($\Delta\varepsilon$ +29.4), 294

nm (Δε –64.3), 315 nm (Δε +2.9); ^{1}H NMR (DMSO-d_6, 800 MHz) δ_H 6.91 (1H, s, H-2'), 6.88 (1H, d, J = 8.0 Hz, H-5), 6.77 (1H, brs, H-2), 6.76 (1H, dd, J = 8.0 Hz, 1.3 Hz, H-6), 6.00 (1H, d, J = 0.8 Hz, OCH_2O), 5.99 (1H, d, J = 0.8 Hz, OCH_2O), 5.77 (1H, d, J = 3.3 Hz, H-7), 4.12 (1H, d, J = 7.7 Hz, H-1''), 3.88 (1H, m, H-6''a), 3.81 (1H, m, H-9'a), 3.80 (3H, s, OCH_3-3'), 3.66 (1H, m, H-9a), 3.56 (1H, m, H-6''b), 3.43 (1H, m, H-9b), 3.41 (1H, m, H-9'b), 3.41 (1H, m, H-8), 3.13 (1H, m, H-3''), 3.08 (1H, m, H-5''), 3.05 (1H, m, H-4''), 2.97 (1H, m, H-2''), 2.69 (1H, m, H-7'a), 2.67 (1H, m, H-7'b), 1.79 (1H, m, H-8'a), 1.76 (1H, m, H-8'b). ^{13}C NMR (DMSO-d_6, 200 MHz) δ_C 147.7 (C-3), 147.1 (C-4), 146.8 (C-4'), 142.9 (C-3'), 135.9 (C-1) 131.9 (C-1'), 125.6 (C-5'), 120.5 (C-6'), 118.6 (C-6), 115.0 (C-2'), 108.5 (C-5), 105.7 (C-2), 103.1 (C-1''), 101.3 (OCH_2O), 86.4 (C-7), 77.0 (C-5''), 76.9 (C-3''), 73.7 (C-2''), 70.3 (C-4''), 67.9 (C-9'), 61.3 (C-9), 61.2 (C-6''), 56.2 (OCH_3-3'), 54.9 (C-8), 29.8 (C-8'), 29.1 (C-7'); LRESIMS m/z 577 $[M + Na]^+$; HRESIMS m/z 577.1437 $[M + Na]^+$ (calcd for $C_{26}H_{31}{}^{35}ClNaO_{11}$, 577.1447).

Compound 13: Brown gum (3.8 mg, 19%); $[\alpha]_D^{25}$ –58.7 (c 0.05, MeOH); UV (MeOH) λ_{max} (log ε) 206 (20.0), 236 (5.7), 294 (2.4) nm; ECD (MeOH) λ_{ext} 251 nm (Δε +35.6), 298 nm (Δε –72.8), 316 nm (Δε +9.7); ^{1}H NMR (DMSO-d_6, 800 MHz) δ_H 7.27 (1H, s, H-5), 6.95 (1H, s, H-2'), 6.55 (1H, s, H-2), 6.06 (1H, d, J = 1.0 Hz, OCH_2O), 6.01 (1H, d, J = 1.0 Hz, OCH_2O), 5.91 (1H, d, J = 2.9 Hz, H-7), 4.14 (1H, d, J = 8.0 Hz, H-1''), 3.95 (1H, dd, J = 10.8 Hz, 3.3 Hz, H-9a), 3.81 (2H, m, H-9'a, H-6''a), 3.81 (3H, s, OCH_3-3'), 3.66 (dd, J = 10.8 Hz, 2.7 Hz, H-9b), 3.45 (1H, m, H-9'b), 3.45 (1H, m, H-6''b), 3.31 (1H, m, H-8), 3.14 (1H, t, J = 3.14 Hz, H-3''), 3.09 (1H, m, H-5''), 3.06 (1H, m, H-4''), 2.98 (1H, m, H-2''), 2.71 (1H, m, H-7'a), 2.69 (1H, m, H-7'b), 1.81 (1H, m, H-8'a), 1.78 (1H, m, H-8'b). ^{13}C NMR (DMSO-d_6, 200 MHz) δ_C 147.6 (C-3), 147.0 (C-4), 146.0 (C-4'), 142.6 (C-3'), 133.1 (C-1), 133.0 (C-1'), 127.2 (C-5'), 114.3 (C-2'), 112.4 (C-5), 110.9 (C-6'), 109.8 (C-6), 105.6 (C-2), 102.4 (C-1''), 101.8 (OCH_2O), 84.4 (C-7), 76.4 (C-5''), 76.3 (C-3''), 73.0 (C-2''), 69.6 (C-4''), 67.1 (C-9'), 60.6 (2C, C-9, C-6''),

55.5 (OCH_3-3'), 54.4 (C-8), 30.8 (C-7'), 29.3 (C-8'); LRESIMS *m/z* 697 [M(^{79}Br)+Na]$^+$, 699 [M(^{81}Br)+Na]$^+$; HRESIMS *m/z* 699.0033 [M(^{79}Br)+Na]$^+$ (calcd for $C_{26}H_{30}{}^{79}Br_2NaO_{11}$, 699.0047).

Compound 14: Brown gum (2.3 mg, 11%); $[\alpha]_D^{25}$ –58.8 (*c* 0.05, MeOH); UV (MeOH) λ_{max} (log ε) 205 (42.9), 236 (13.3), 295 (3.8) nm; ECD (MeOH) λ_{ext} 238 nm ($\Delta\varepsilon$ +31.4), 231 nm ($\Delta\varepsilon$ +1.8), 301 nm ($\Delta\varepsilon$ –39.1), 315 nm ($\Delta\varepsilon$ +4.8); ^{1}H NMR (DMSO-d_6, 800 MHz) 7.29 (1H, s, H-5), 6.64 (1H, s, H-2), 6.06 (1H, d, J = 0.9 Hz, OCH_2O), 6.02 (1H, d, J = 0.9 Hz, OCH_2O), 6.02 (1H, d, J = 3.3 Hz, H-7), 4.15 (1H, d, J = 8.0 Hz, H-1''), 3.92 (2H, m, H-9a, H-9b), 3.85 (3H, s, OCH_3-3'), 3.84 (1H, m, H-9'a), 3.66 (1H, dd, J = 12.0, 2.0 Hz, H-6''a), 3.53 (1H, m, H-9'b), 3.45 (1H, m, H-6''b), 3.35 (1H, m, H-8), 3.13 (1H, t, J = 8.0 Hz, H-3''), 3.08 (1H, m, H-5''), 3.05 (1H, m, H-4''), 2.96 (1H, dd, J = 9.0, 8.0 Hz, H-2''), 2.93 (1H, m, H-7'a), 2.91 (1H, m, H-7'b), 1.74 (1H, m, H-8'a), 1.72 (1H, m, H-8'b). ^{13}C NMR (DMSO-d_6, 200 MHz) δ_C149.7 (C-3), 148.3 (C-4), 147.7 (C-4'), 139.9 (C-3'), 132.9 (2C, C-1, C-1'), 129.1 (C-5'), 118.1 (C-6'), 114.1 (C-2'), 113.0 (C-5), 111.6 (C-6), 106.4 (C-2), 102.8 (C-1''), 102.4 (OCH_2O), 86.0 (C-7), 76.9 (C-5''), 76.8 (C-3''), 73.5 (C-2''), 70.1 (C-4''), 68.1 (C-9'), 61.1 (C-6''), 60.6 (C-9), 60.0 (OCH_3-3'), 54.8 (C-8), 32.9 (C-7'), 28.5 (C-8'); LRESIMS *m/z* 777 [M(^{79}Br)+Na]$^+$, 779 [M(^{79}Br)+Na]$^+$; HRESIMS *m/z* 776.9117 [M(^{79}Br)+Na]$^+$ (calcd for $C_{26}H_{29}{}^{79}Br_3NaO_{11}$, 776.9152).

Compound 15: Yellow needles (5.0 mg, 25%); mp 98–100 °C; $[\alpha]_D^{25}$ –31.3 (*c* 0.08, MeOH); UV (MeOH) λ_{max} (log ε) 210 (0.41), 234 (0.32), 271 (0.24) nm; ECD (MeOH) λ_{ext} 202 nm ($\Delta\varepsilon$ +19.9), 217 nm ($\Delta\varepsilon$ –56.9), 247 nm ($\Delta\varepsilon$ +7.0); ^{1}H NMR (DMSO-d_6, 800 MHz) δ_H 6.91 (1H, s, H-2'), 6.88 (1H, d, J = 7.9 Hz, H-5), 6.71 (1H, d, J = 7.9 Hz, 1.5 Hz, H-6), 6.71 (1H, brs, H-2), 5.97 (1H, d, J = 0.9 Hz, OCH_2O), 5.96 (1H, d, J = 0.9 Hz, OCH_2O), 5.71 (1H, d, J = 2.3 Hz, H-7), 4.13 (1H, d, J = 8.0 Hz, H-1''), 3.92 (1H, dd, J = 10.7, 9.8 Hz, H-9a), 3.88 (1H, m, H-6''a), 3.79 (3H, s, OCH_3-3'), 3.78 (1H, m, H-9'a), 3.56 (1H, dd, J = 11.7, 2.0 Hz, H-6''b), 3.44 (1H, m, H-9b), 3.41 (2H, m, H-9'b), 3.41 (1H, m, H-8), 3.14 (1H, t, J = 9.0 Hz, H-3''), 3.09 (1H,

m, H-5''), 3.06 (1H, m, H-4''), 2.97 (1H, t, J = 8.3 Hz, H-2''), 2.70 (1H, m, H-7'a), 2.63 (1H, m, H-7'b), 1.76 (1H, m, H-8'a), 1.73 (1H, m, H-8'b). ^{13}C NMR (DMSO-d_6, 200 MHz) δ_C 147.7 (C-3), 147.0 (C-4), 145.4 (C-4'), 144.2 (C-3'), 137.0 (C-1'), 135.9 (C-1), 131.4 (C-5'), 118.2 (C-6), 114.2 (C-2'), 108.5 (C-5), 105.4 (C-2), 103.0 (C-1''), 101.3 (OCH_2O), 86.5 (C-6'), 85.0 (C-7), 77.0 (C-5''), 76.9 (C-3''), 73.7 (C-2''), 70.2 (C-4''), 67.8 (C-9'), 61.6 (C-9), 61.2 (C-6''), 58.6 (OCH_3-3'), 56.1 (C-8), 35.8 (C-7'), 30.2 (C-8'); LRESIMS m/z 669 [M+Na]$^+$; HRESIMS m/z 669.0795 [M+Na]$^+$ (calcd. for $C_{26}H_{31}INaO_{11}$, 669.0803).

Compound 17: Pale yellow gum (0.5 mg, 2.5%); $[\alpha]_D^{24}$ –42.9 (c 0.14, MeOH); UV (MeOH) λ_{max} (log ε) 211 (0.62), 233 (0.51), 270 (0.47) nm; ECD (MeOH) λ_{ext} 247 nm ($\Delta\varepsilon$ +26.4), 294 nm ($\Delta\varepsilon$ –60.7), 315 nm ($\Delta\varepsilon$ +1.7); ^{1}H NMR (DMSO-d_6, 800 MHz) δ_H 6.89 (1H, s, H-2'), 6.88 (1H, d, J = 6.4 Hz, H-5), 6.74 (1H, brs, H-2), 6.74 (1H, dd, J = 6.4, 1.6 Hz, H-6), 6.00 (1H, d, J = 0.9 Hz, OCH_2O), 5.99 (1H, d, J = 0.9 Hz, OCH_2O), 5.64 (1H, d, J = 3.4 Hz, H-7), 3.80 (3H, s, OCH_3-3'), 3.75 (1H, dd, J = 9.7, 3.5 Hz,, H-9'a), 3.61 (1H, m, H-9'b), 3.46 (1H, m, H-8), 3.34 (2H, t, J = 6.3 Hz, H-9a, H-9b), 3.32 (3H, s, OCH_3-9), 3.25 (3H, s, OCH_3-9'), 2.65 (1H, m, H-7'a), 2.63 (1H, m, H-7'b), 1.76 (1H, m, H-8'a), 1.74 (1H, m, H-8'b). ^{13}C NMR (DMSO-d_6, 200 MHz) δ_C 148.0 (C-3), 147.4 (C-4), 146.9 (C-4'), 143.7 (C-3'), 135.8 (C-1), 134.0 (C-1'), 127.2 (C-5'), 118.8 (C-6), 115.2 (C-2'), 110.6 (C-6'), 108.8 (C-5), 105.9 (C-2), 101.6 (OCH_2O), 86.6 (C-7), 72.2 (C-9'), 71.6 (C-9), 58.9 (OCH_3-9'), 58.3 (OCH_3-9), 56.4 (OCH_3-3'), 53.6 (C-8), 32.1 (C-7'), 30.3 (C-8'); LRESIMS m/z 487 [M(^{79}Br)+Na]$^+$, 489 [M(^{81}Br)+Na]$^+$; HRESIMS m/z 487.0722 [M+Na]$^+$ (calcd for $C_{22}H_{25}{}^{79}BrNaO_6$, 487.0727).

Compound 18: Pale yellow oil (9.6 mg, 48%); $[\alpha]_D^{24}$ –73 (c 0.1, MeOH); UV (MeOH) λ_{max} (log ε) 210 (0.45), 233 (0.5), 281 (0.34); ECD (MeOH) λ_{ext} 209 nm ($\Delta\varepsilon$ +25.7), 221 nm ($\Delta\varepsilon$ –50.4), 243 nm ($\Delta\varepsilon$ +26.1); ^{1}H NMR (DMSO-d_6, 800 MHz) δ_H 7.28 (1H, s, H-5), 6.92 (1H, brs, H-2'), 6.60 (1H, brs, H-2), 6.07 (1H, d, J = 0.9 Hz, OCH_2O), 6.02 (1H, d, J = 0.9 Hz, OCH_2O), 5.86 (1H, d, J = 3.5 Hz, H-7), 3.83 (2H, m, H-9'a, H-9'b), 3.82 (3H, s, OCH_3-3'), 3.47 (1H, m,

H-8), 3.37 (2H, m, H-9a, H-9b), 3.28 (3H, s, OCH_3-9'), 3.25 (3H, s, OCH_3-9), 2.67 (1H, m, H-7'a), 2.66 (1H, m, H-7'b), 1.78 (1H, m, H-8'a), 1.76 (1H, m, H-8'b). ^{13}C NMR (DMSO-d_6, 200 MHz) δ_C 148.2 (C-3), 147.5 (C-4), 146.4 (C-4'), 143.1 (C-3'), 133.9 (C-1), 132.9 (C-1'), 127.0 (C-5'), 114.6 (C-2'), 112.8 (C-5), 112.8 (C-6), 110.0 (C-6'), 106.2 (C-2), 102.3 (OCH_2O), 85.2 (C-7), 71.7 (C-9'), 71.2 (C-9), 58.6 (OCH_3-9), 57.8 (OCH_3-9'), 55.9 (OCH_3-3'), 52.5 (C-8), 31.6 (C-7'), 29.8 (C-8'); LRESIMS *m/z* 565 [M(^{79}Br)+Na]$^+$, 567 [M(^{81}Br)+Na]$^+$; HRESIMS *m/z* 564.9823 [M+Na]$^+$ (calcd for $C_{22}H_{24}{}^{79}Br_2NaO_6$, 564.9832).

Figure S1 ^{1}H NMR (800 MHz) spectrum of suberifolioside B (**1**) in DMSO-d_6

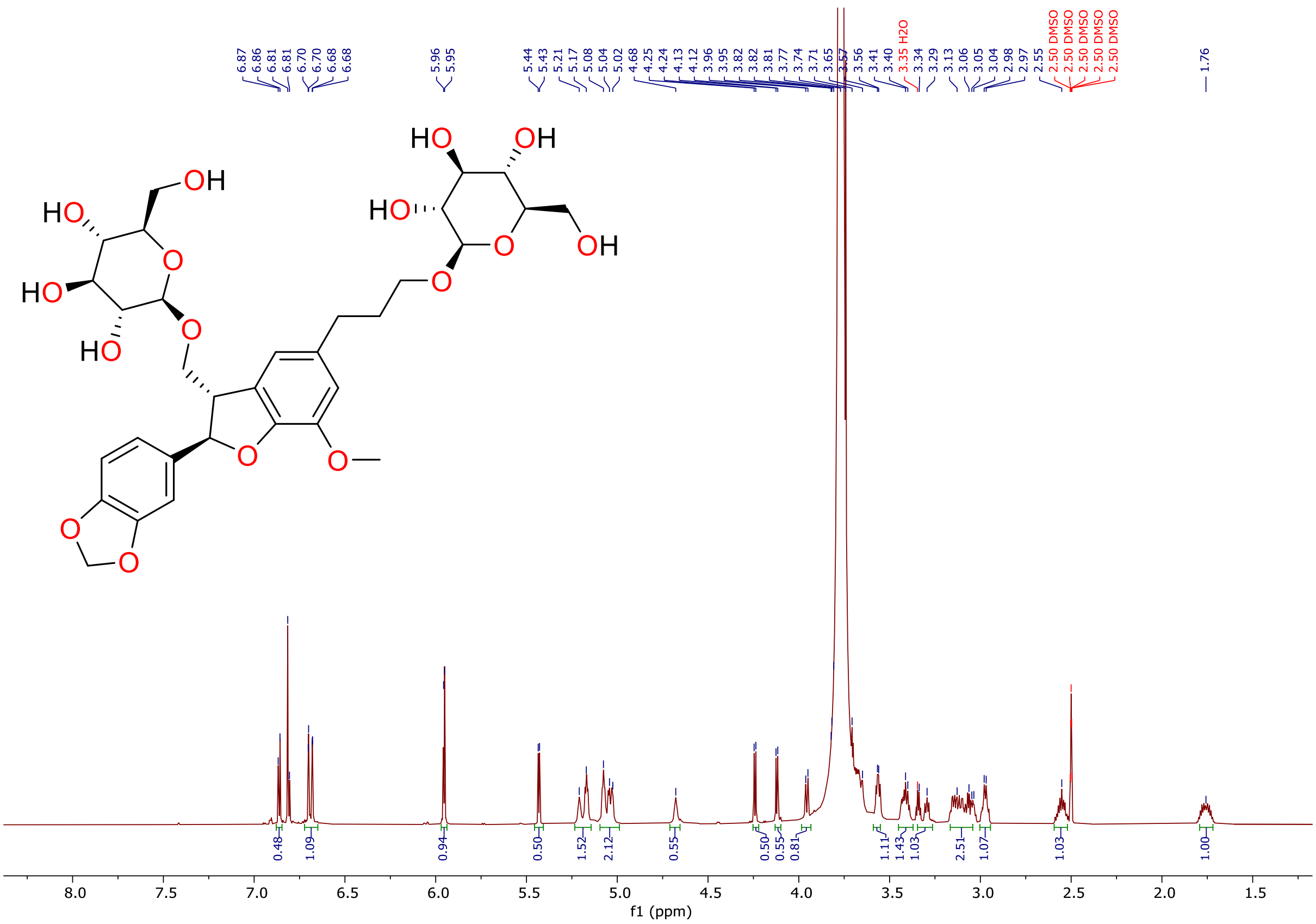

Figure S2 ^{13}C NMR (200 MHz) spectrum of suberifolioside B (**1**) in DMSO-d_6

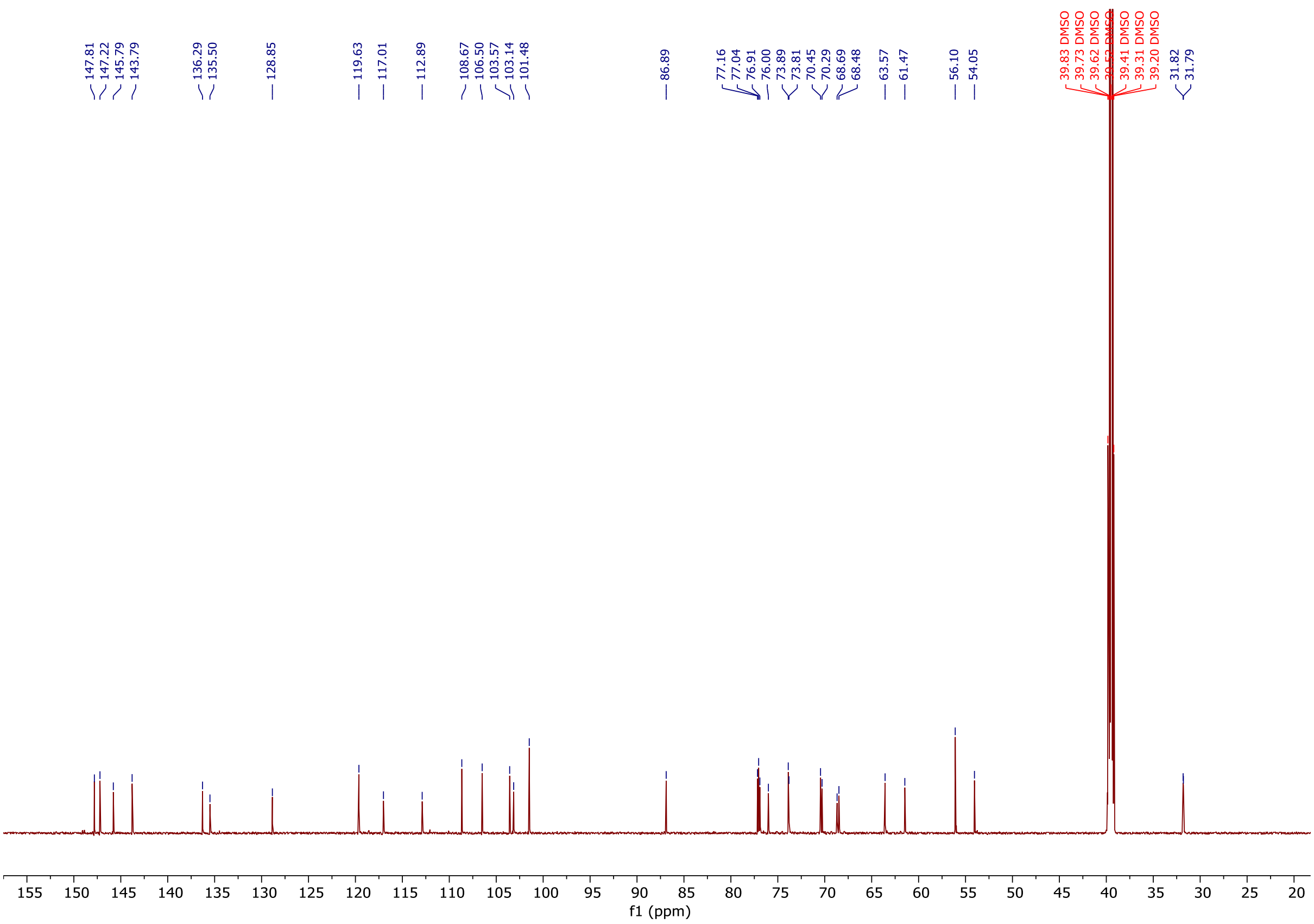

Figure S3 COSY spectrum of suberifolioside B (**1**) in DMSO-d_6

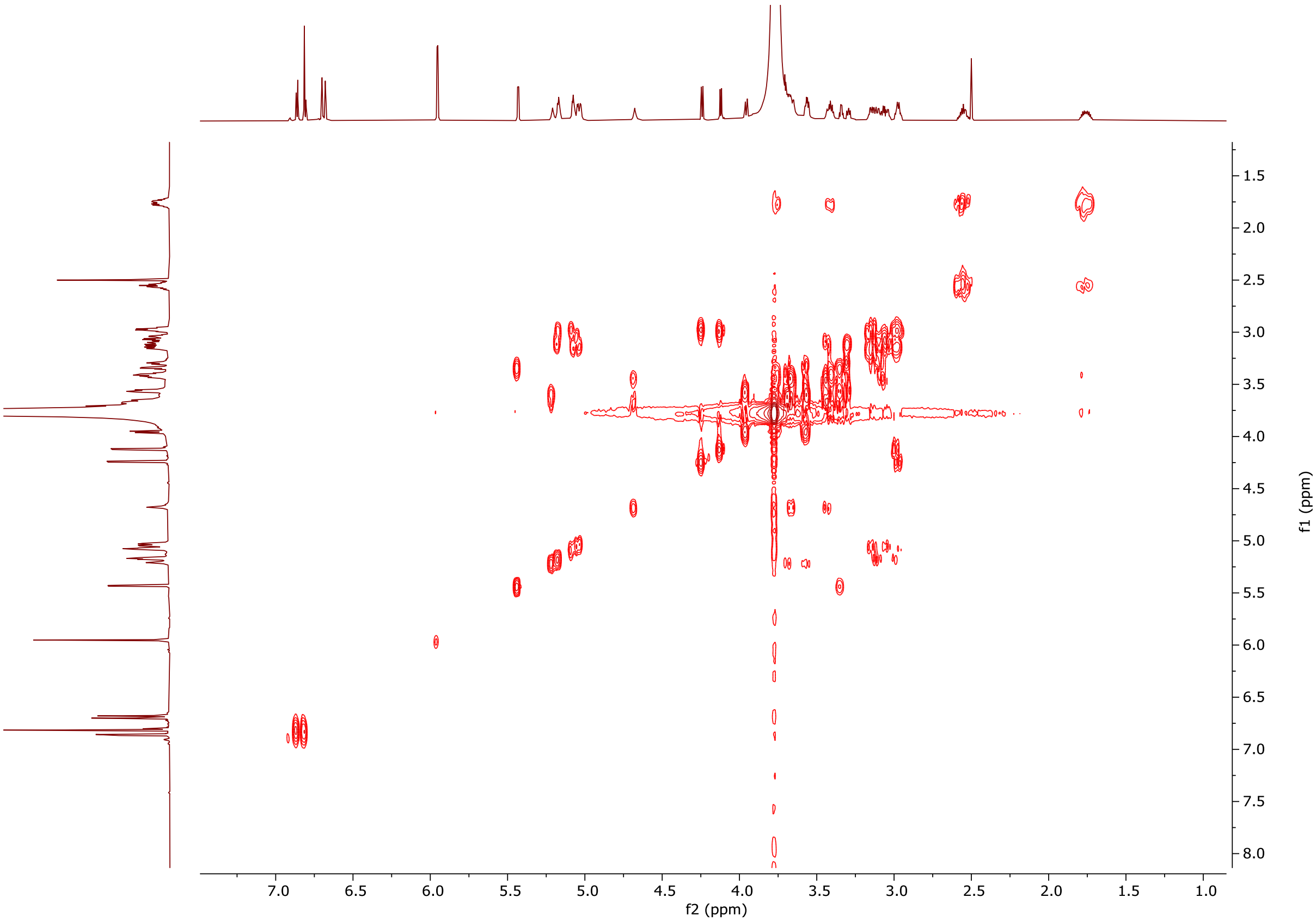

Figure S4 HSQC spectrum of suberifolioside B (**1**) in DMSO-d_6

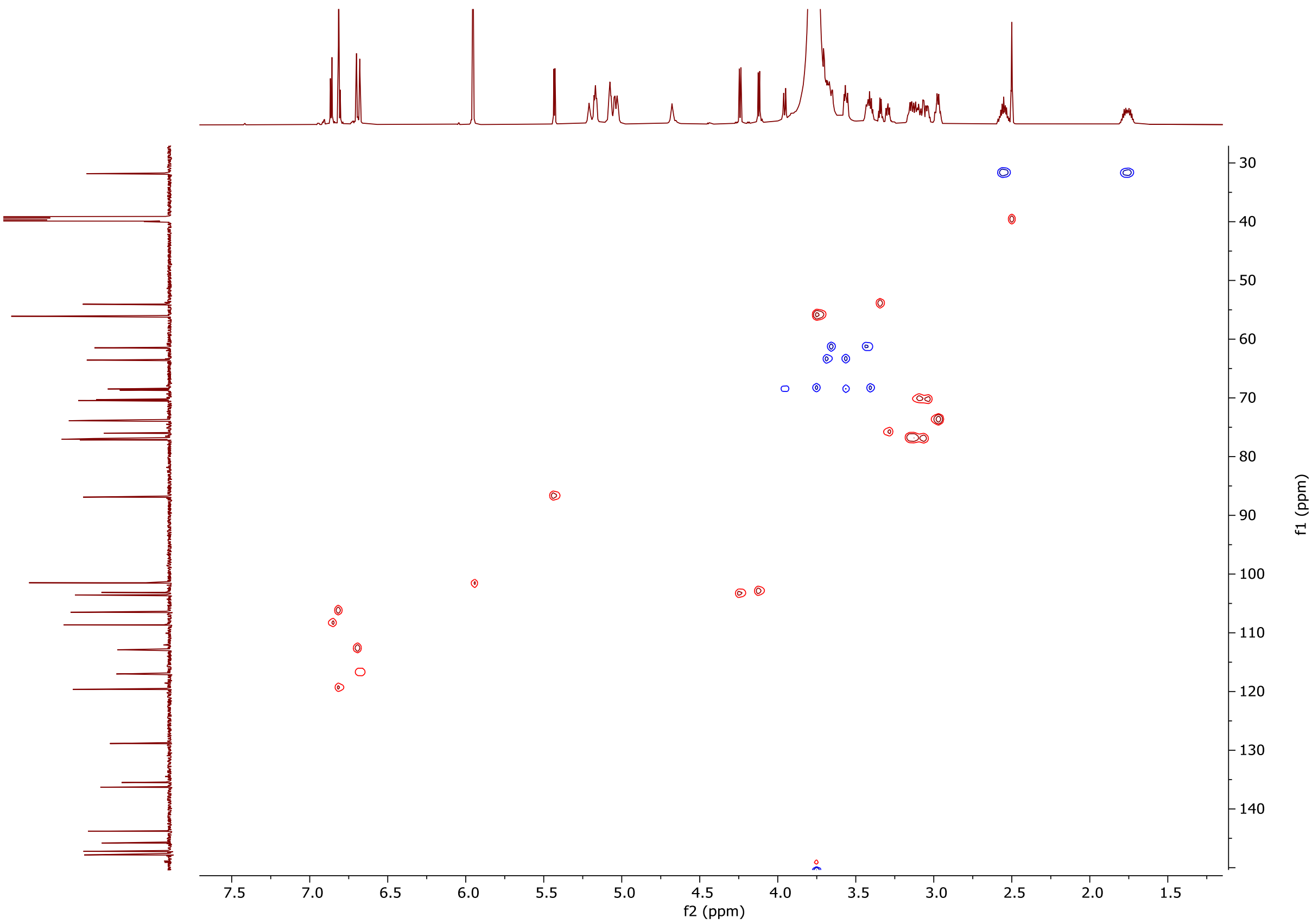

Figure S5 HMBC spectrum of suberifolioside B (**1**) in DMSO-d_6

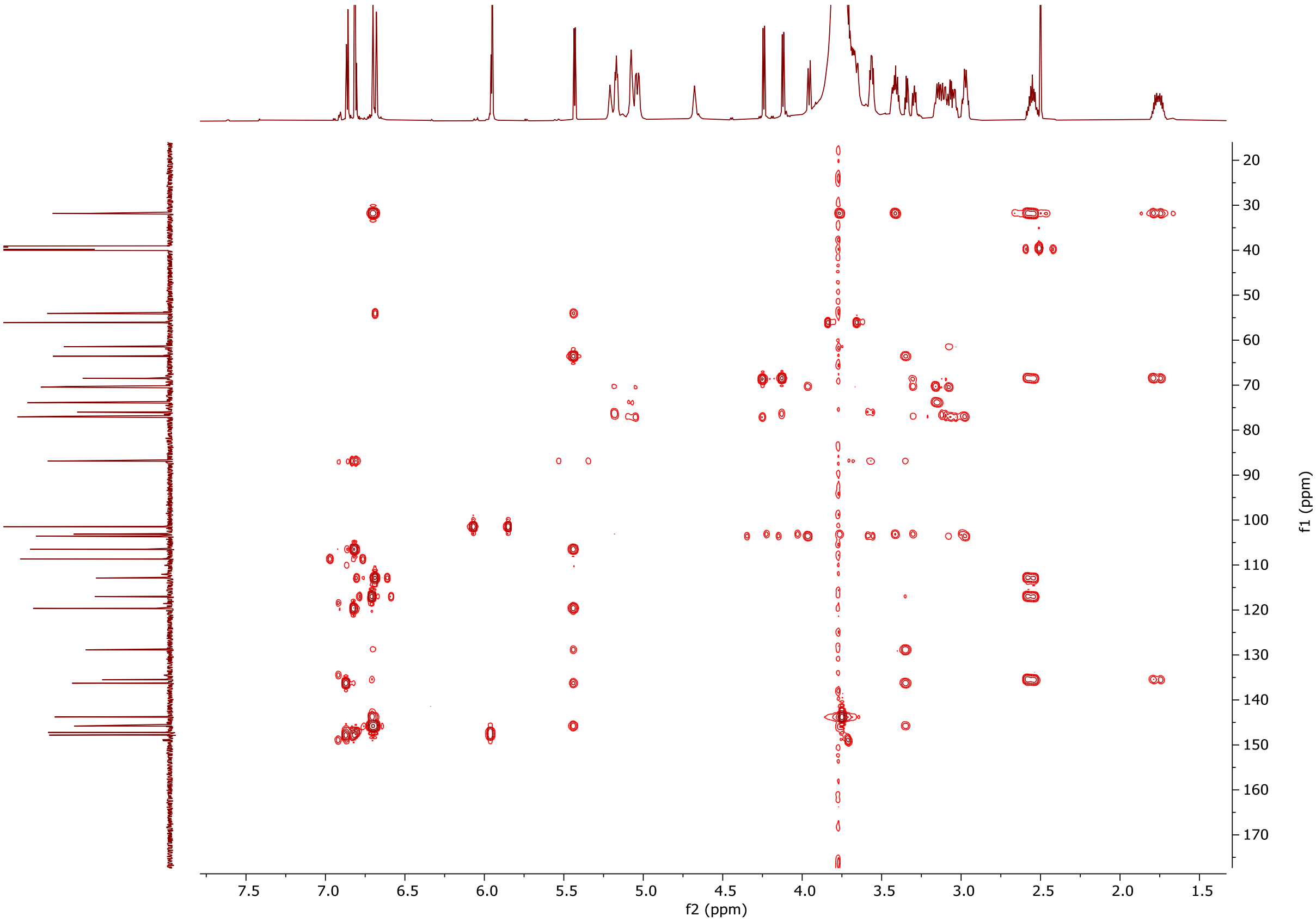

Figure S6 ROESY spectrum of suberifolioside B (**1**) in DMSO-d_6

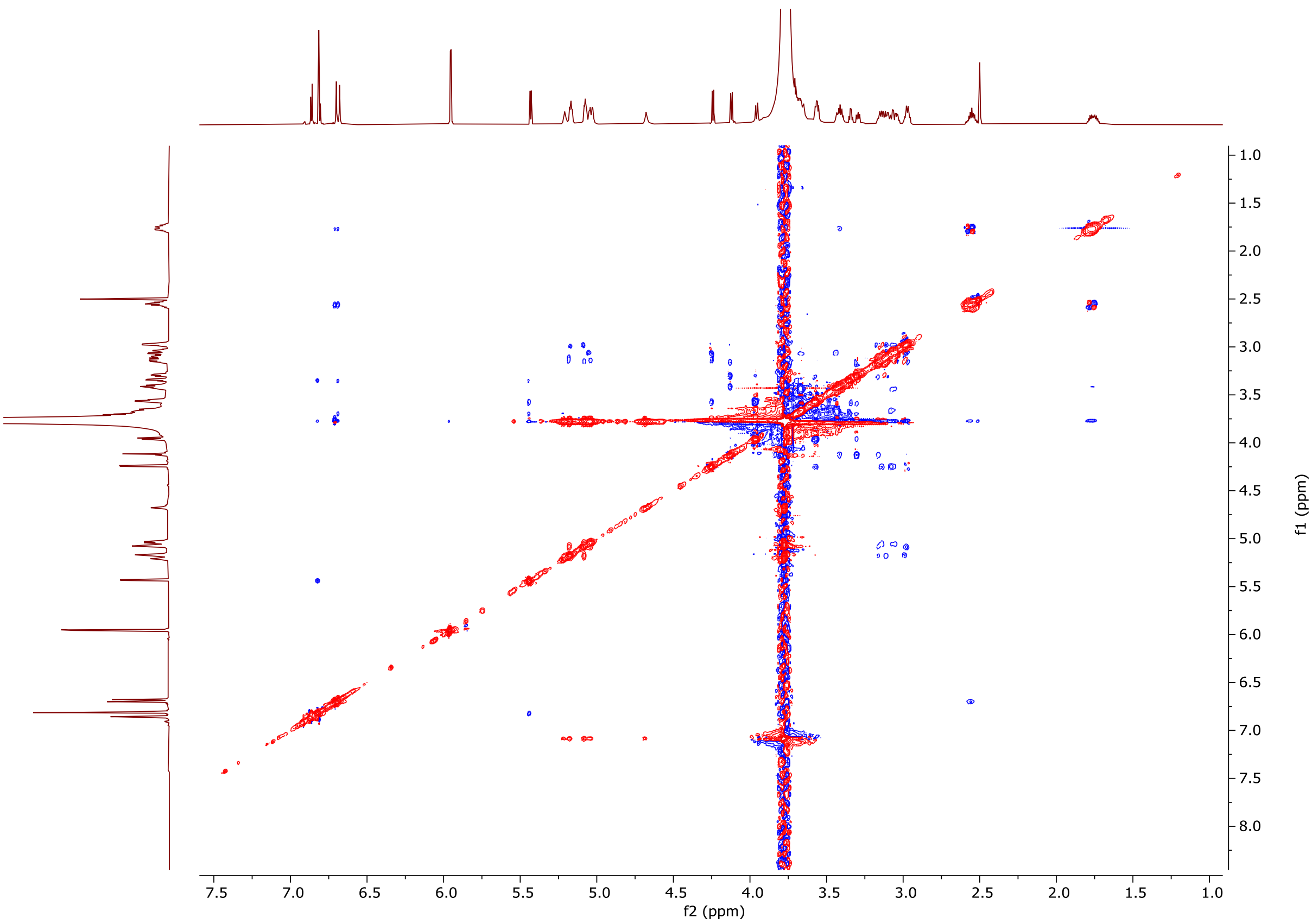

Figure S7 ^{1}H NMR (800 MHz) spectrum of 7*R*,8*S*-dihydrodehydrodiconiferyl alcohol (**2**) in DMSO-d_6

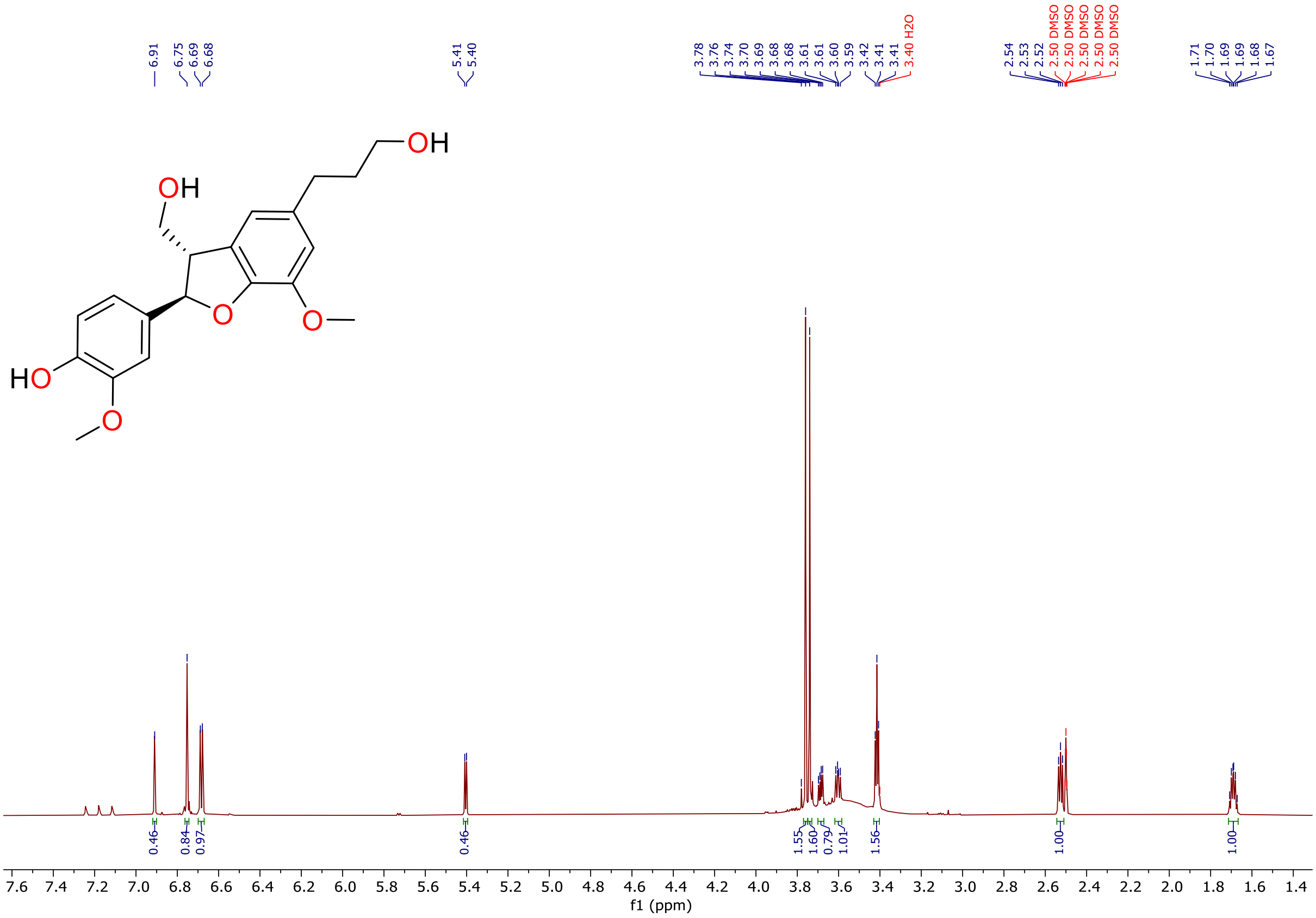

Figure S8 ^{13}C NMR (200 MHz) spectrum of 7*R*,8*S*-dihydrodehydrodiconiferyl alcohol (**2**) in DMSO-d_6

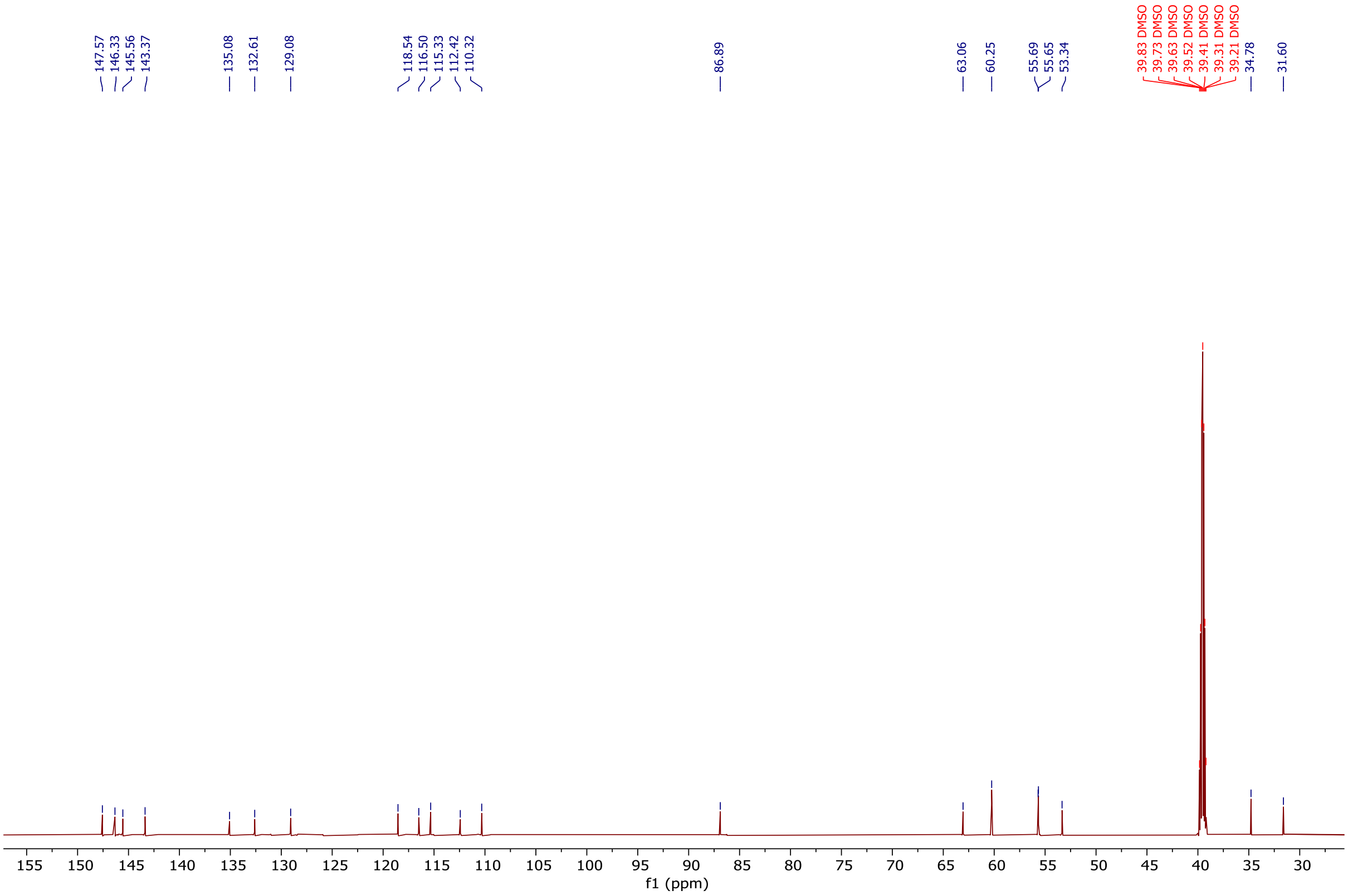

Figure S9 ^{1}H NMR (800 MHz) spectrum of 3',4-*O*-dimethylcedrusin (**3**) in DMSO-d_6

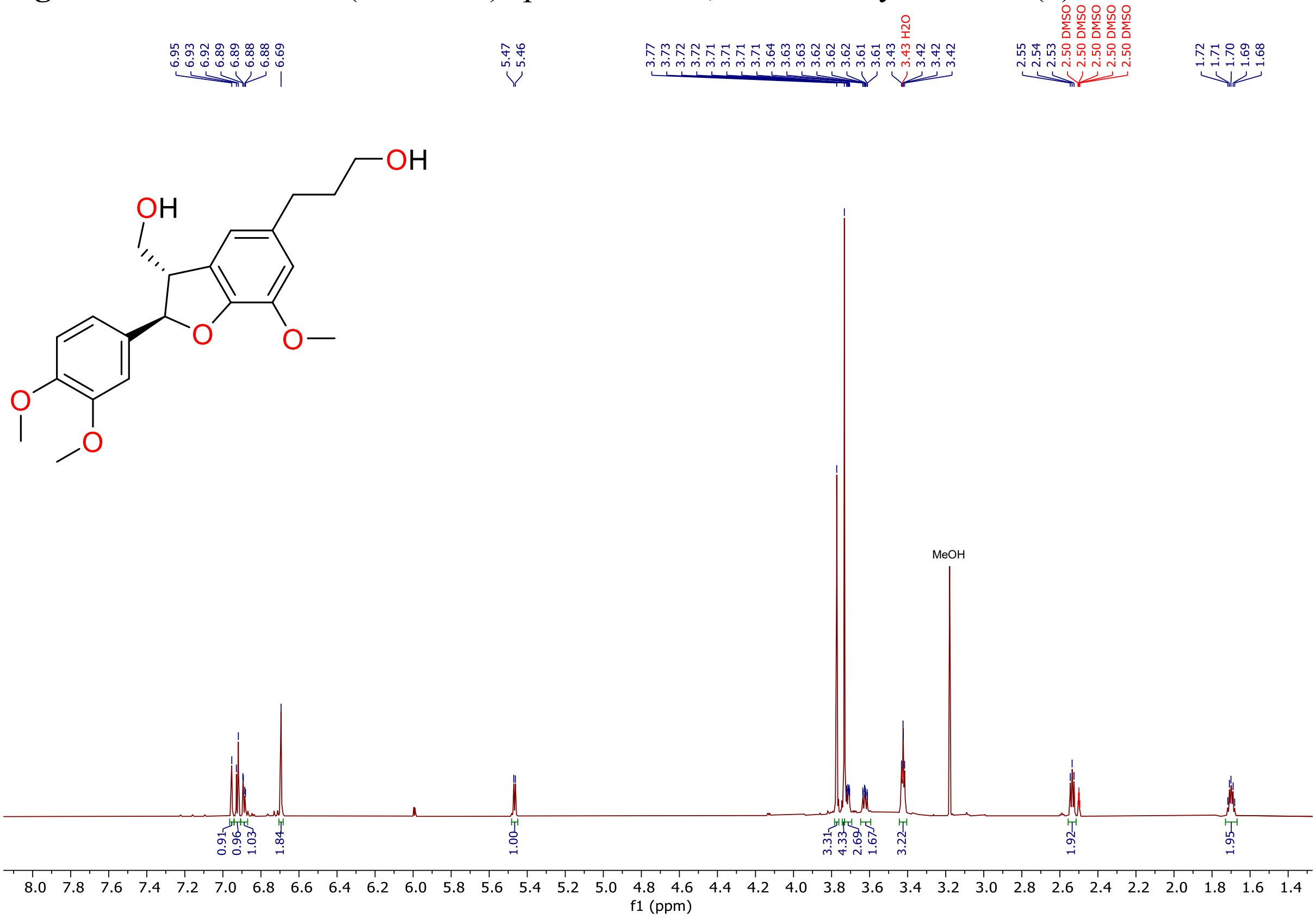

Figure S10 ^{13}C NMR (200 MHz) spectrum of 3',4-*O*-dimethylcedrusin (**3**) in DMSO-d_6

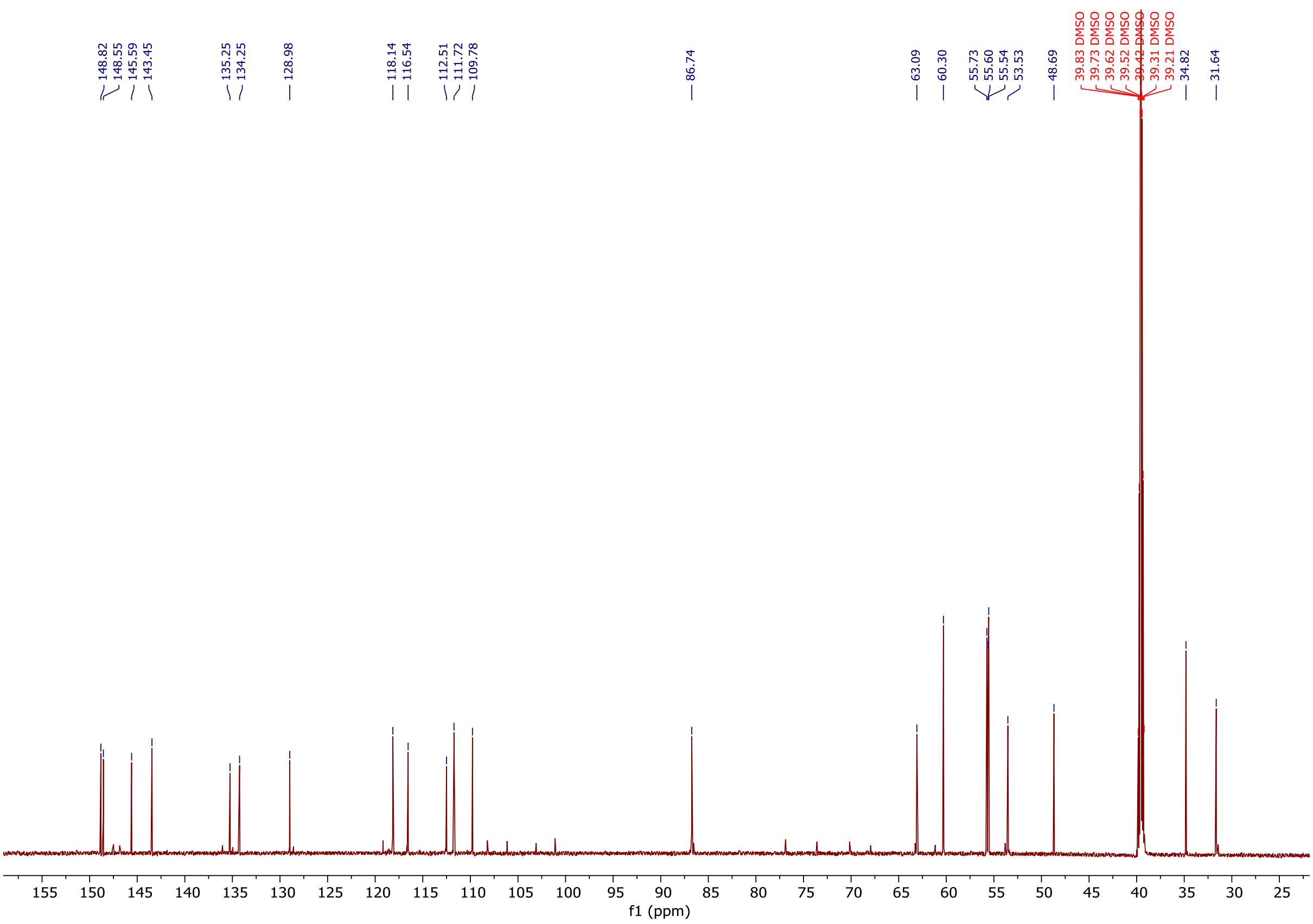

Figure S11 ^{1}H NMR (800 MHz) spectrum of suberifolioside A (**4**) in DMSO-d_6

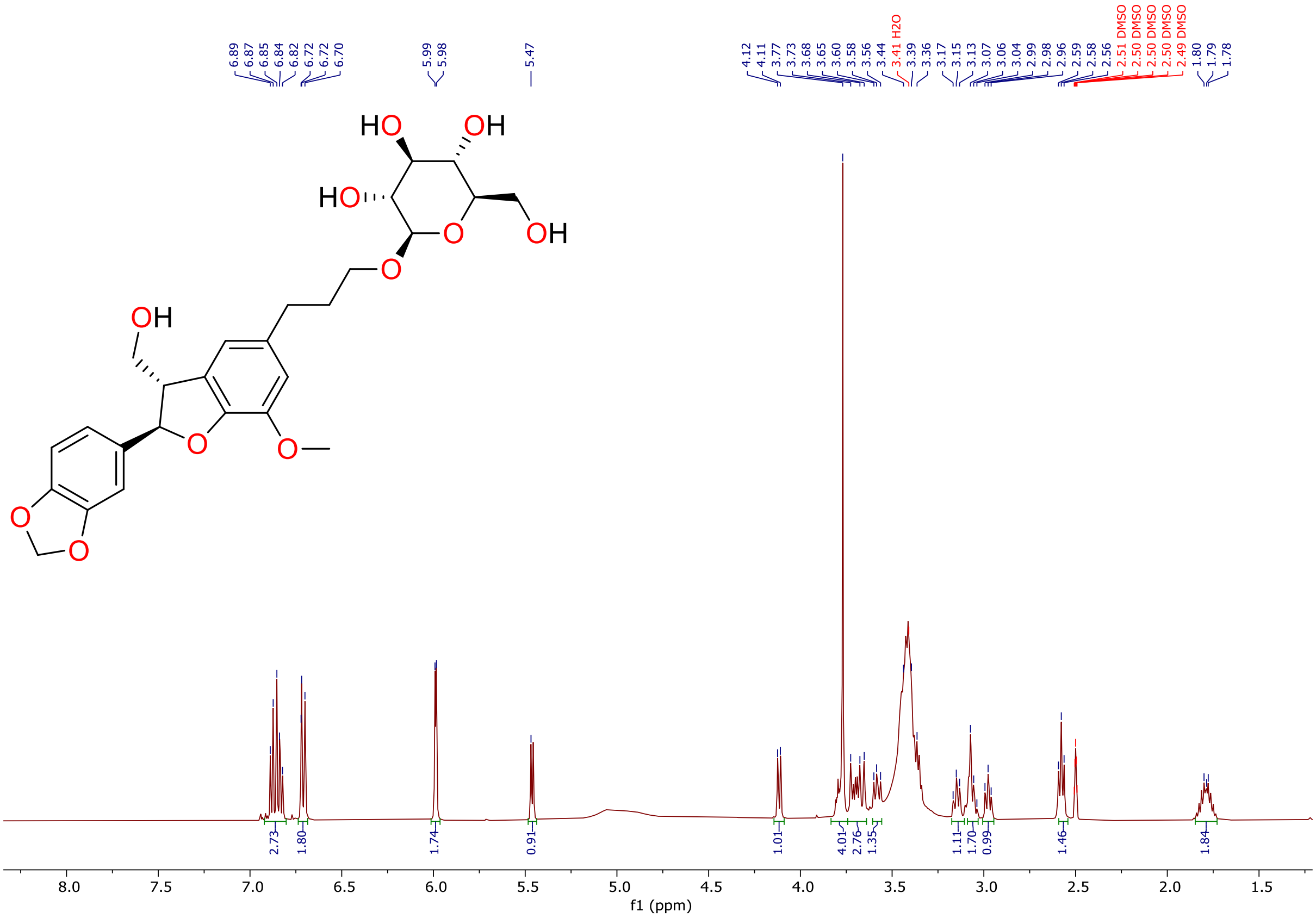

Figure S12 ^{13}C NMR (200 MHz) spectrum of suberifolioside A (**4**) in DMSO-d_6

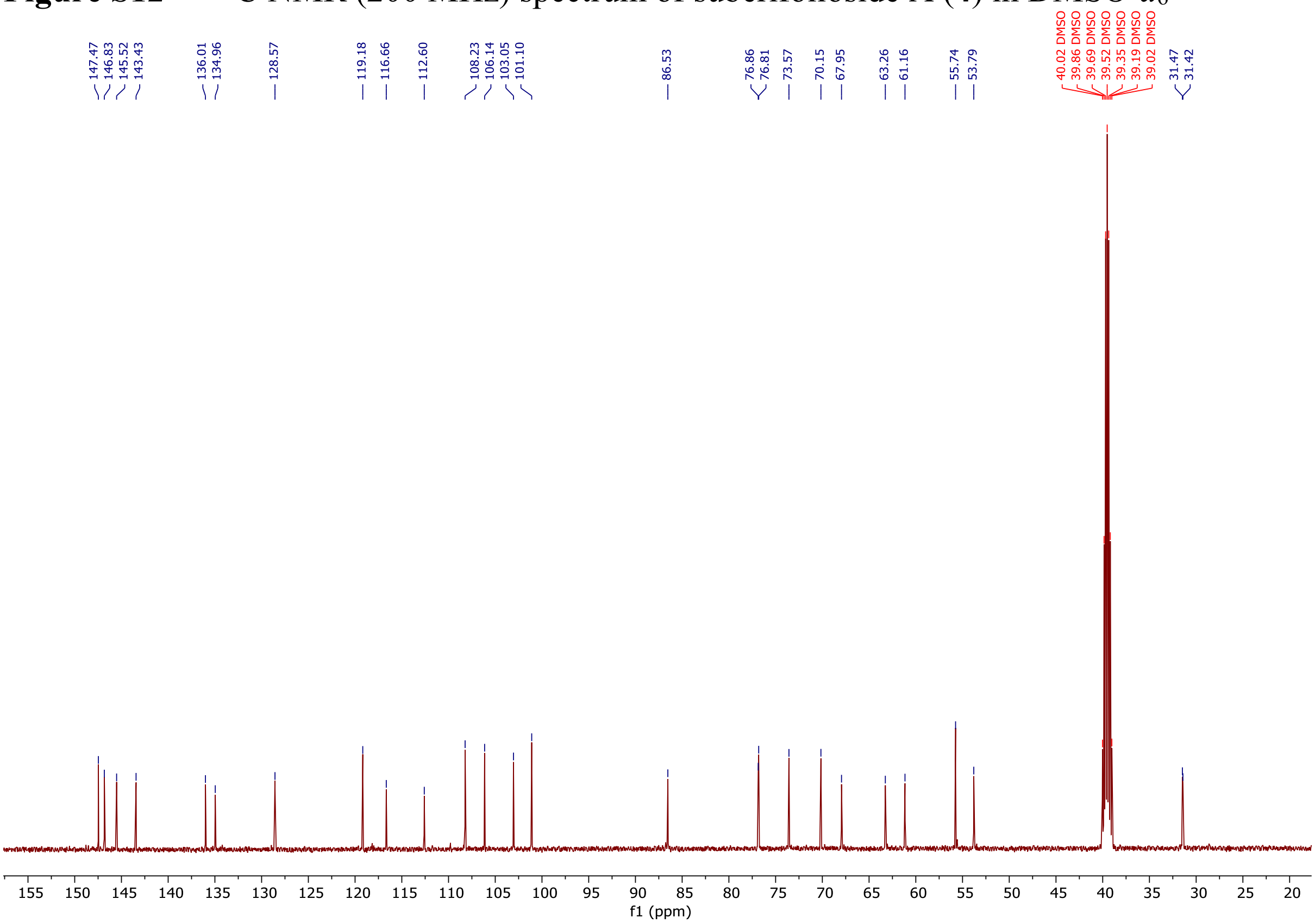

Figure S13 ^{1}H NMR (800 MHz) spectrum of equiselignan B (**5**) in DMSO-d_6

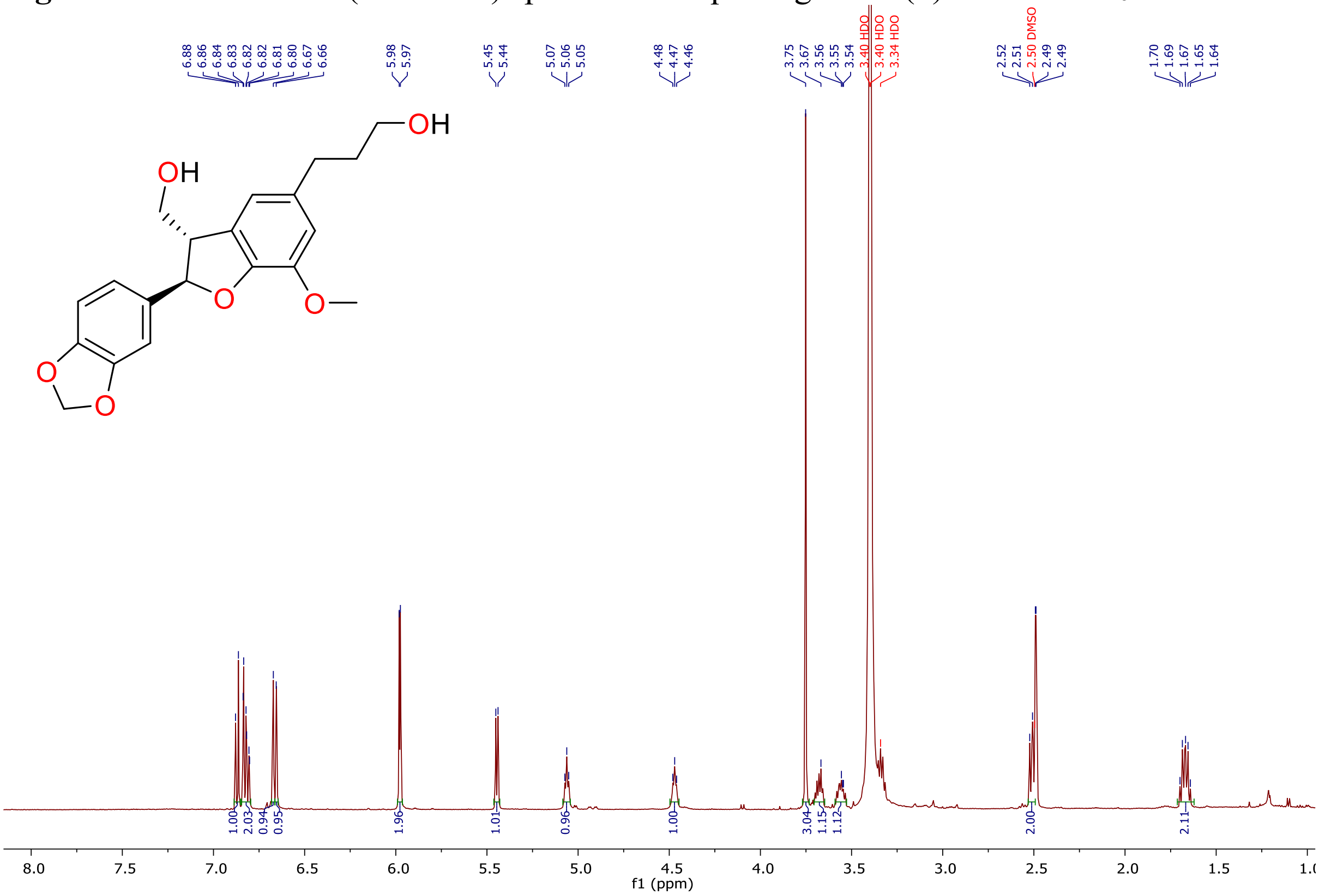

Figure S14 ^{13}C NMR (200 MHz) spectrum of equiselignan B (**5**) in DMSO-d_6

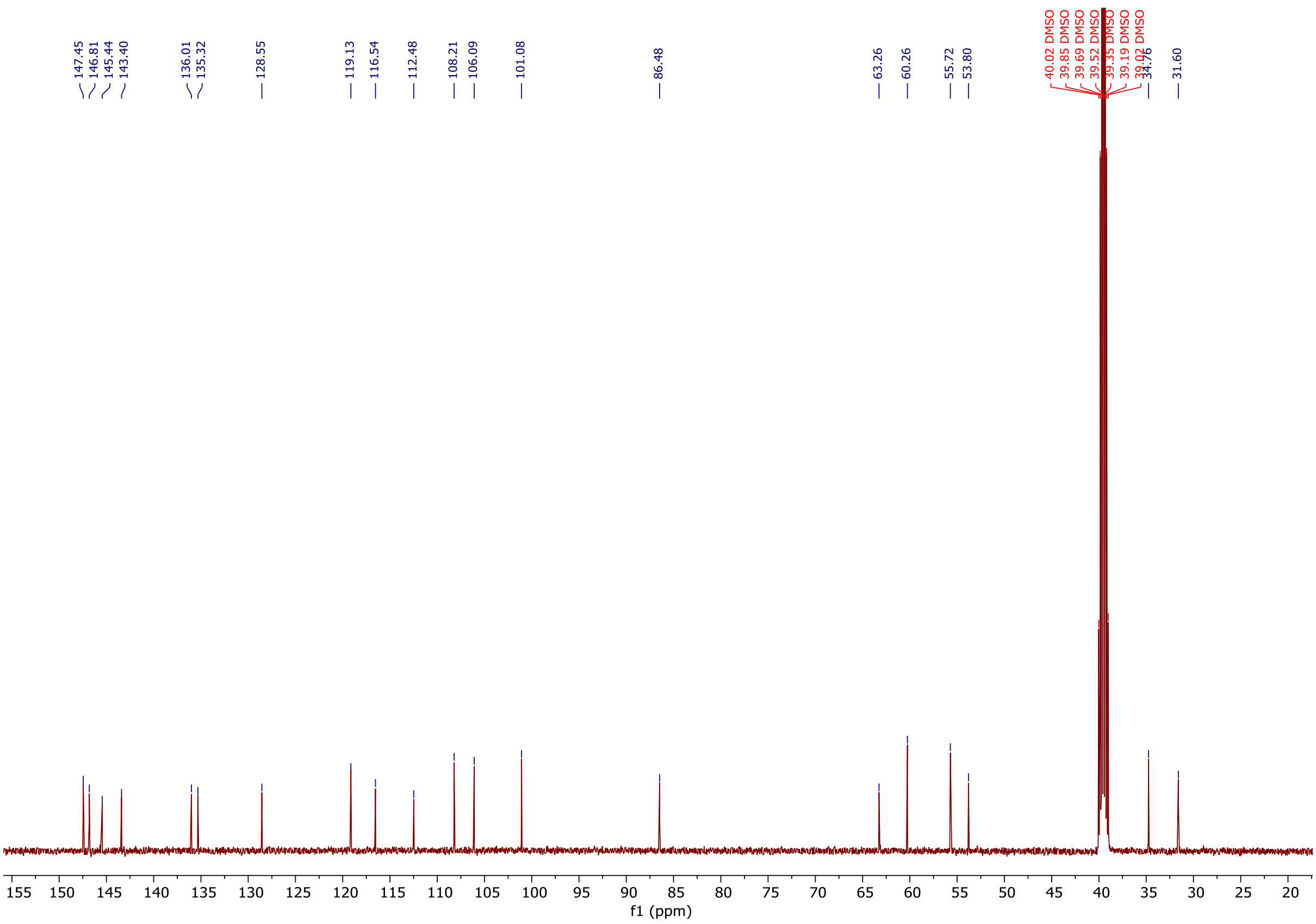

Figure S15 ^{1}H NMR (800 MHz) spectrum of egonol (**6**) in DMSO-d_6

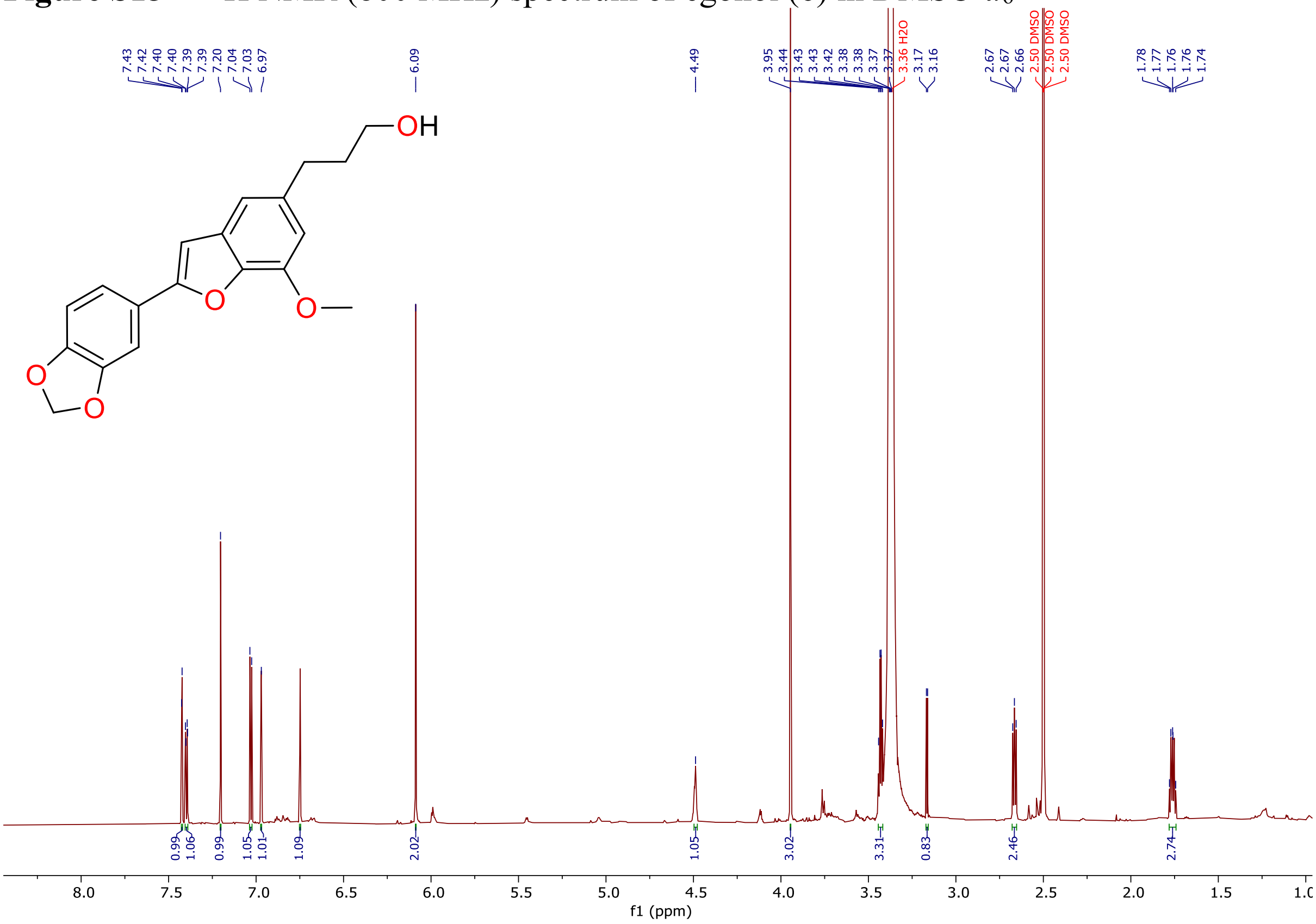

Figure S16 ^{13}C NMR (200 MHz) spectrum of egonol (**6**) in DMSO-d_6

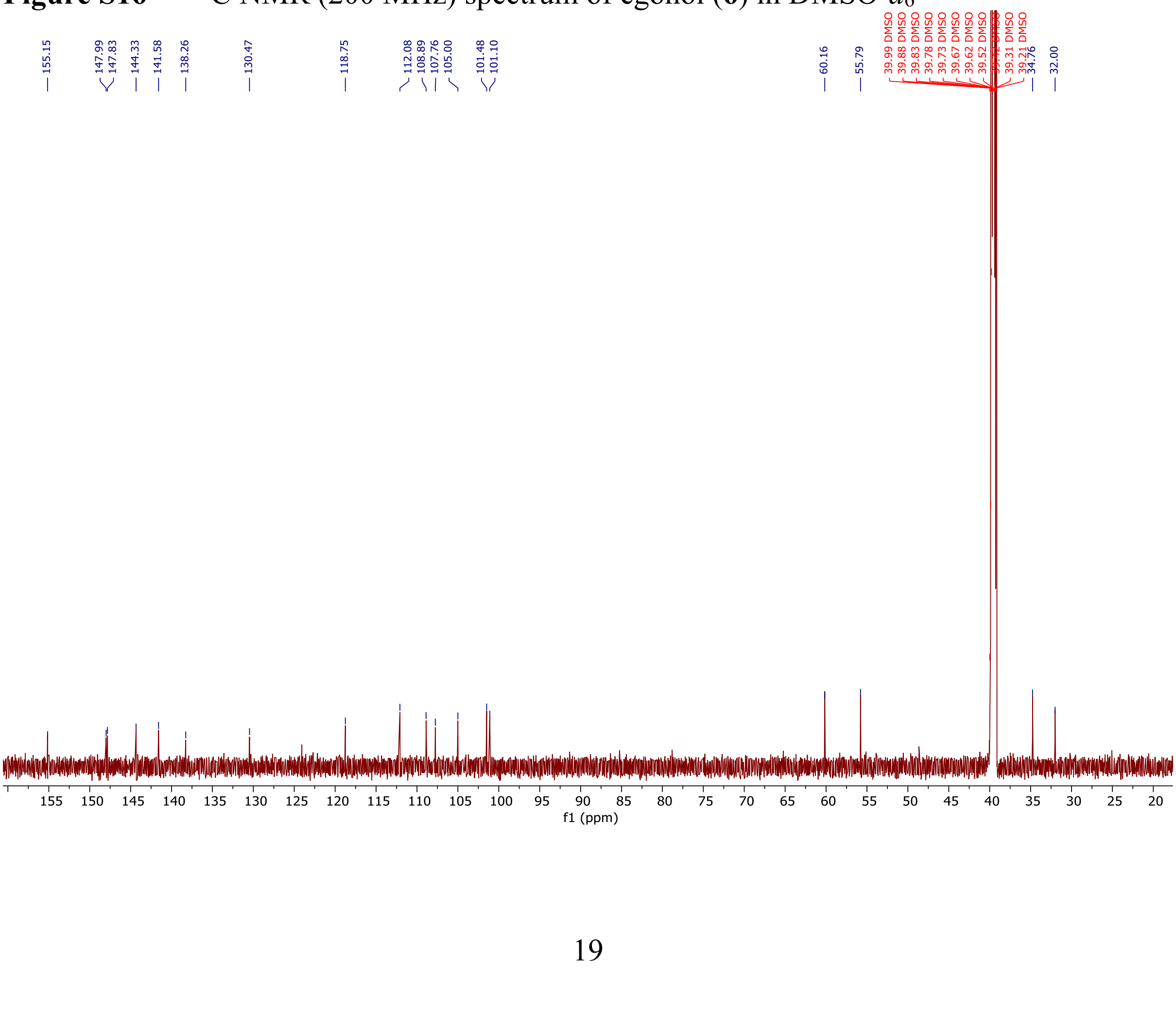

Figure S17 ^{1}H NMR (800 MHz) spectrum of compound **7** in DMSO-d_6

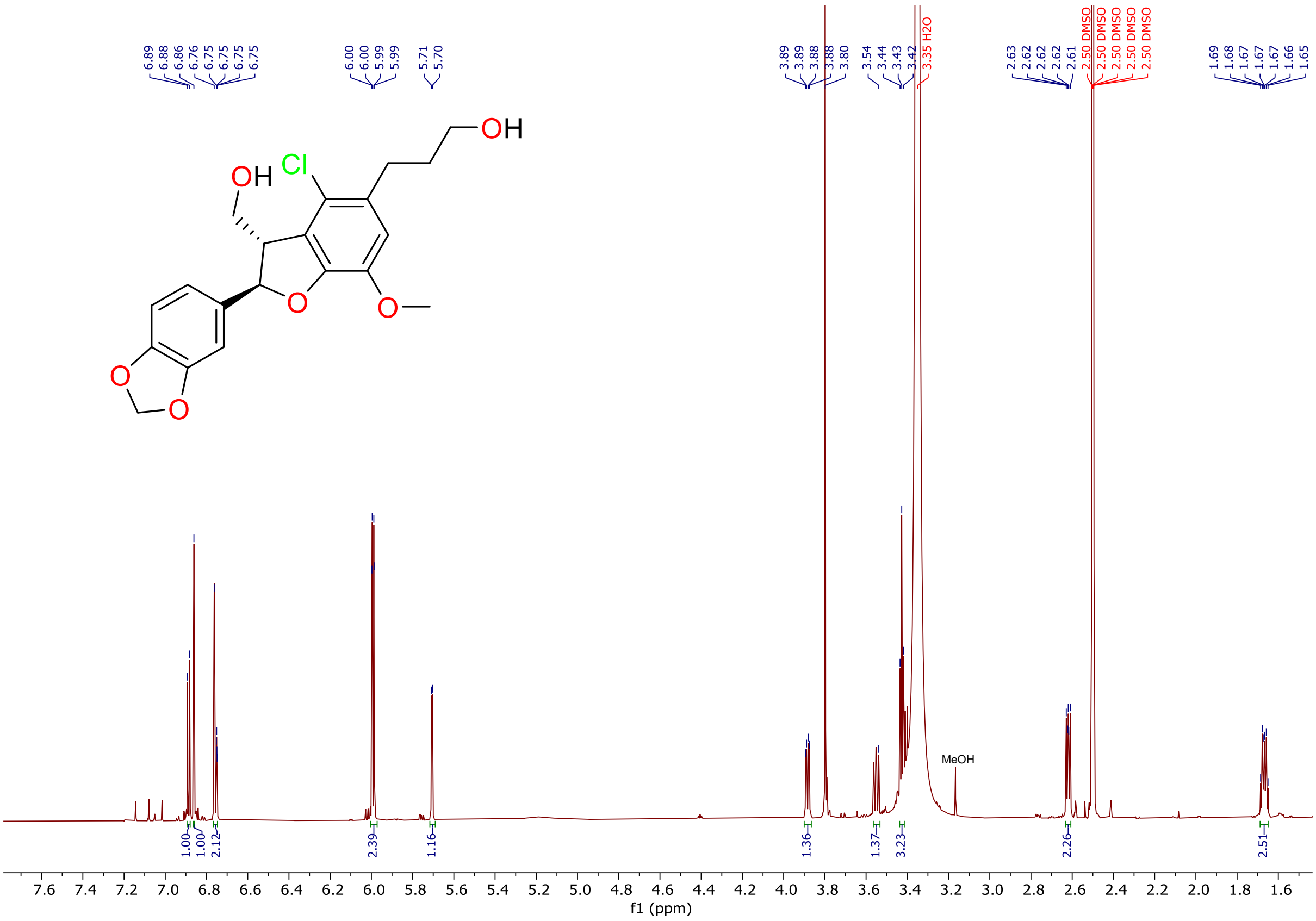

Figure S18 ^{13}C NMR (200 MHz) spectrum of compound **7** in DMSO-d_6

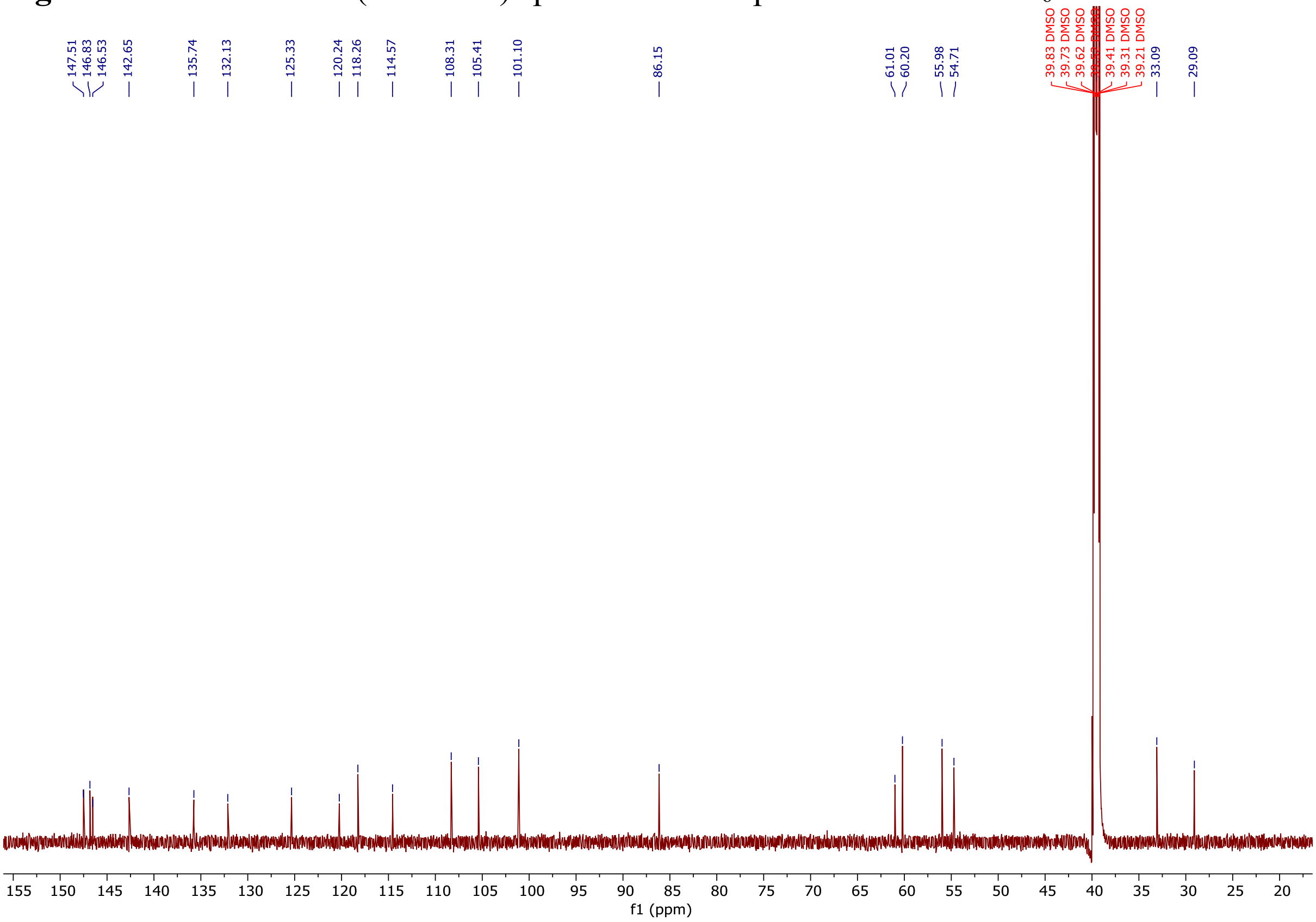

Figure S19 COSY spectrum of compound **7** in DMSO-d_6

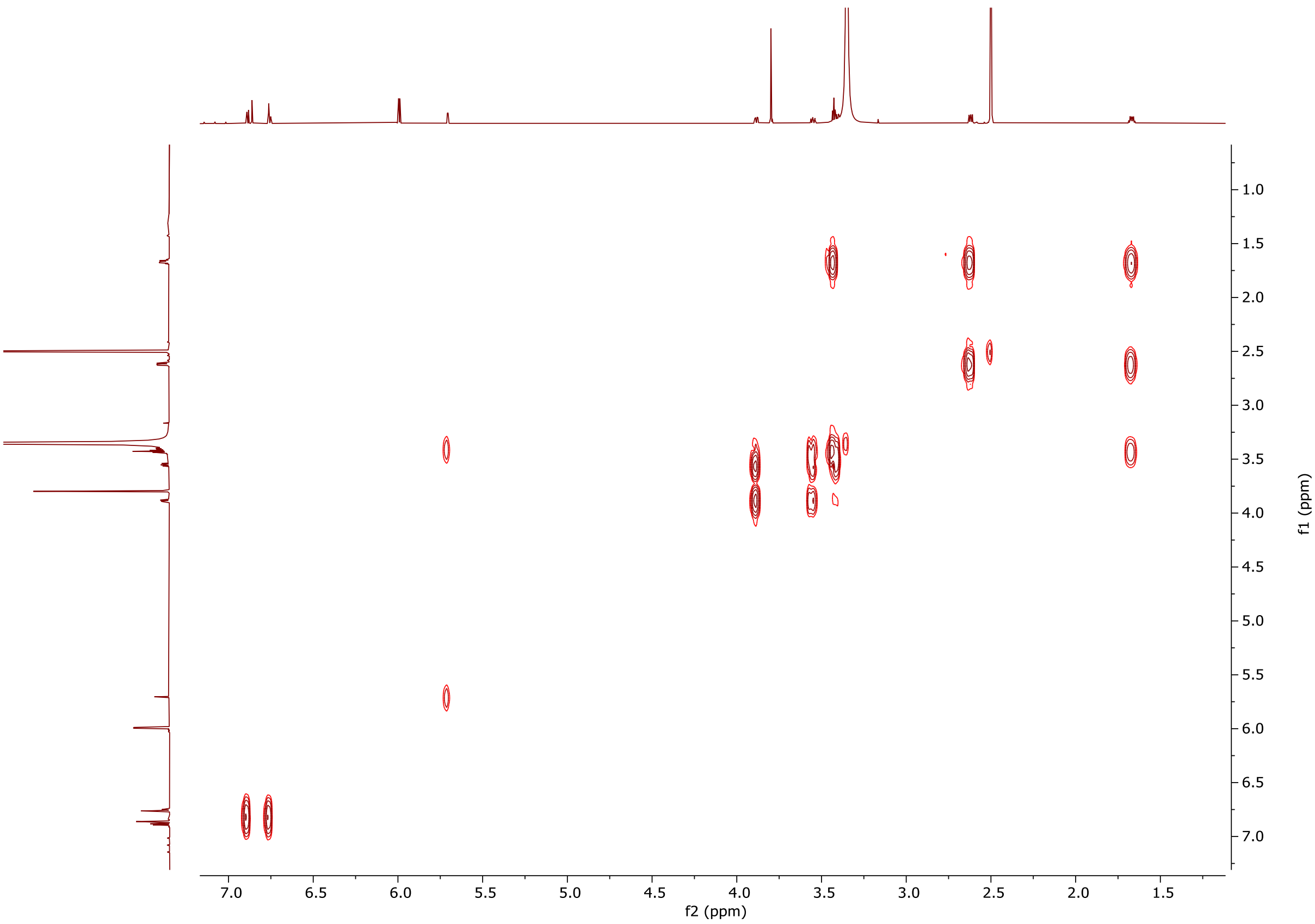

Figure S20 HSQC spectrum of compound **7** in DMSO-d_6

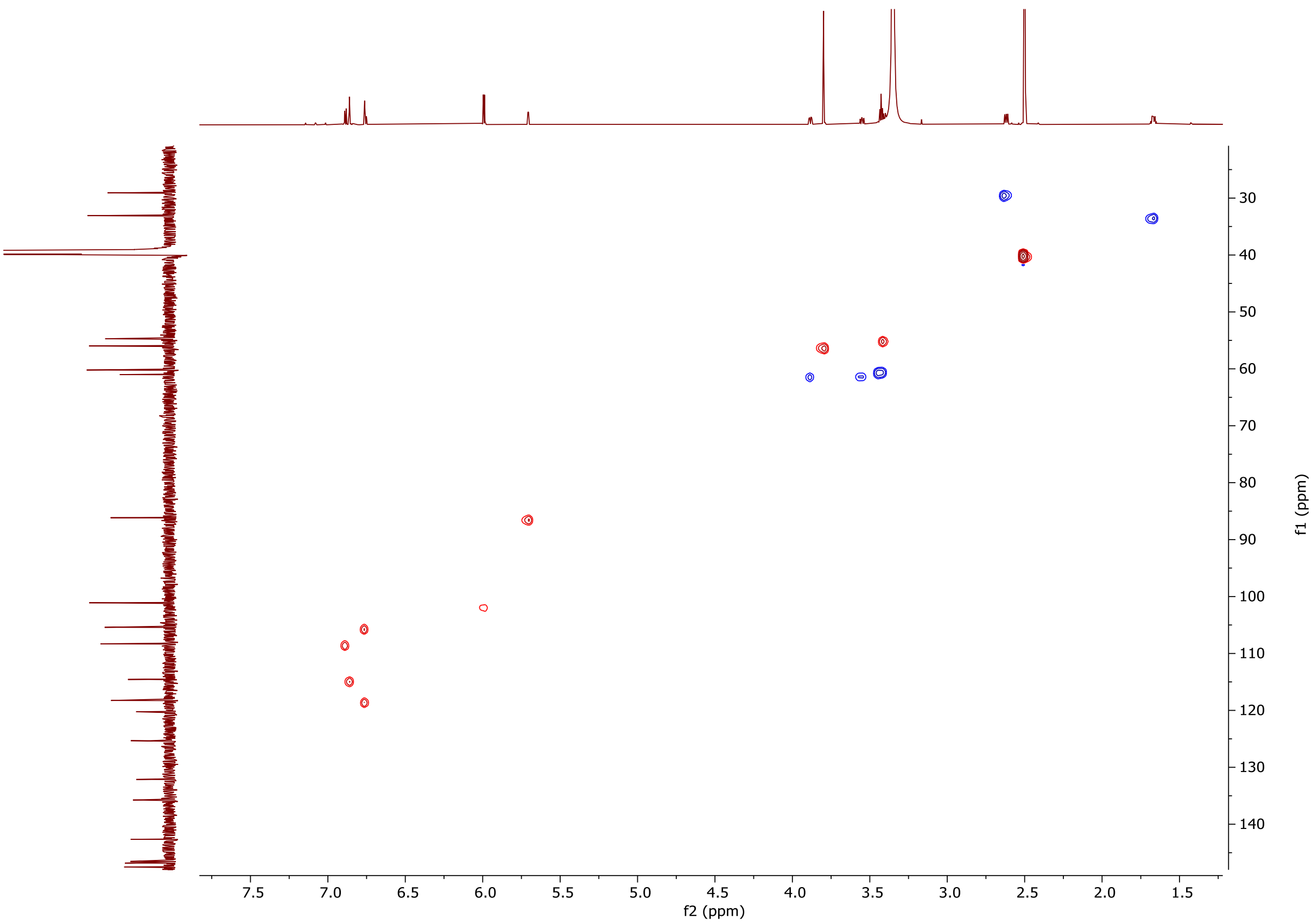

Figure S21 HMBC spectrum of compound **7** in DMSO-d_6

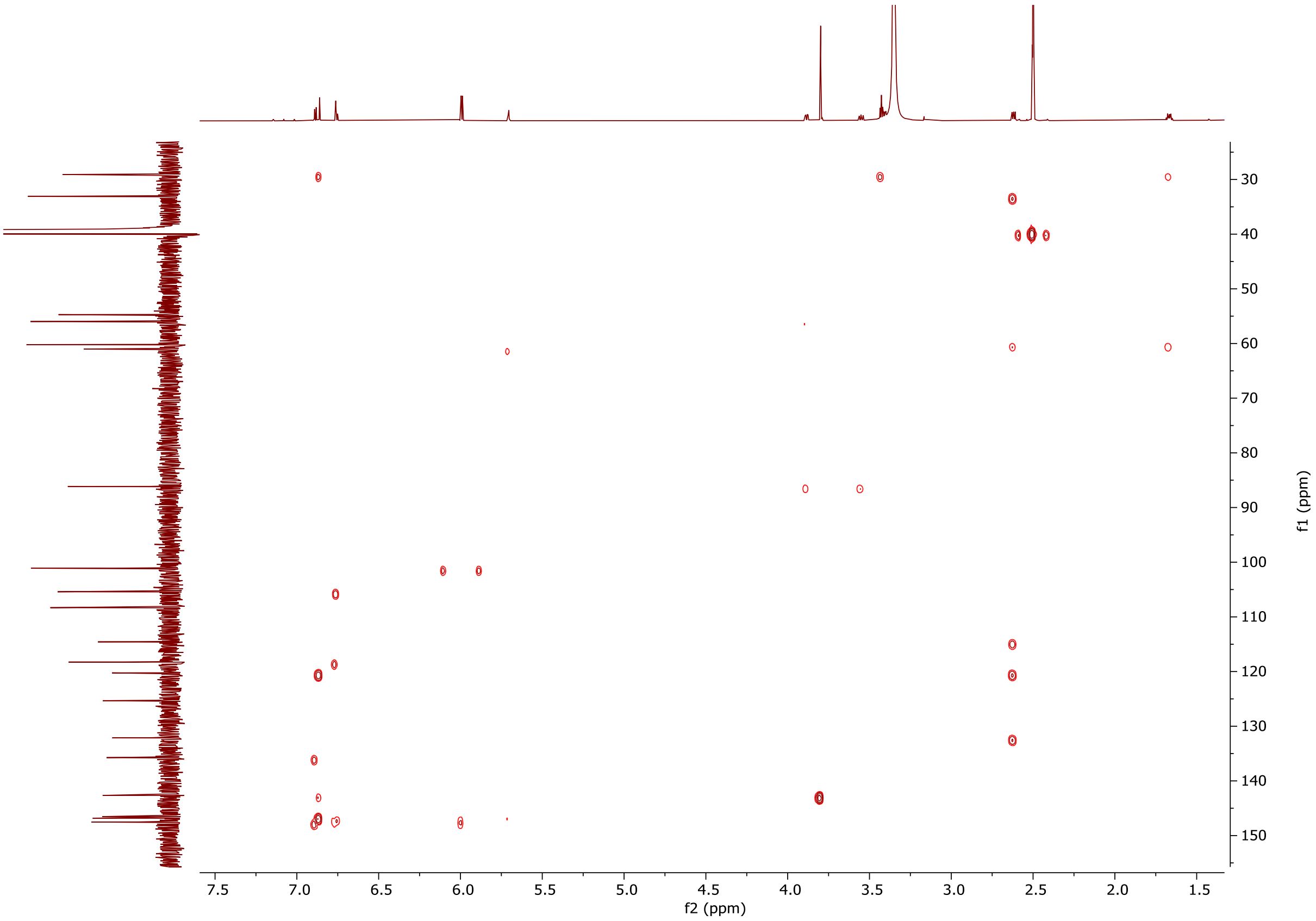

Figure S22 ROESY spectrum of compound **7** in DMSO-d_6

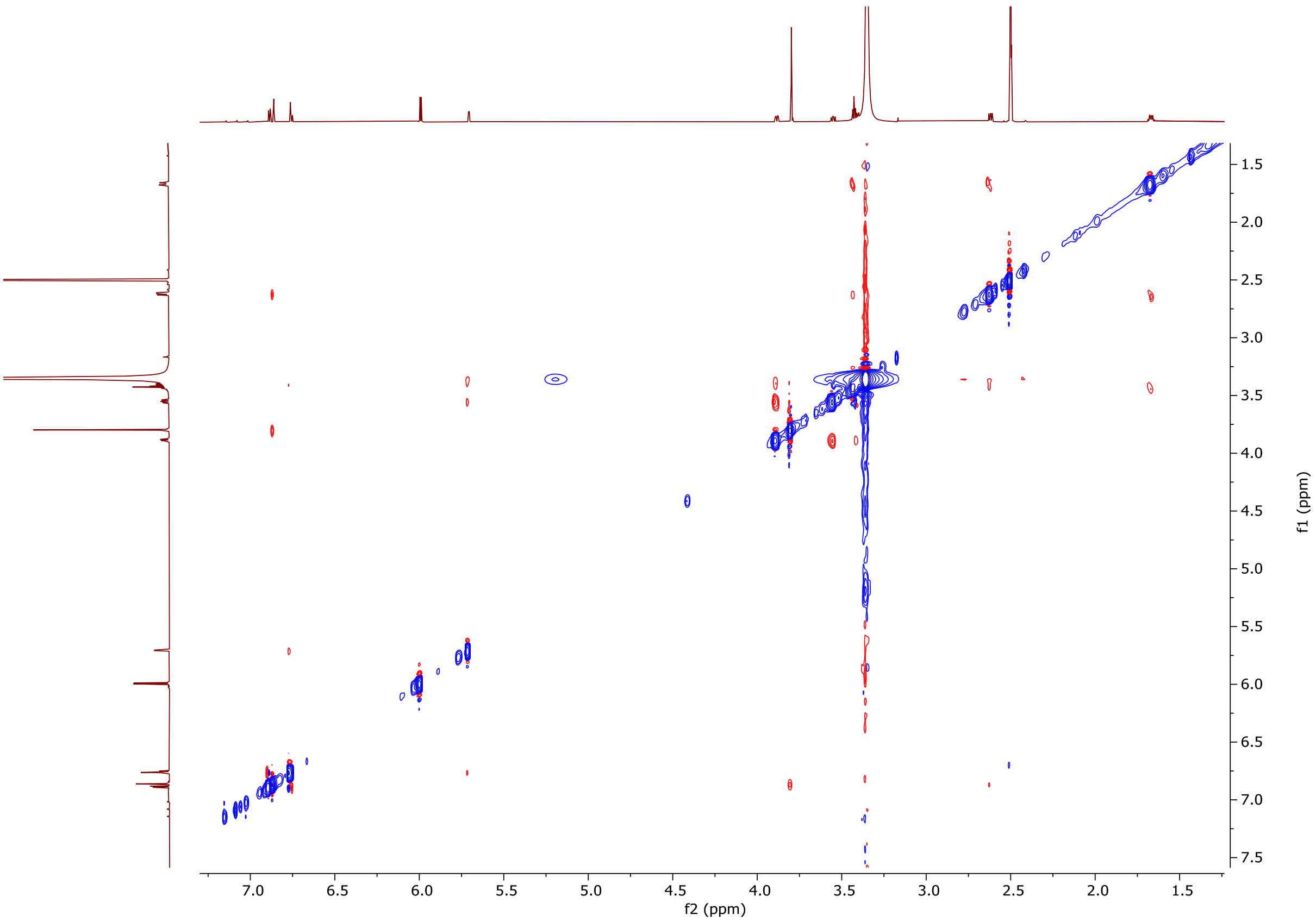

Figure S23 ^{1}H NMR (800 MHz) spectrum of compound **8** in DMSO-d_6

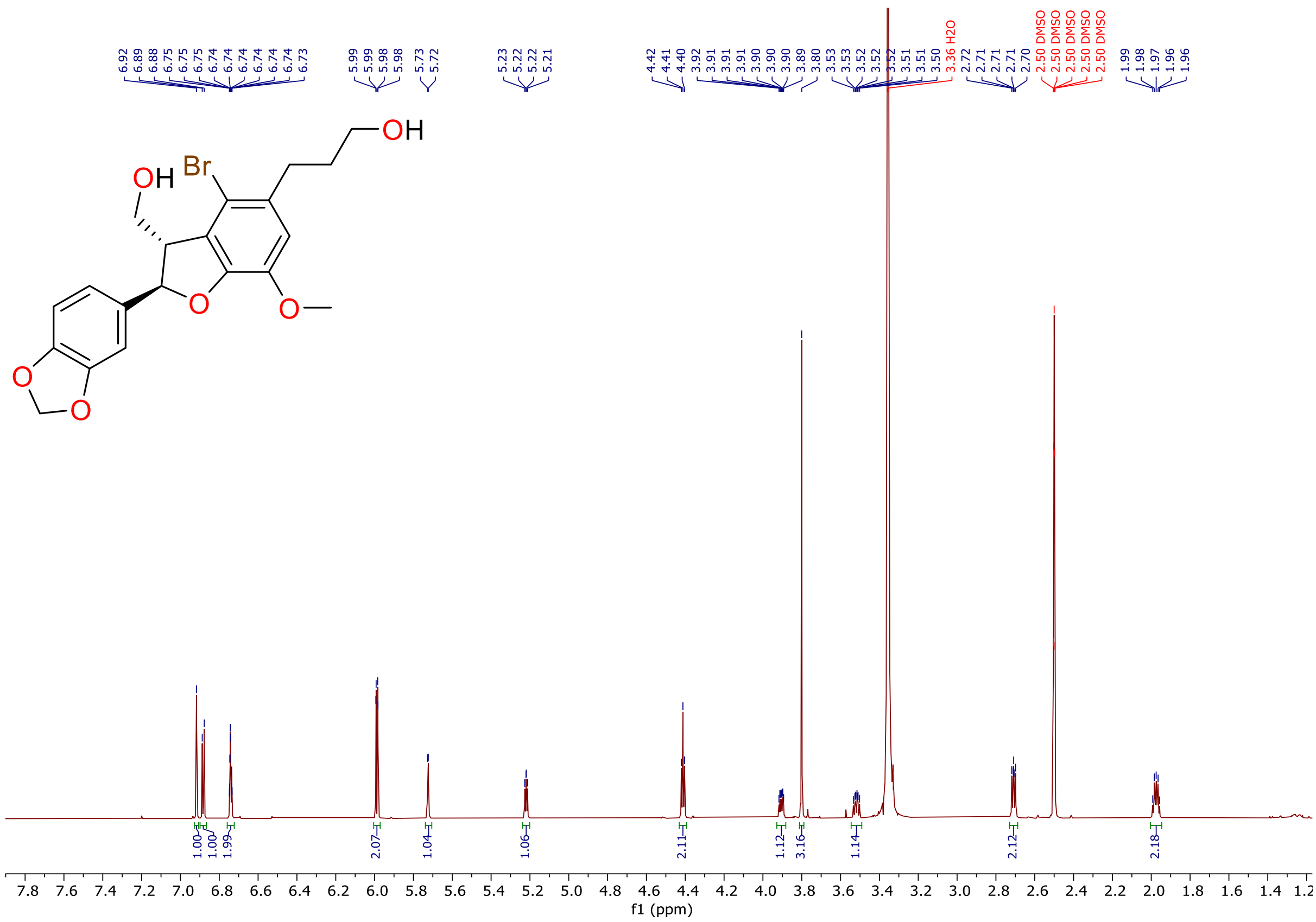

Figure S24 ^{13}C NMR (200 MHz) spectrum of compound **8** in DMSO-d_6

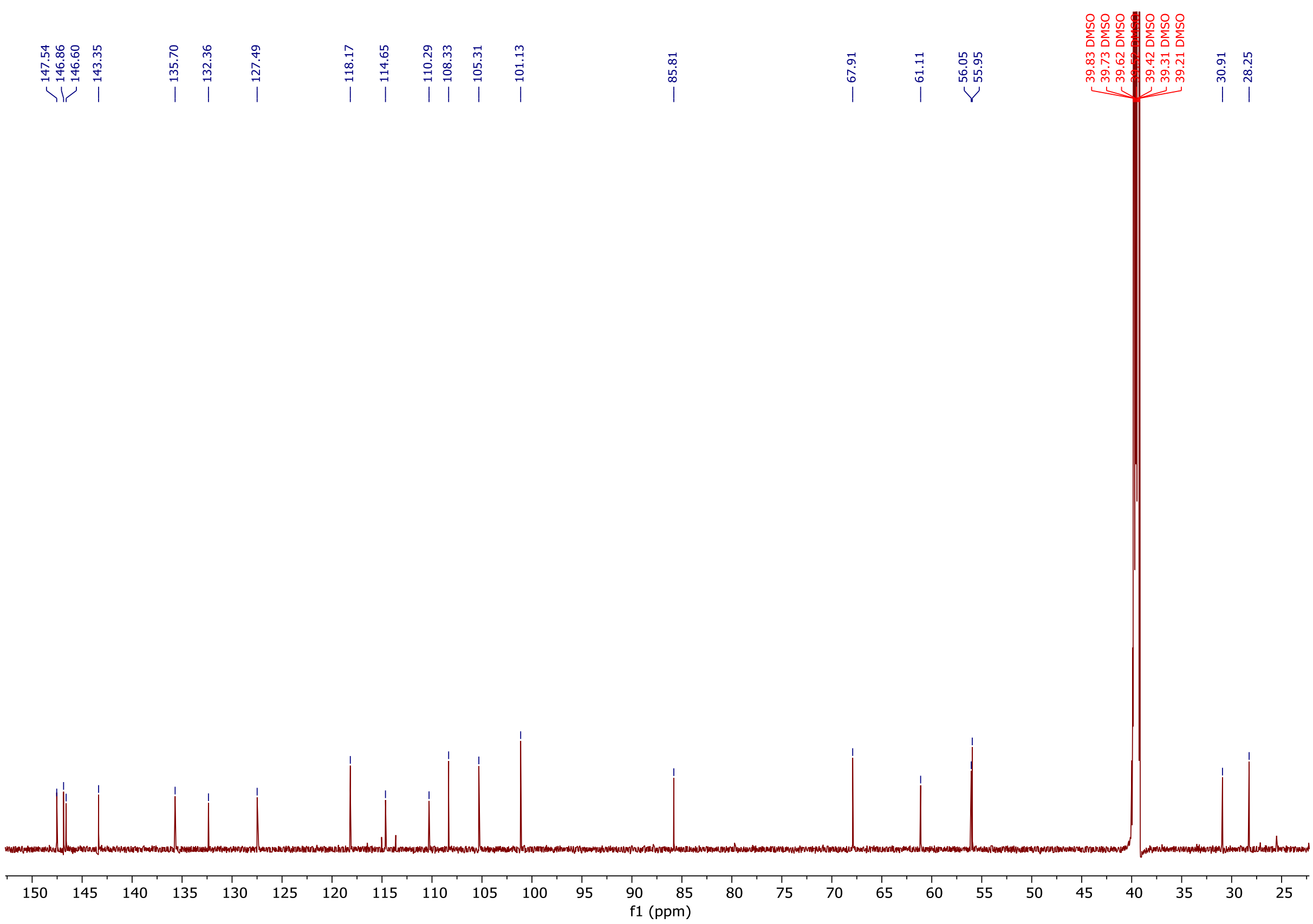

Figure S25 COSY spectrum of compound **8** in DMSO-d_6

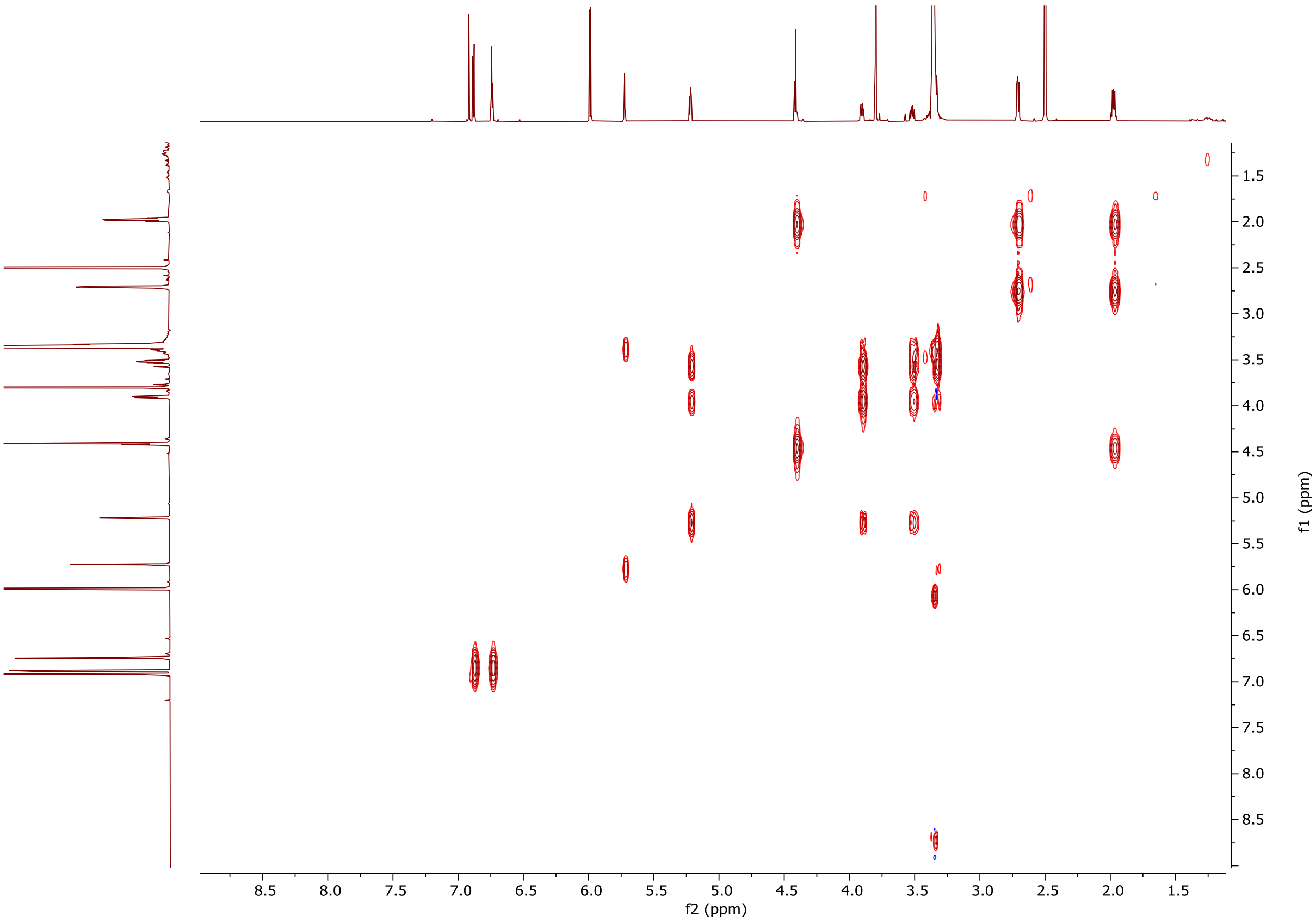

Figure S26 HSQC spectrum of compound **8** in DMSO-d_6

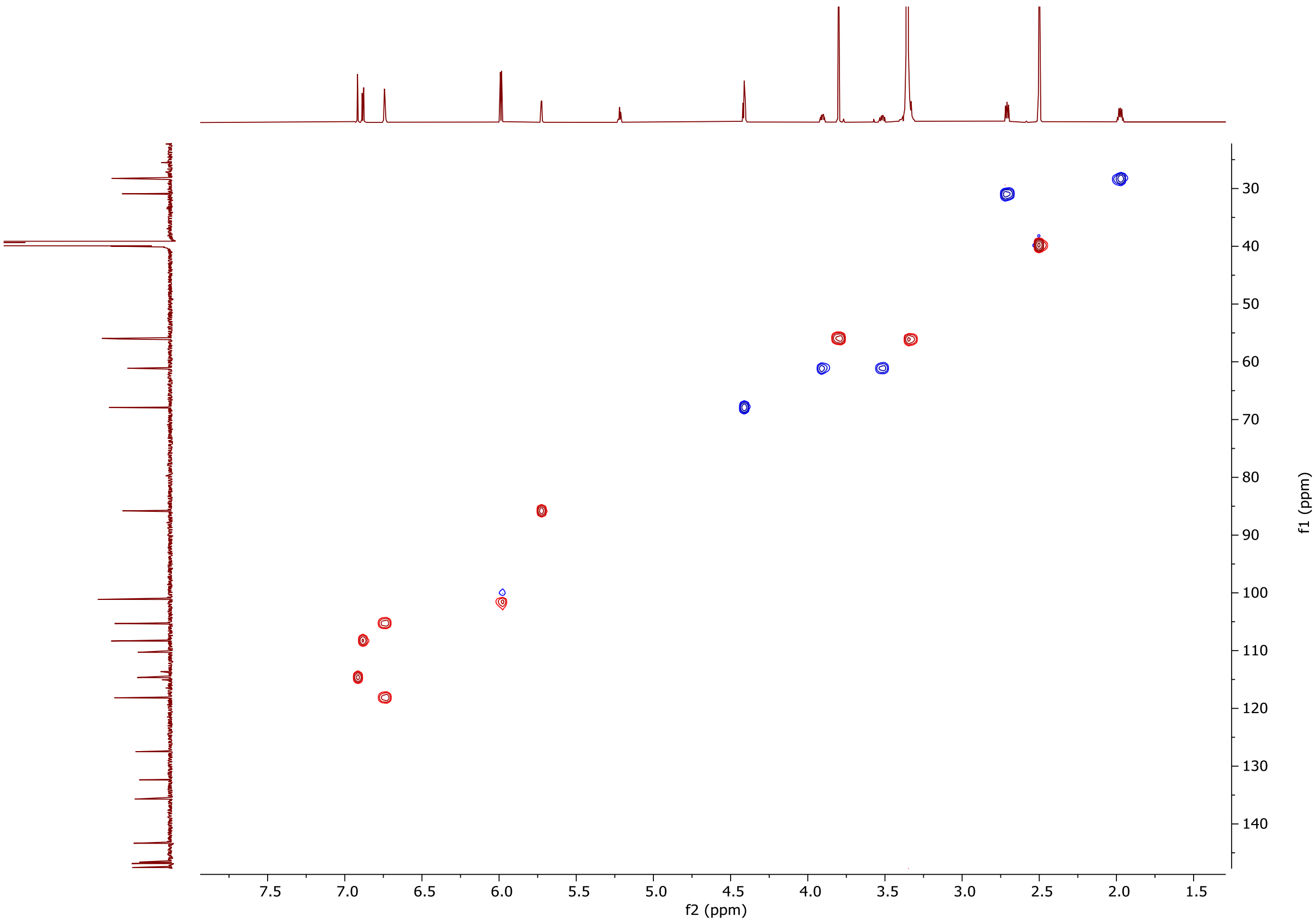

Figure S27 HMBC spectrum of compound **8** in DMSO-d_6

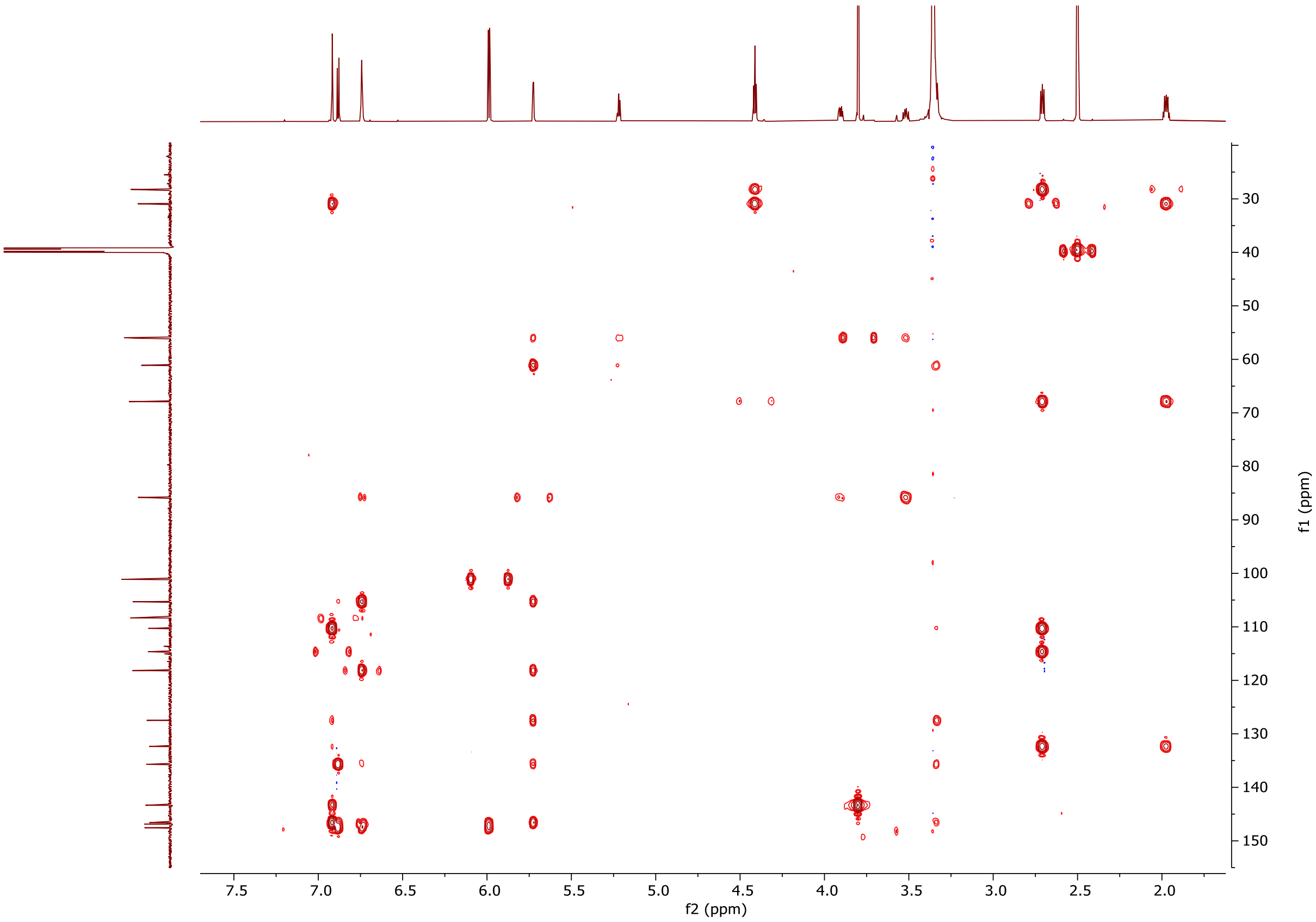

Figure S28 ROESY spectrum of compound **8** in DMSO-d_6

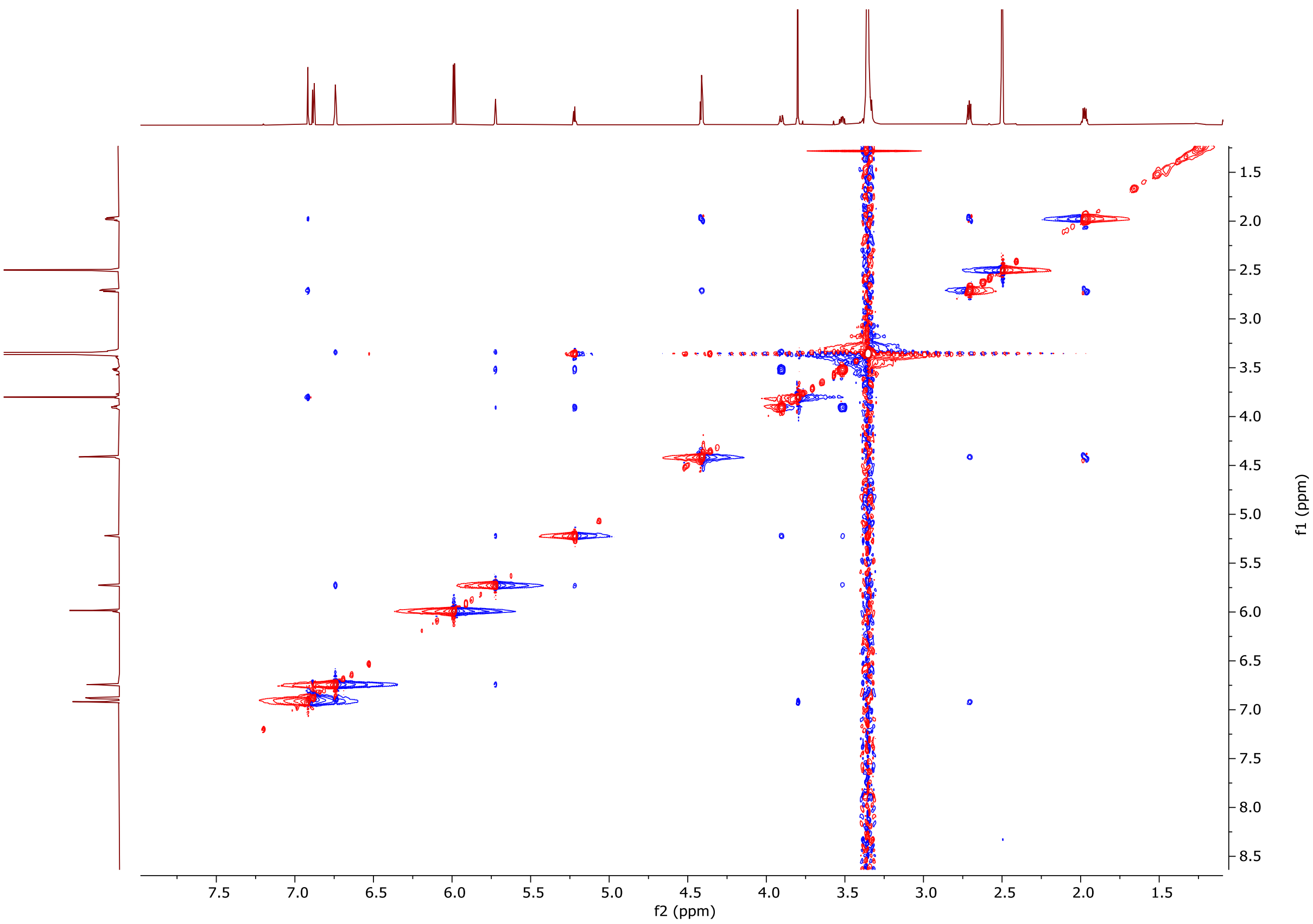

Figure S29 ^{1}H NMR (800 MHz) spectrum of compound **9** in DMSO-d_6

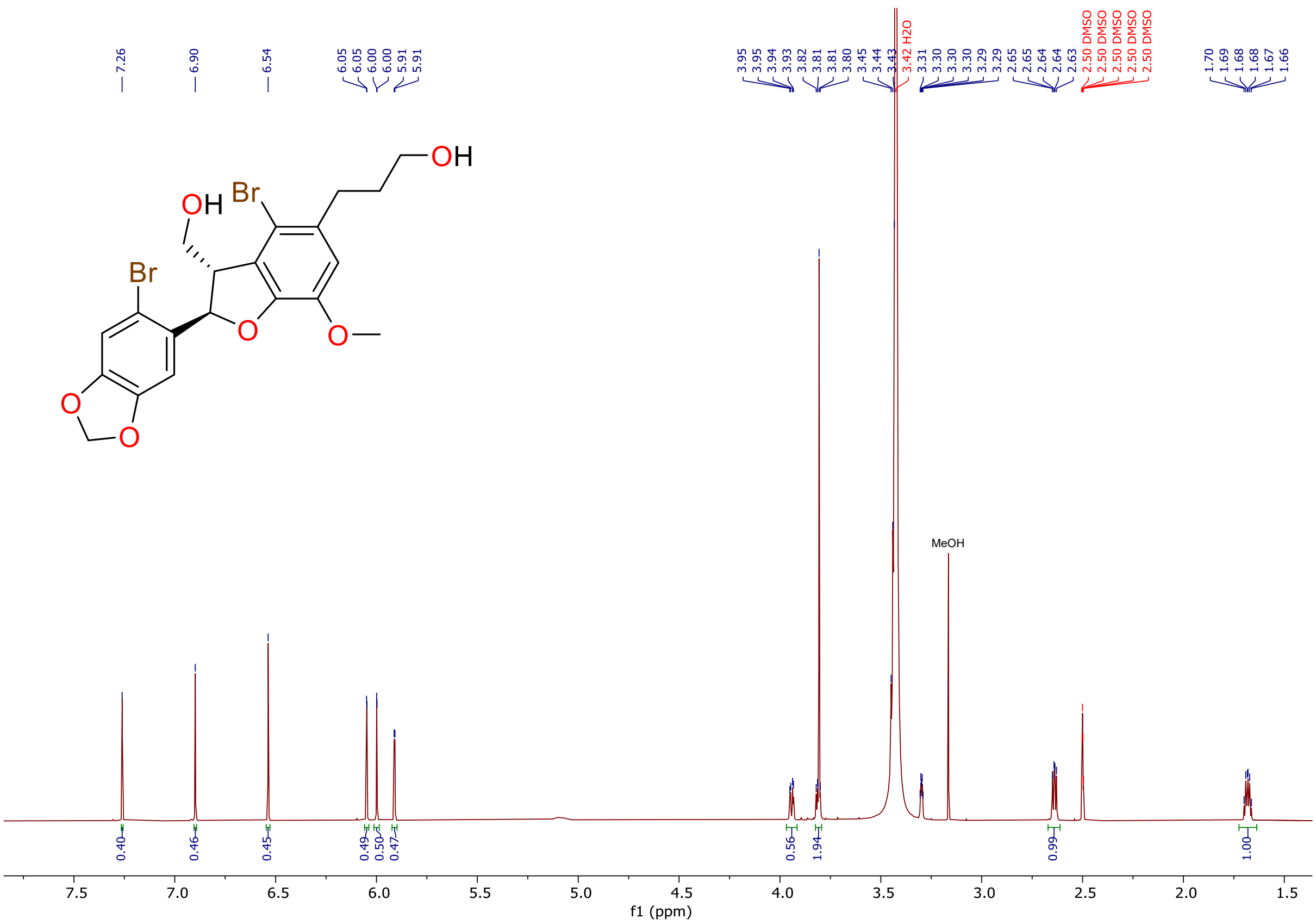

Figure S30 ^{13}C NMR (200 MHz) spectrum of compound **9** in DMSO-d_6

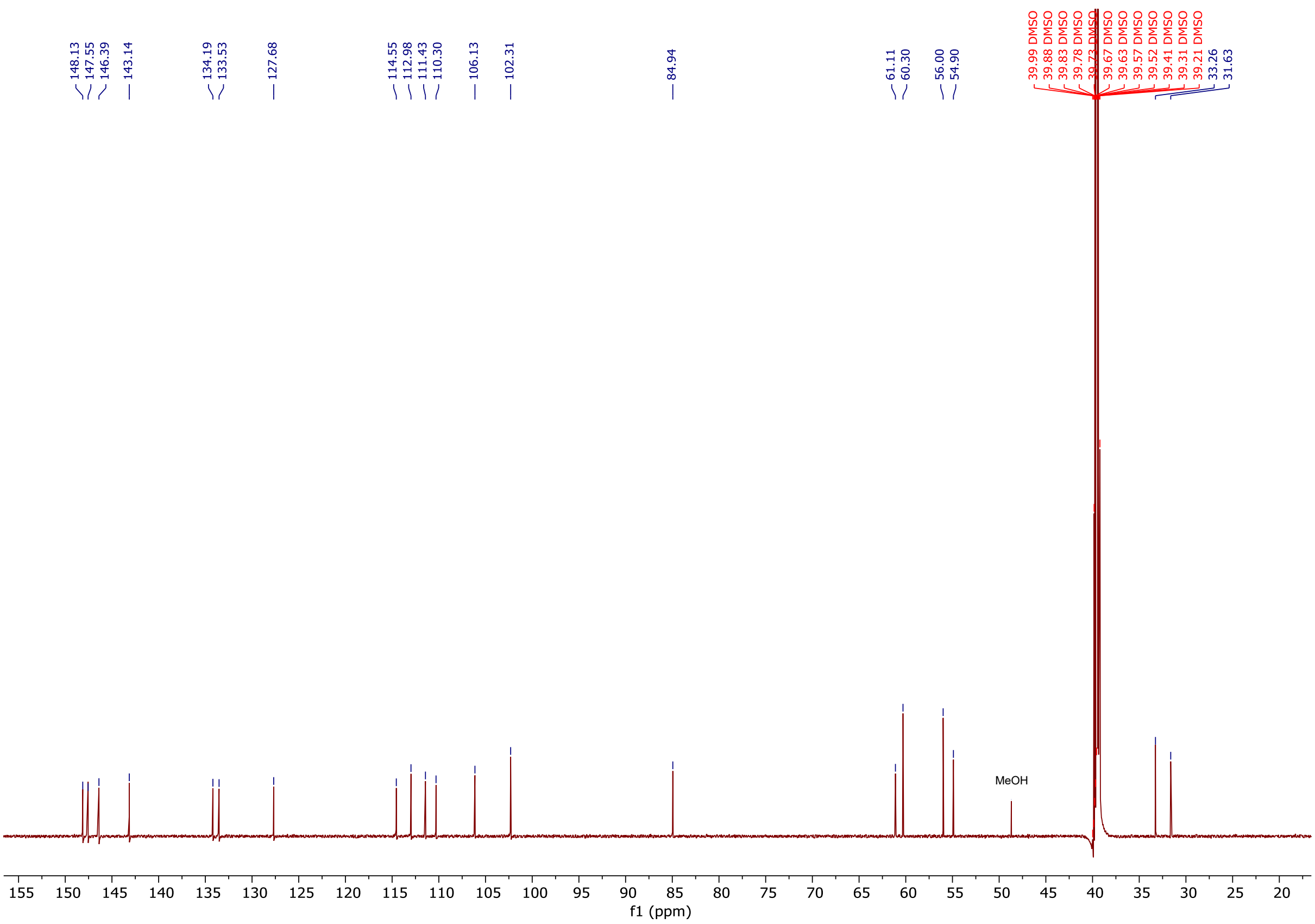

Figure S31 COSY spectrum of compound **9** in DMSO-d_6

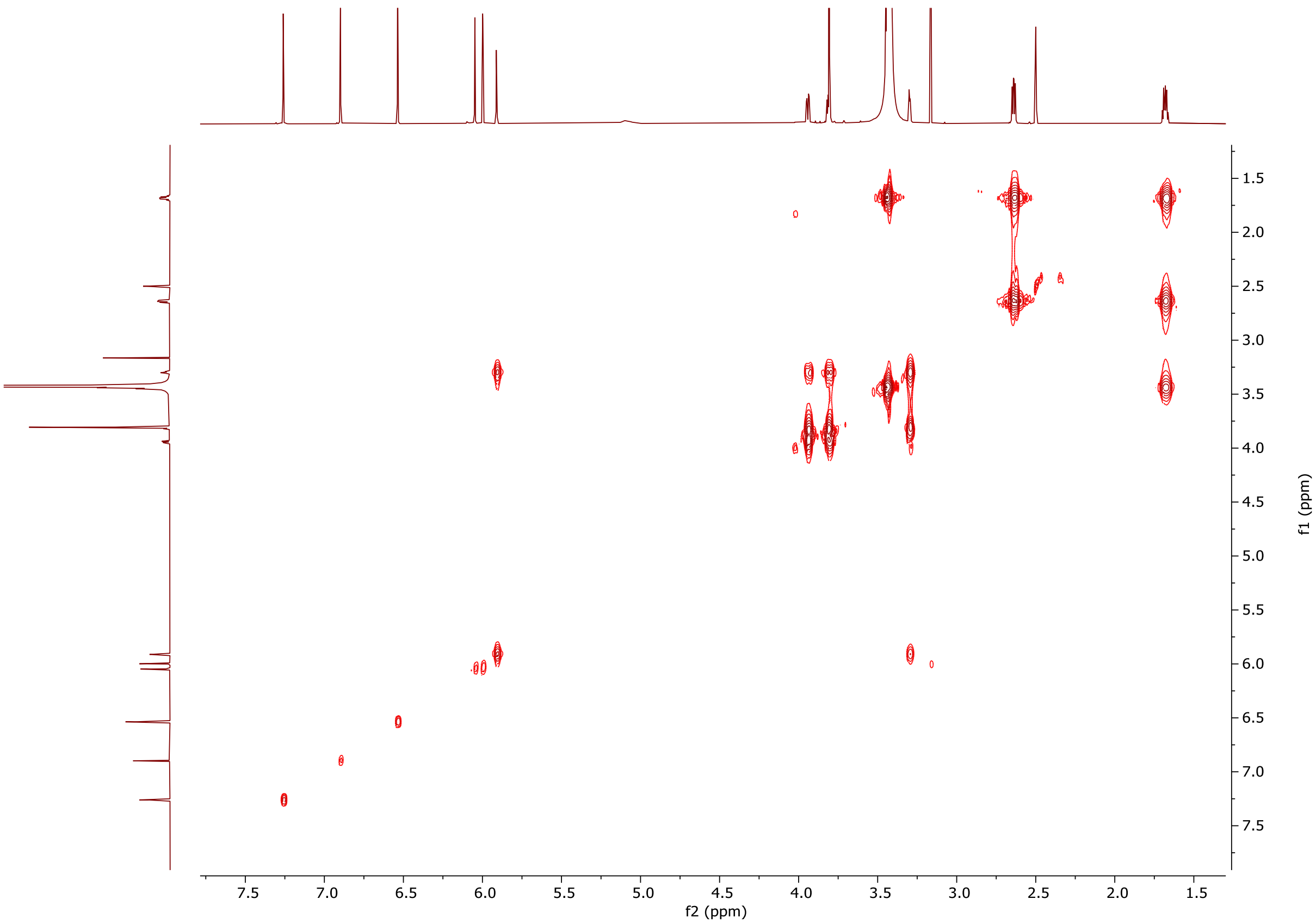

Figure S32 HSQC spectrum of compound **9** in DMSO-d_6

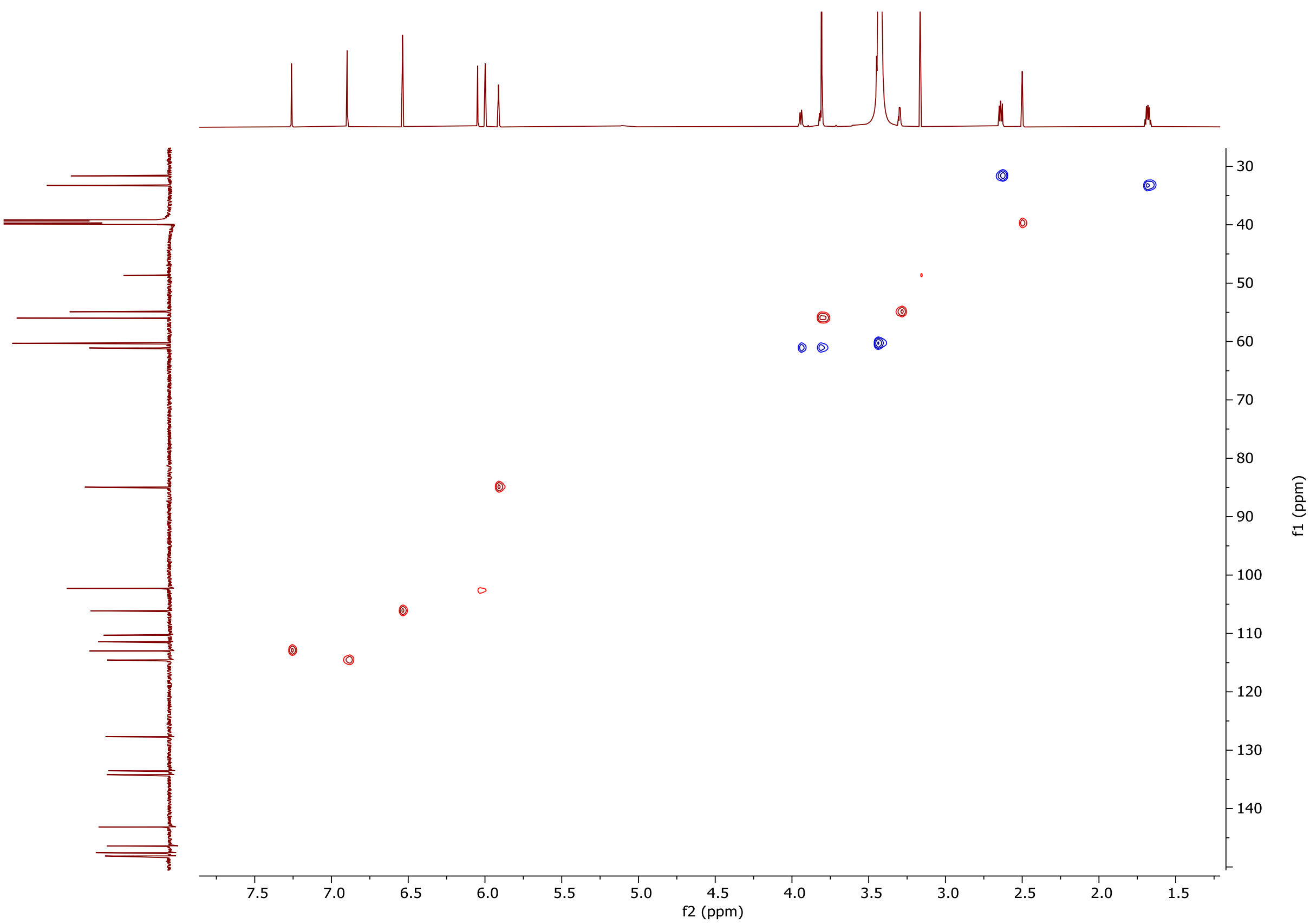

Figure S33 HMBC spectrum of compound **9** in DMSO-d_6

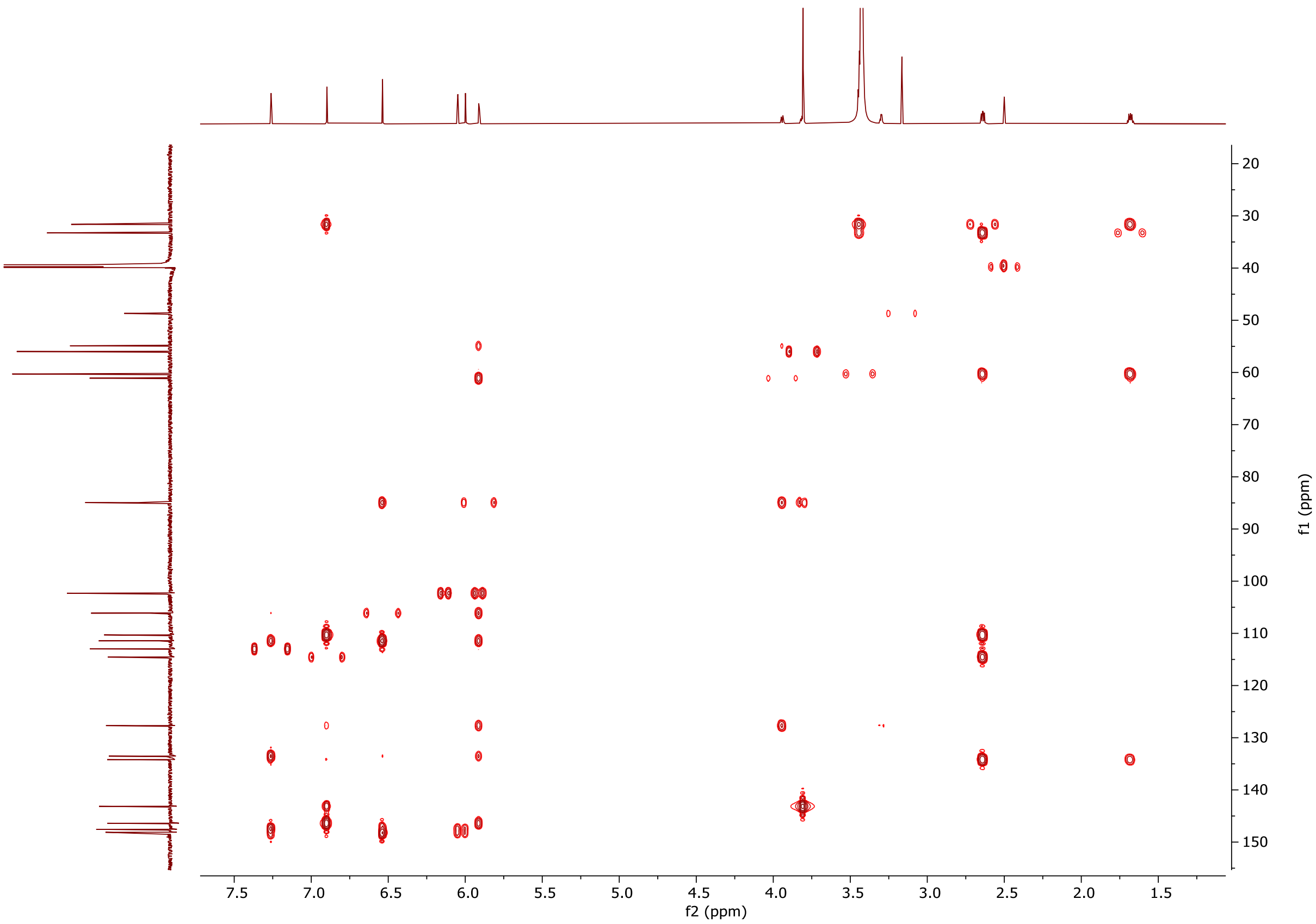

Figure S34 ROESY spectrum of compound **9** in DMSO-d_6

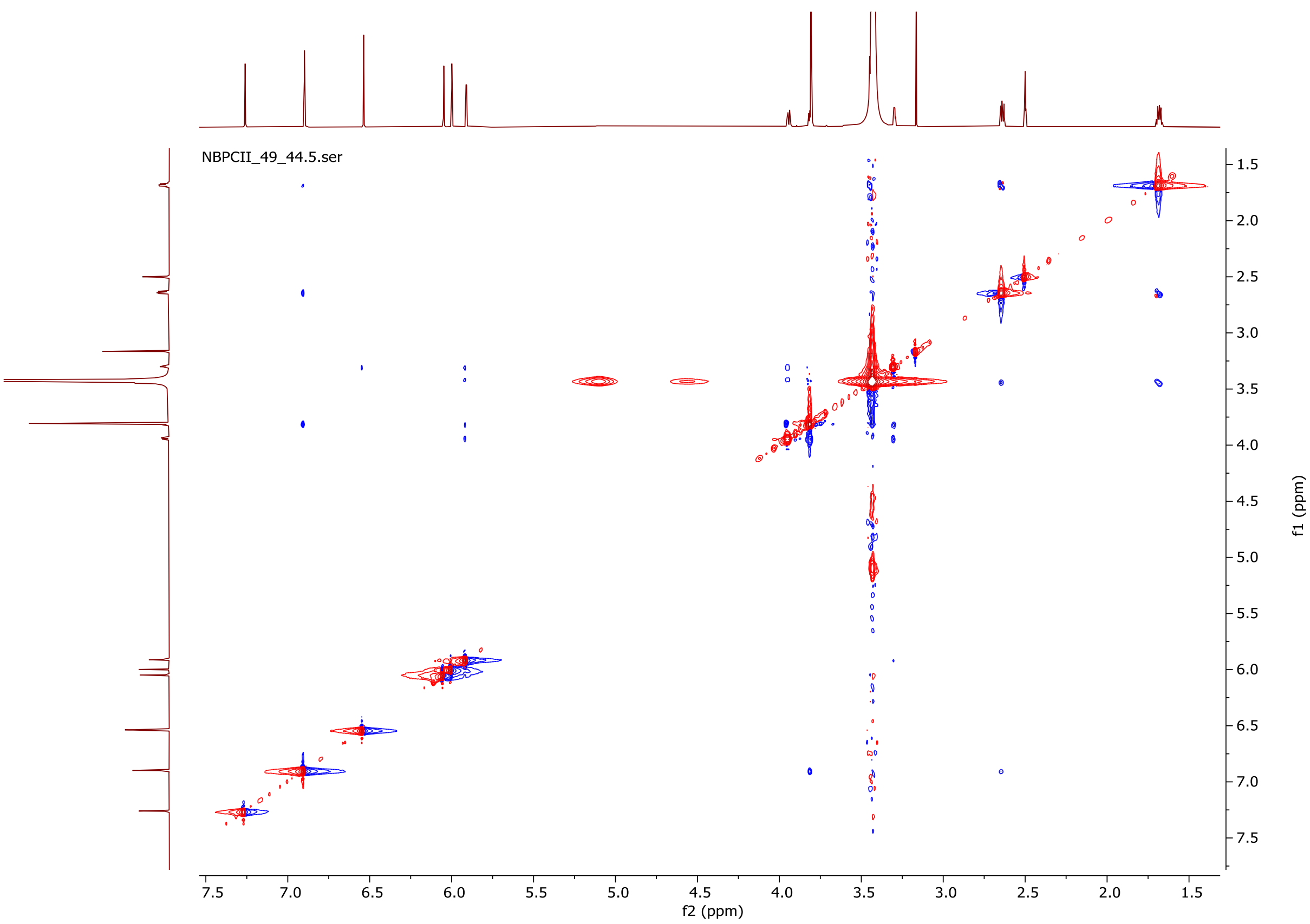

Figure S35 ^{1}H NMR (800 MHz) spectrum of compound **10** in DMSO-d_6

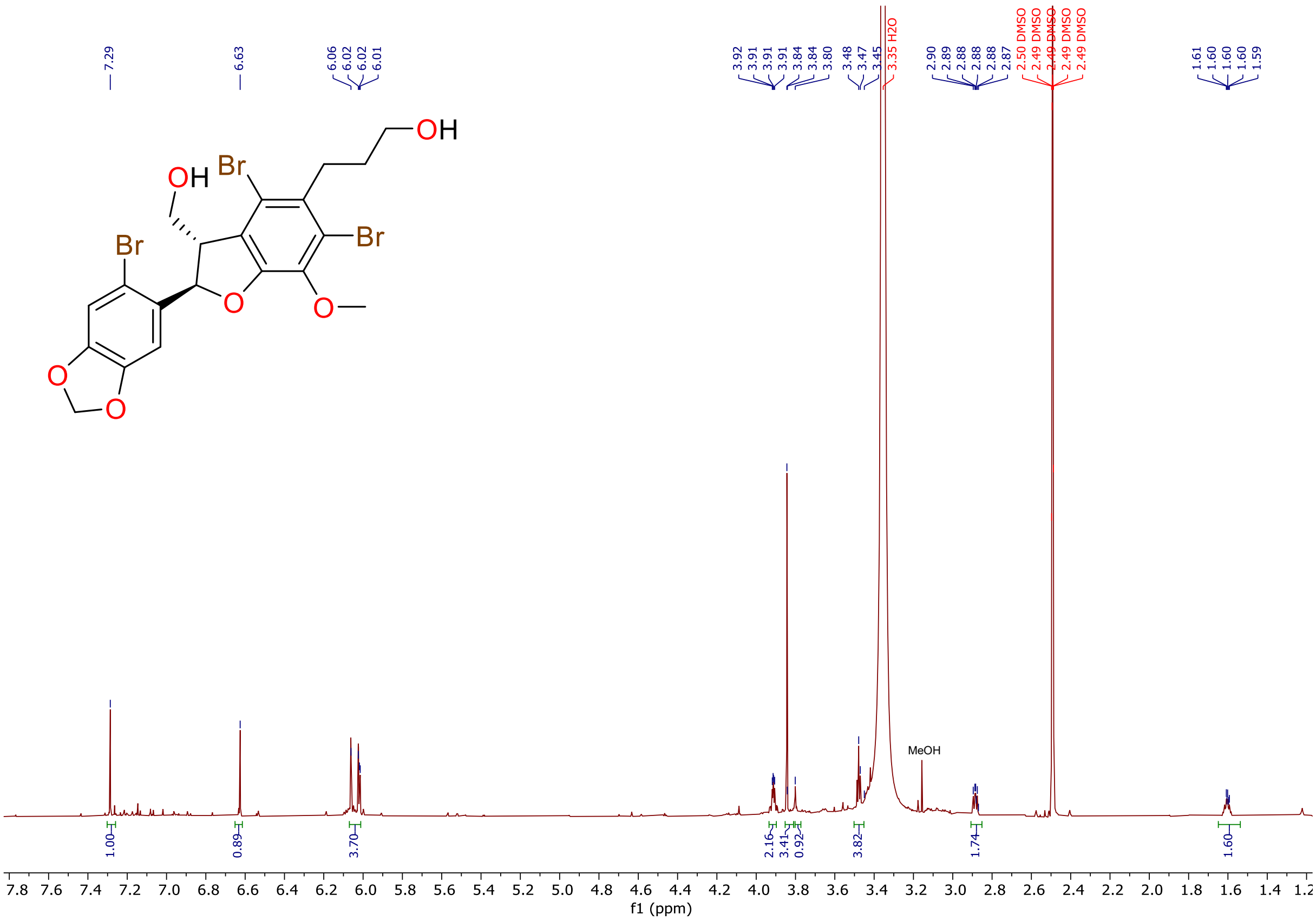

Figure S36 ^{13}C NMR (200 MHz) spectrum of compound **10** in DMSO-d_6

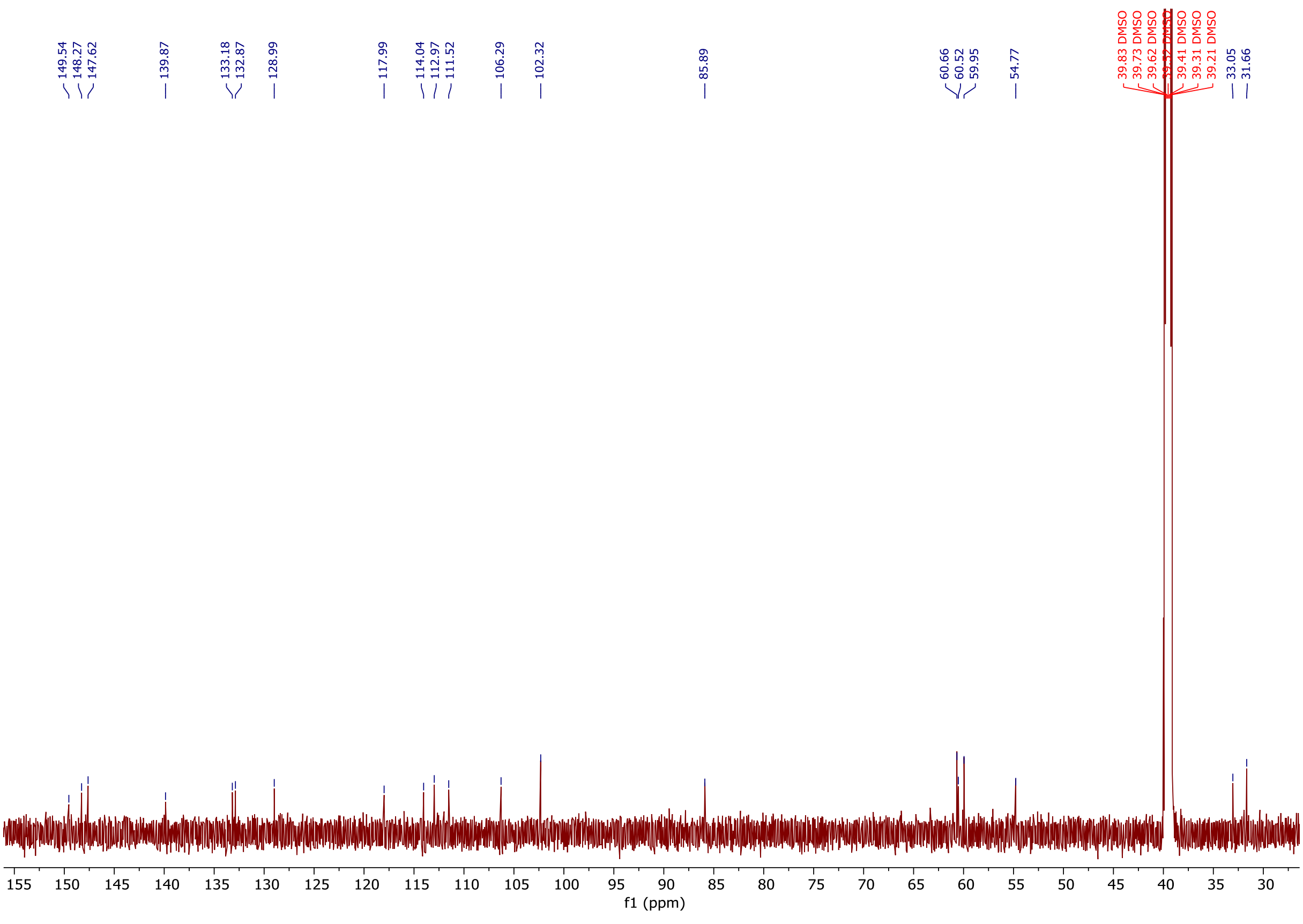

Figure S37 COSY spectrum of compound **10** in DMSO-d_6

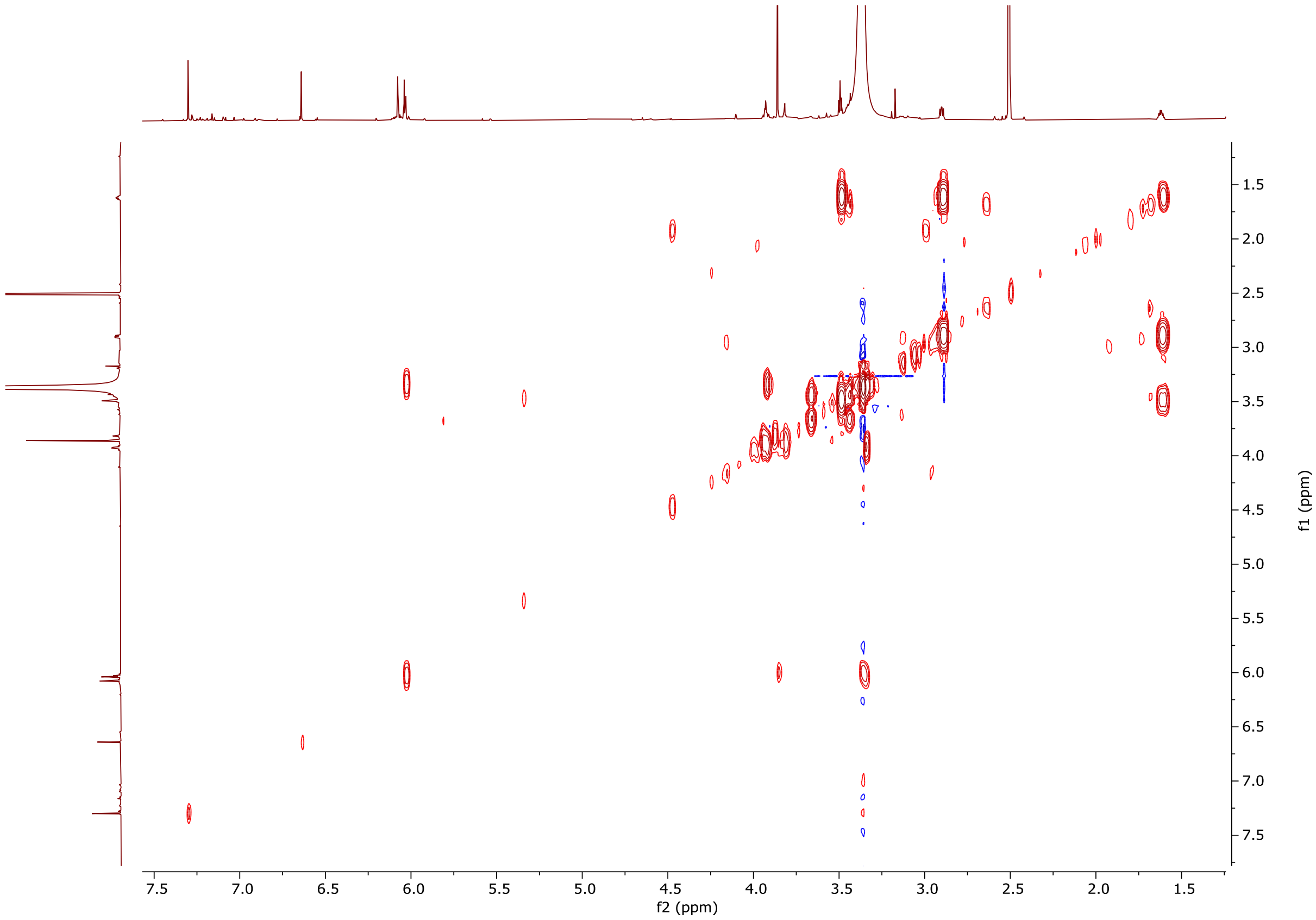

Figure S38 HSQC spectrum of compound **10** in DMSO-d_6

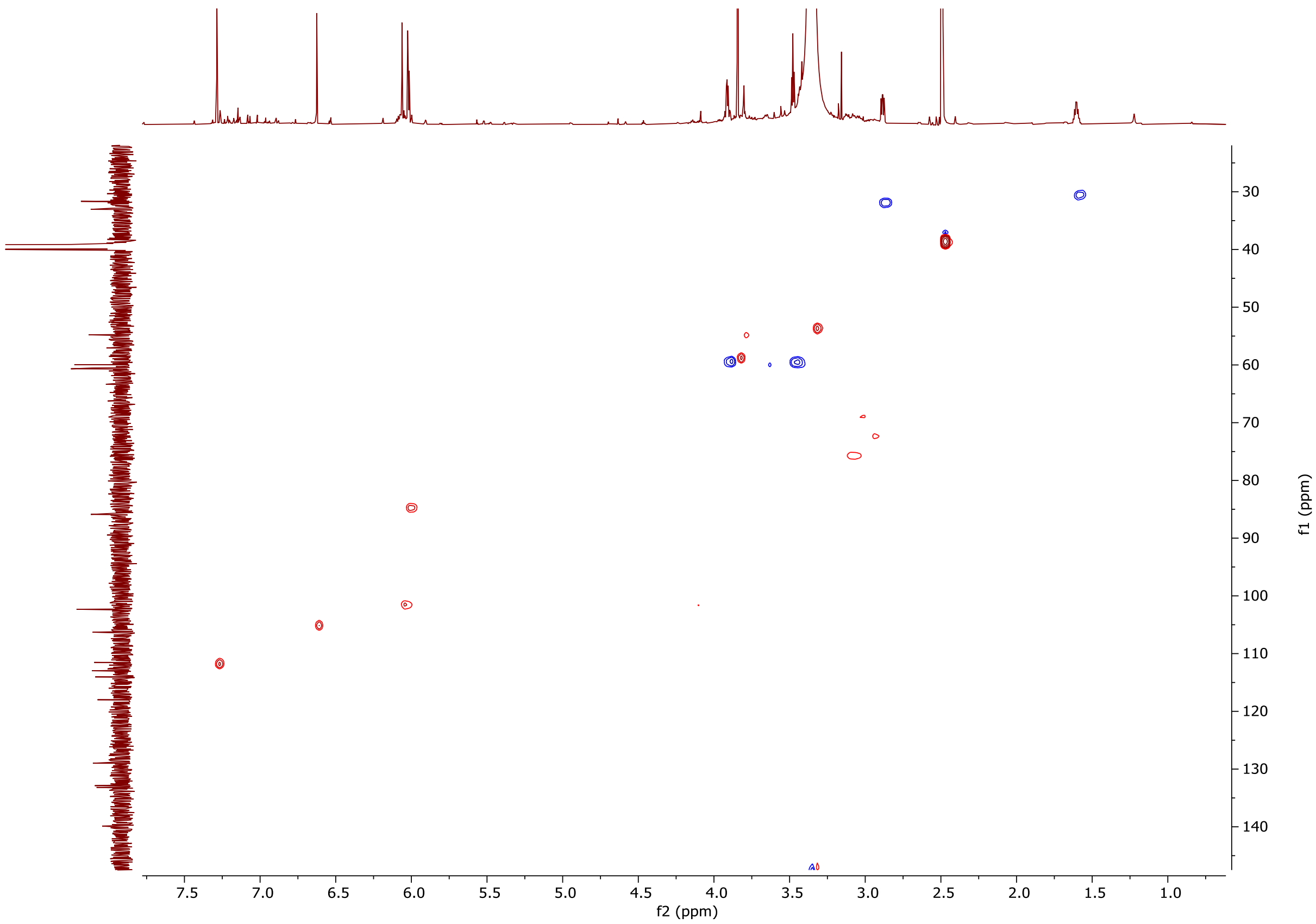

Figure S39 HMBC spectrum of compound **10** in DMSO-d_6

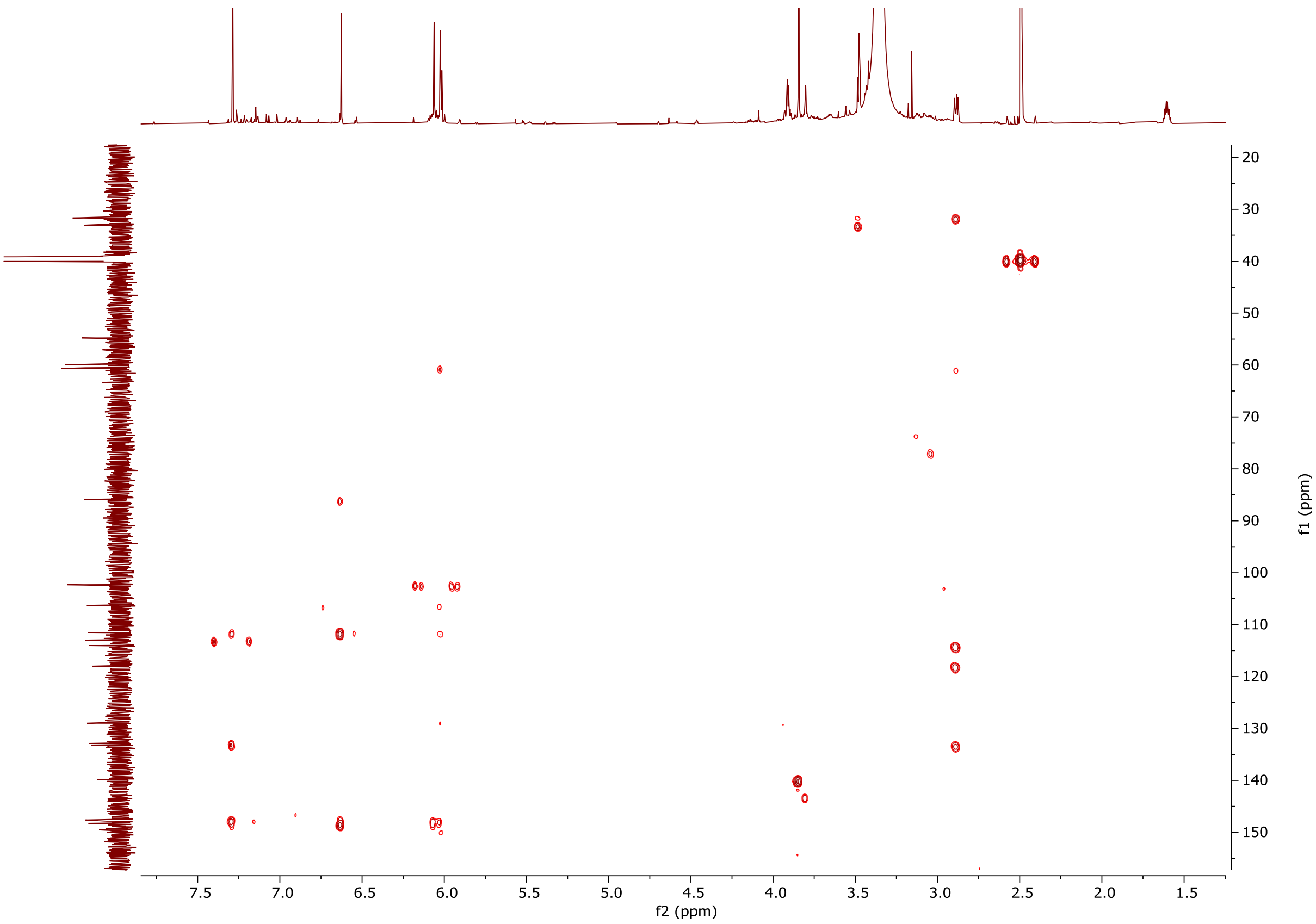

Figure S40 ROESY spectrum of compound **10** in DMSO-d_6

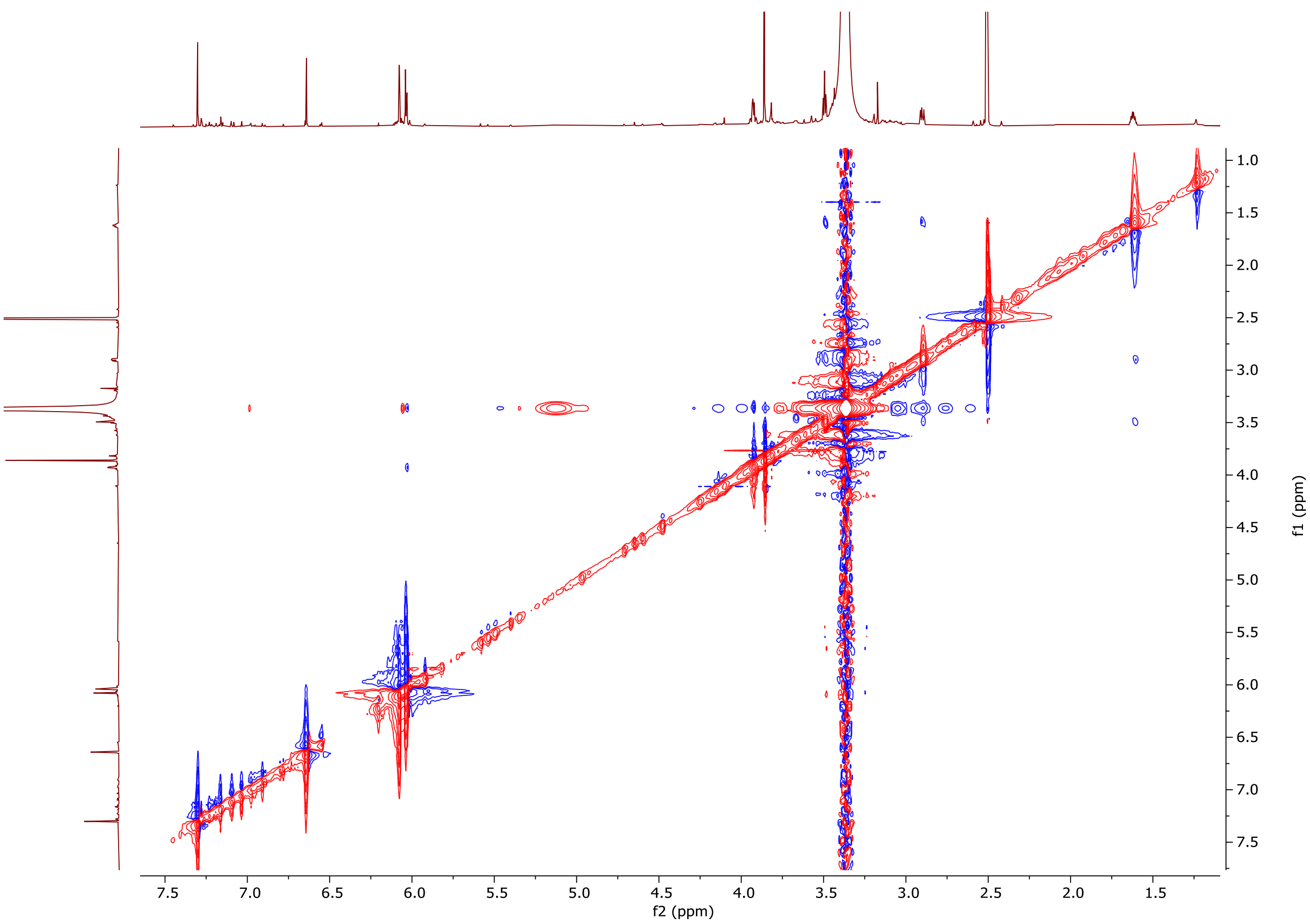

Figure S41 ^{1}H NMR (800 MHz) spectrum of compound **11** in DMSO-d_6

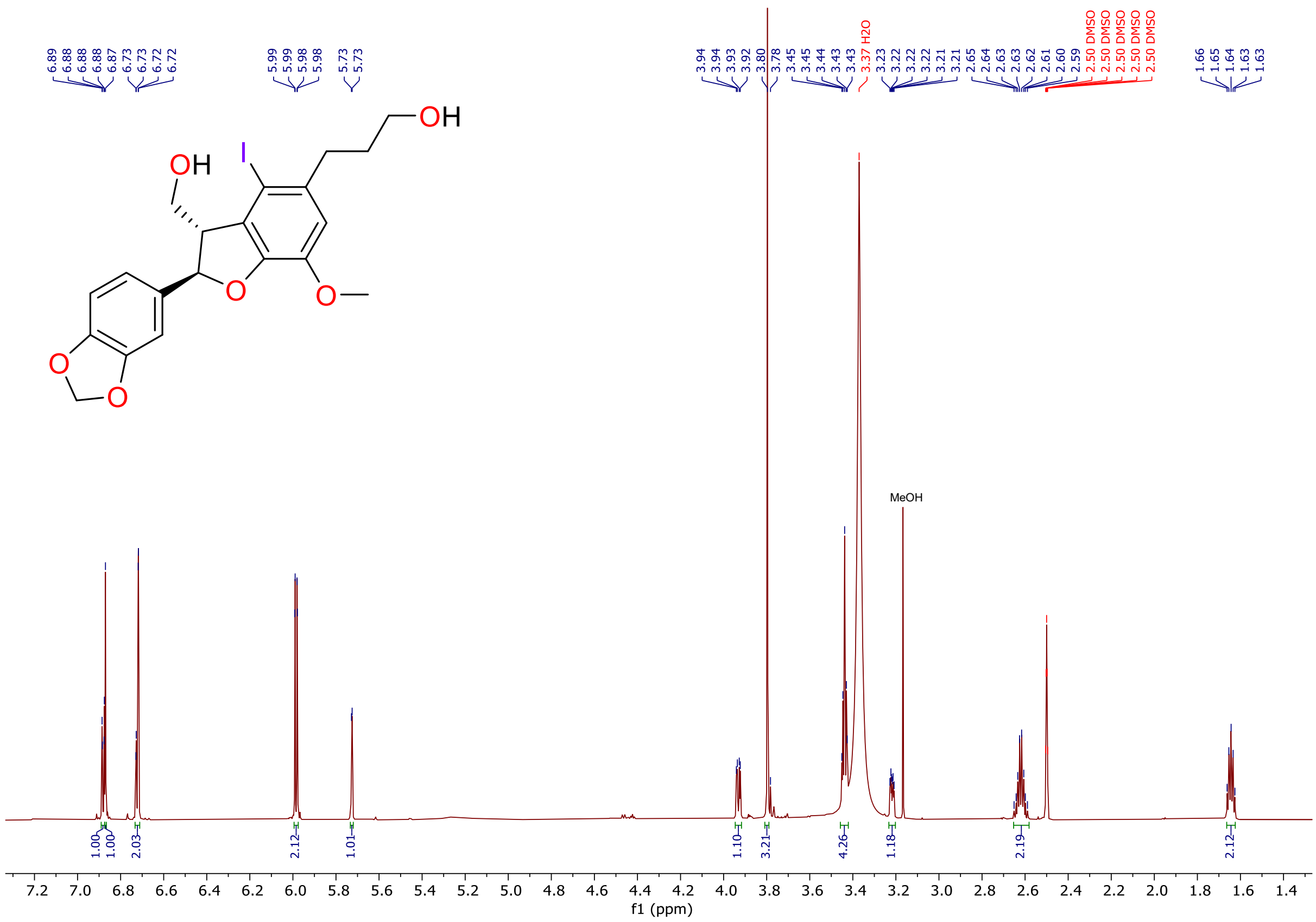

Figure S42 ^{13}C NMR (200 MHz) spectrum of compound **11** in DMSO-d_6

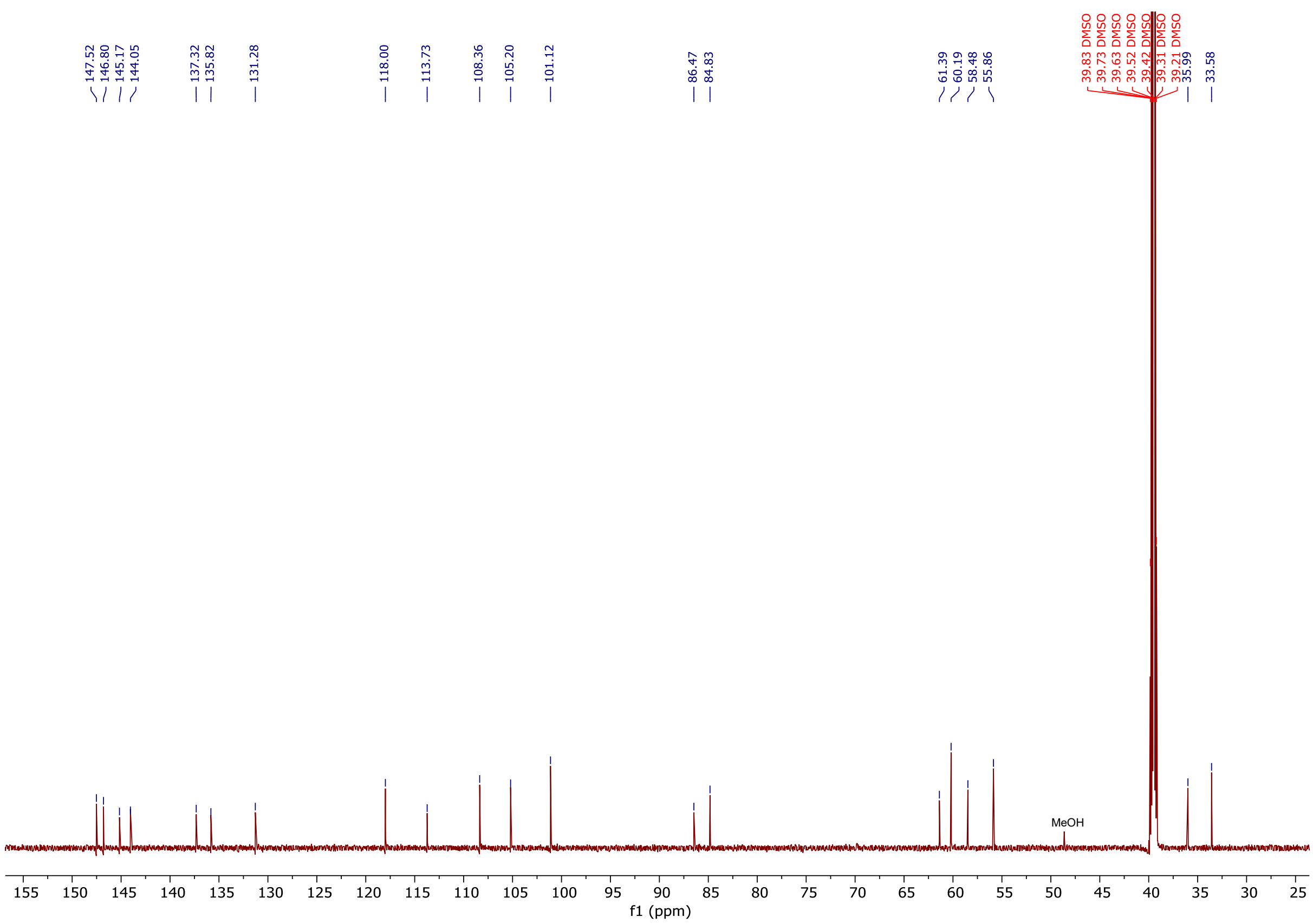

Figure S43 COSY spectrum of compound **11** in DMSO-d_6

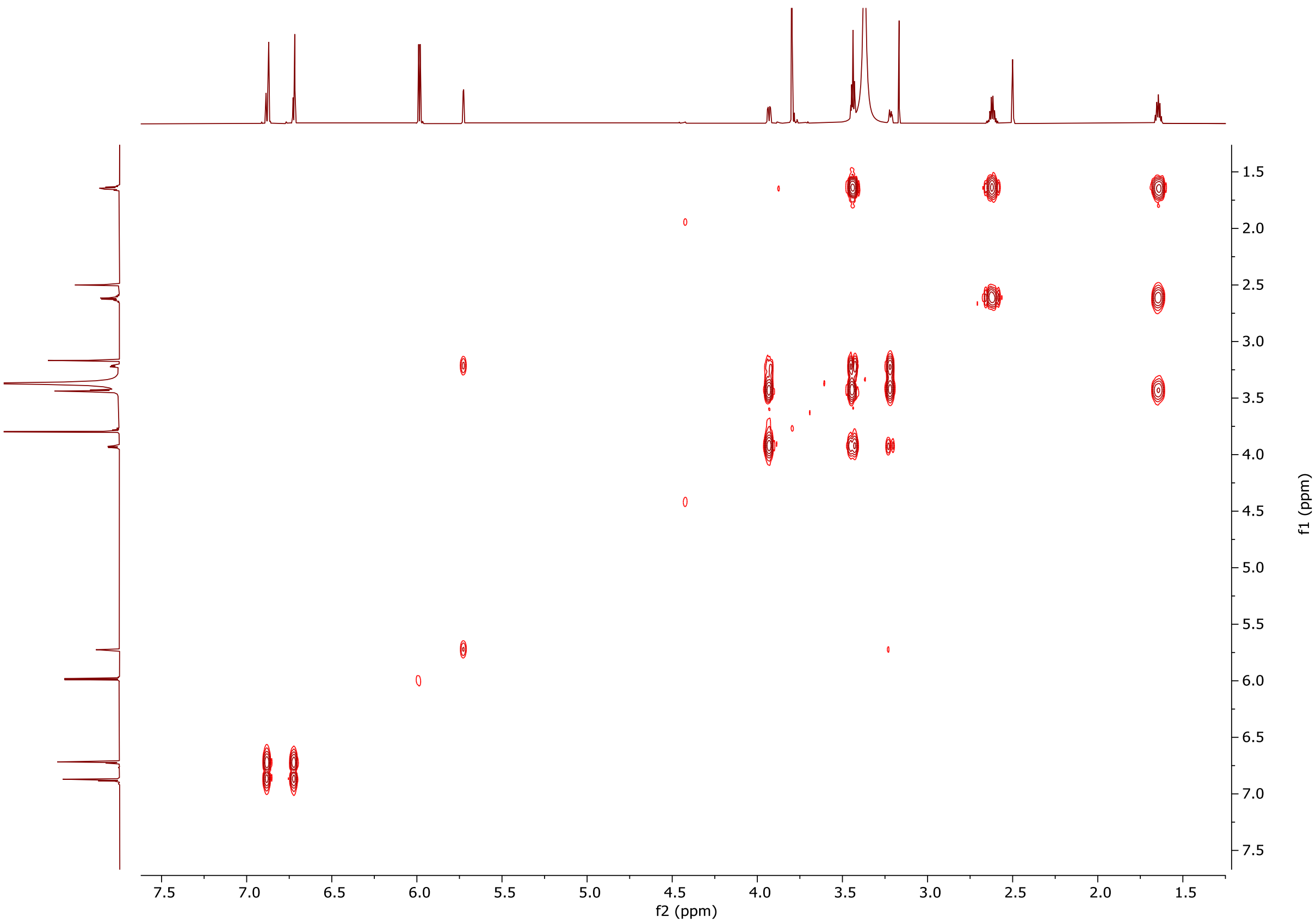

Figure S44 HSQC spectrum of compound **11** in DMSO-d_6

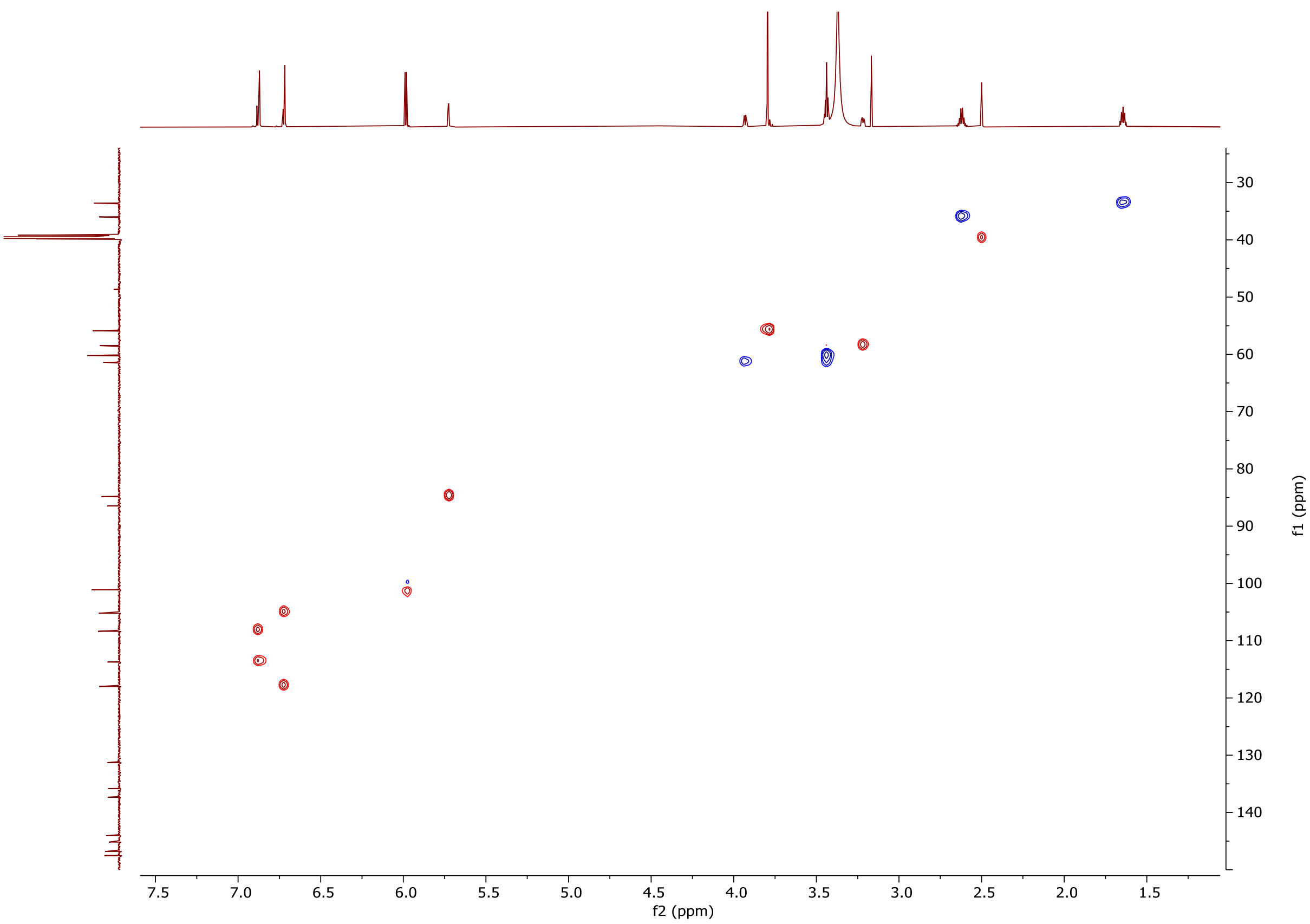

Figure S45 HMBC spectrum of compound **11** in DMSO-d_6

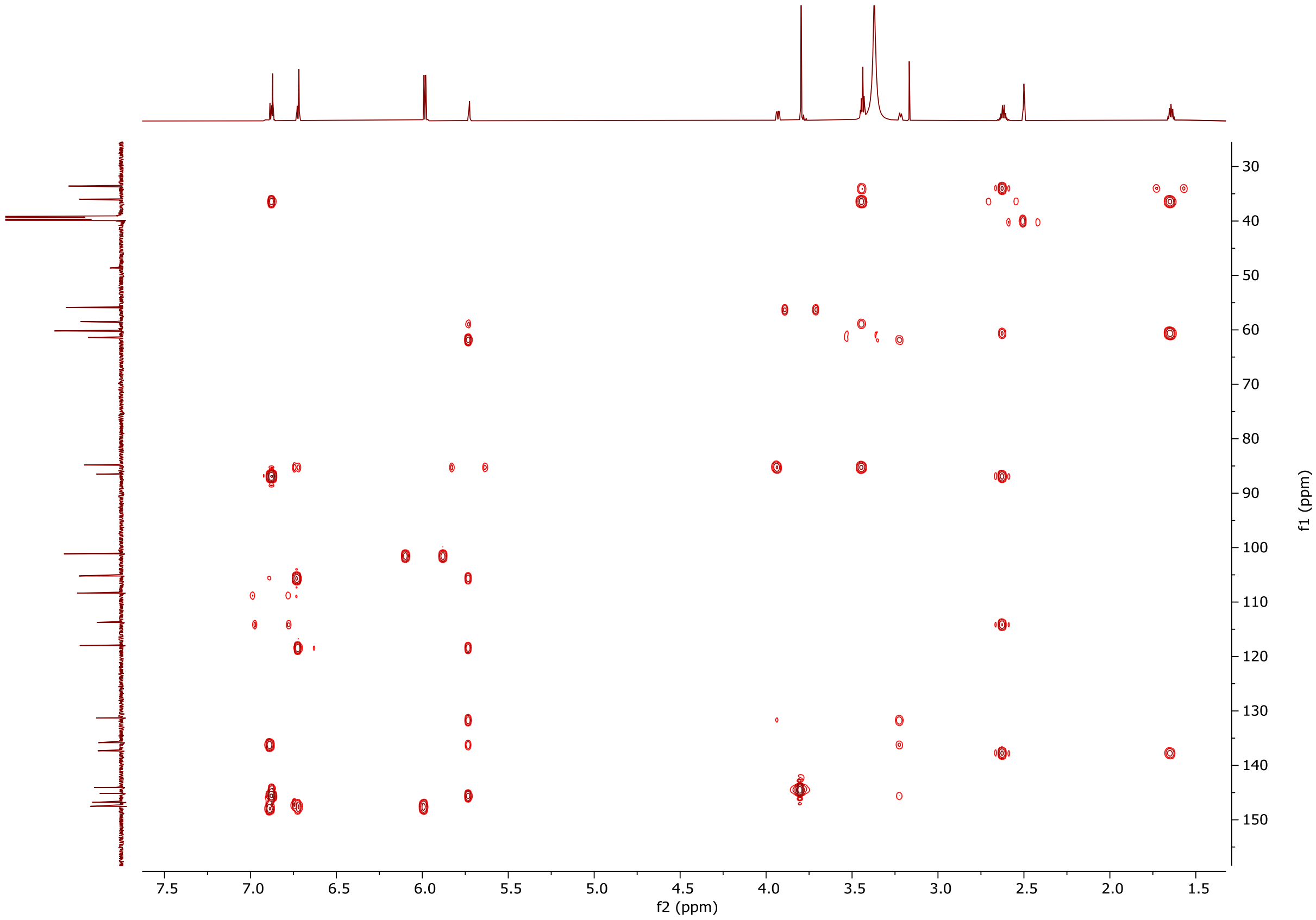

Figure S46 ROESY spectrum of compound **11** in DMSO-d_6

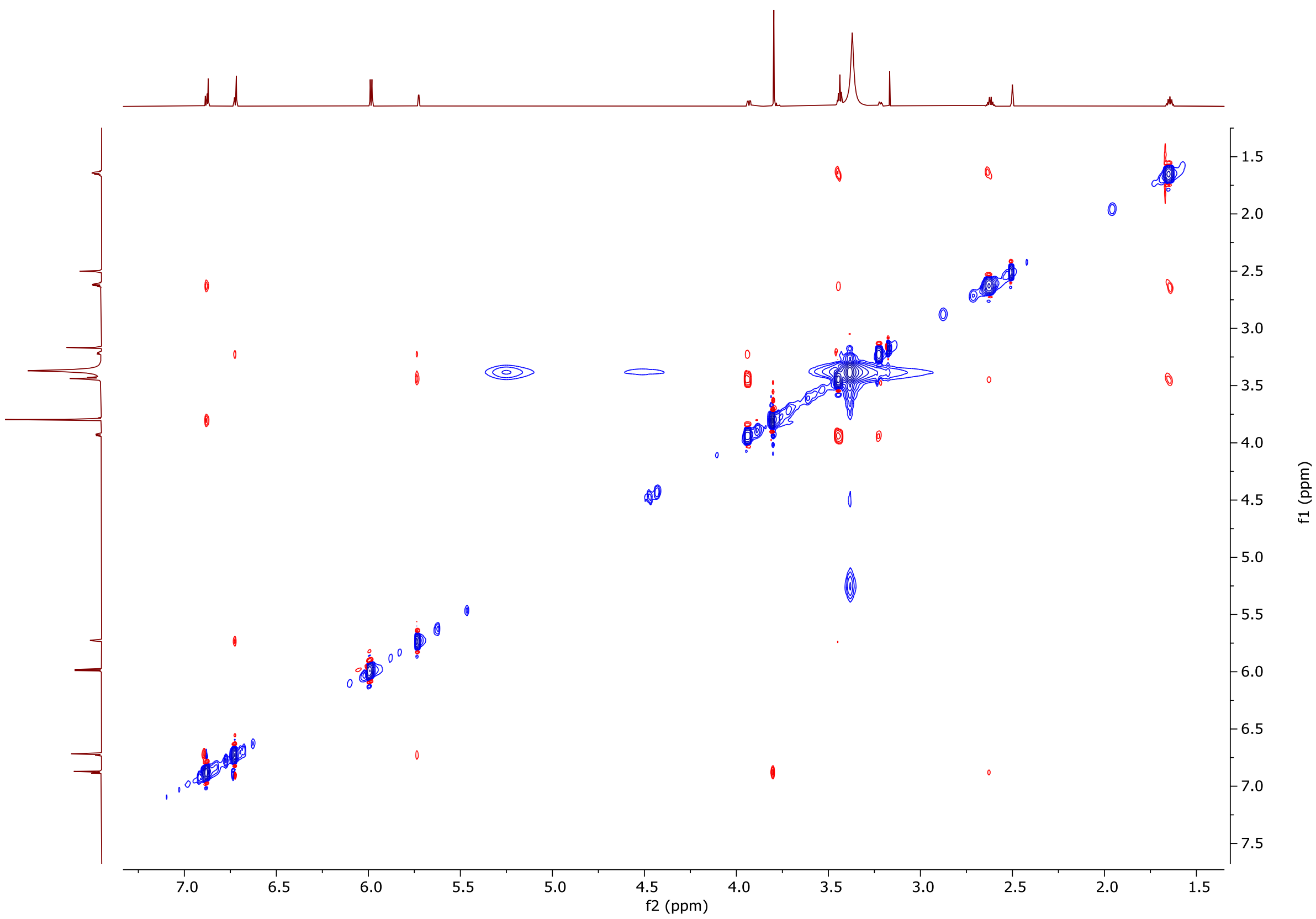

Figure S47 ^{1}H NMR (800 MHz) spectrum of compound **12** in DMSO-d_6

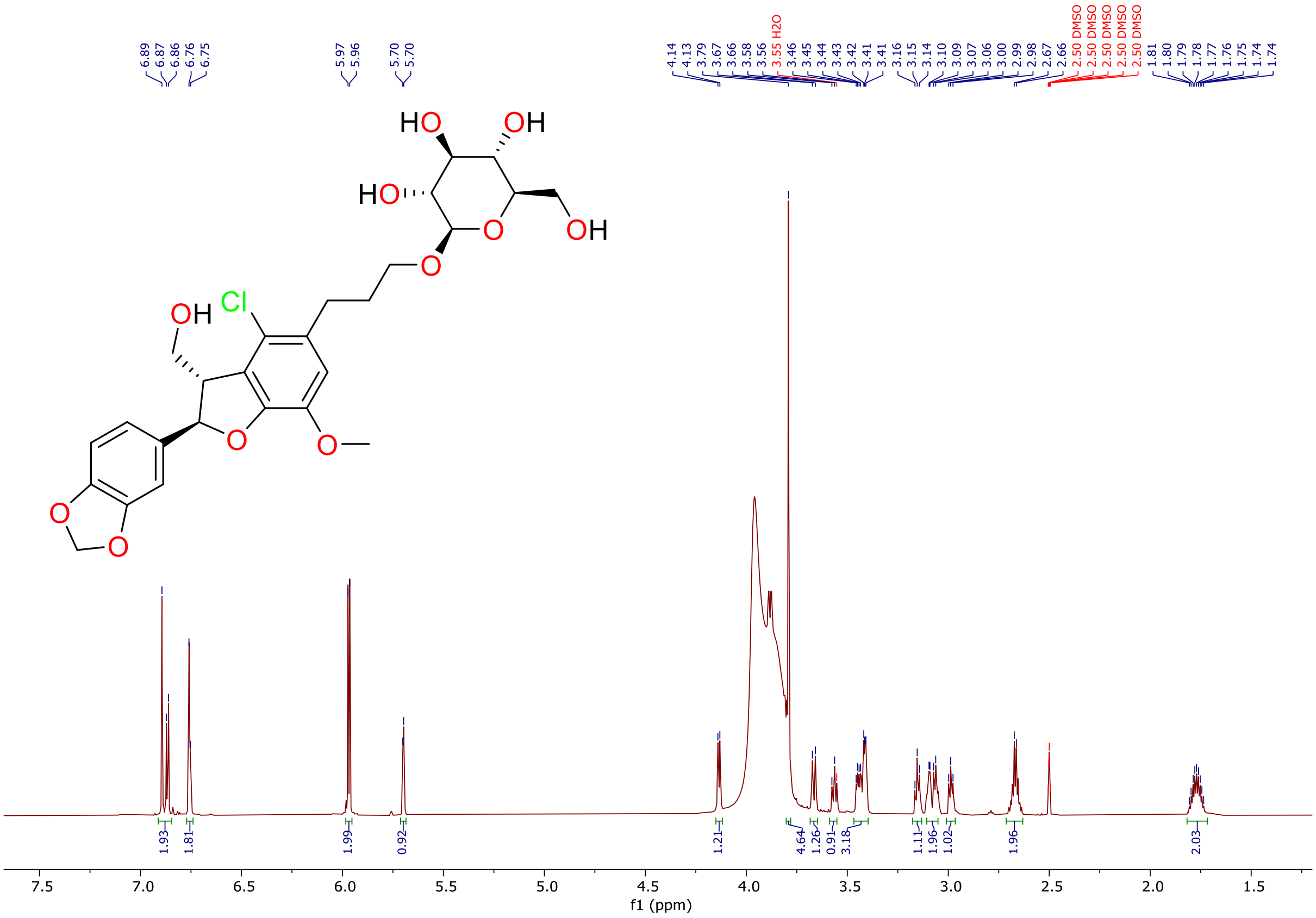

Figure S48 ^{13}C NMR (200 MHz) spectrum of compound **12** in DMSO-d_6

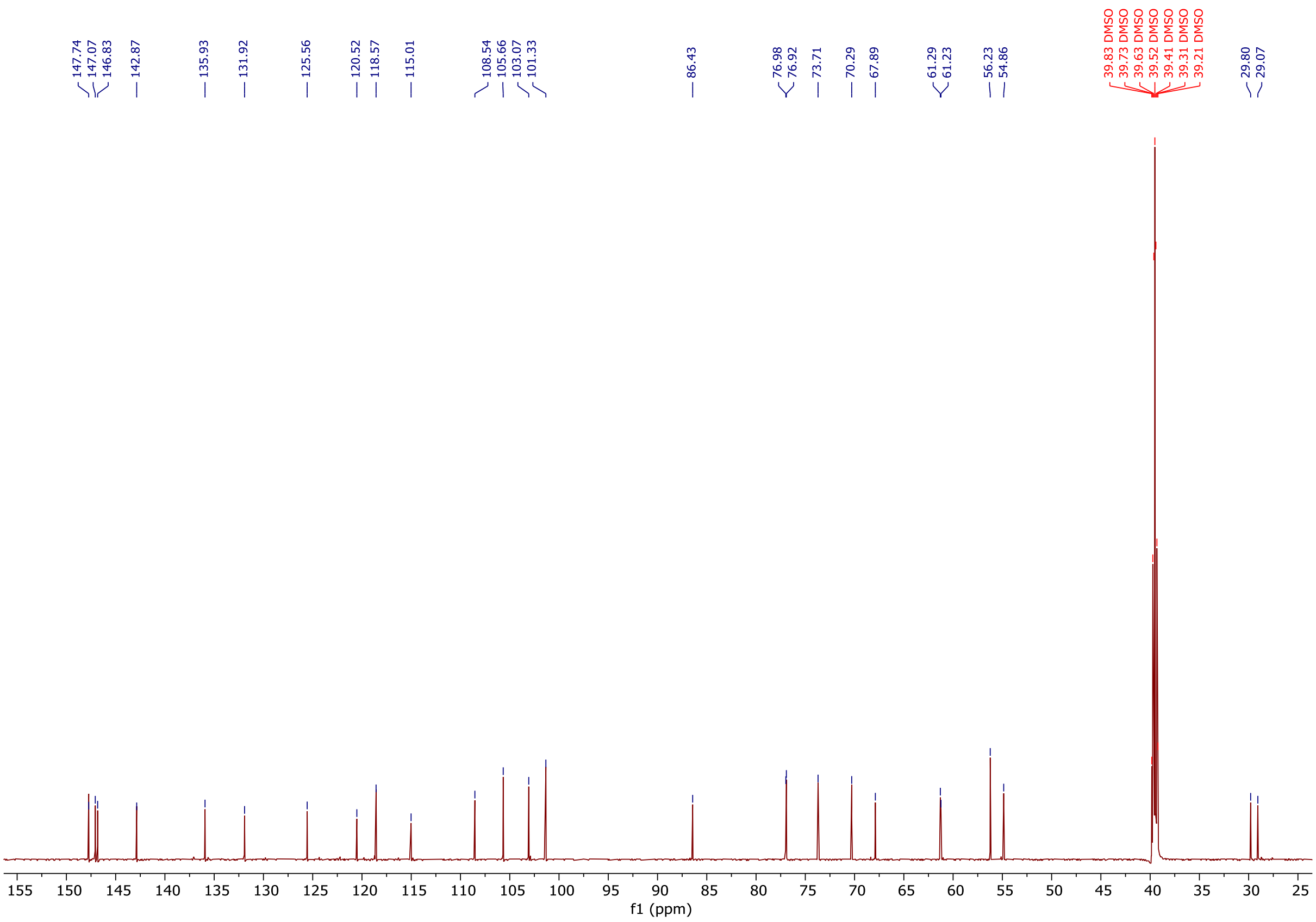

Figure S49 COSY spectrum of compound **12** in DMSO-d_6

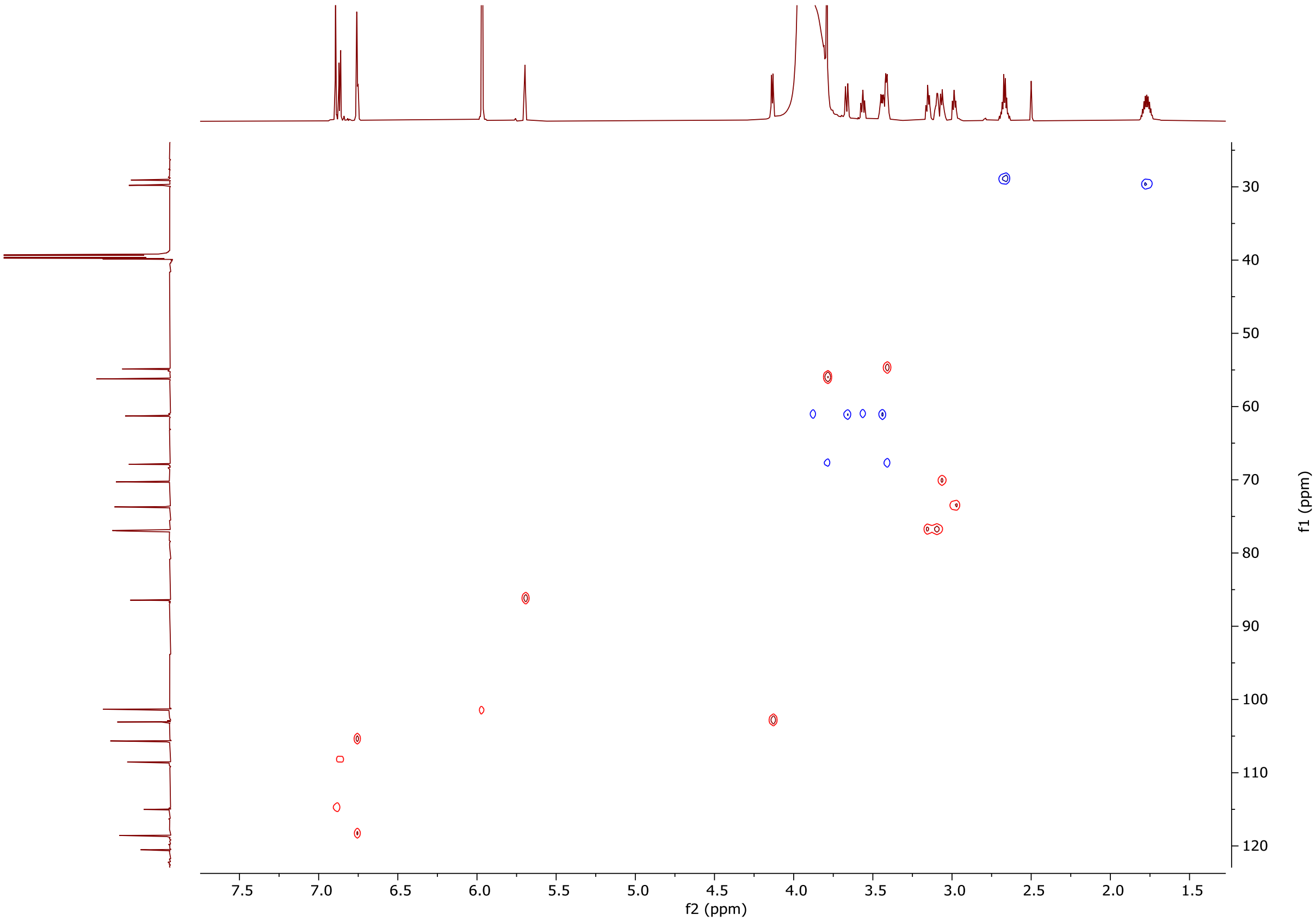

Figure S50 HSQC spectrum of compound **12** in DMSO-d_6

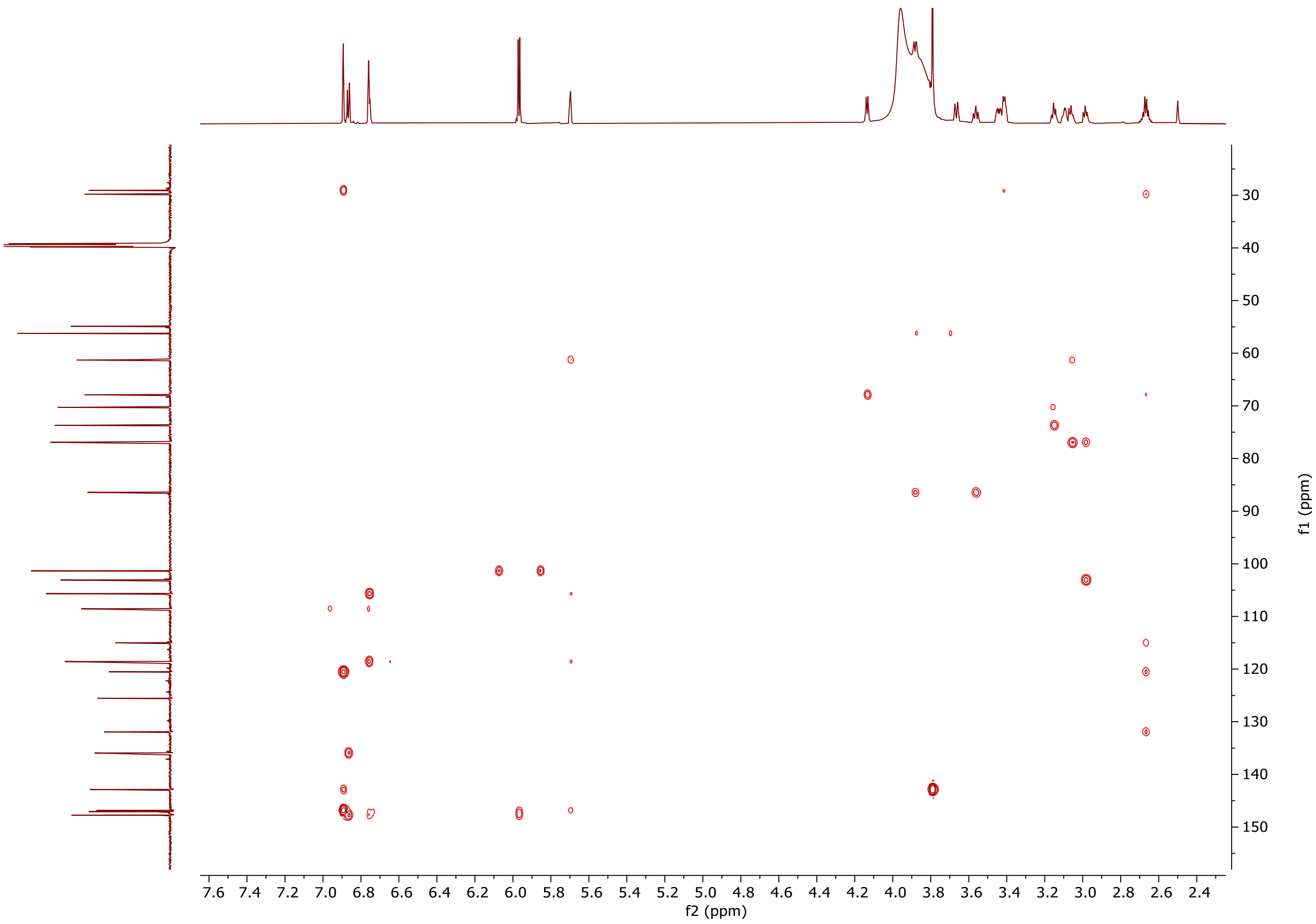

Figure S51 HMBC spectrum of compound **12** in DMSO-d_6

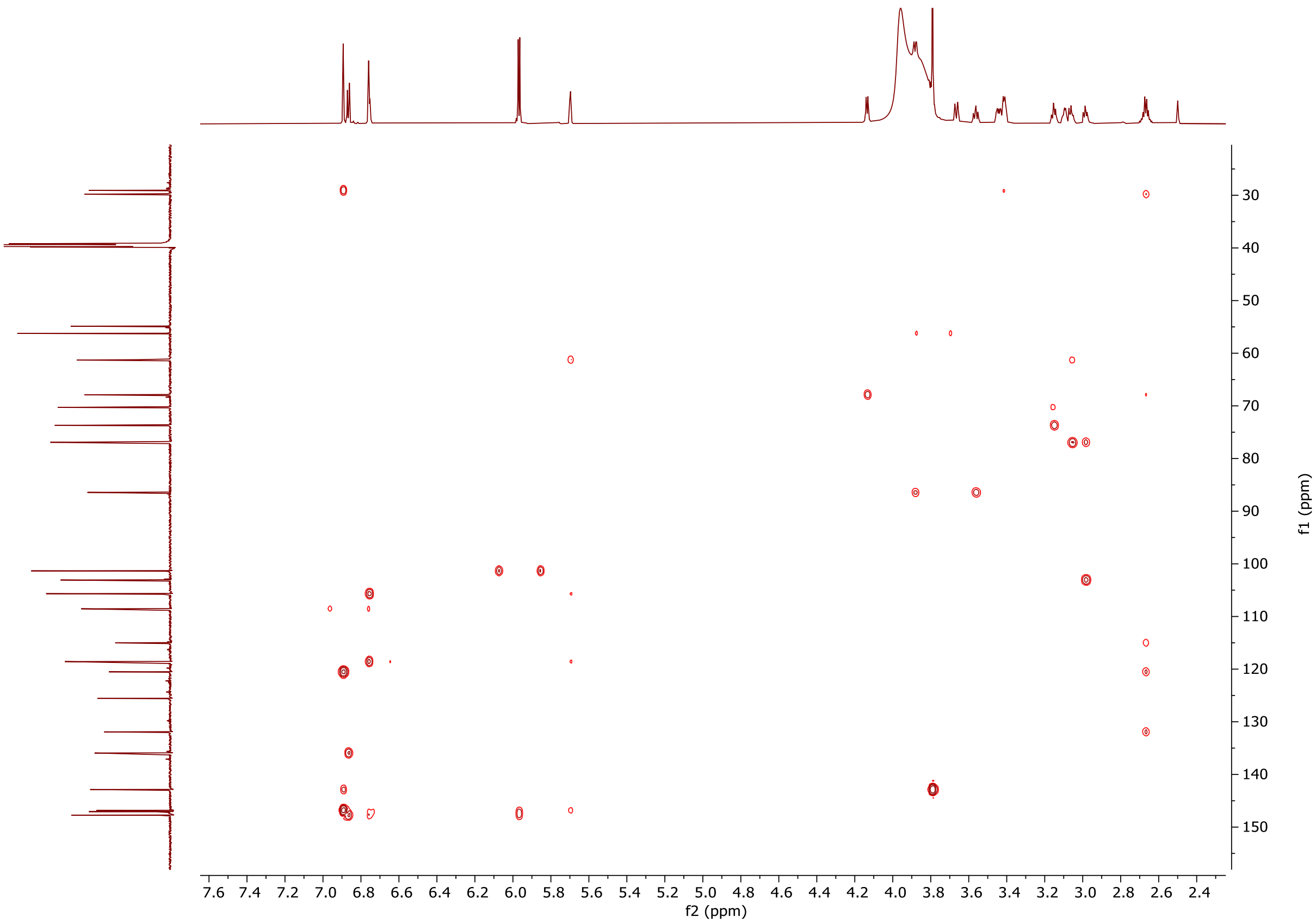

Figure S52 ROESY spectrum of compound **12** in DMSO-d_6

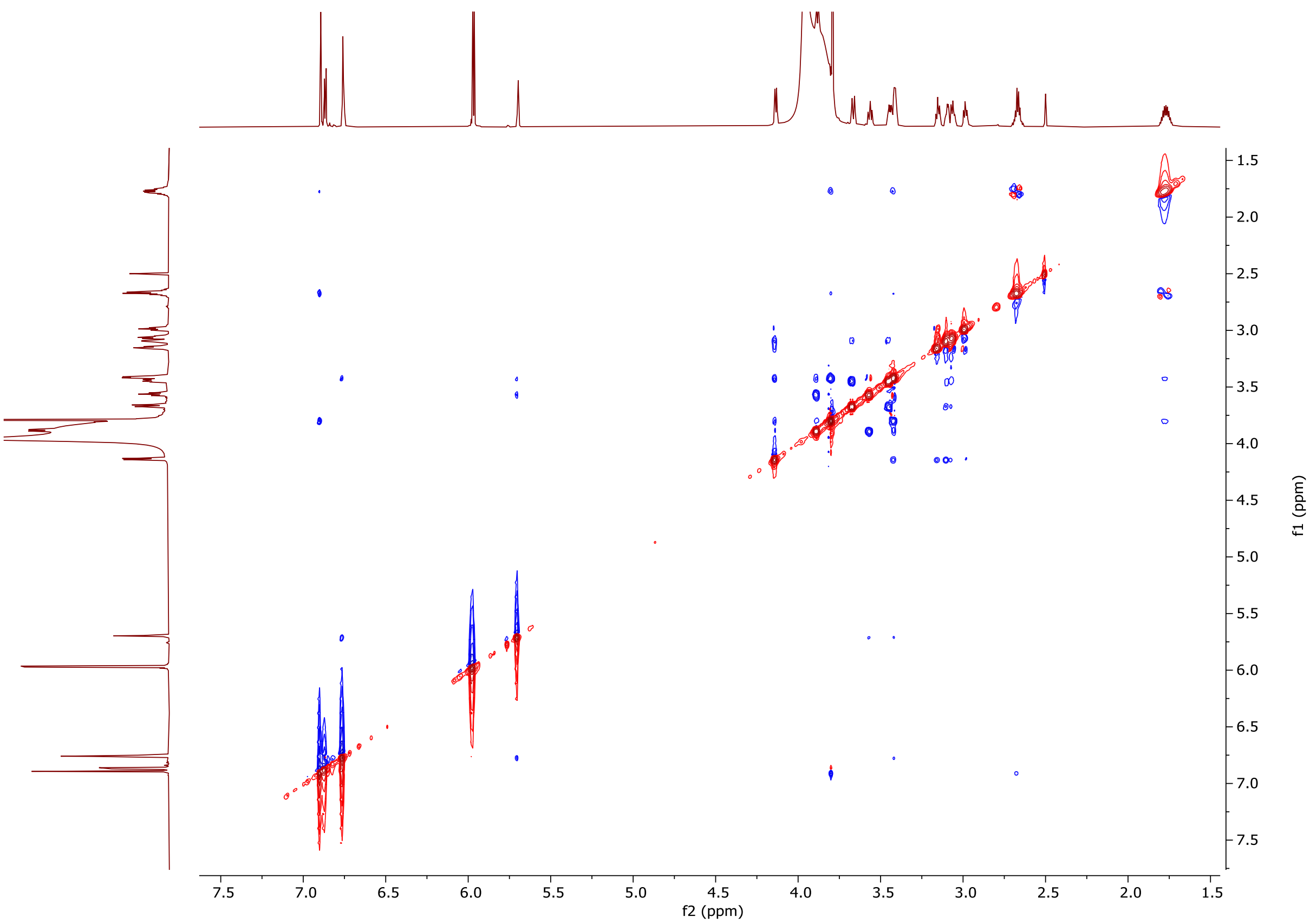

Figure S53 ^{1}H NMR (800 MHz) spectrum of compound **13** in DMSO-d_6

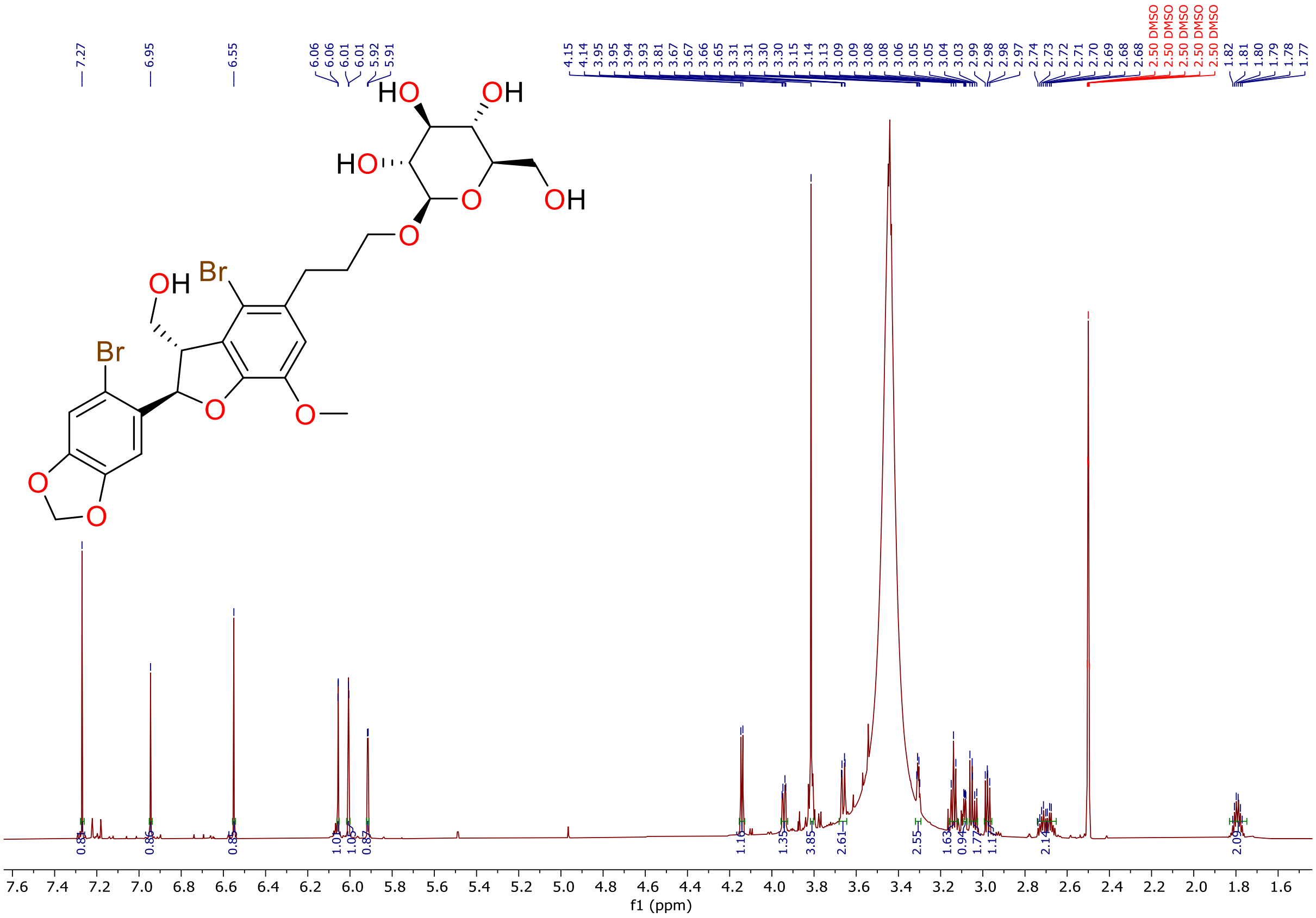

Figure S54 ^{13}C NMR (200 MHz) spectrum of compound **13** in DMSO-d_6

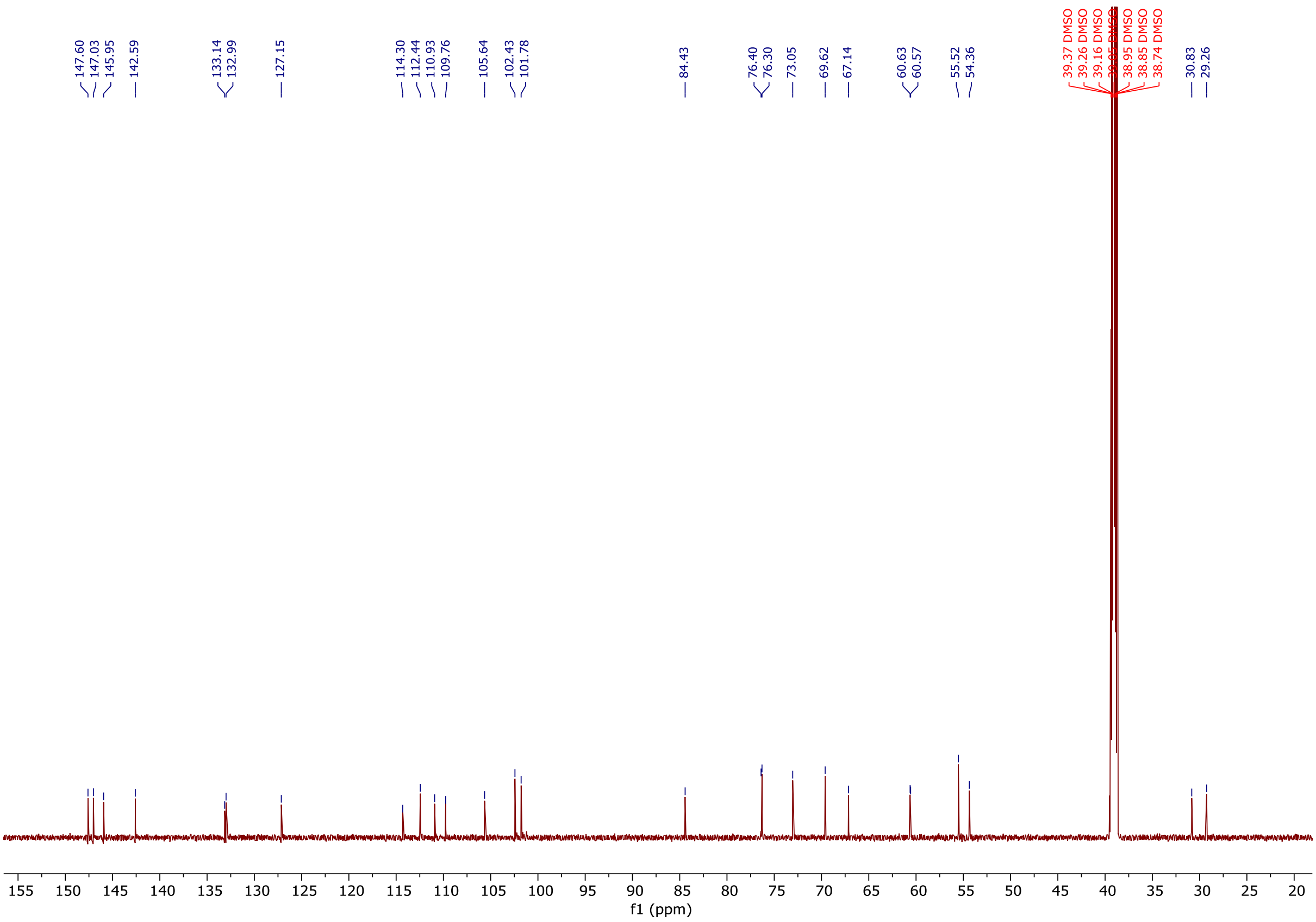

Figure S55 COSY spectrum of compound **13** in DMSO-d_6

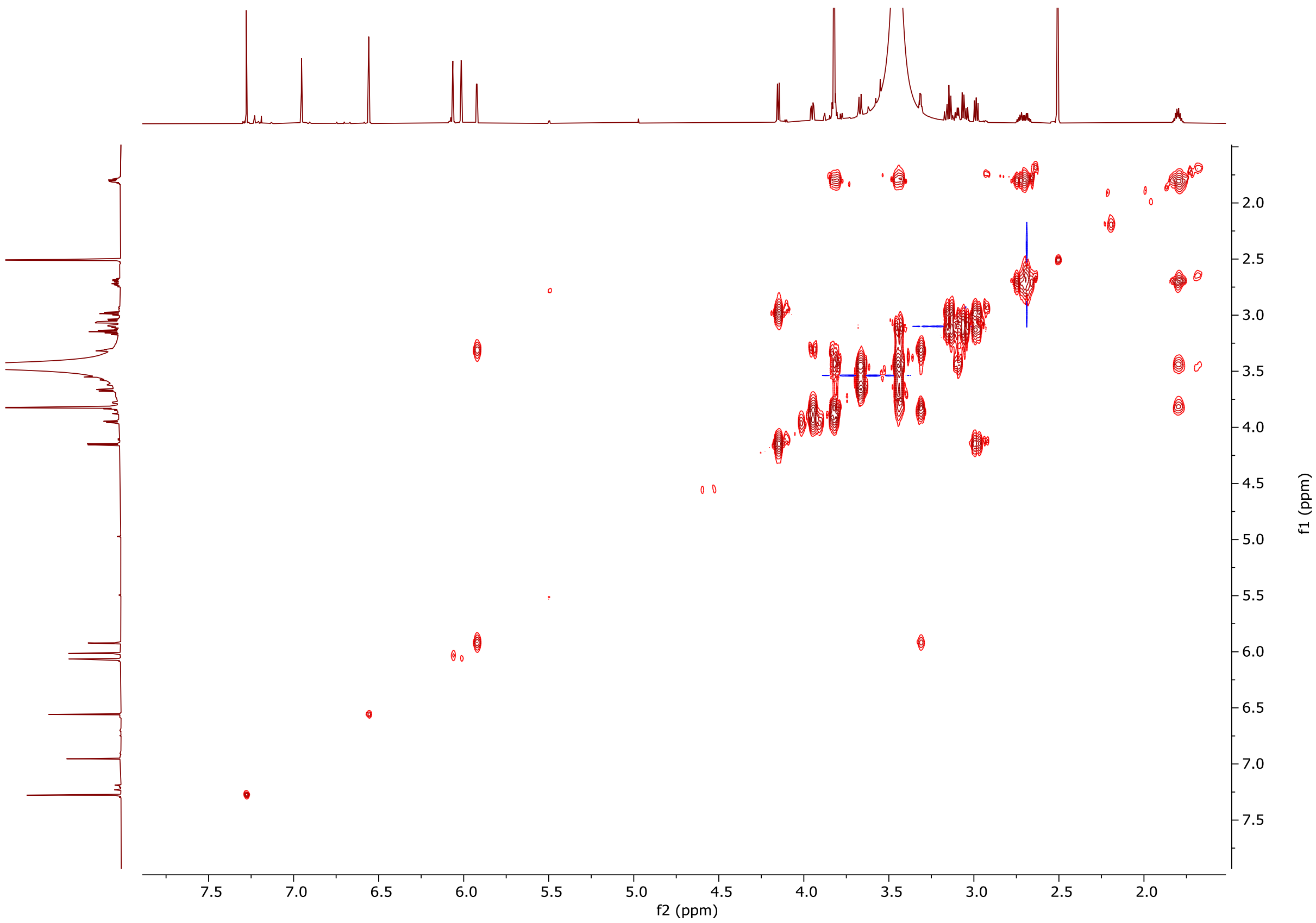

Figure S56 HSQC spectrum of compound **13** in DMSO-d_6

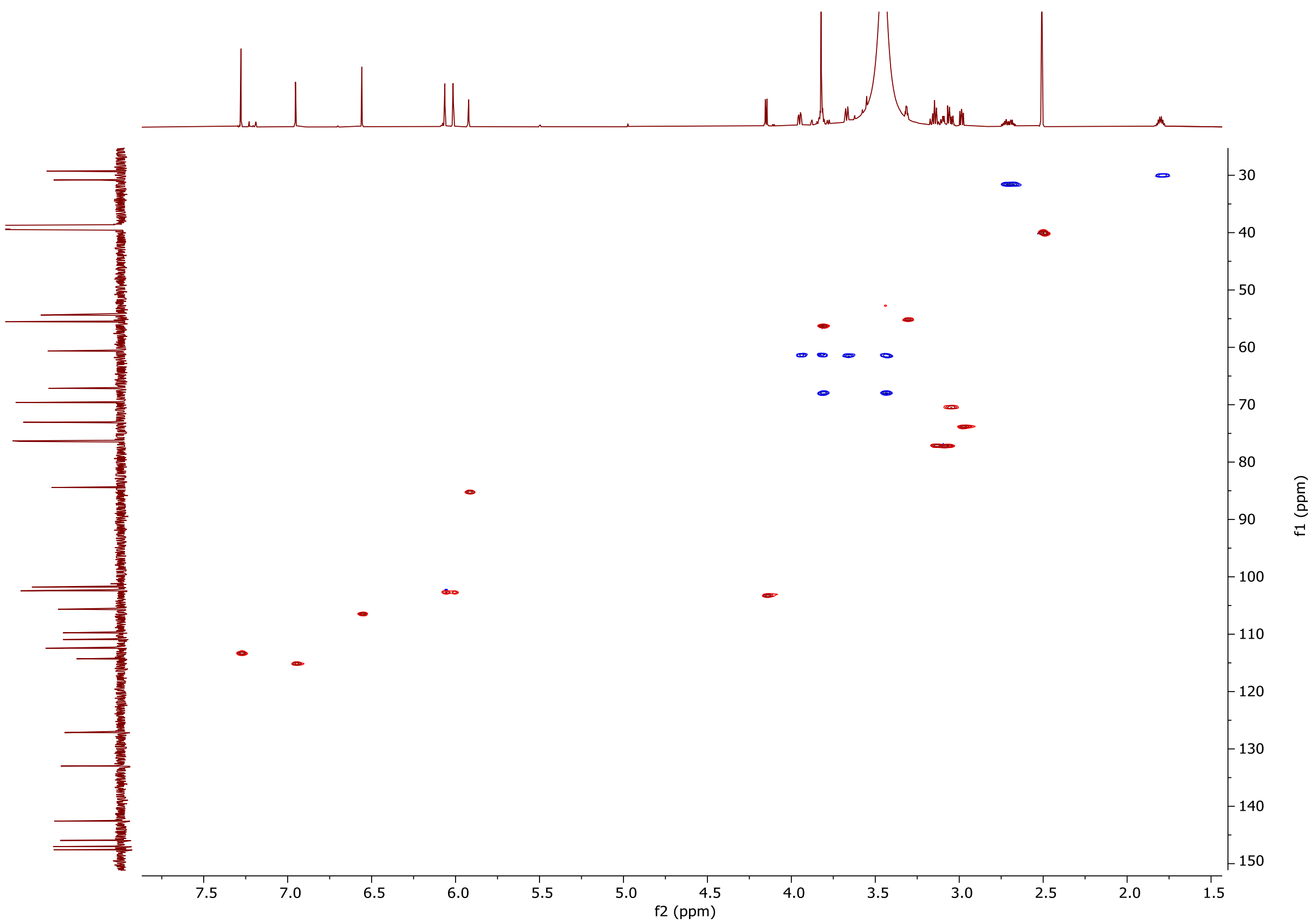

Figure S57 HMBC spectrum of compound **13** in DMSO-d_6

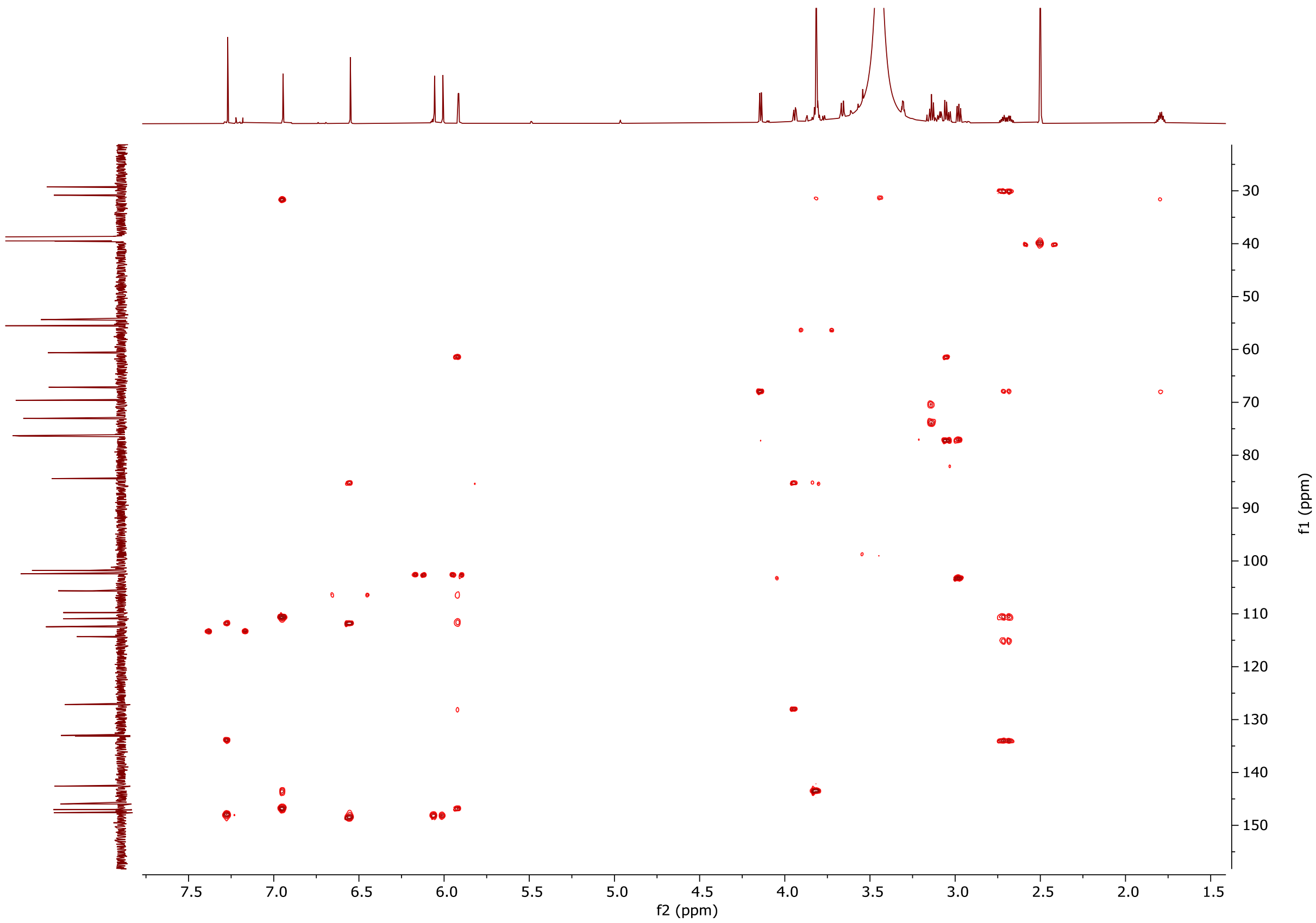

Figure S58 ROESY spectrum of compound **13** in DMSO-d_6

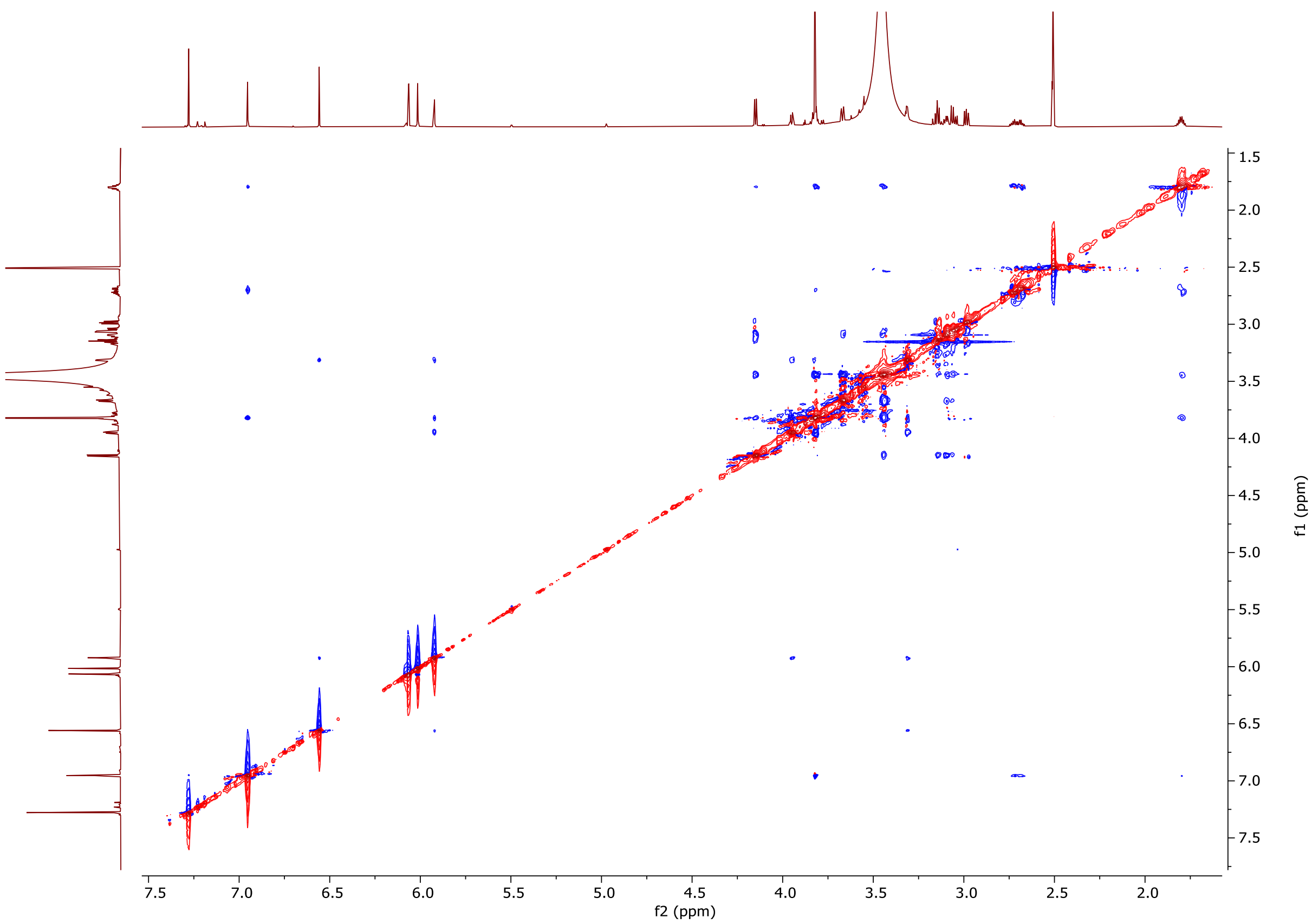

Figure S59 ^{1}H NMR (800 MHz) spectrum of compound **14** in DMSO-d_6

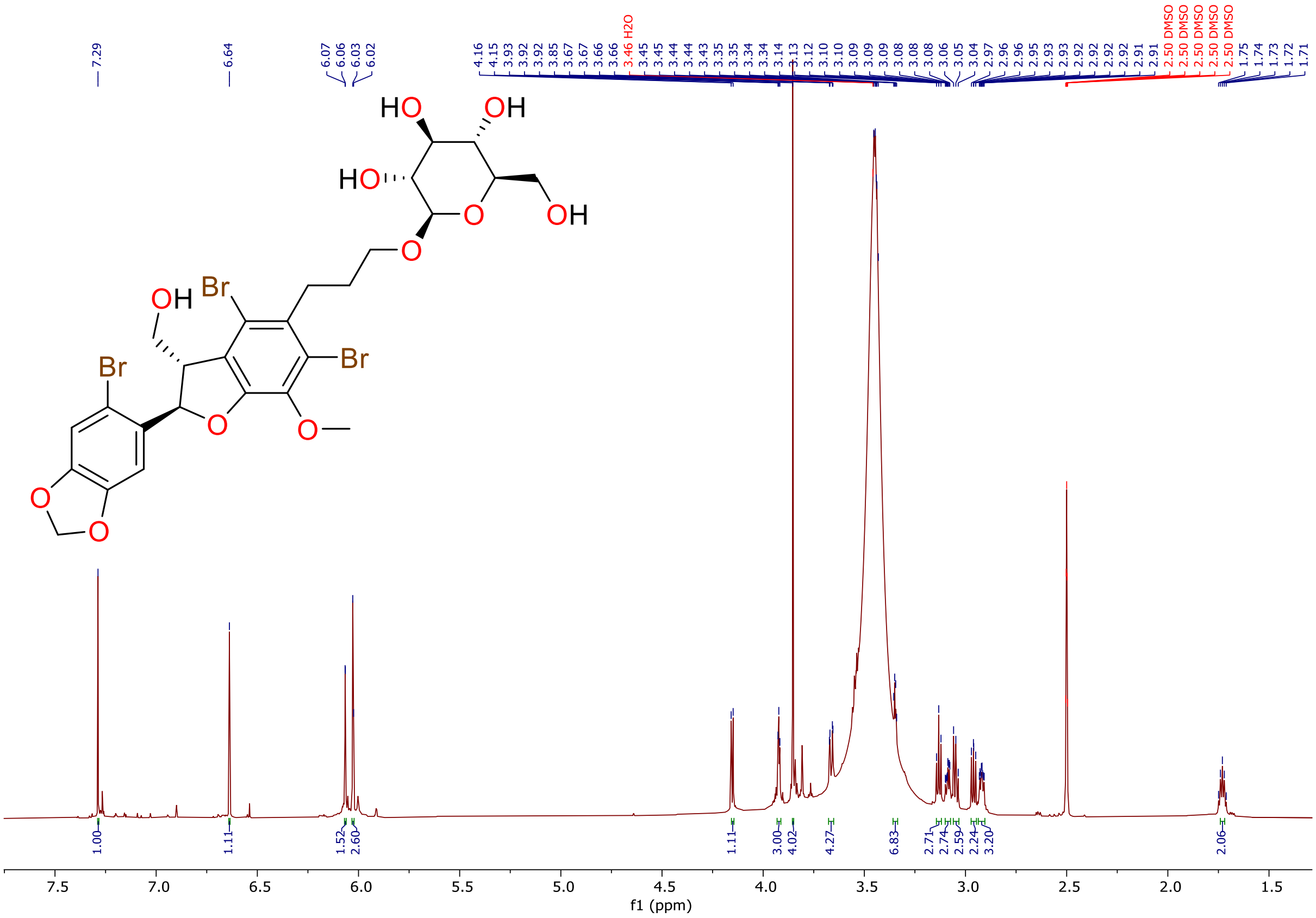

Figure S60 ^{13}C NMR (200 MHz) spectrum of compound **14** in DMSO-d_6

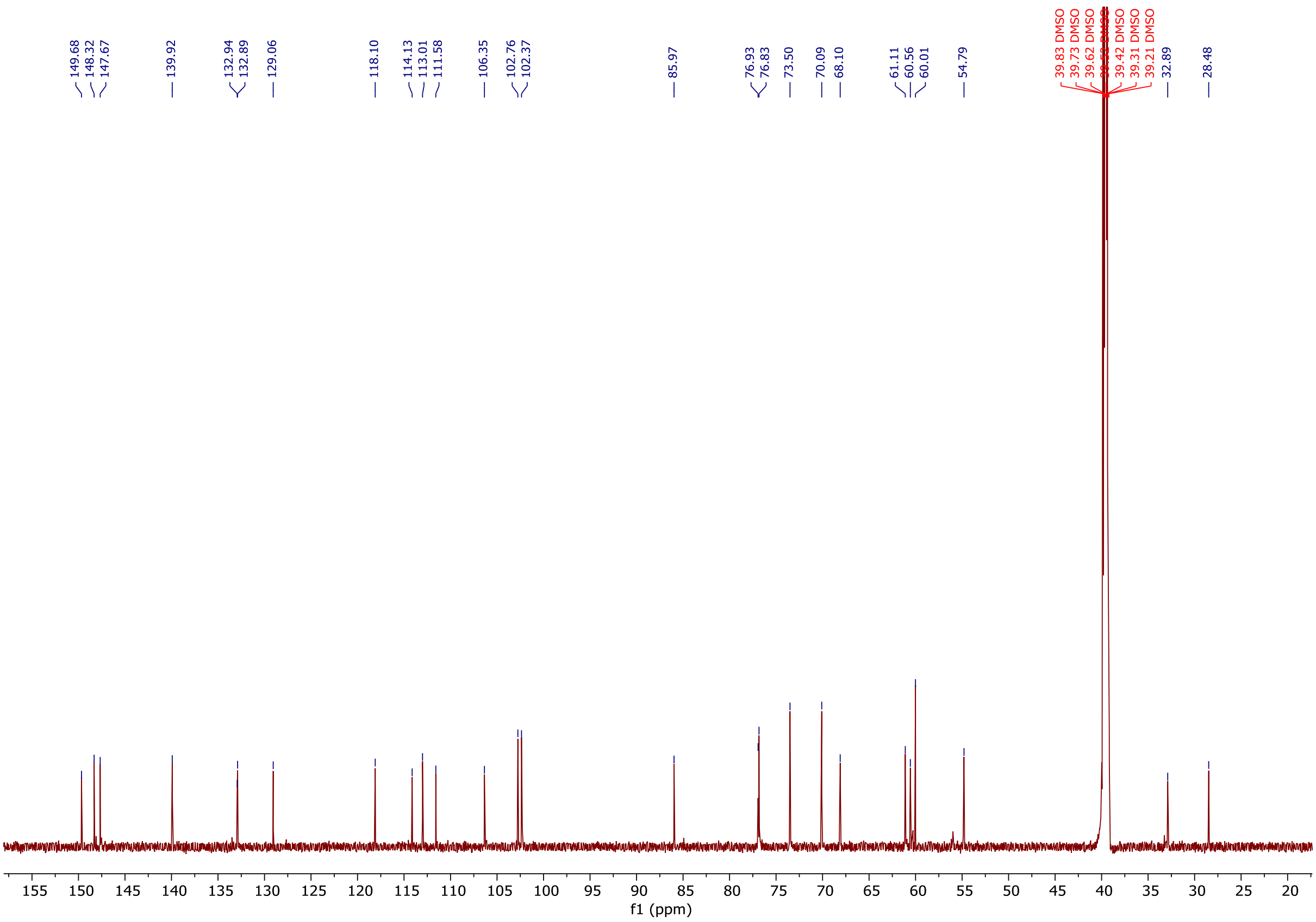

Figure S61 COSY spectrum of compound **14** in DMSO-d_6

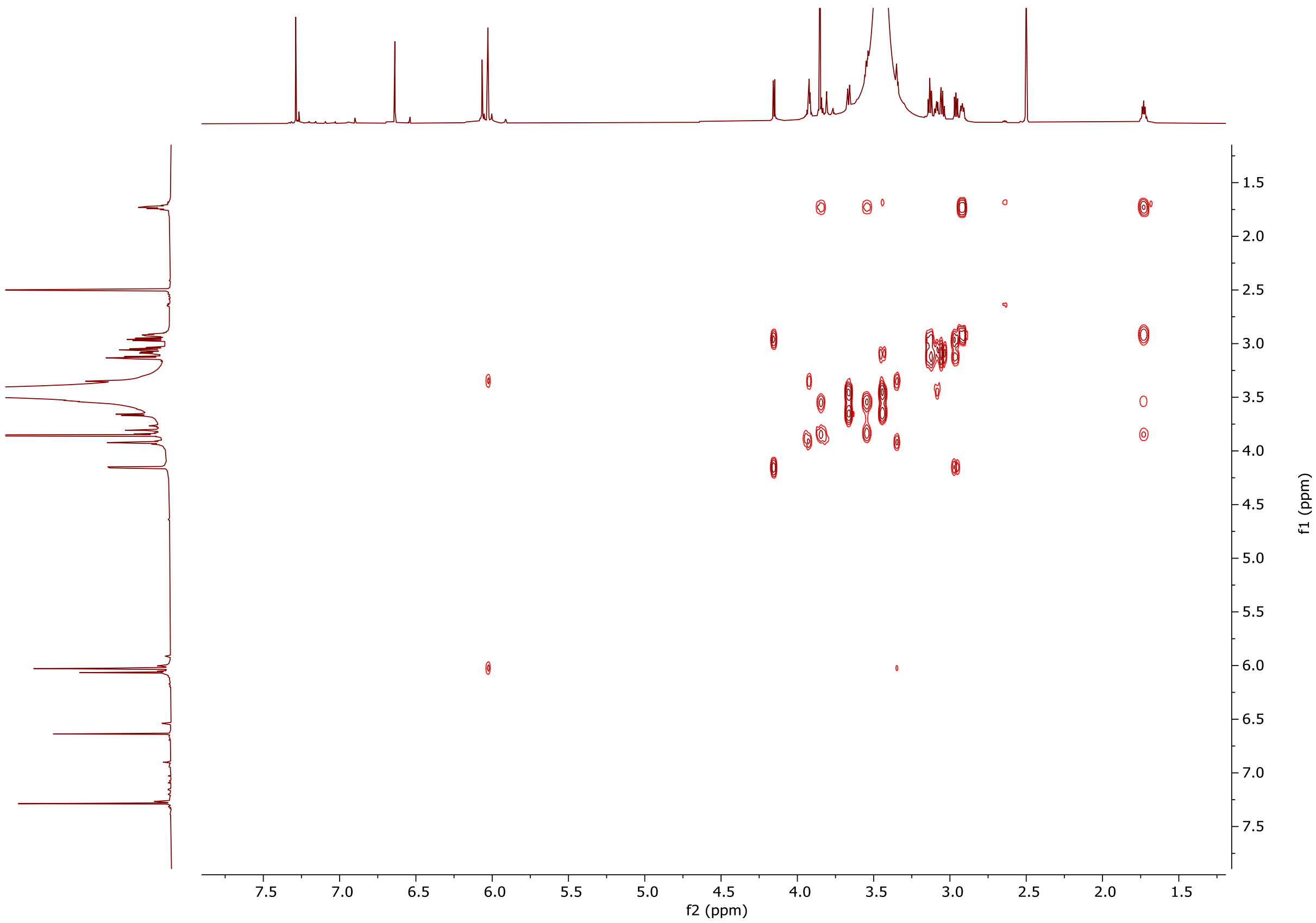

Figure S62 HSQC spectrum of compound **14** in DMSO-d_6

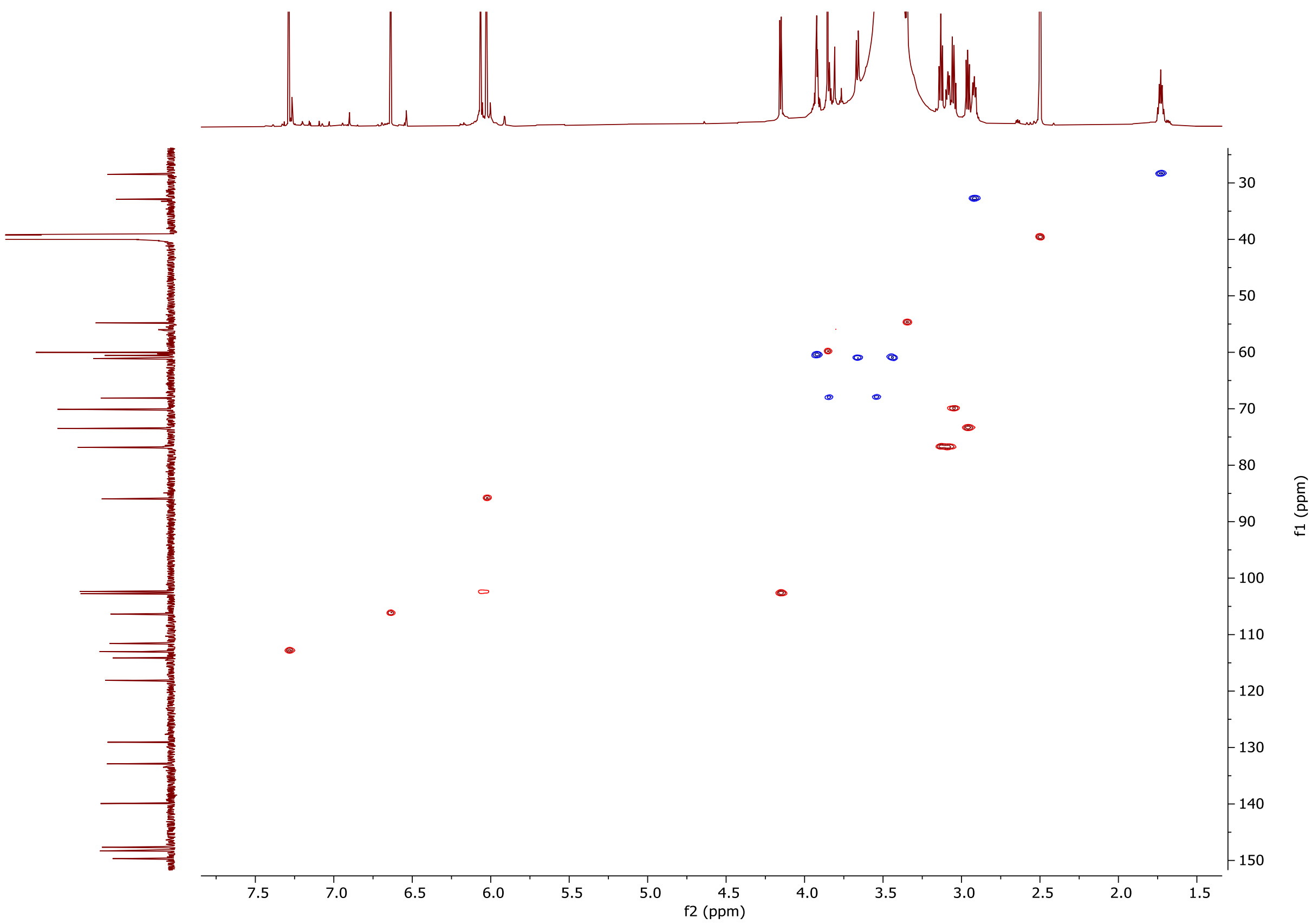

Figure S63 HMBC spectrum of compound **14** in DMSO-d_6

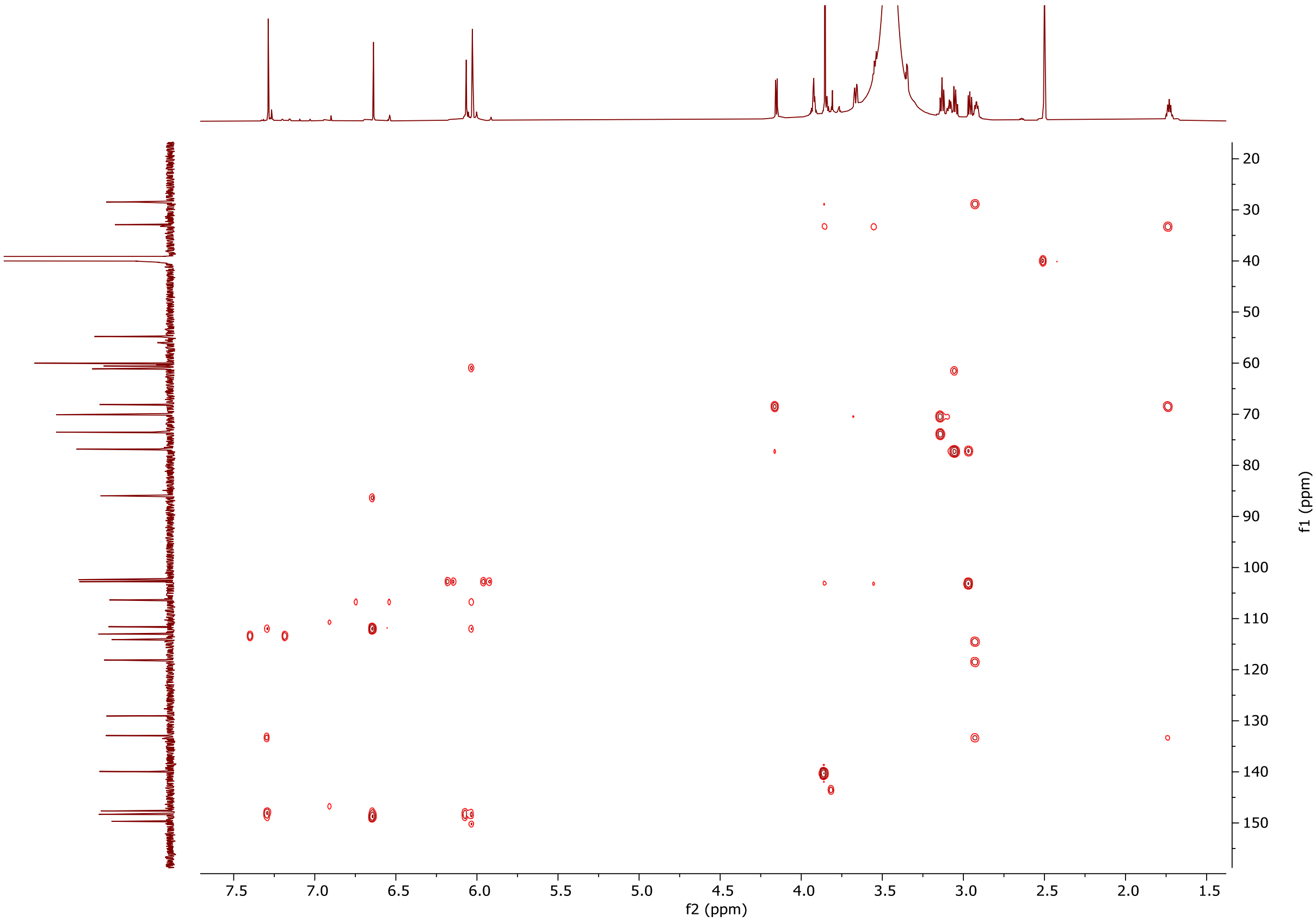

Figure S64 ROESY spectrum of compound **14** in DMSO-d_6

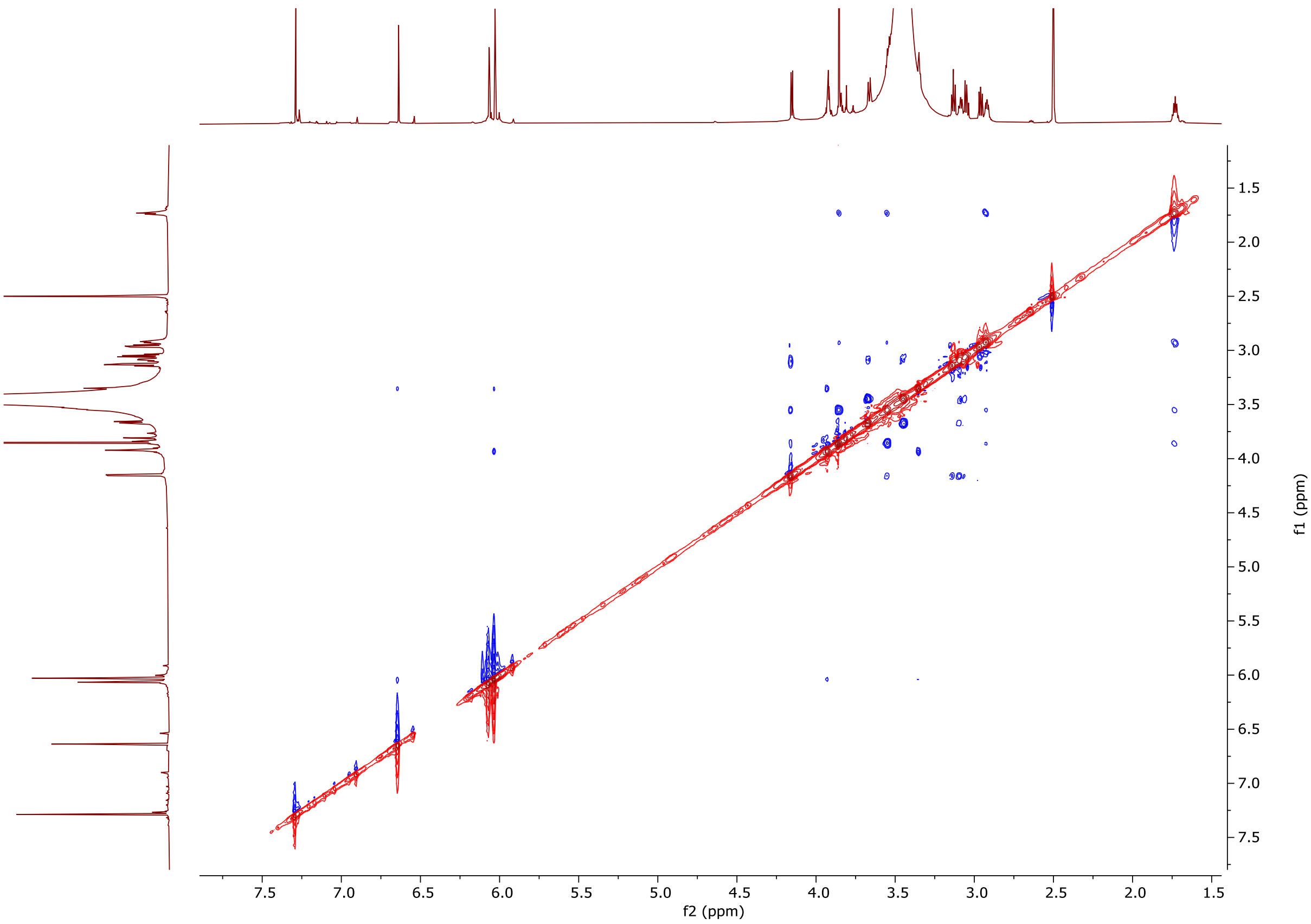

Figure S65 ^{1}H NMR (800 MHz) spectrum of compound **15** in DMSO-d_6

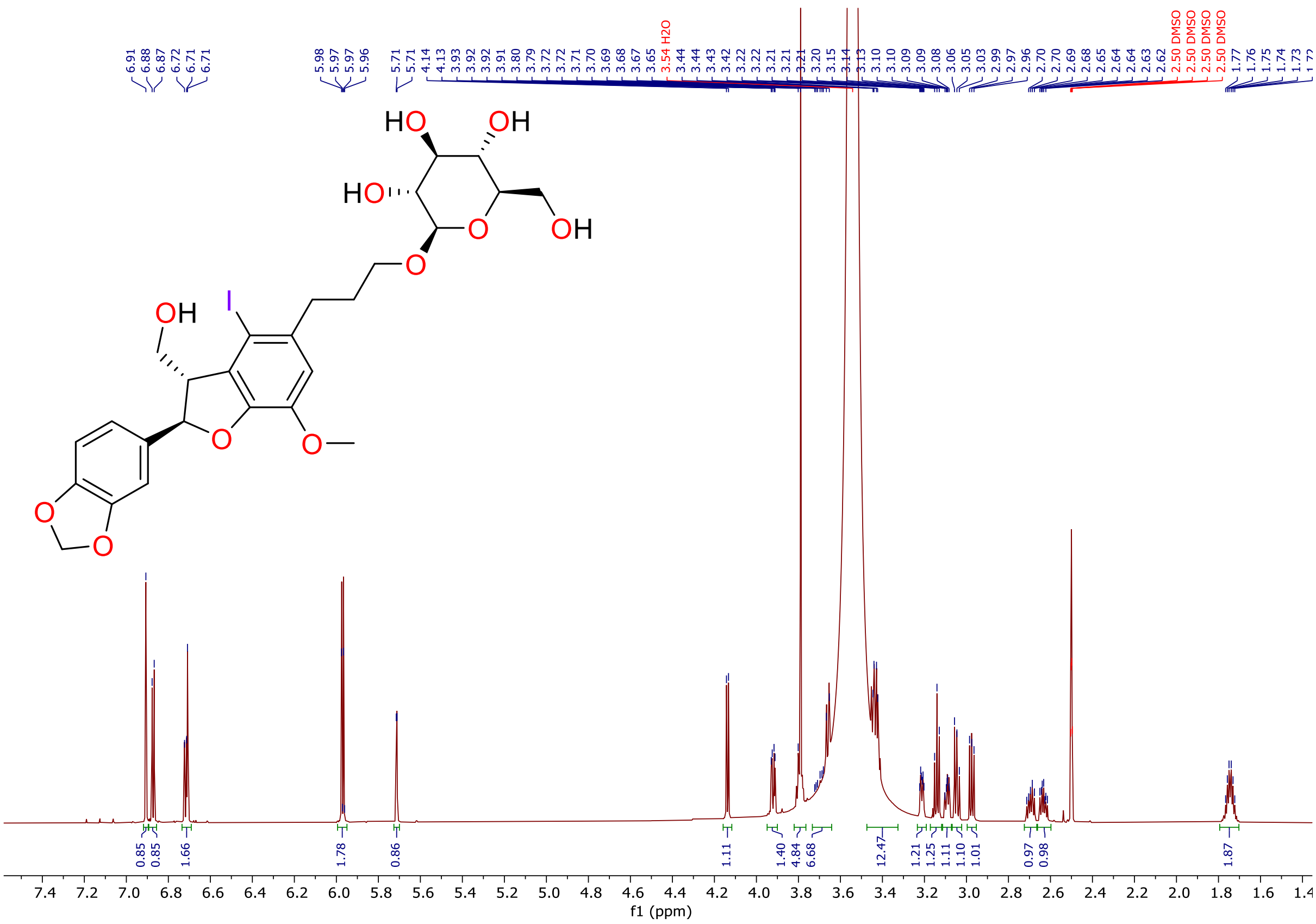

Figure S66 ^{13}C NMR (200 MHz) spectrum of compound **15** in DMSO-d_6

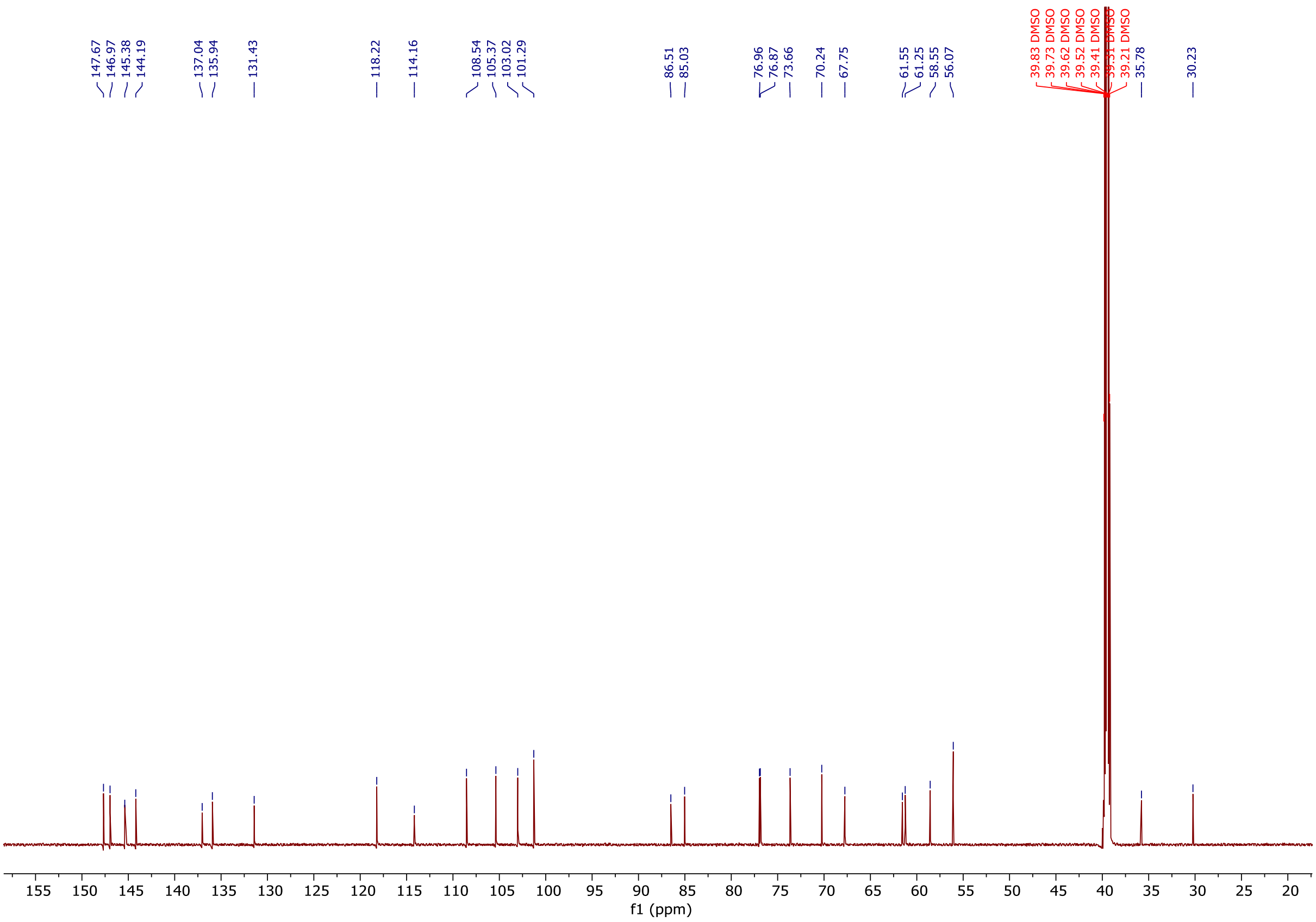

Figure S67 COSY spectrum of compound **15** in DMSO-d_6

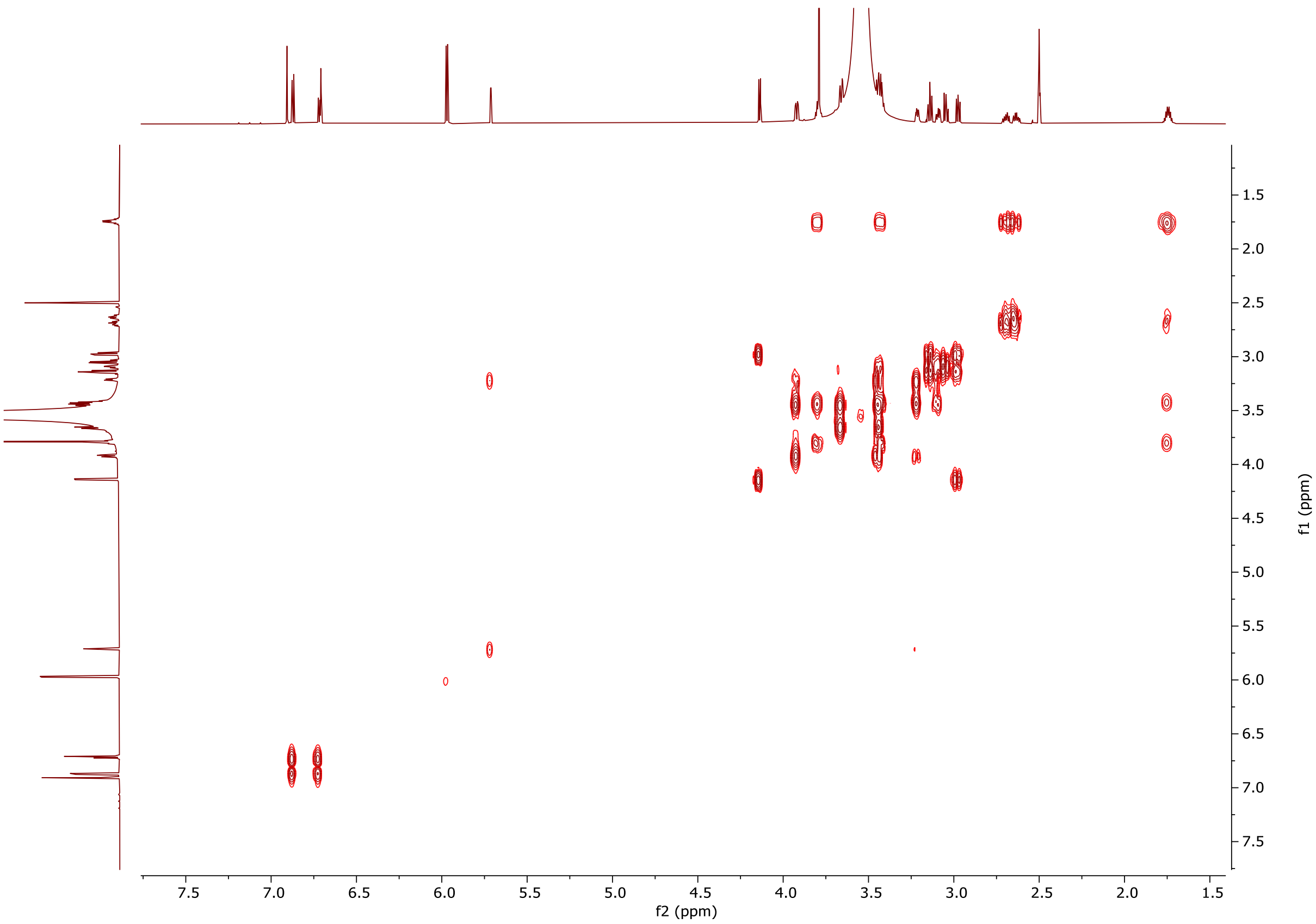

Figure S68 HSQC spectrum of compound **15** in DMSO-d_6

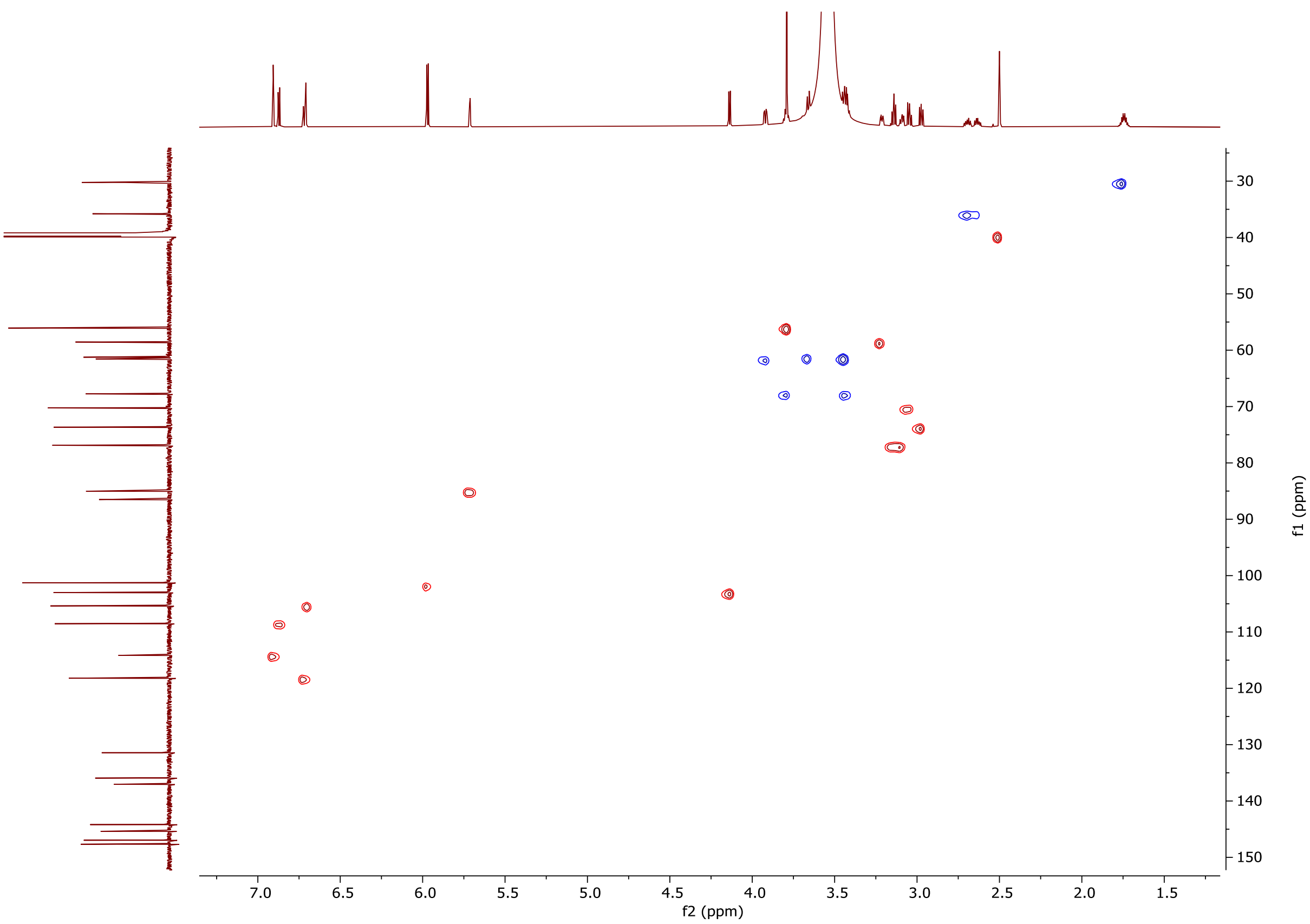

Figure S69 HMBC spectrum of compound **15** in DMSO-d_6

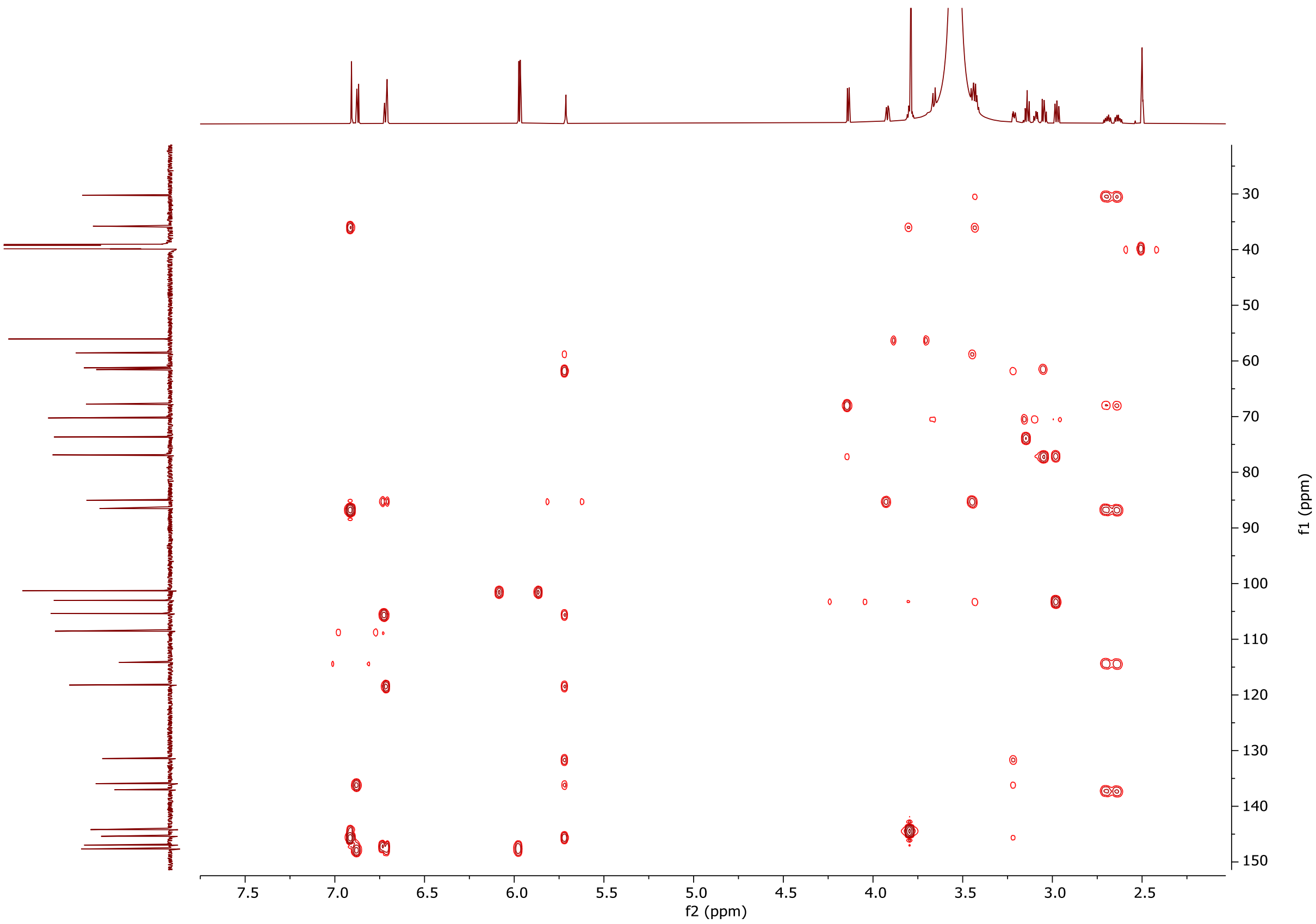

Figure S70 ROESY spectrum of compound **15** in DMSO-d_6

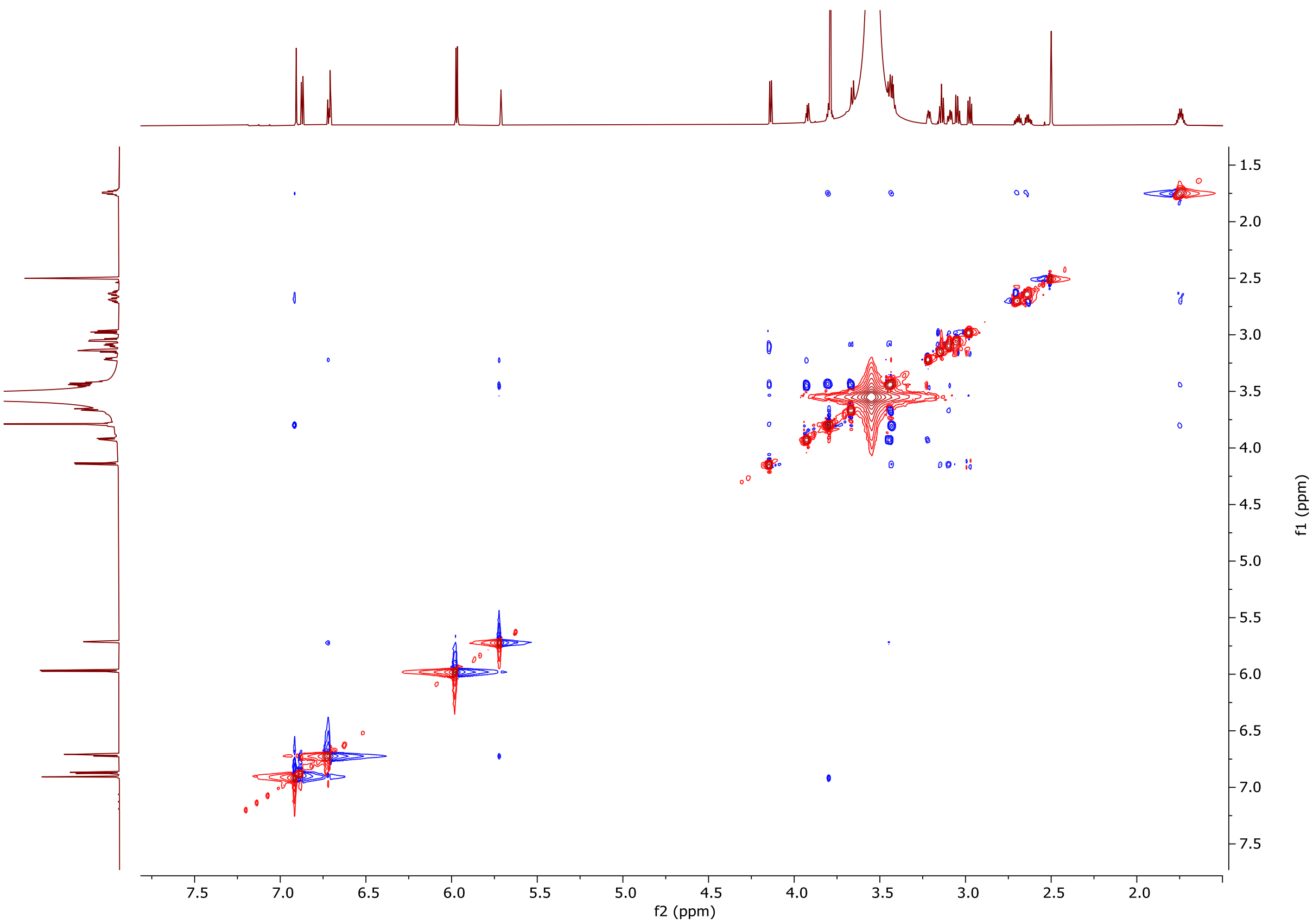

Figure S71 ^{1}H NMR (800 MHz) spectrum of compound **16** in DMSO-d_6

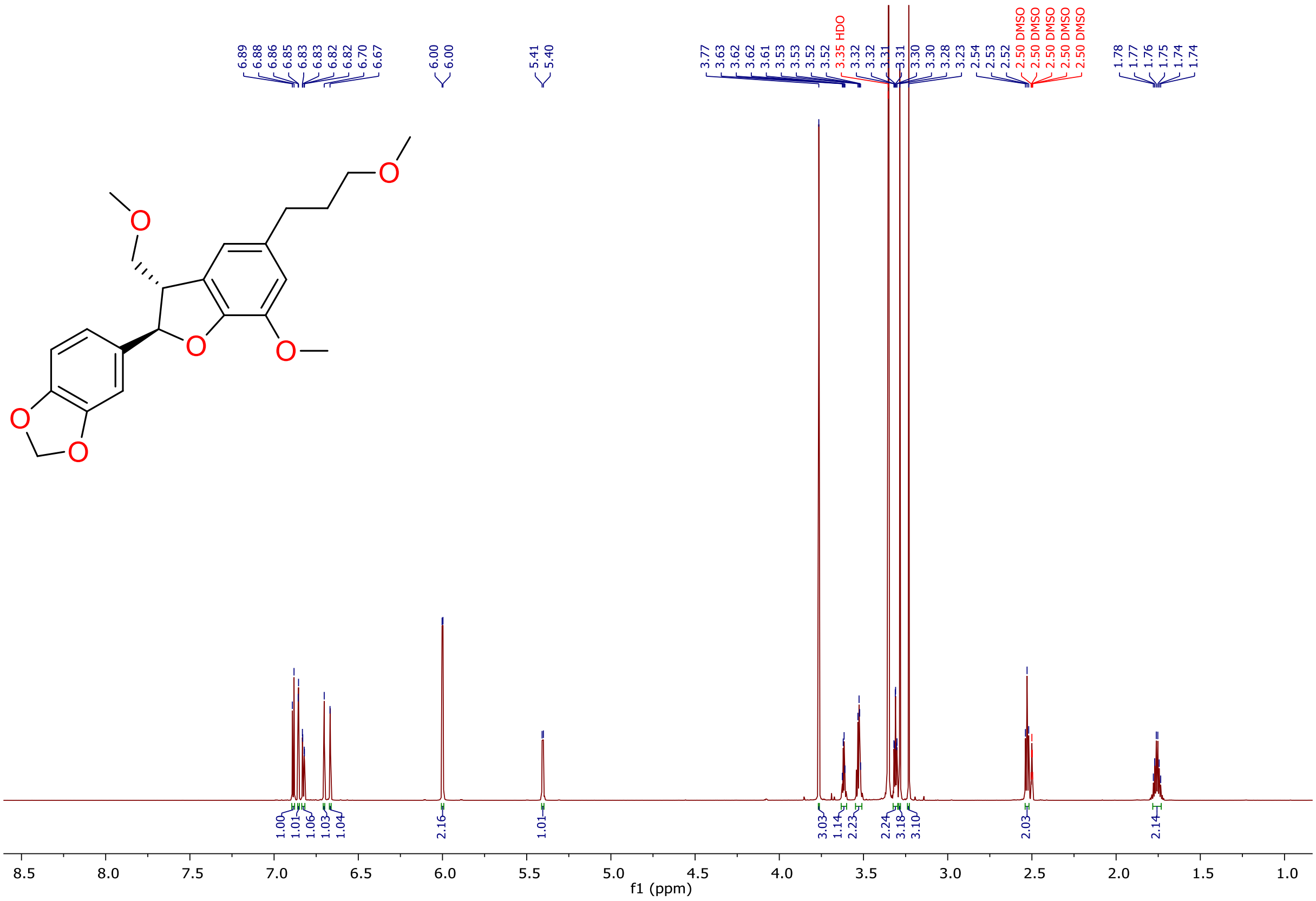

Figure S72 ^{13}C NMR (200 MHz) spectrum of compound **16** in DMSO-d_6

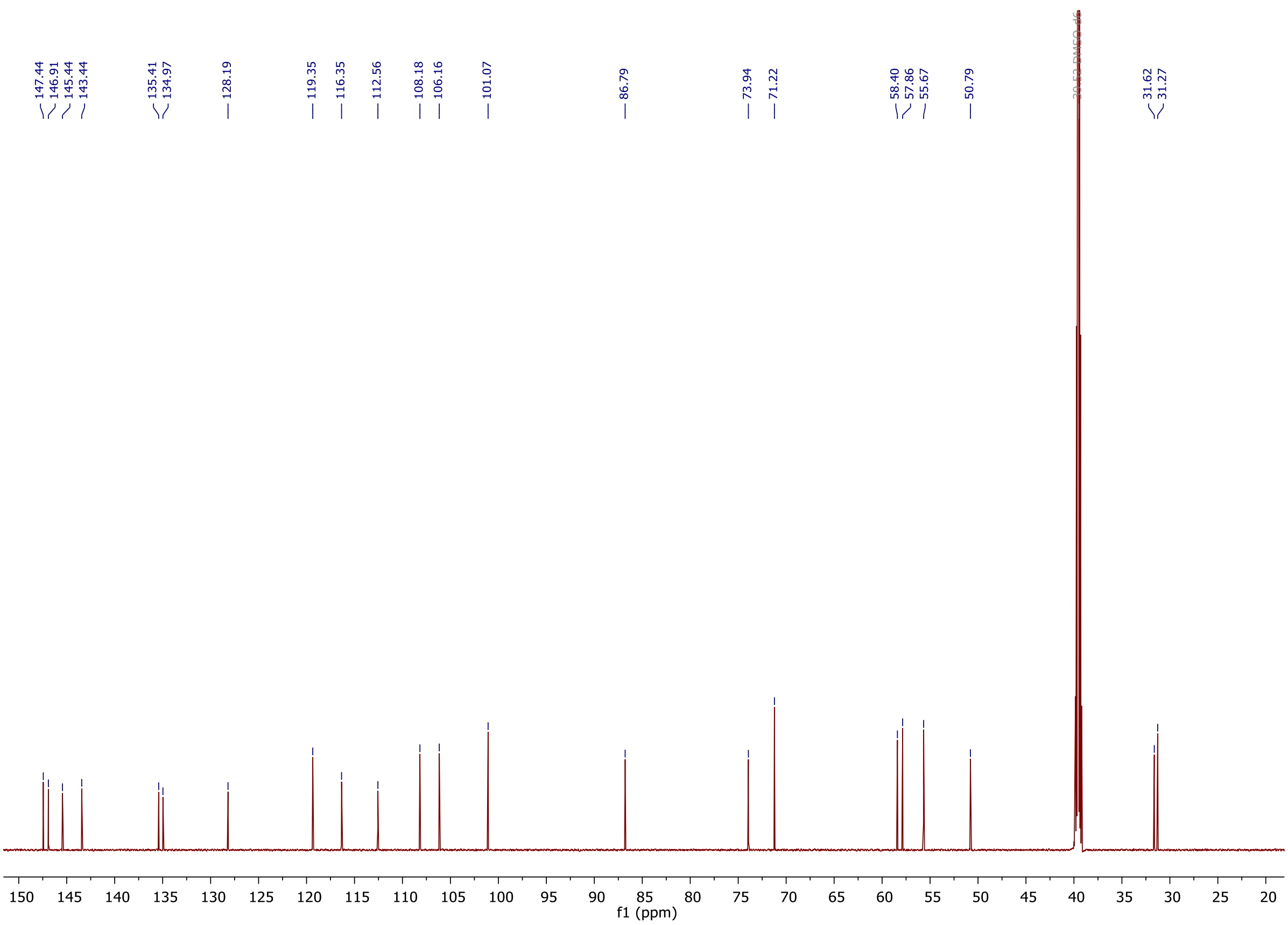

Figure S73 ^{1}H NMR (800 MHz) spectrum of compound **17** in DMSO-d_6

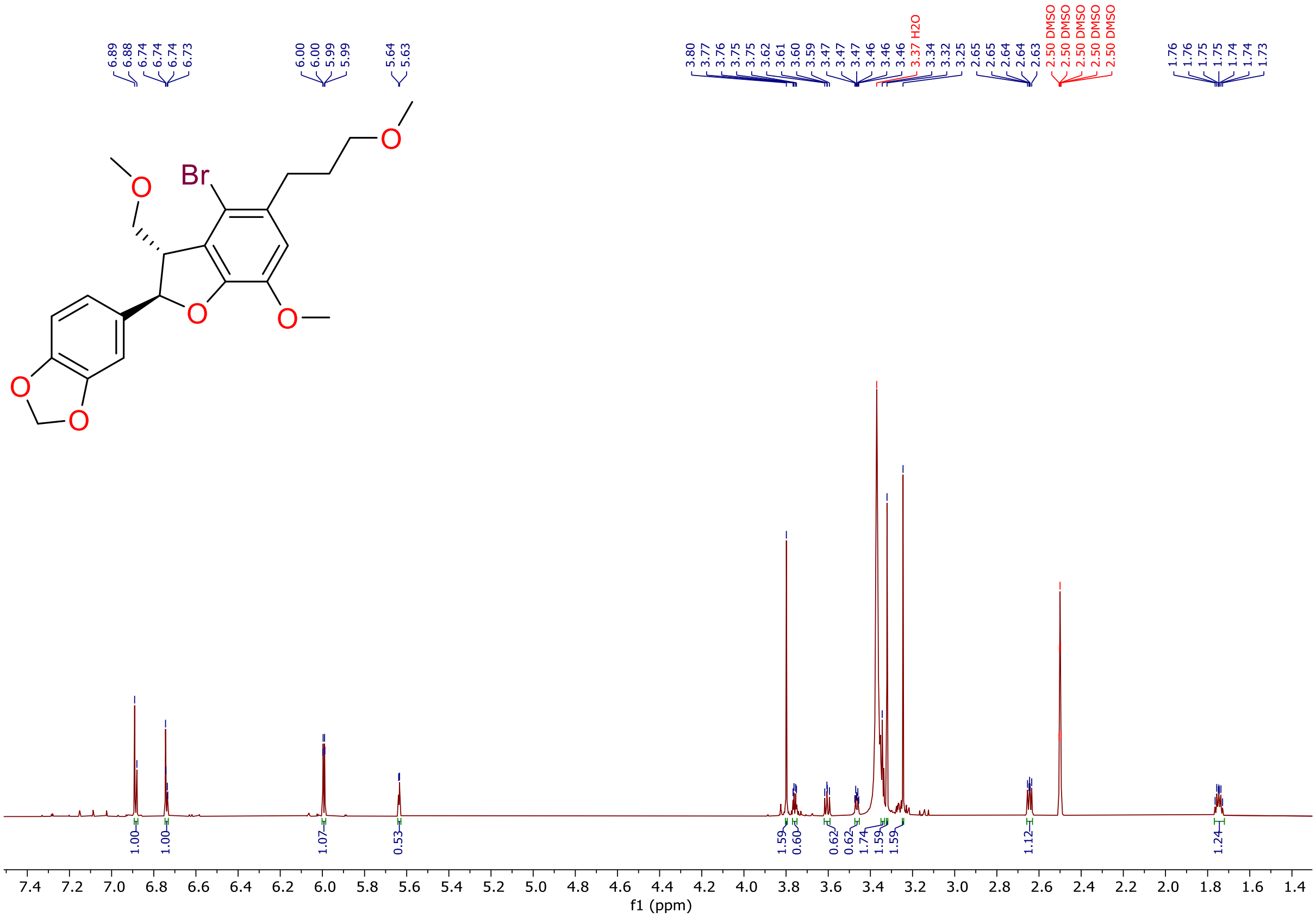

Figure S74 ^{13}C NMR (200 MHz) spectrum of compound **17** in DMSO-d_6

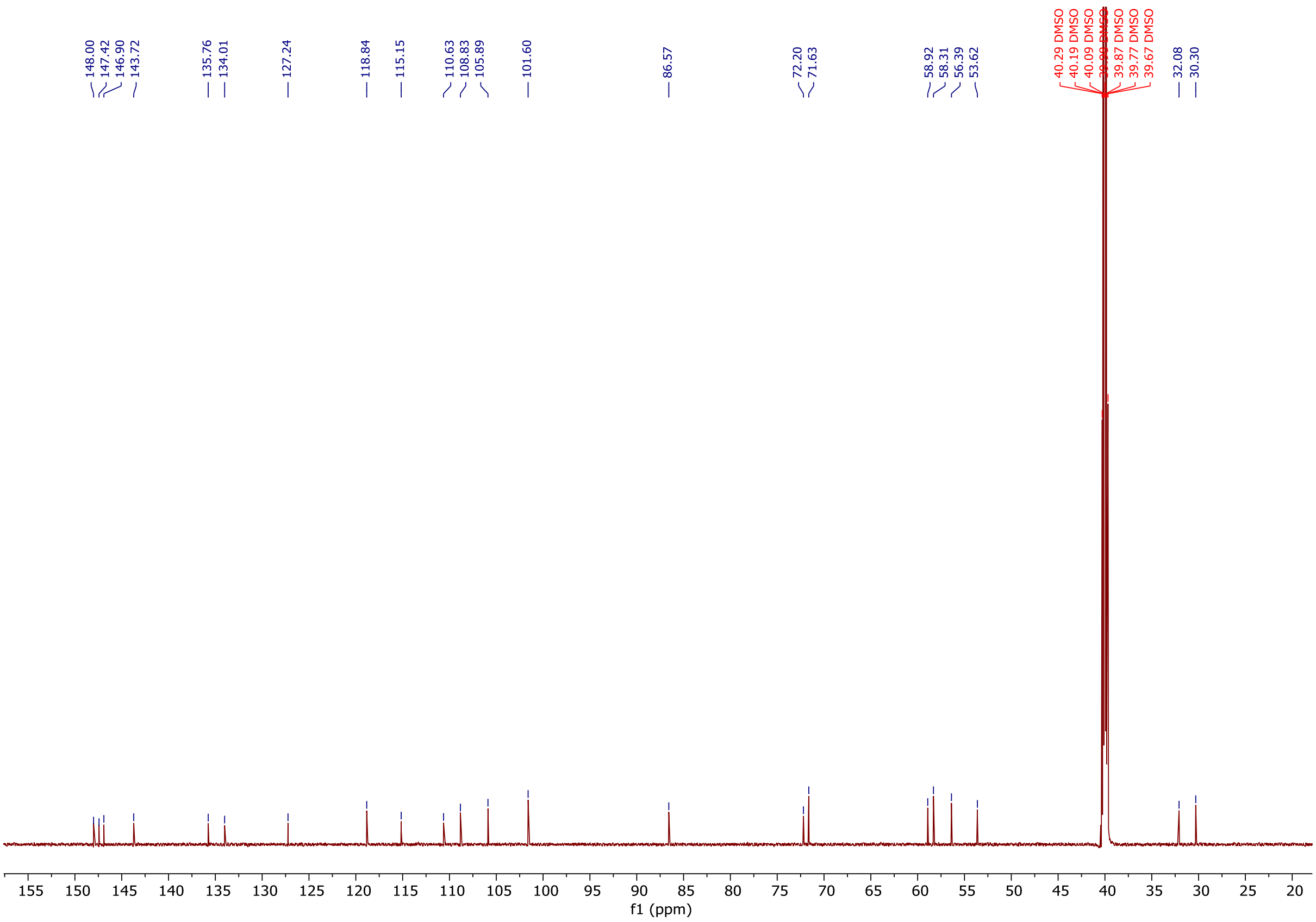

Figure S75 COSY spectrum of compound **17** in DMSO-d_6

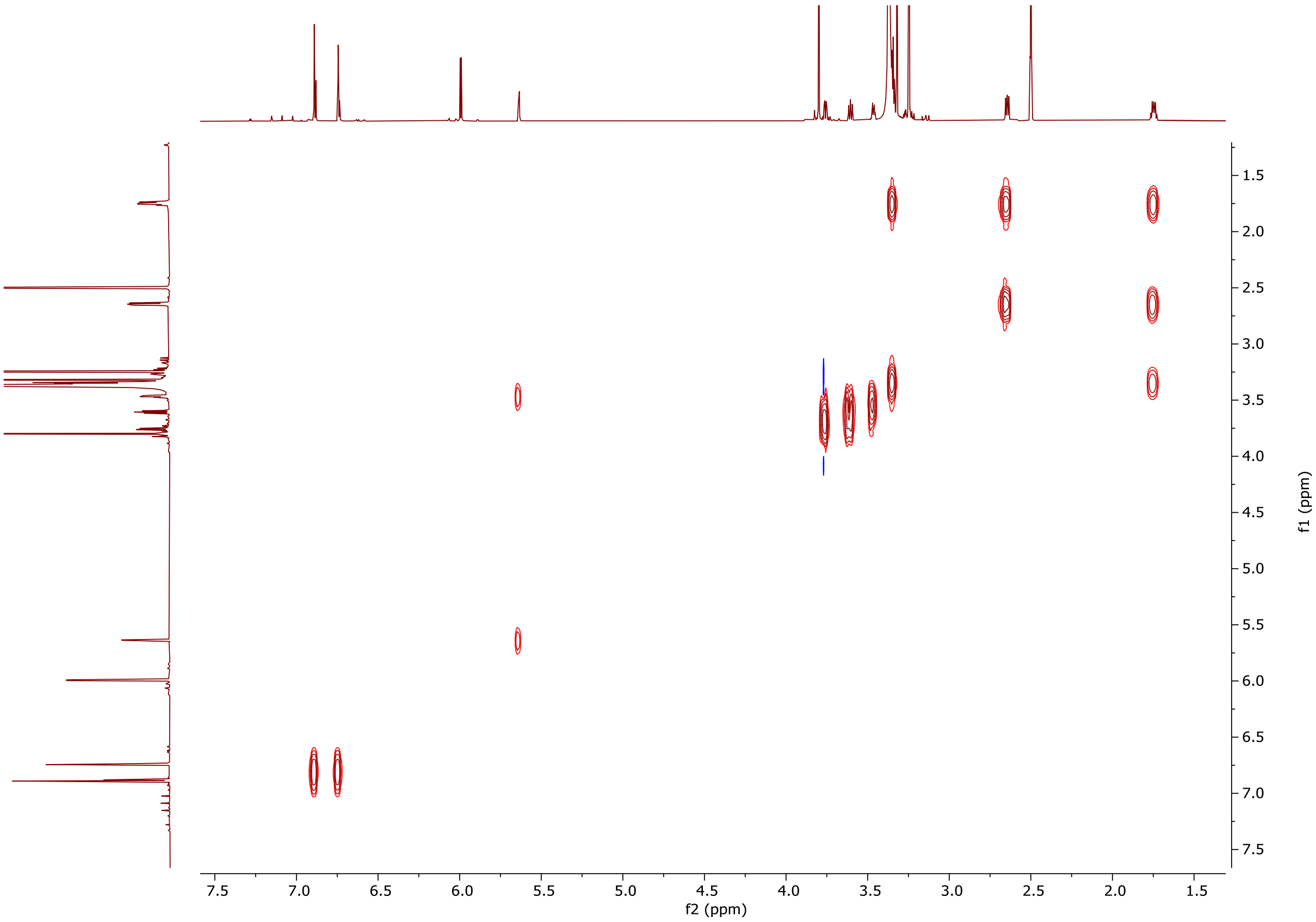

Figure S76 HSQC spectrum of compound **17** in DMSO-d_6

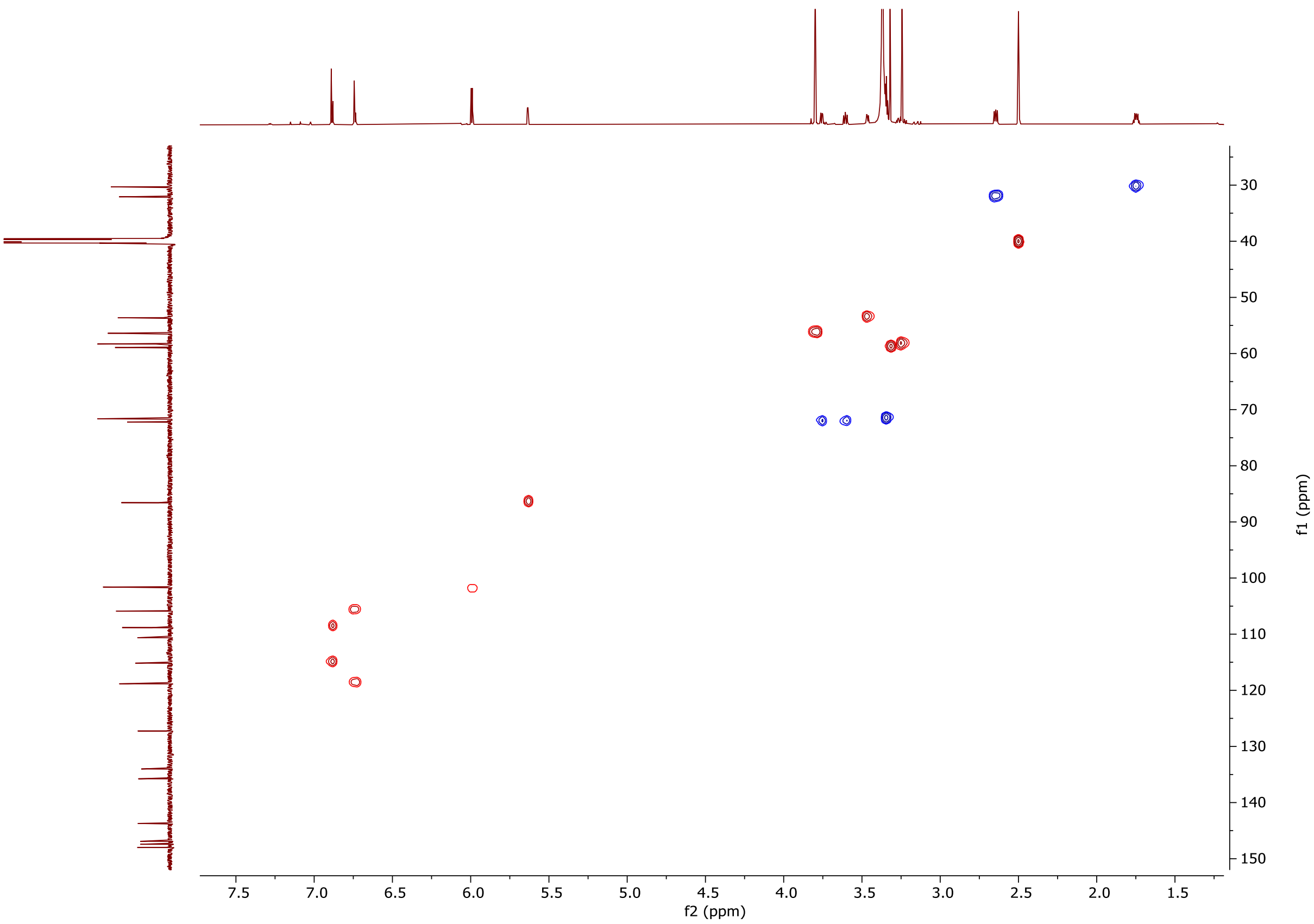

Figure S77 HMBC spectrum of compound **17** in DMSO-d_6

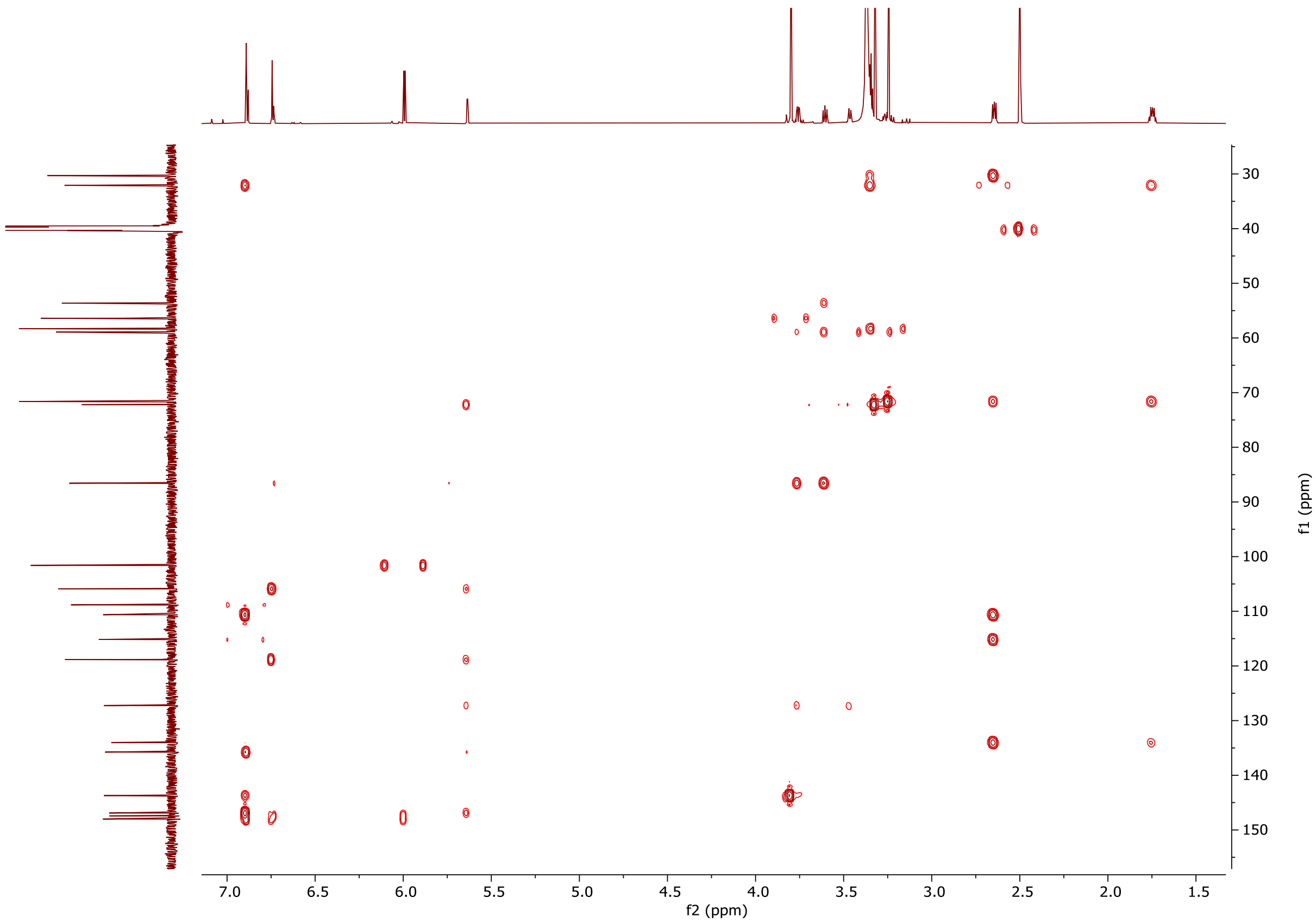

Figure S78 ROESY spectrum of compound **17** in DMSO-d_6

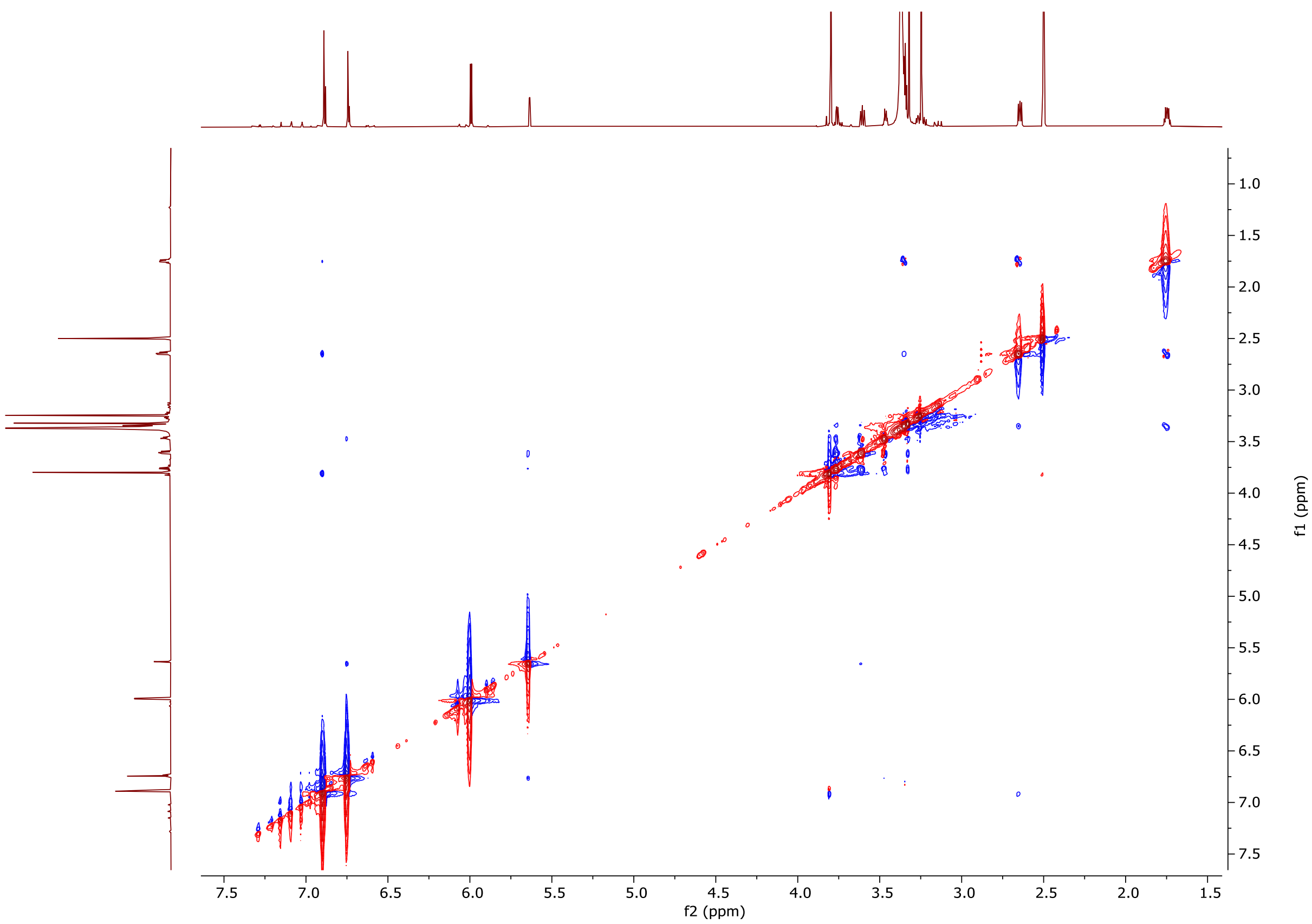

Figure S79 ^{1}H NMR (800 MHz) spectrum of compound **18** in DMSO-d_6

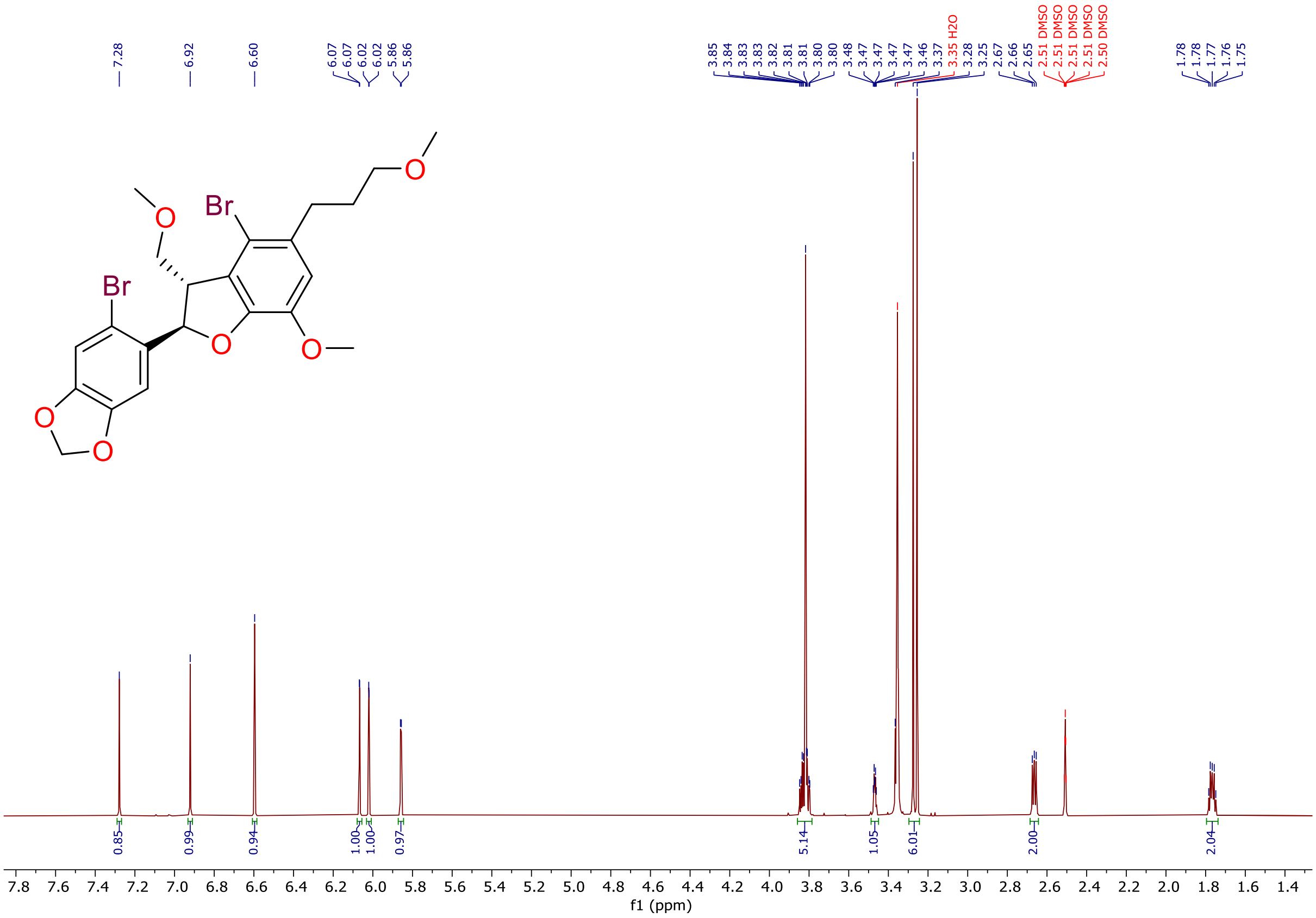

Figure S80 ^{13}C NMR (200 MHz) spectrum of compound **18** in DMSO-d_6

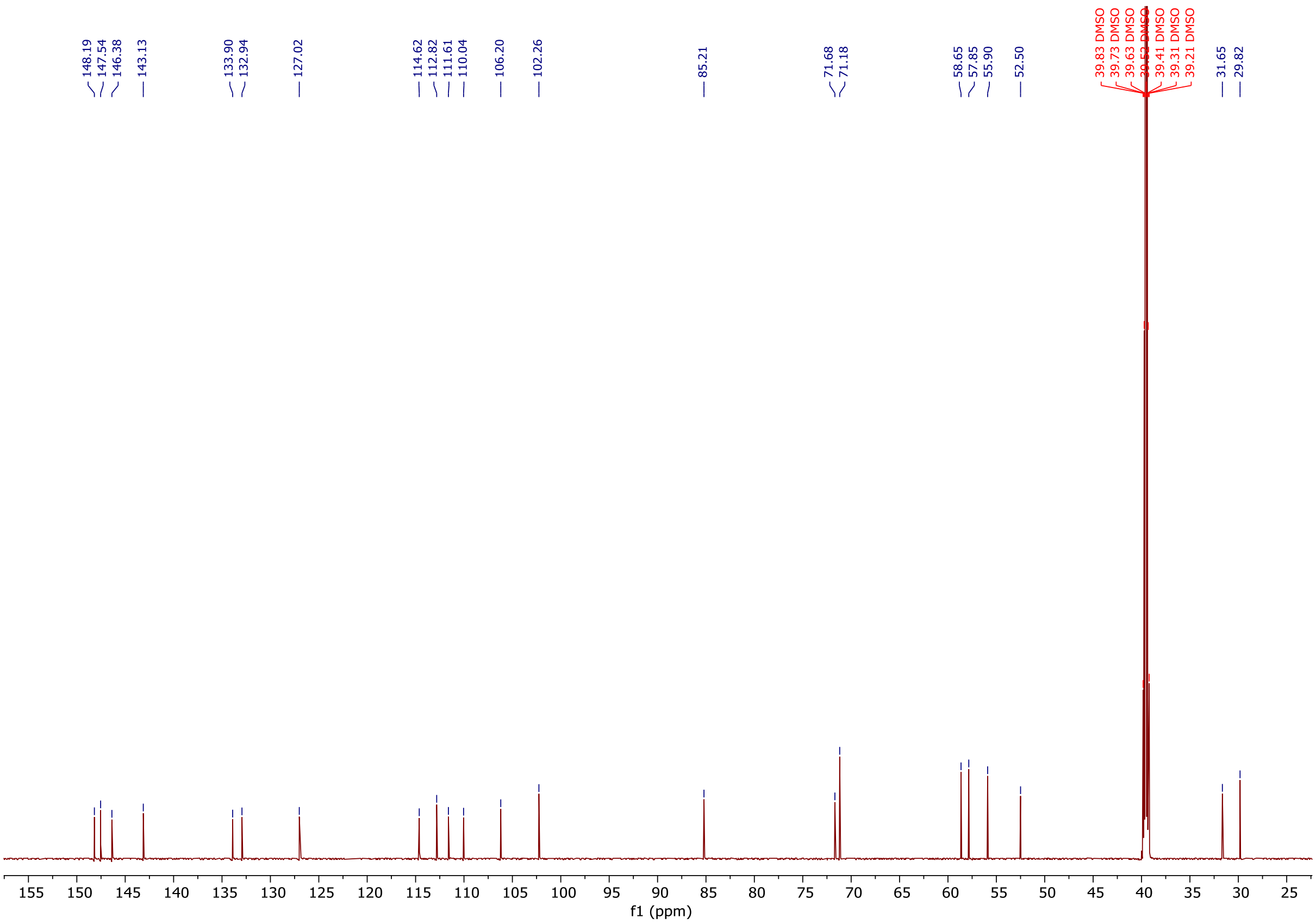

Figure S81 COSY spectrum of compound **18** in DMSO-d_6

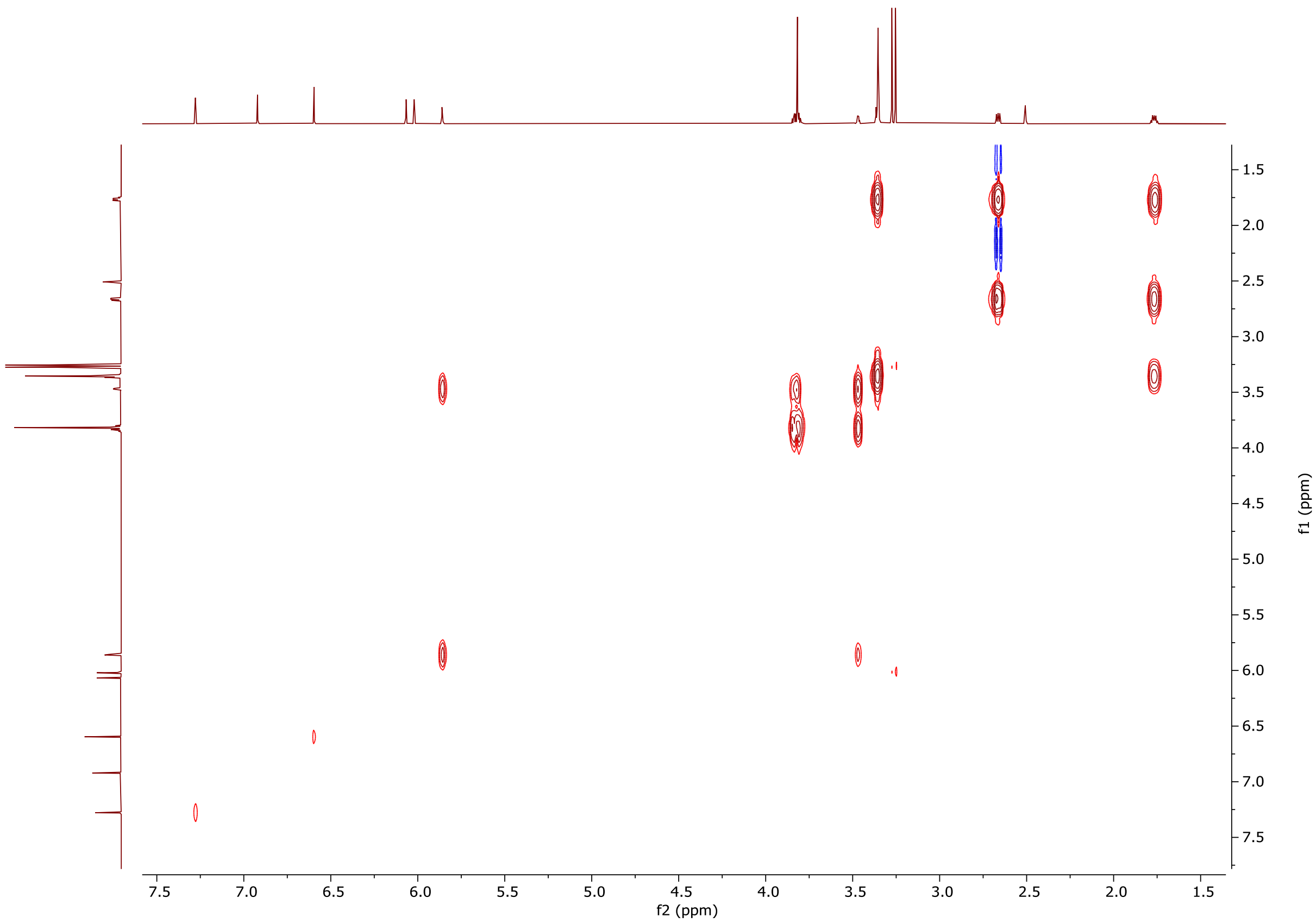

Figure S82 HSQC spectrum of compound **18** in DMSO-d_6

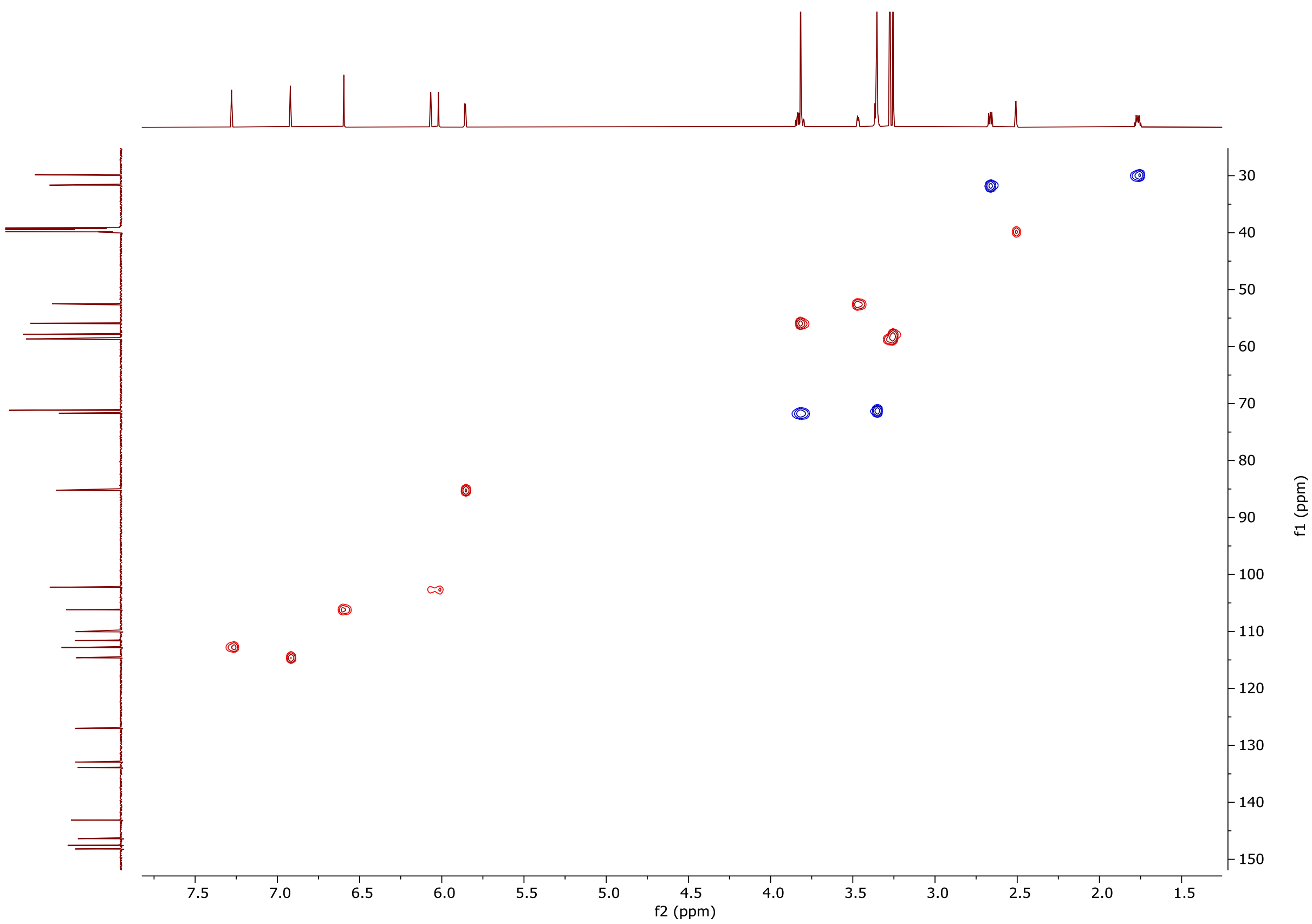

Figure S83 HMBC spectrum of compound **18** in DMSO-d_6

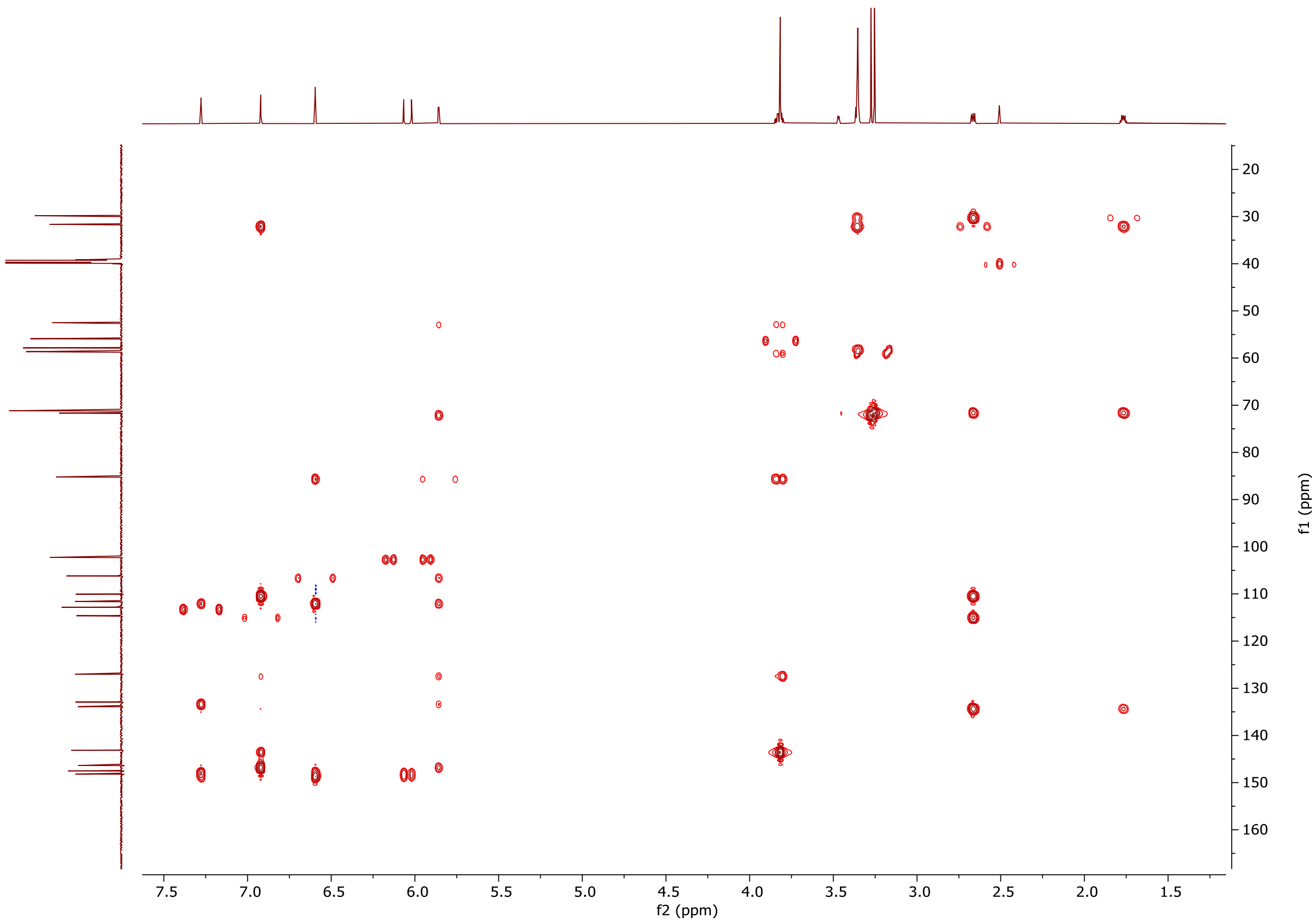

Figure S84 ROESY spectrum of compound **18** in DMSO-d_6

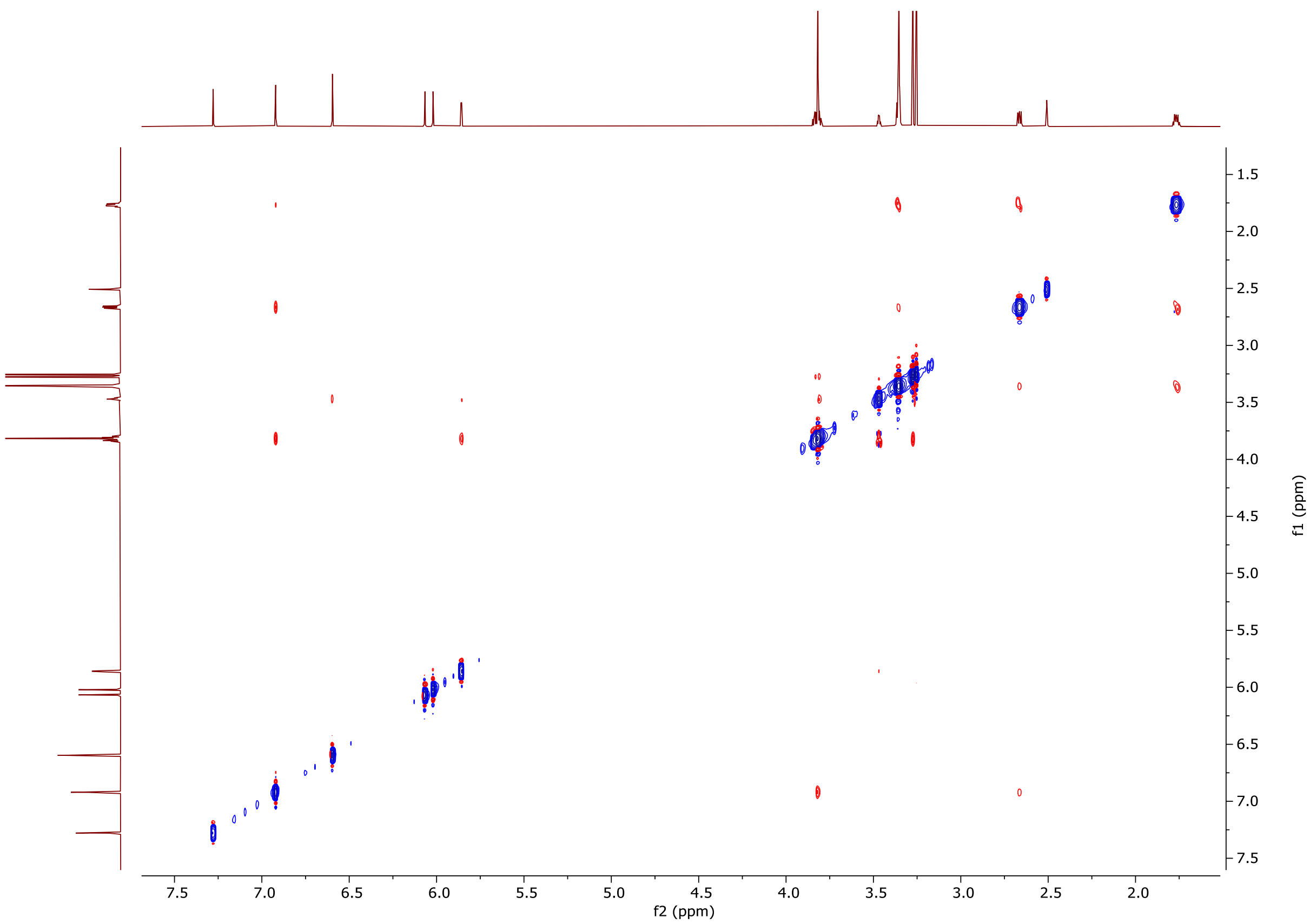

Figure S85 ECD data for suberifolioside B (**1**)

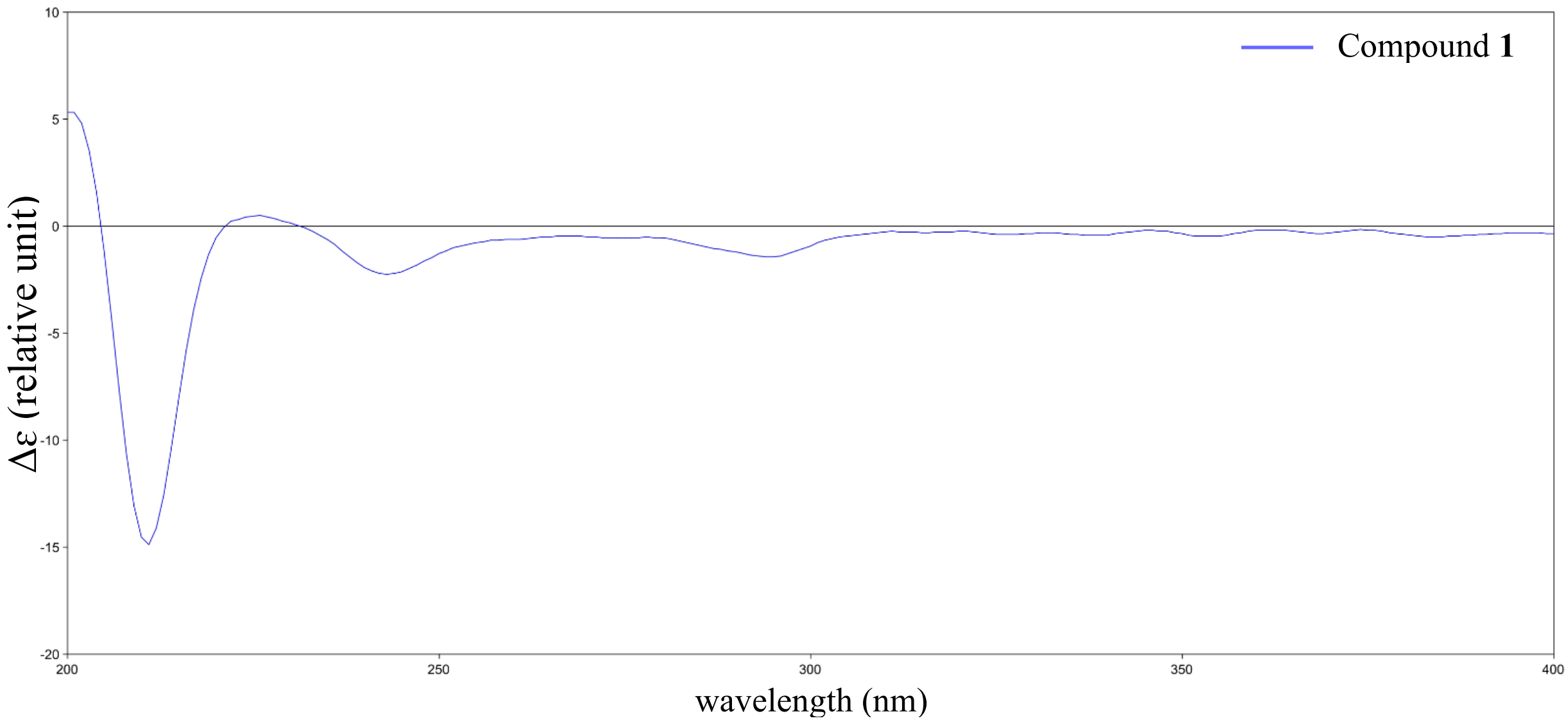

Figure S86 ECD data for 7*R*,8*S*-dihydrodehydrodiconiferyl alcohol (**2**), 3',4-*O*-dimethylcedrusin (**3**), suberifolioside A (**4**), equiselignan B (**5**)

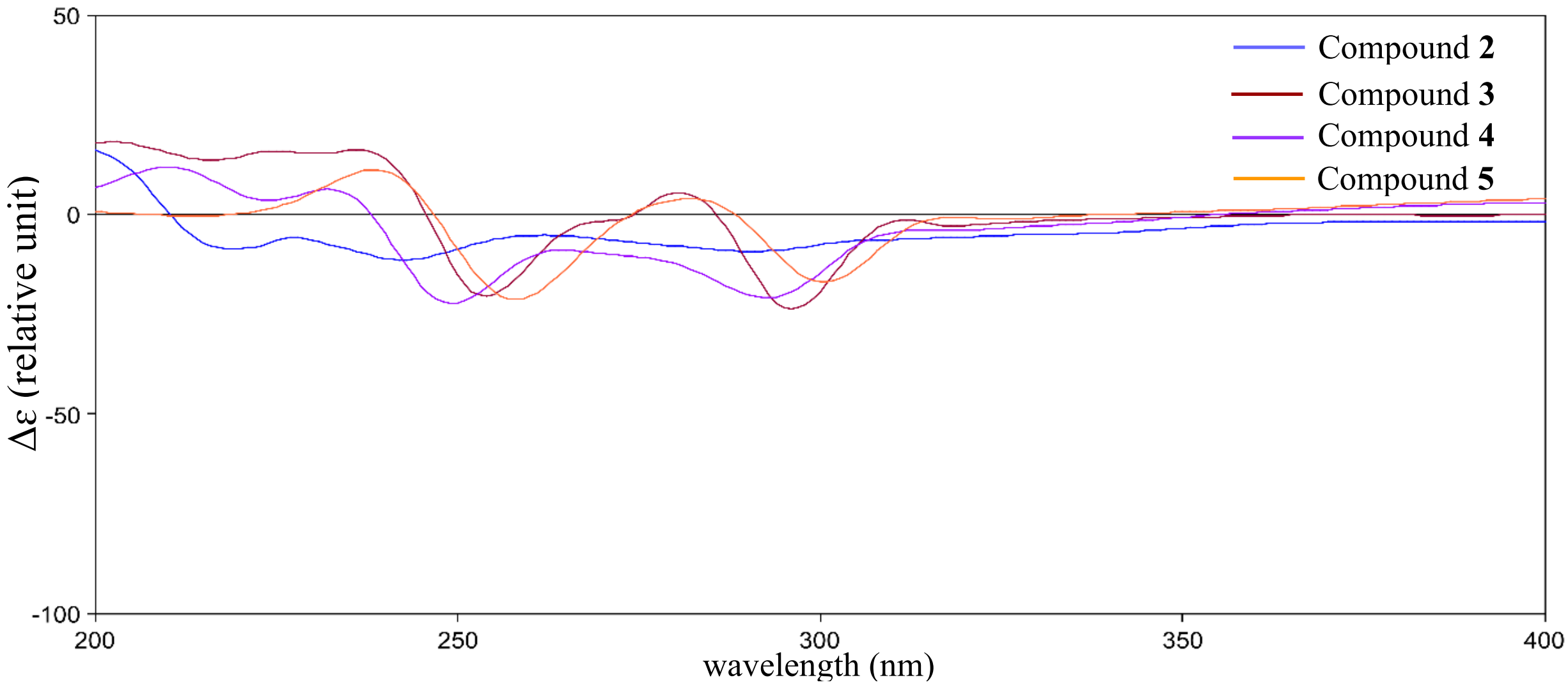

Figure S87 ECD data for the compounds **7** and **12**

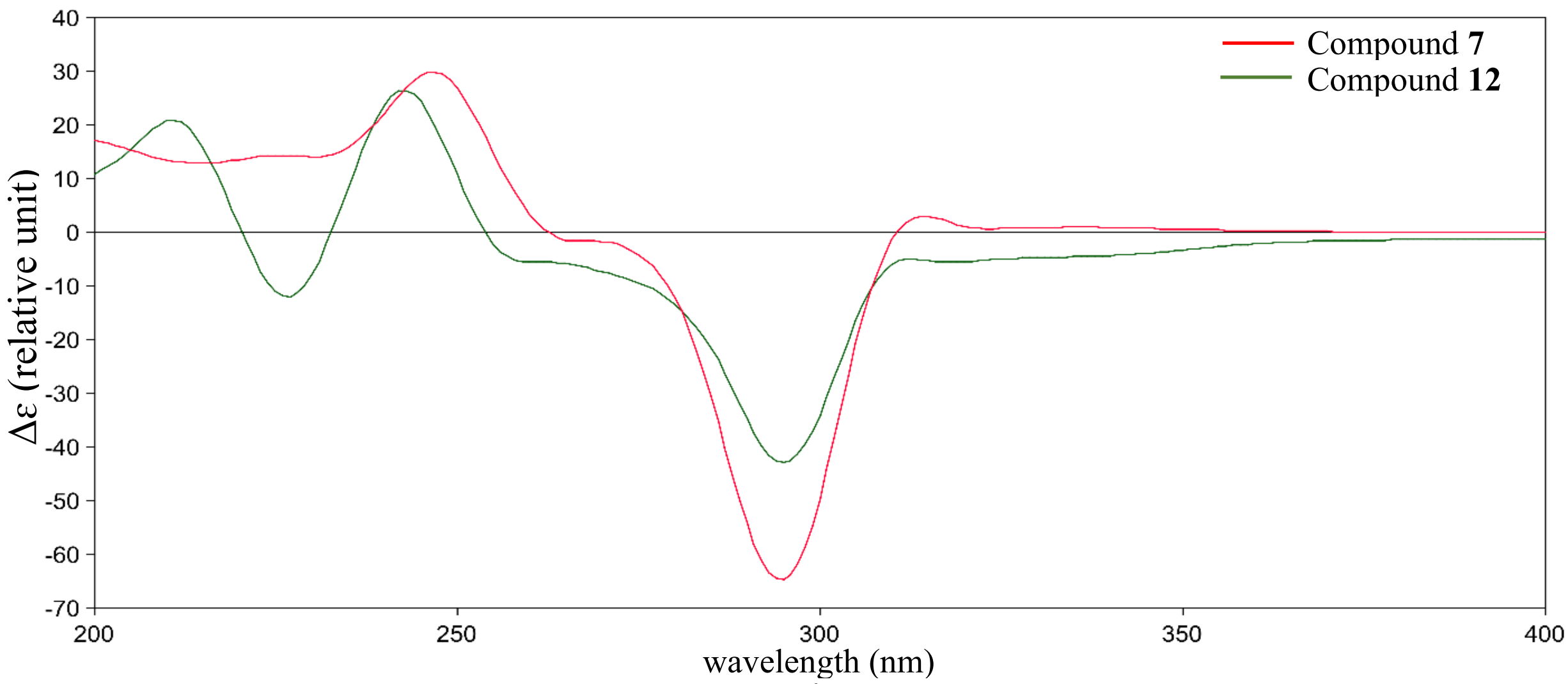

Figure S88 ECD data for the compounds **8–10**, **13** and **14**

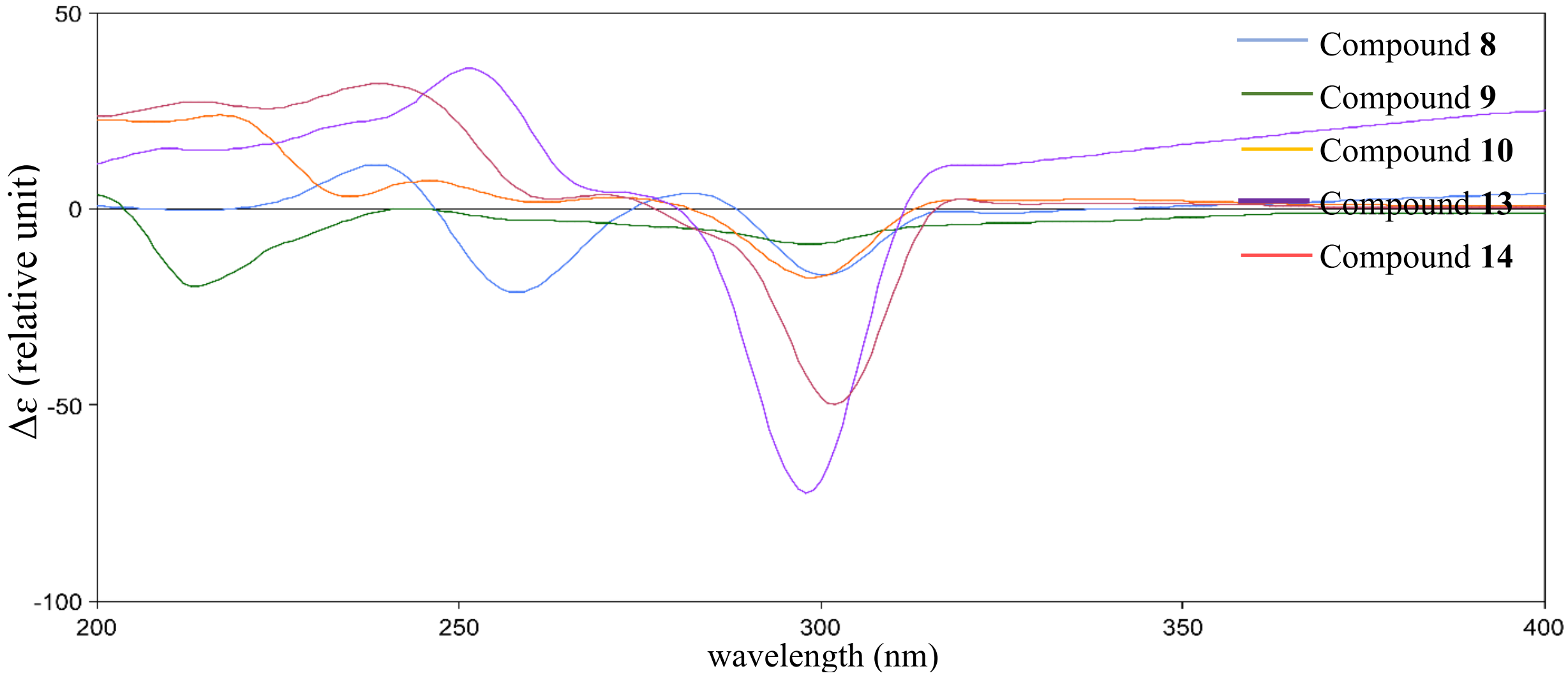

Figure S89 ECD data for the compounds **11** and **15**

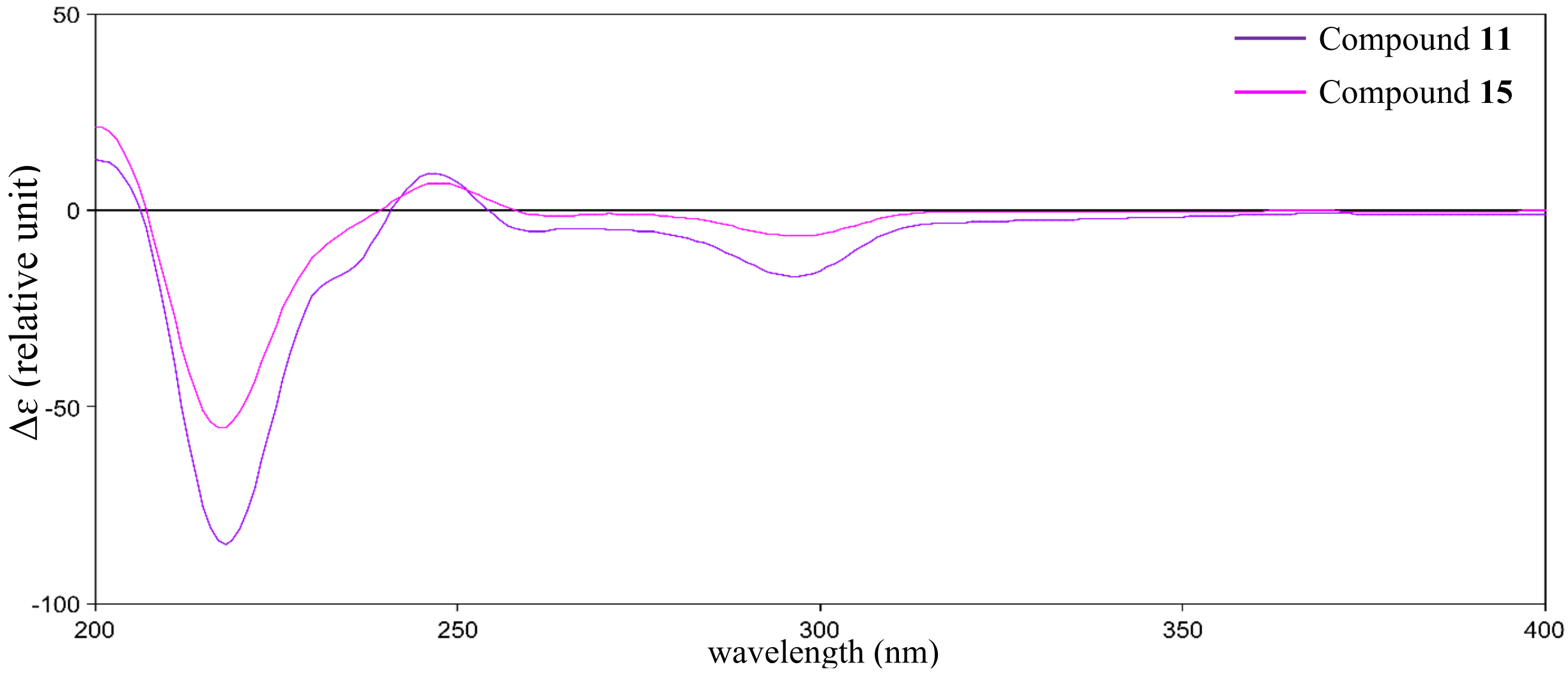

Figure S90 ECD data for the compounds **16–18**

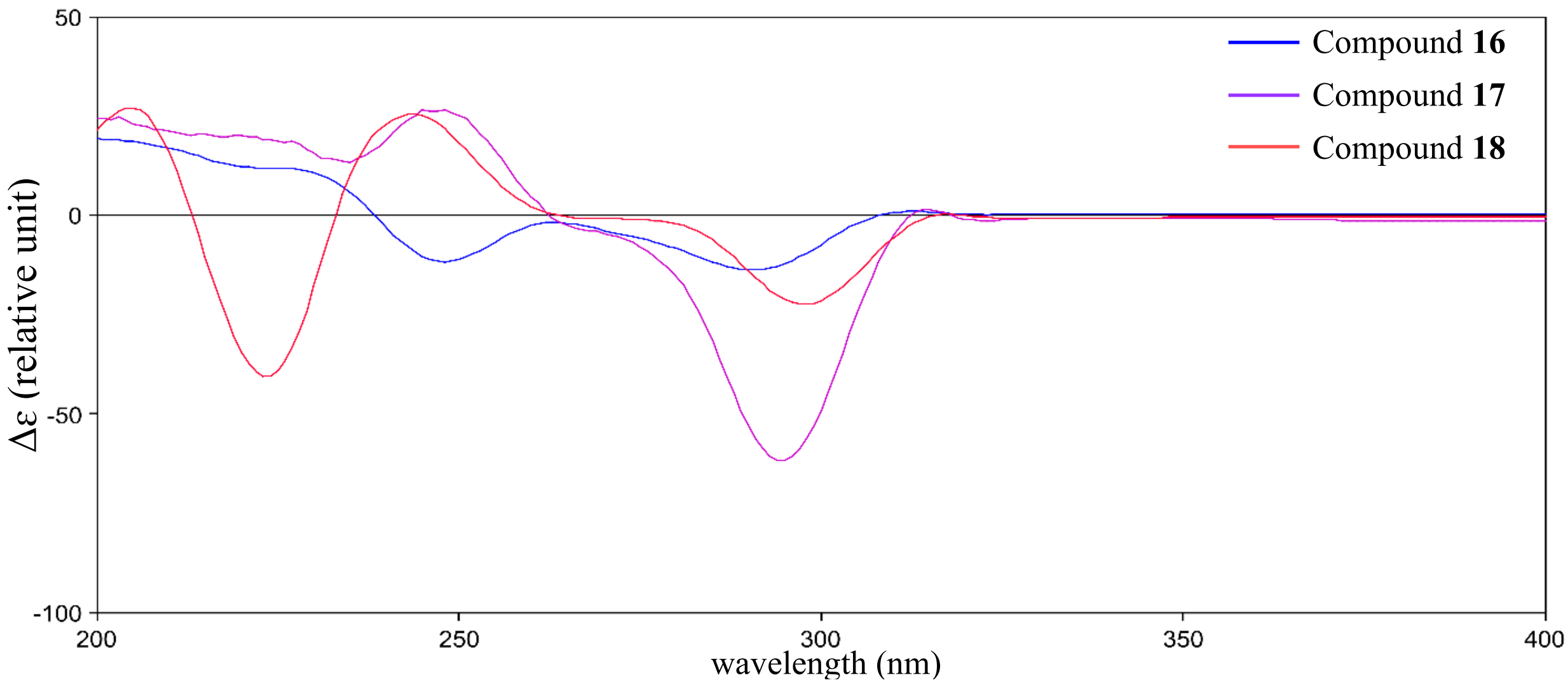

Figure S91 LC-MS data of suberifolioside B (**1**)

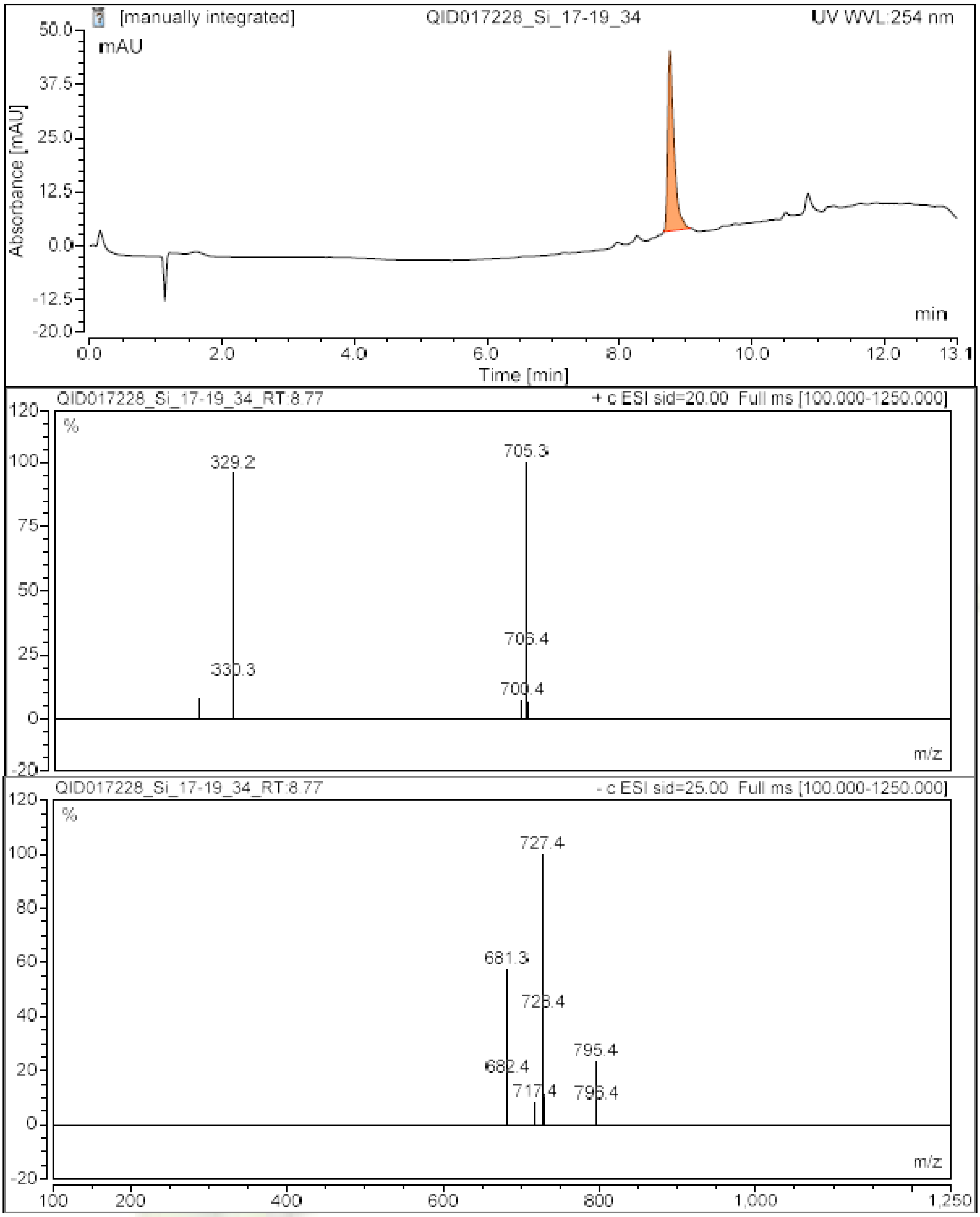

Figure S92 HRESIMS data of suberifolioside B (**1**)

High Resolution Mass Spectrum

Analysis Info

Analysis Name	D:\Data\Kanchana\20240830\RAD1467000001.d		
Method	DirectInfusion_2018_pos.m	Acquisition Date	8/30/2024 11:13:45 AM
Sample Name	RAD1467	Instrument	maXis II ETD 1823391.22321
Comment			

+MS, 0.0min #1-2

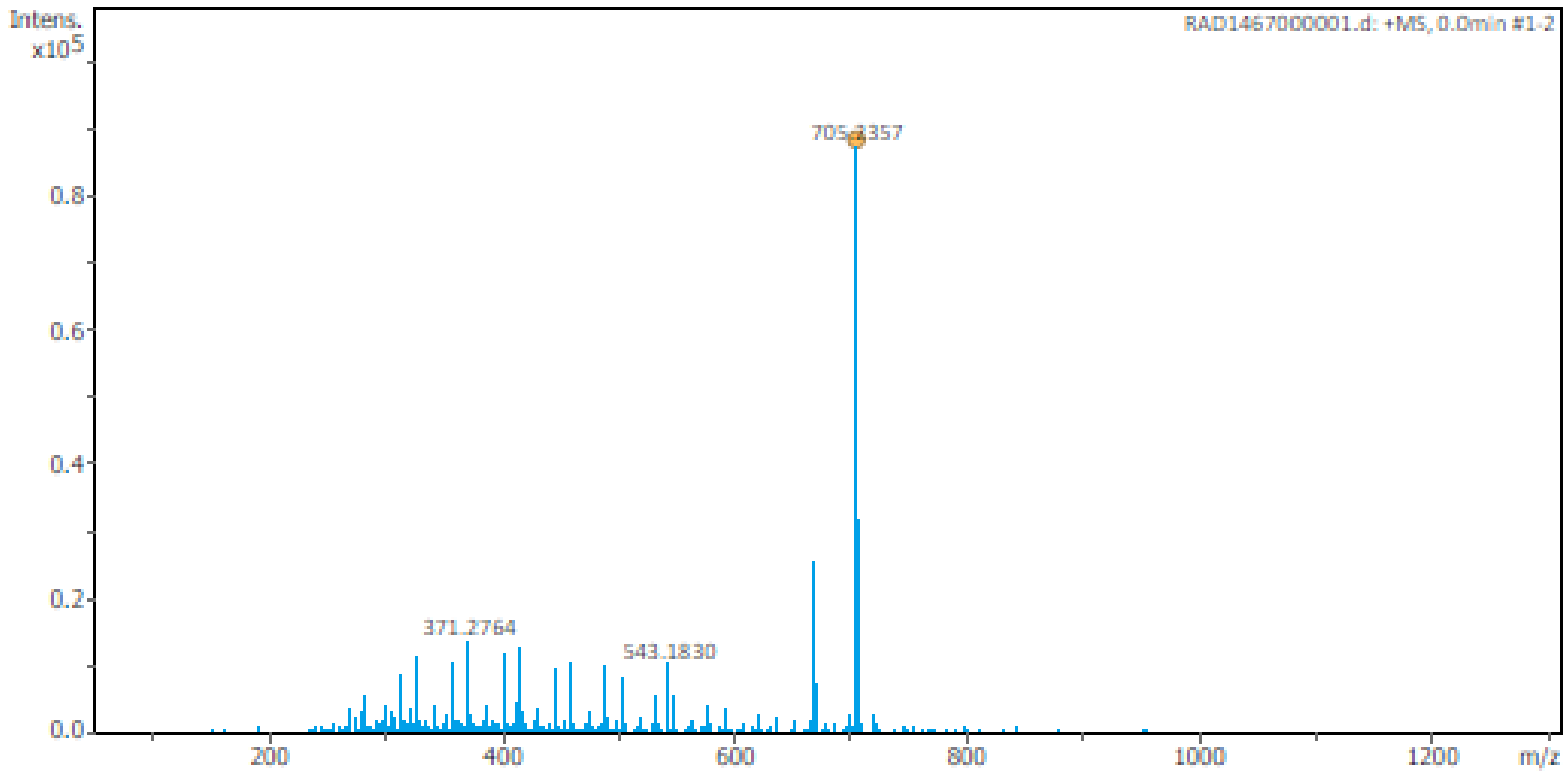

Meas. m/z	#	Ion Formula	m/z	err [ppm]	mSigma	# mSigma	Score	rdb	e⁻ Conf	N-Rule
705.2357	1	C32H42NaO16	705.2365	1.2	2.5	1	100.00	12.0	even	ok

RAD1467000001.d
Bruker Compass DataAnalysis 5.2 printed: 8/30/2024 11:16:26 AM by: demo Page 1 of 1

Figure S93 LC-MS data of 7*R*,8*S*-dihydrodehydrodiconiferyl alcohol (**2**)

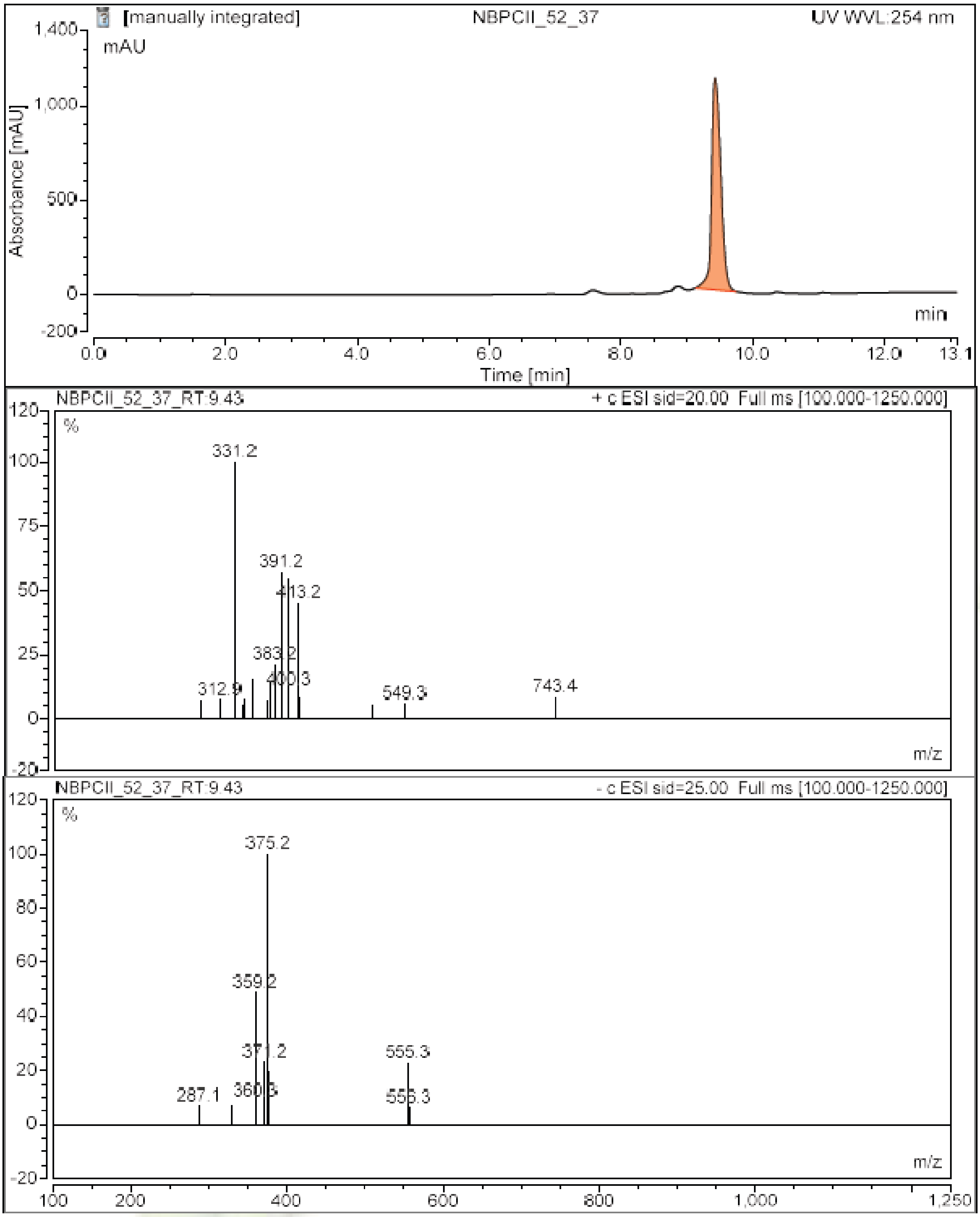

Figure S94 LC-MS data of 3',4-*O*-dimethylcedrusin (**3**)

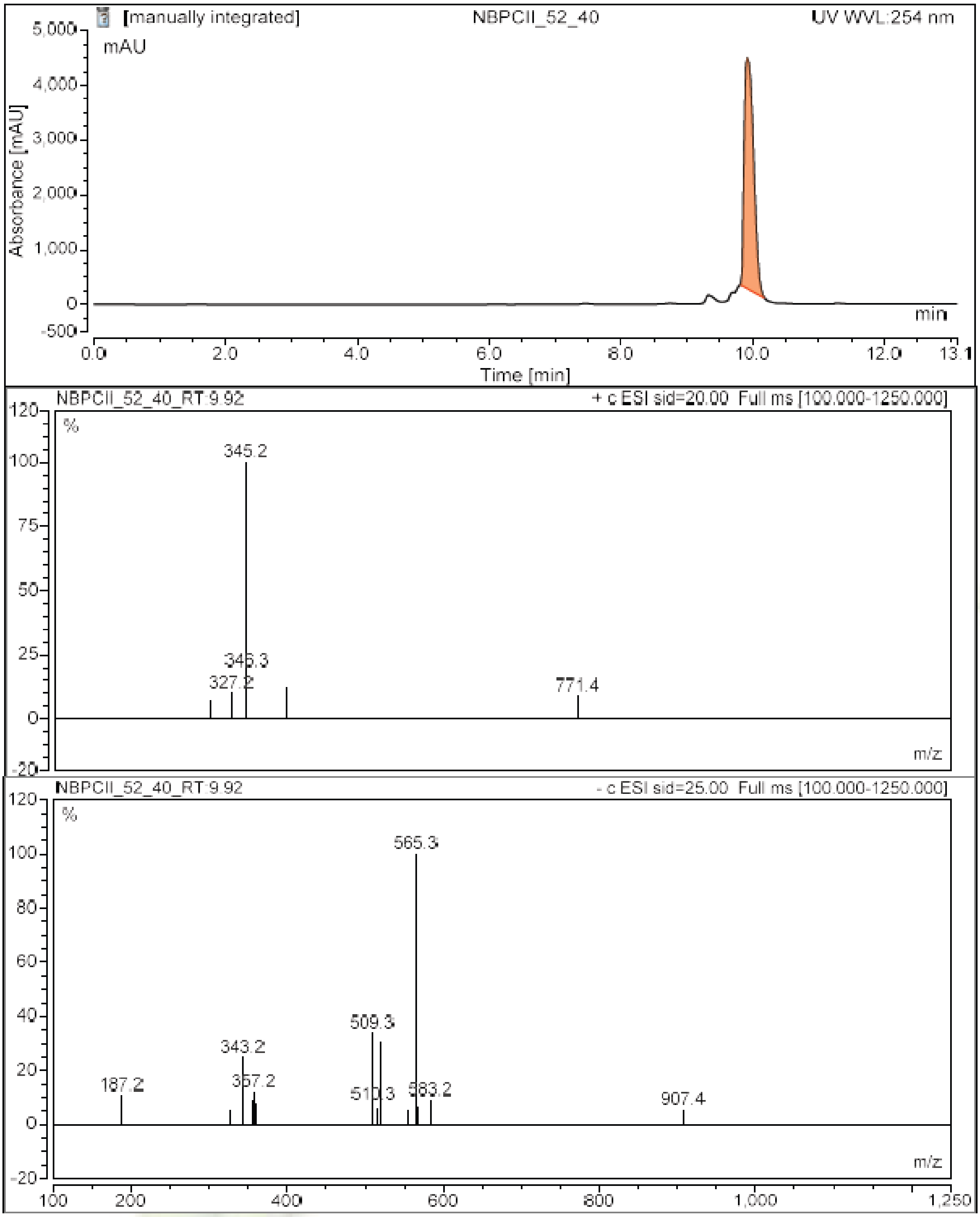

Figure S95 LC-MS data of suberifolioside A (**4**)

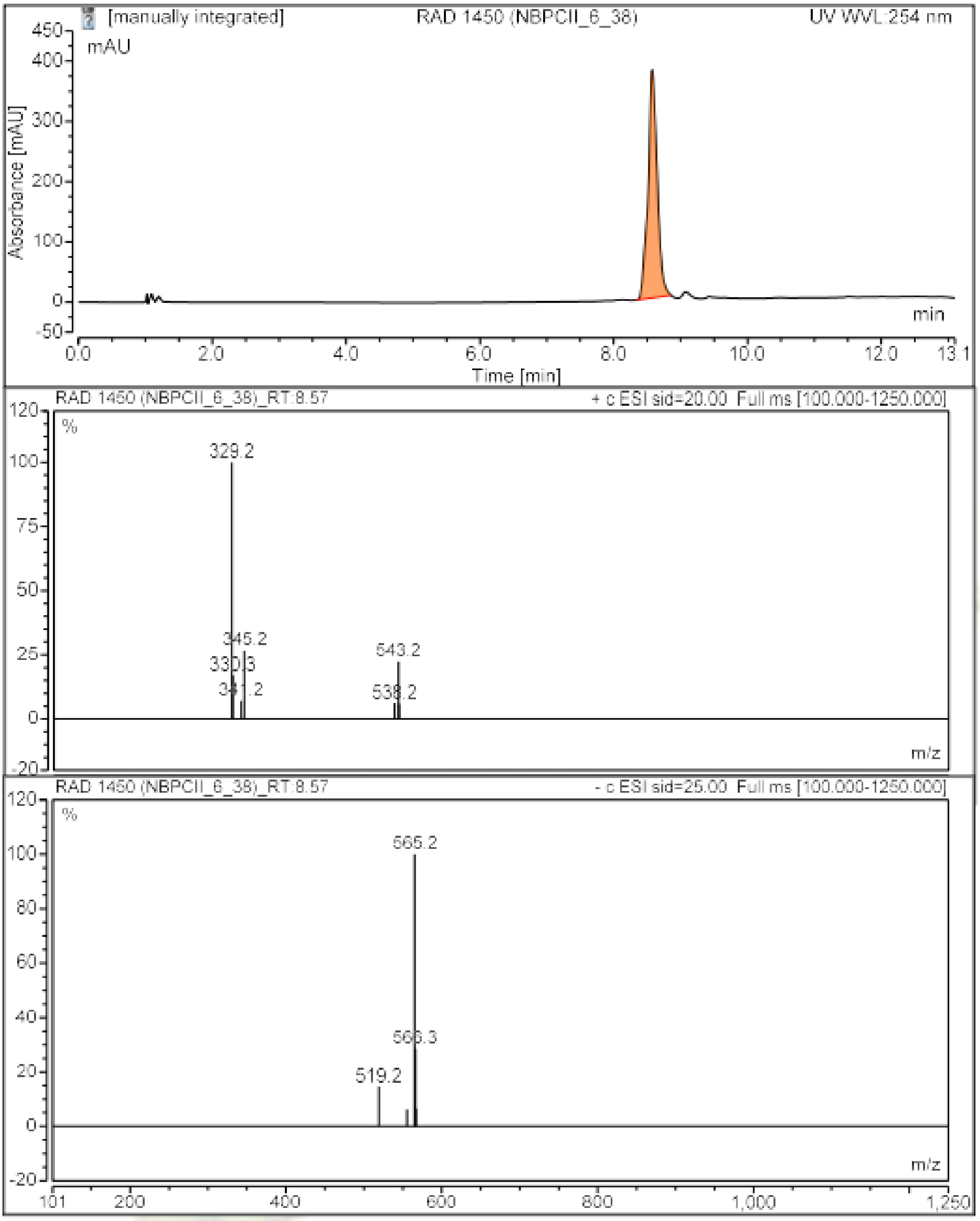

Figure S96 LC-MS data of equiselignan B (**5**)

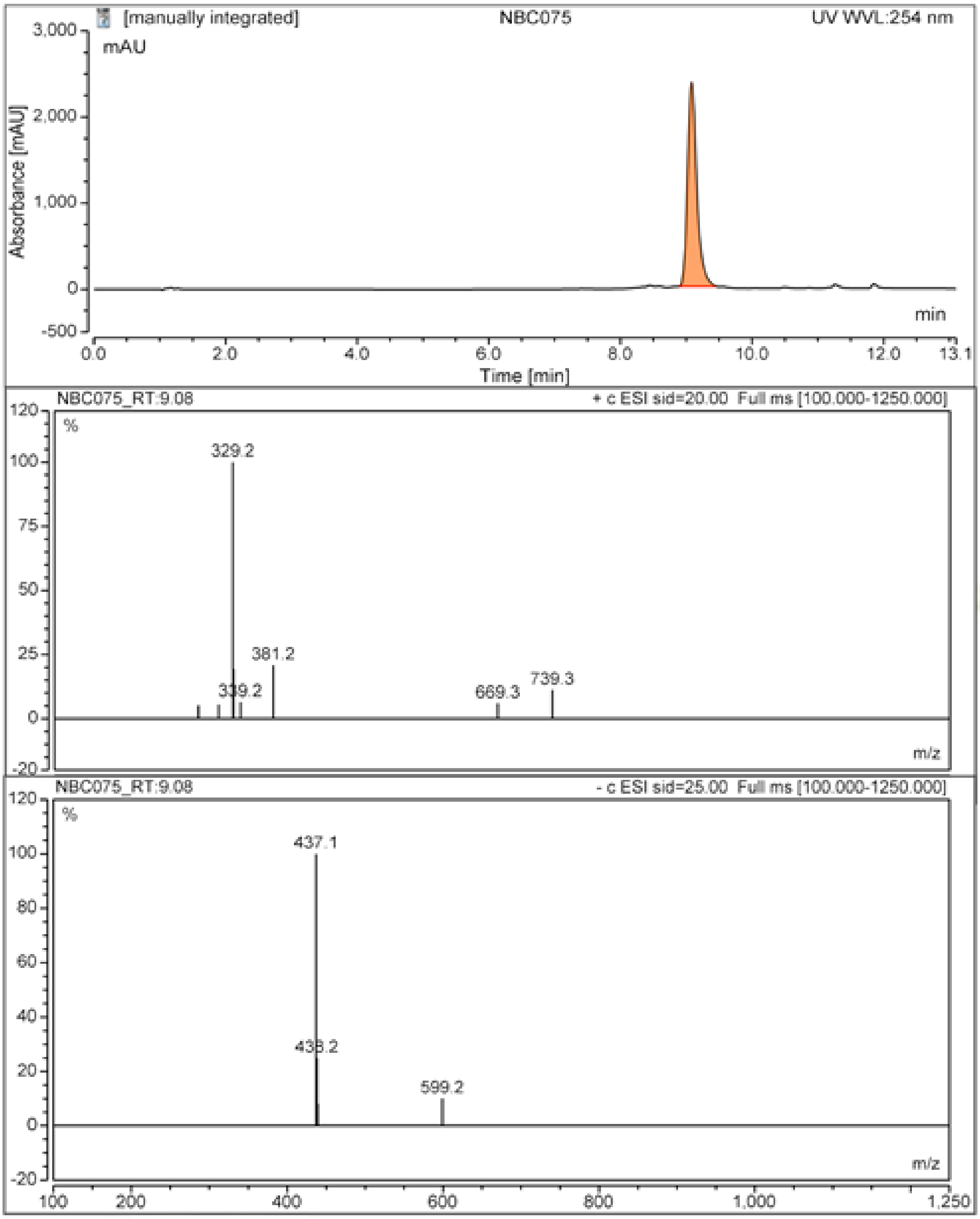

Figure S97 LC-MS data of egonol (**6**)

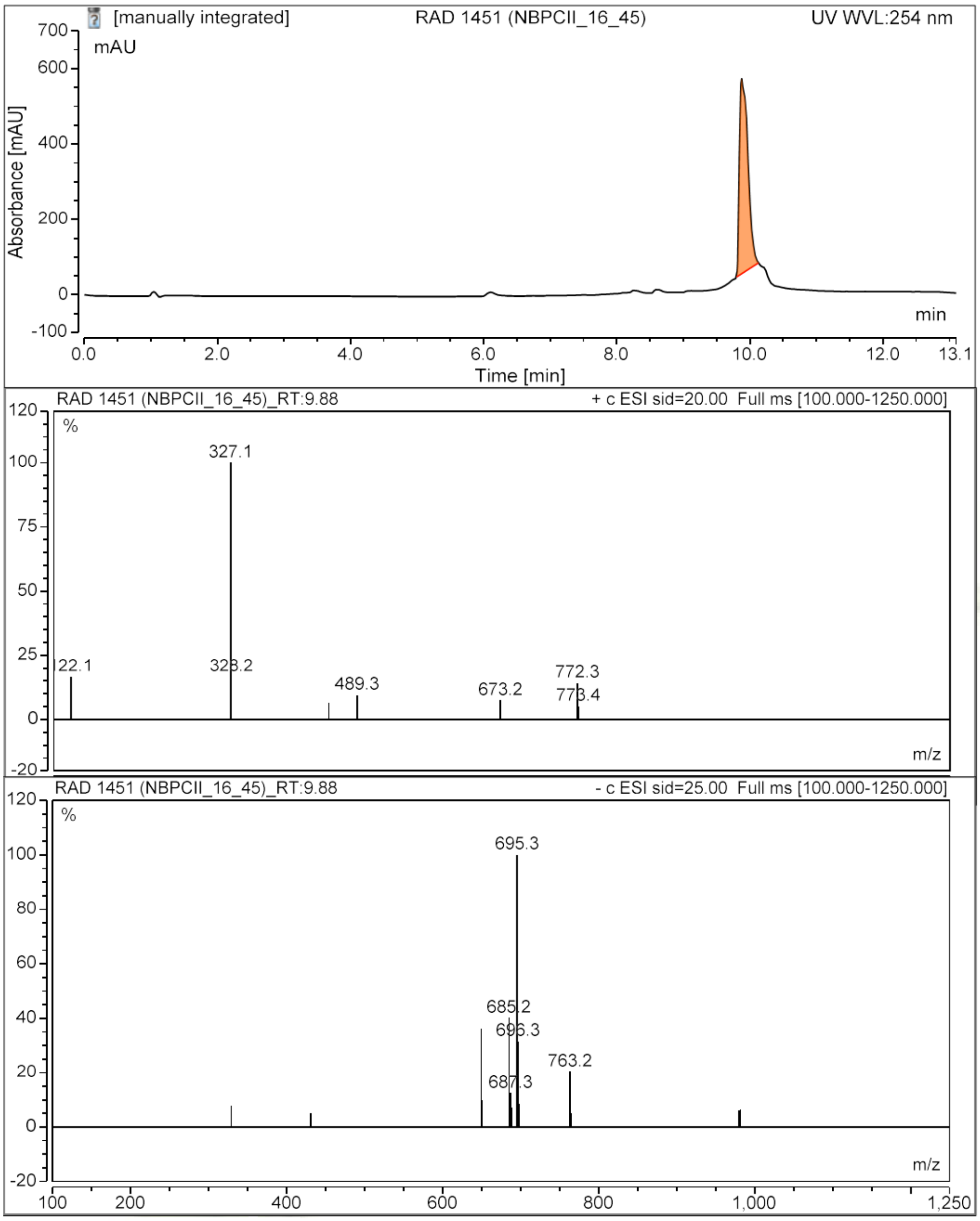

Figure S98 LC-MS data of compound **7**

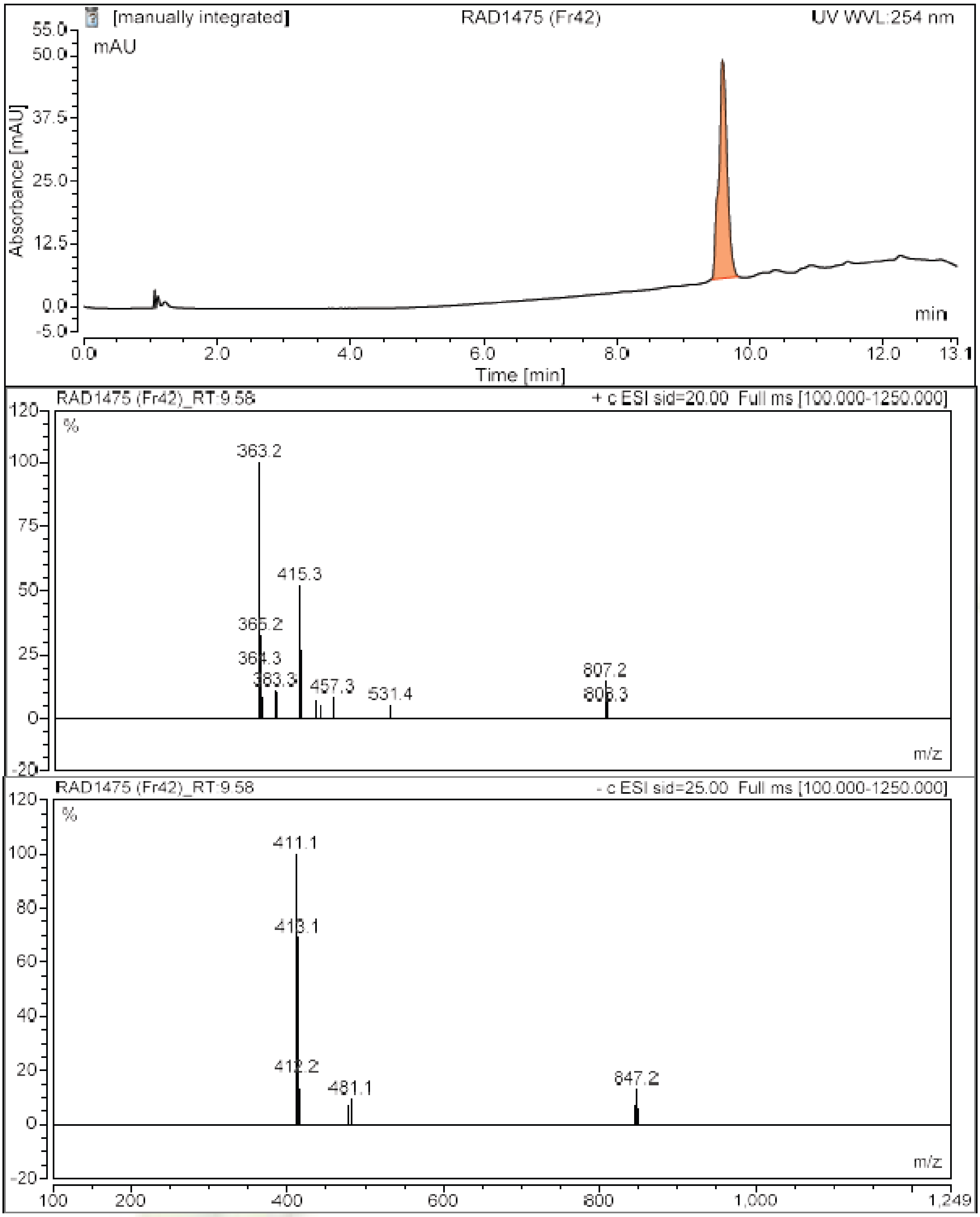

Figure S99 HRESIMS data of compound **7**

Analysis Info

Analysis Name	D:\Data\Kanchana\20240830\RAD1475000001.d		
Method	DirectInfusion_2018_pos.m	Acquisition Date	8/30/2024 11:43:44 AM
Sample Name	RAD1475	Instrument	maXis II ETD 1823391.22321
Comment			

+MS, 0.0min #1

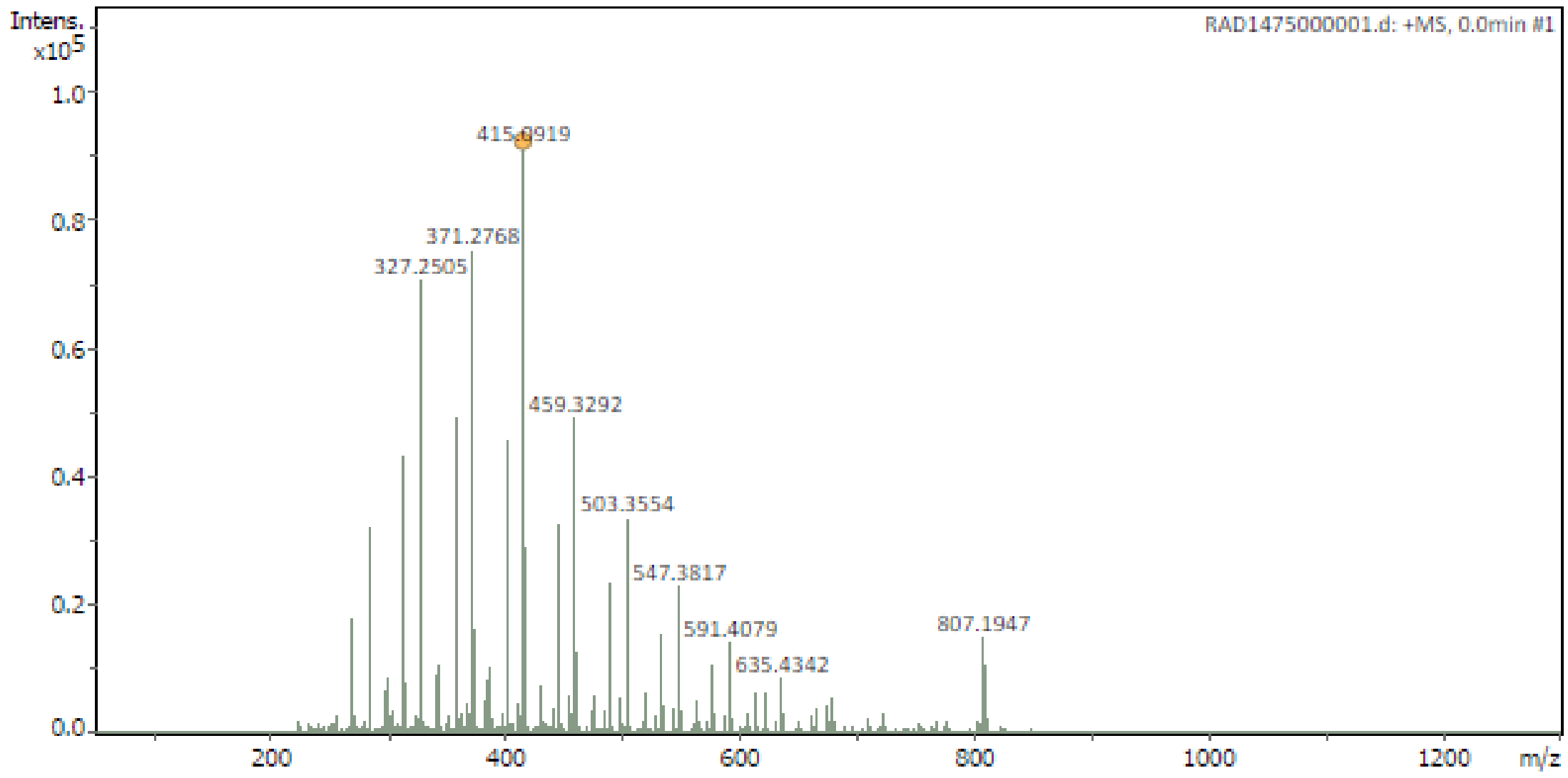

Meas. m/z	#	Ion Formula	m/z	err [ppm]	mSigma	# mSigma	Score	rdb	e⁻ Conf	N-Rule
415.0919	1	C20H21ClNaO6	415.0919	0.0	17.4	1	100.00	13.0	even	ok

Figure S100 LC-MS data of compound **8**

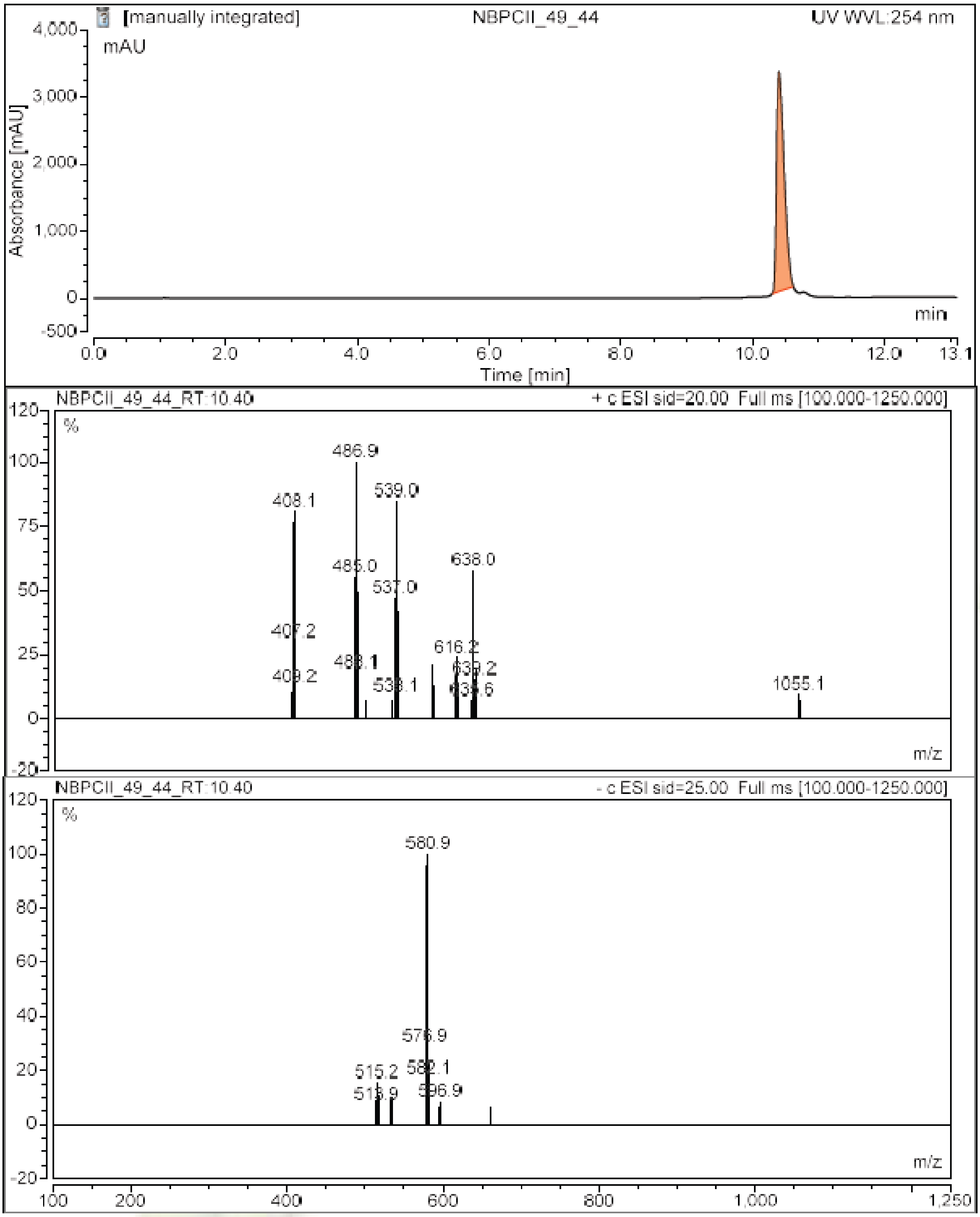

Figure S101 HRESIMS data of compound **8**

High Resolution Mass Spectrum

Analysis Info

Analysis Name D:\Data\Kanchana\20240830\RAD1469000001.d
Method DirectInfusion_2018_pos.m
Sample Name RAD1469
Comment

Acquisition Date 8/30/2024 11:31:56 AM
Instrument maXis II ETD 1823391.22321

+MS, 0.0min #1

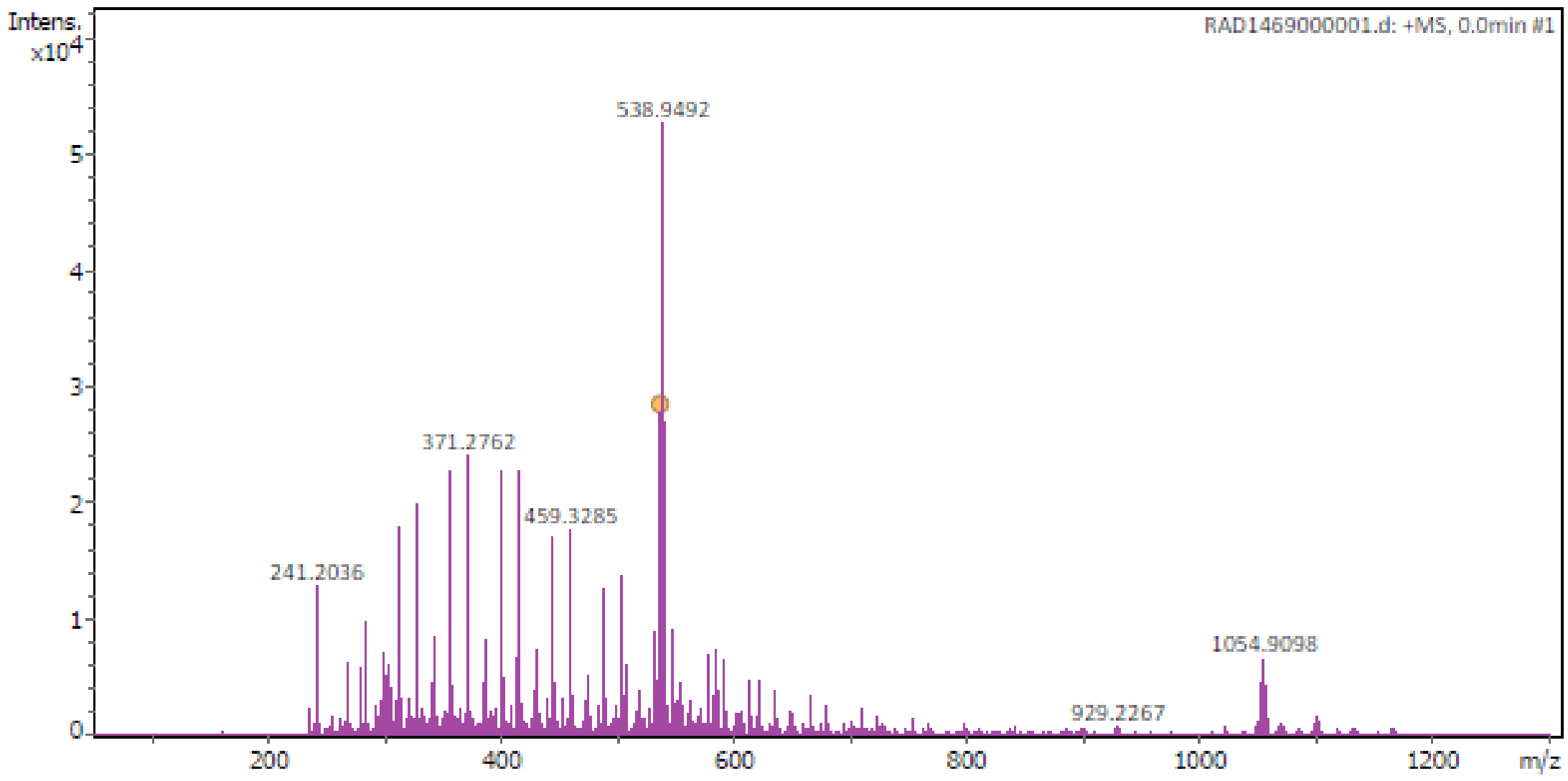

Meas. m/z	#	Ion Formula	m/z	err [ppm]	mSigma	# mSigma	Score	rdb	e⁻ Conf	N-Rule
536.9513	1	C20H20Br2NaO6	536.9519	1.0	9.1	1	100.00	14.0	even	ok

Figure S102 LC-MS data of compound **9**

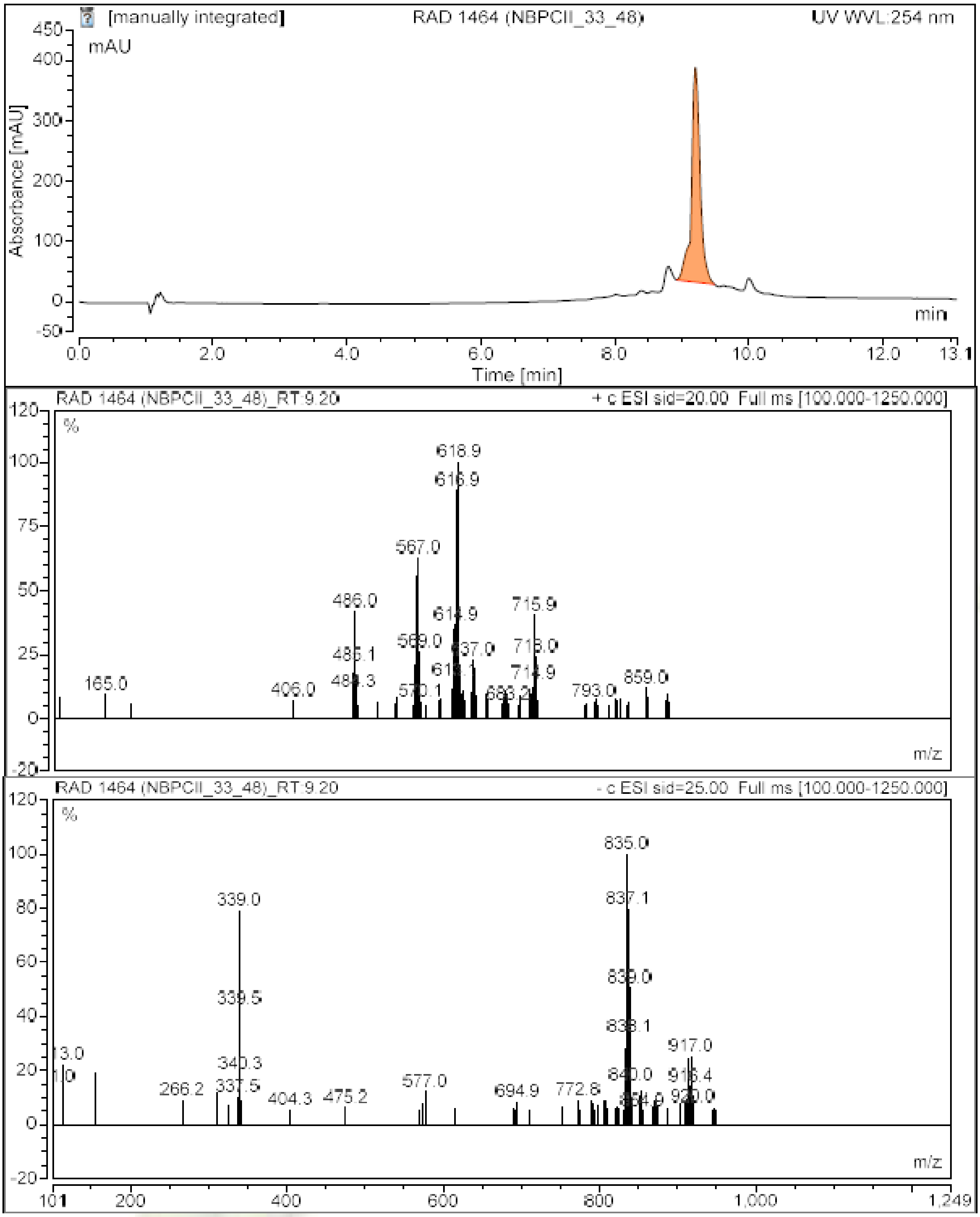

Figure S103 HRESIMS data of compound **9**

Mass Spectrum SmartFormula Report

Analysis Info

Analysis Name	D:\Data\Kanchana\20240108\NBC075_3Br000001.d	Acquisition Date	1/9/2024 12:43:12 PM
Method	DirectInfusion_2018_pos.m	Operator	Demo User
Sample Name	NBC075_3Br	Instrument	maXis II ETD 1823391.22321
Comment			

Acquisition Parameter

Source Type	ESI	Ion Polarity	Positive	Set Nebulizer	0.4 Bar
Focus	Not active	Set Capillary	4500 V	Set Dry Heater	180 °C
Scan Begin	50 m/z	Set End Plate Offset	-500 V	Set Dry Gas	4.0 l/min
Scan End	1300 m/z	Set Collision Cell RF	500.0 Vpp	Set Divert Valve	Waste

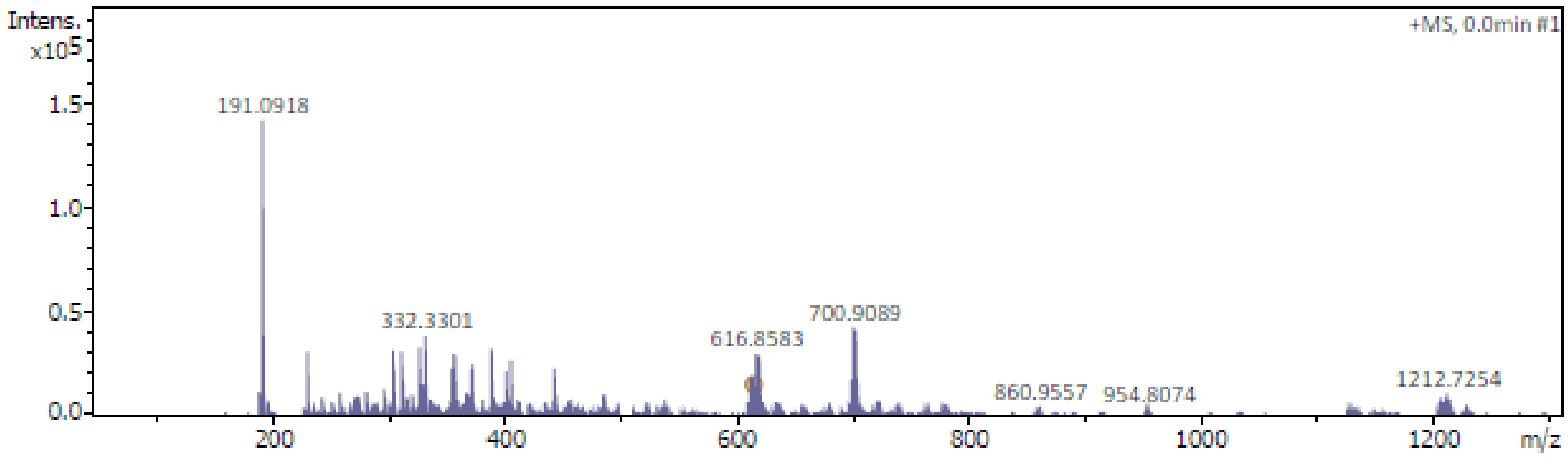

Meas. m/z	#	Ion Formula	m/z	err [ppm]	mSigma	# mSigma	Score	rdb	e⁻ Conf	N-Rule
614.8597	1	C20H19Br3NaO6	614.8624	4.3	11.5	1	51.17	16.0	even	ok

Figure S104 LC-MS data of compound **10**

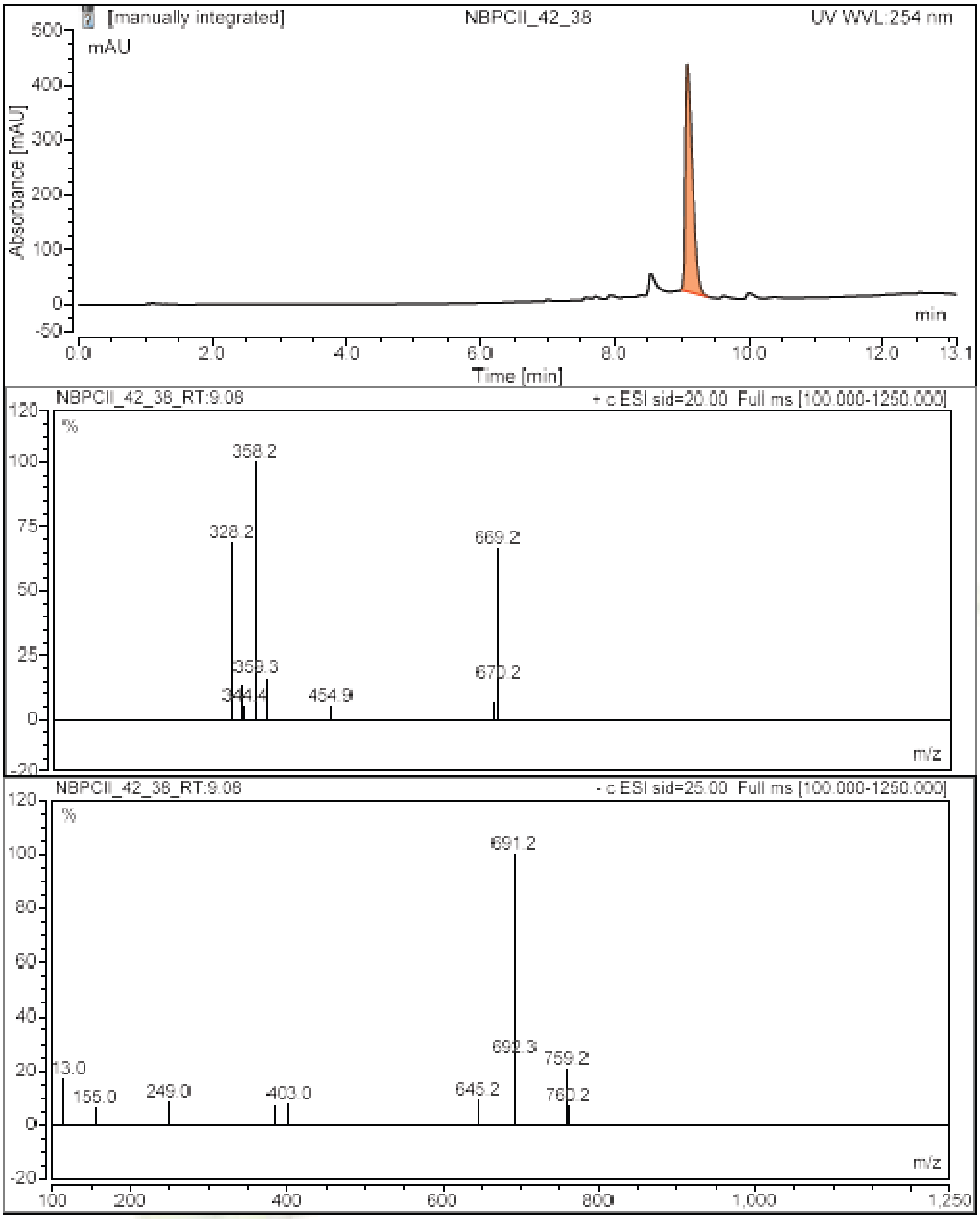

Figure S105 HRESIMS data of compound **10**

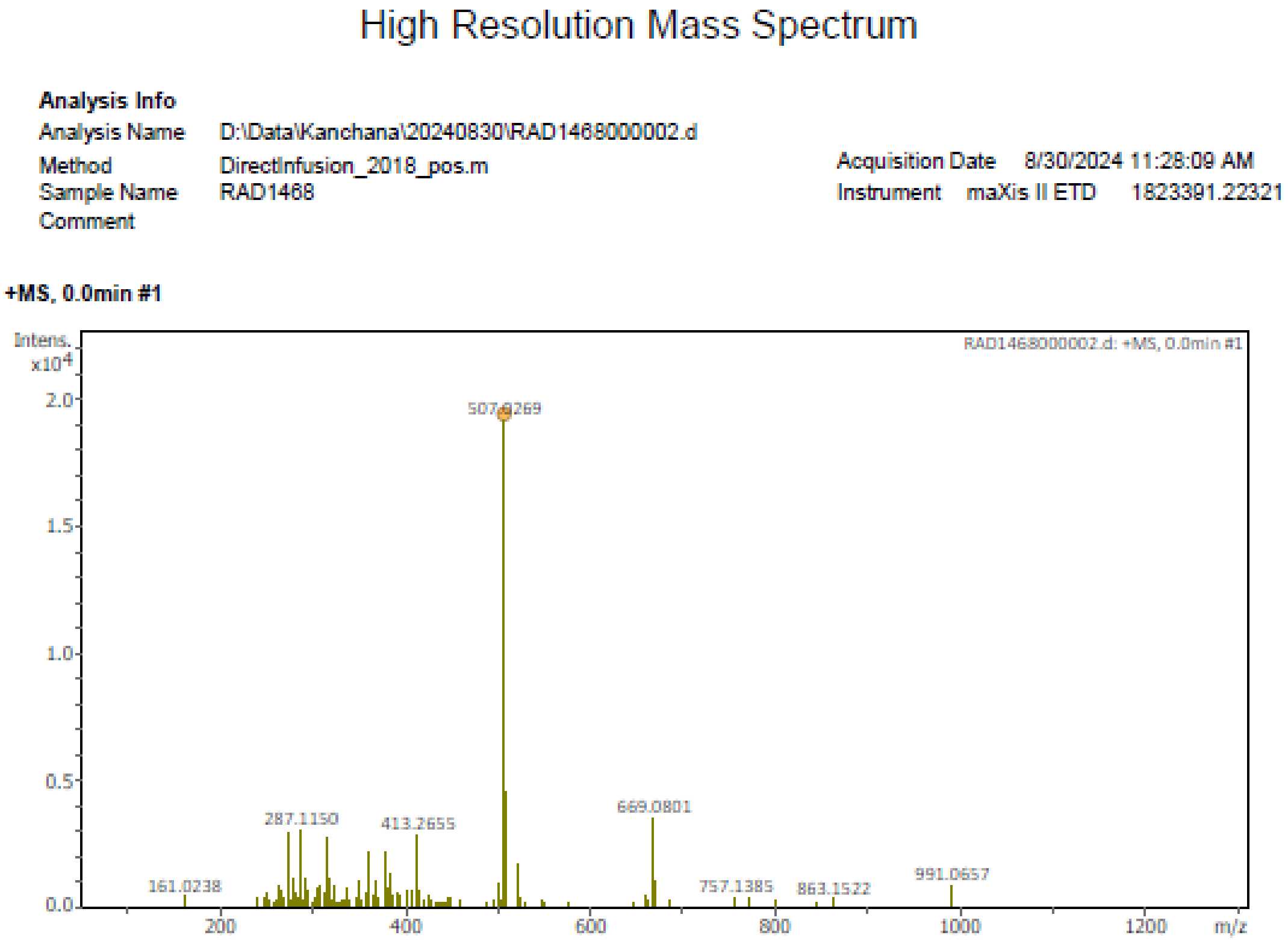

Meas. m/z	#	Ion Formula	m/z	err [ppm]	mSigma	# mSigma	Score	rdb	e⁻ Conf	N-Rule
507.0269	1	C20H21INaO6	507.0275	1.1	11.4	1	100.00	13.0	even	ok

RAD1468000002.d
Bruker Compass DataAnalysis 5.2 printed: 8/30/2024 11:29:04 AM by: demo Page 1 of 1

Figure S106 LC-MS data of compound **11**

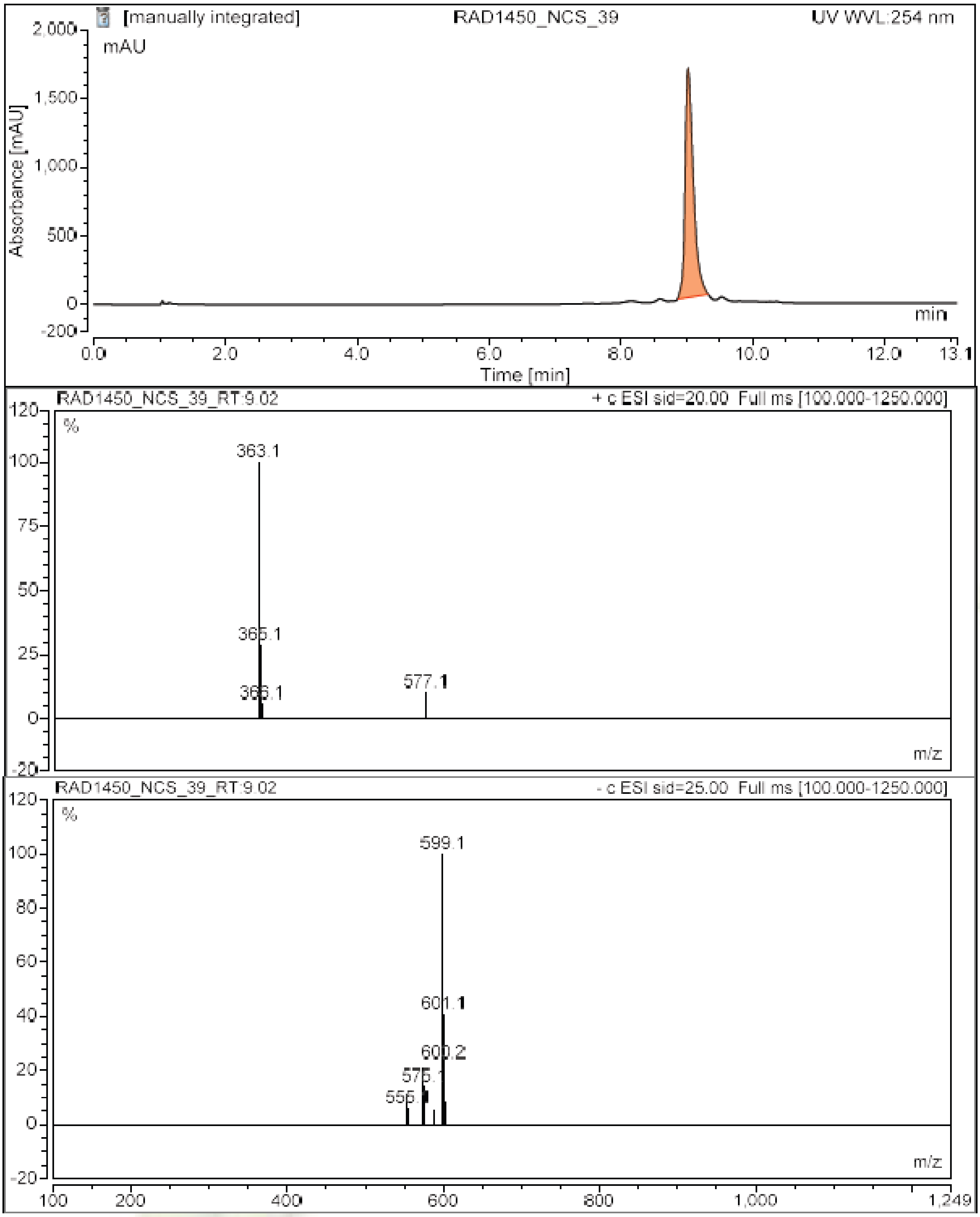

Figure S107 HRESIMS data of compound **11**

High Resolution Mass Spectrum

Analysis Info

Analysis Name D:\Data\Kanchana\20240830\RAD1465000001.d
Method DirectInfusion_2018_pos.m
Sample Name RAD1465
Comment

Acquisition Date 8/30/2024 11:00:11 AM
Instrument maXis II ETD 1823391.22321

+MS, 0.0min #2

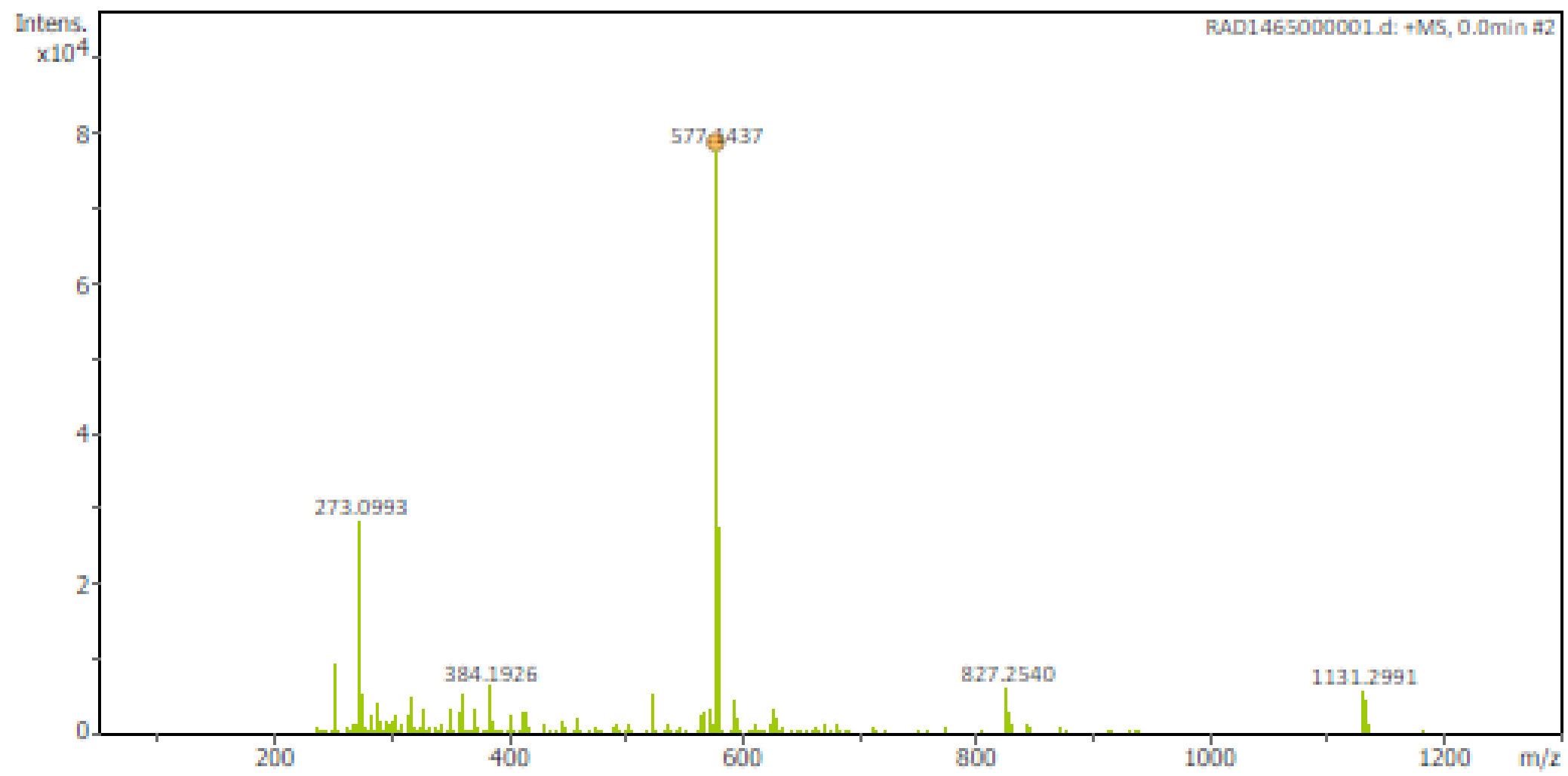

Meas. m/z	#	Ion Formula	m/z	err [ppm]	mSigma	# mSigma	Score	rdb	e⁻ Conf	N-Rule
577.1437	1	C26H31ClNaO11	577.1447	1.8	13.8	1	100.00	14.0	even	ok

Figure S108 LC-MS data of compound **12**

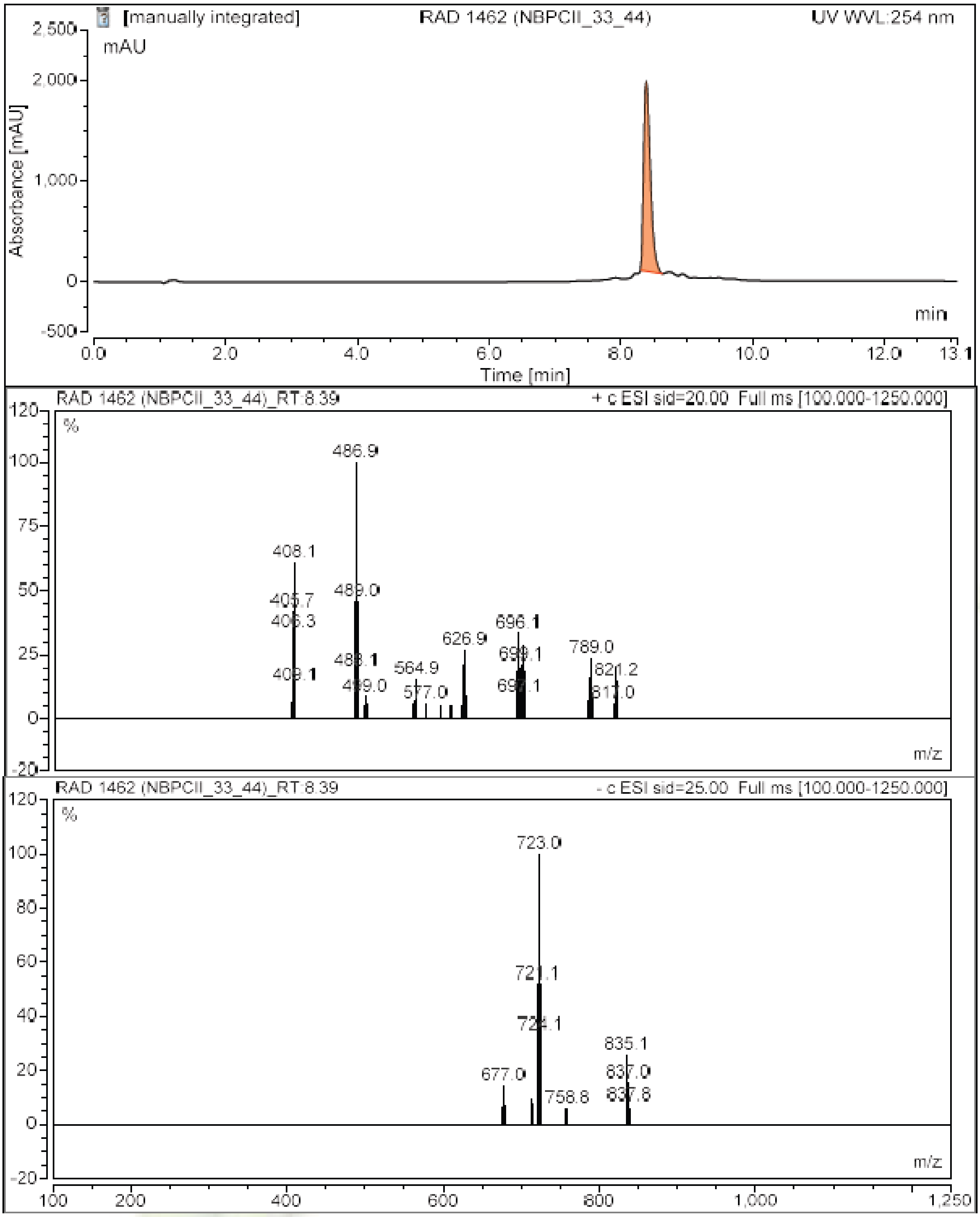

Figure S109 HRESIMS data of compound **12**

High Resolution Mass Spectrum

Analysis Info

Analysis Name D:\Data\Kanchana\20250815\RAD1462(RAD1450_2Br)000001.d
Method DirectInfusion_2018_pos.m
Sample Name RAD1462(RAD1450_2Br)
Comment

Acquisition Date 8/15/2025 2:10:12 PM
Instrument maXis II ETD 1823391.22321

+MS, 0.1min #4

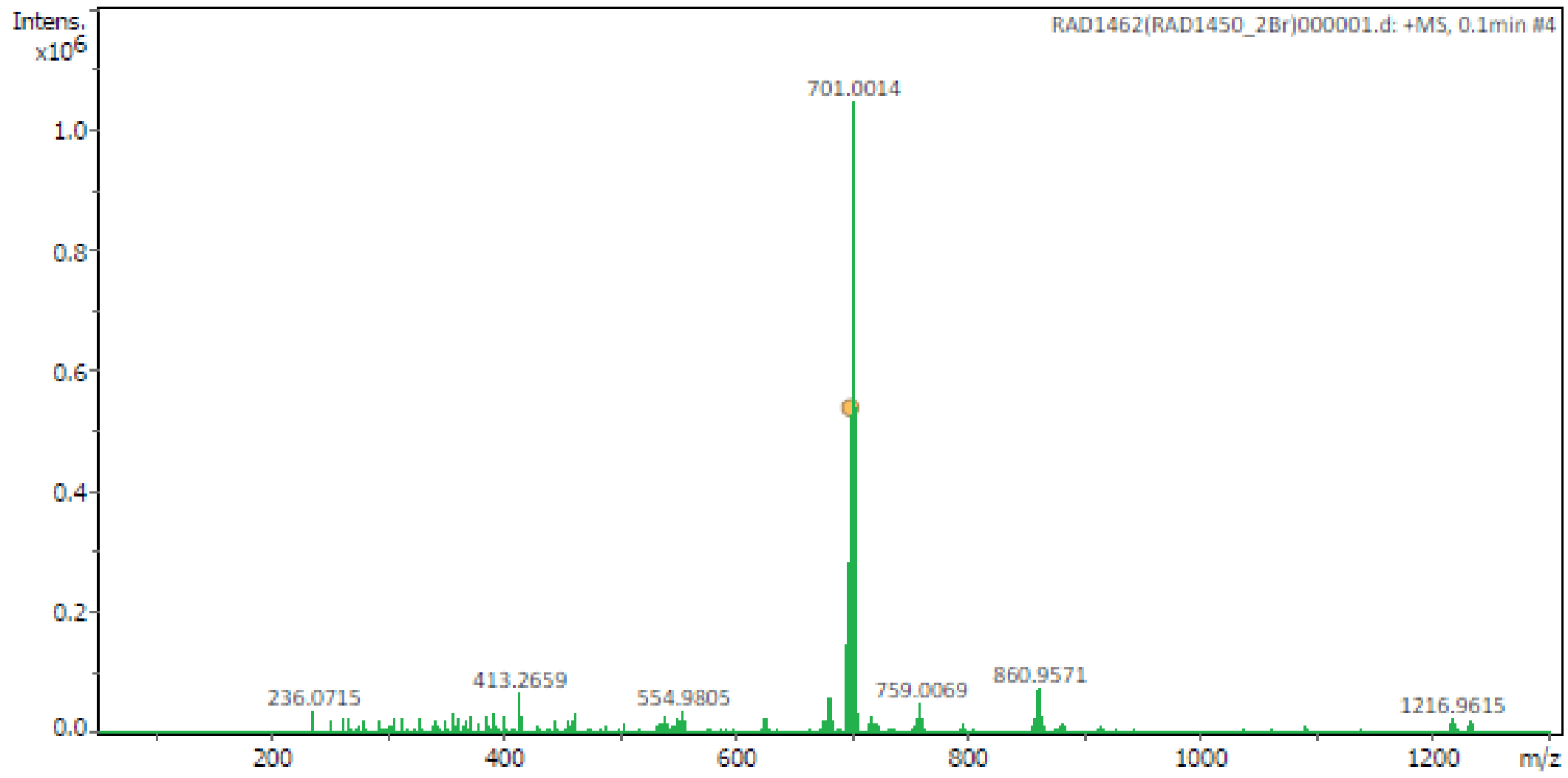

Meas. m/z	#	Ion Formula	m/z	err [ppm]	mSigma	# mSigma	Score	rdb	e⁻ Conf	N-Rule
699.0033	1	C23H22Br2N10NaO5	699.0034	0.2	4.9	1	100.00	21.0	even	ok
	2	C26H30Br2NaO11	699.0047	2.1	7.7	2	46.45	15.0	even	ok

Figure S110 LC-MS data of compound **13**

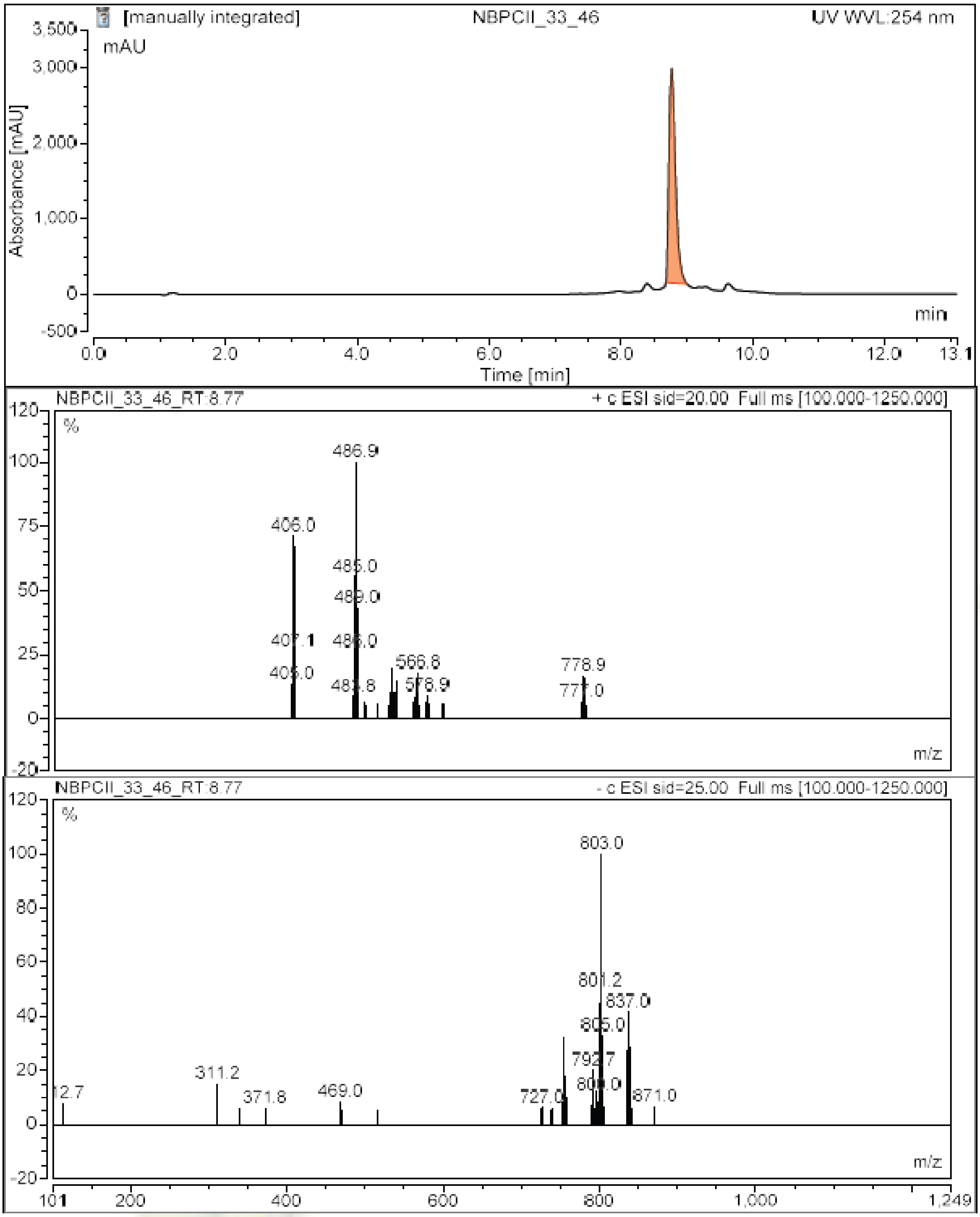

Figure S111 HRESIMS data of compound **13**

Mass Spectrum SmartFormula Report

Analysis Info

Analysis Name	D:\Data\Kanchana\20240108\NBC075-G_3Br000001.d	Acquisition Date	1/9/2024 12:40:20 PM
Method	DirectInfusion_2018_pos.m	Operator	Demo User
Sample Name	NBC075-G_3Br	Instrument	maXis II ETD 1823391.22321
Comment			

Acquisition Parameter

Source Type	ESI	Ion Polarity	Positive	Set Nebulizer	0.4 Bar
Focus	Not active	Set Capillary	4500 V	Set Dry Heater	180 °C
Scan Begin	50 m/z	Set End Plate Offset	-500 V	Set Dry Gas	4.0 l/min
Scan End	1300 m/z	Set Collision Cell RF	500.0 Vpp	Set Divert Valve	Waste

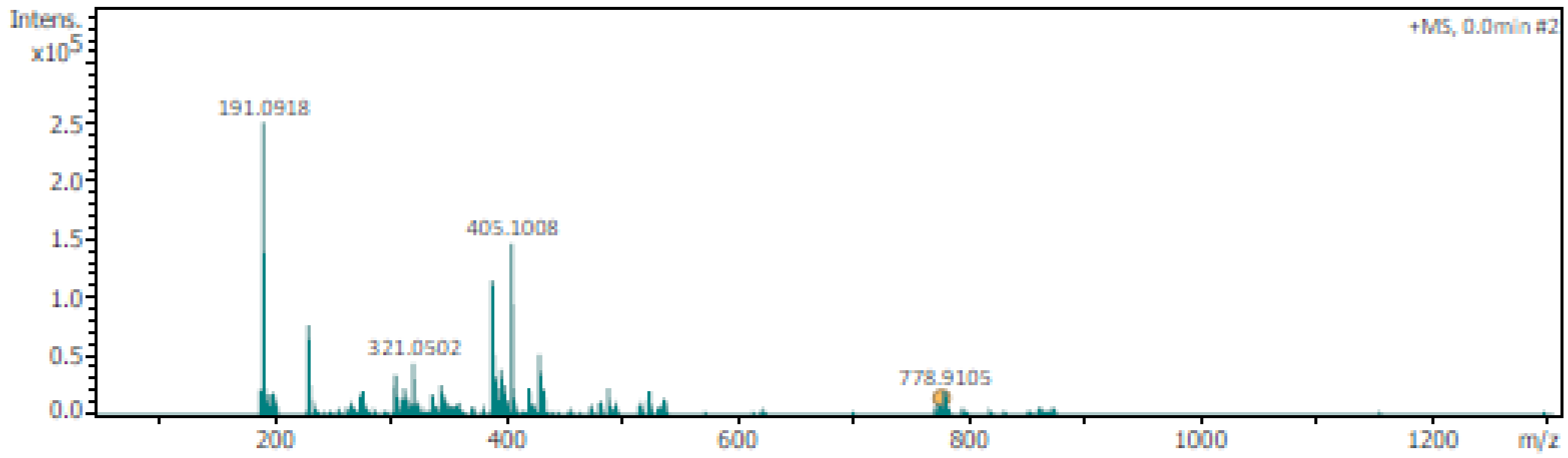

Meas. m/z	#	Ion Formula	m/z	err [ppm]	mSigma	# mSigma	Score	rdb	e⁻ Conf	N-Rule
776.9117	1	C26H29Br3NaO11	776.9152	4.5	15.9	1	100.00	17.0	even	ok

Figure S112 LC-MS data of compound **14**

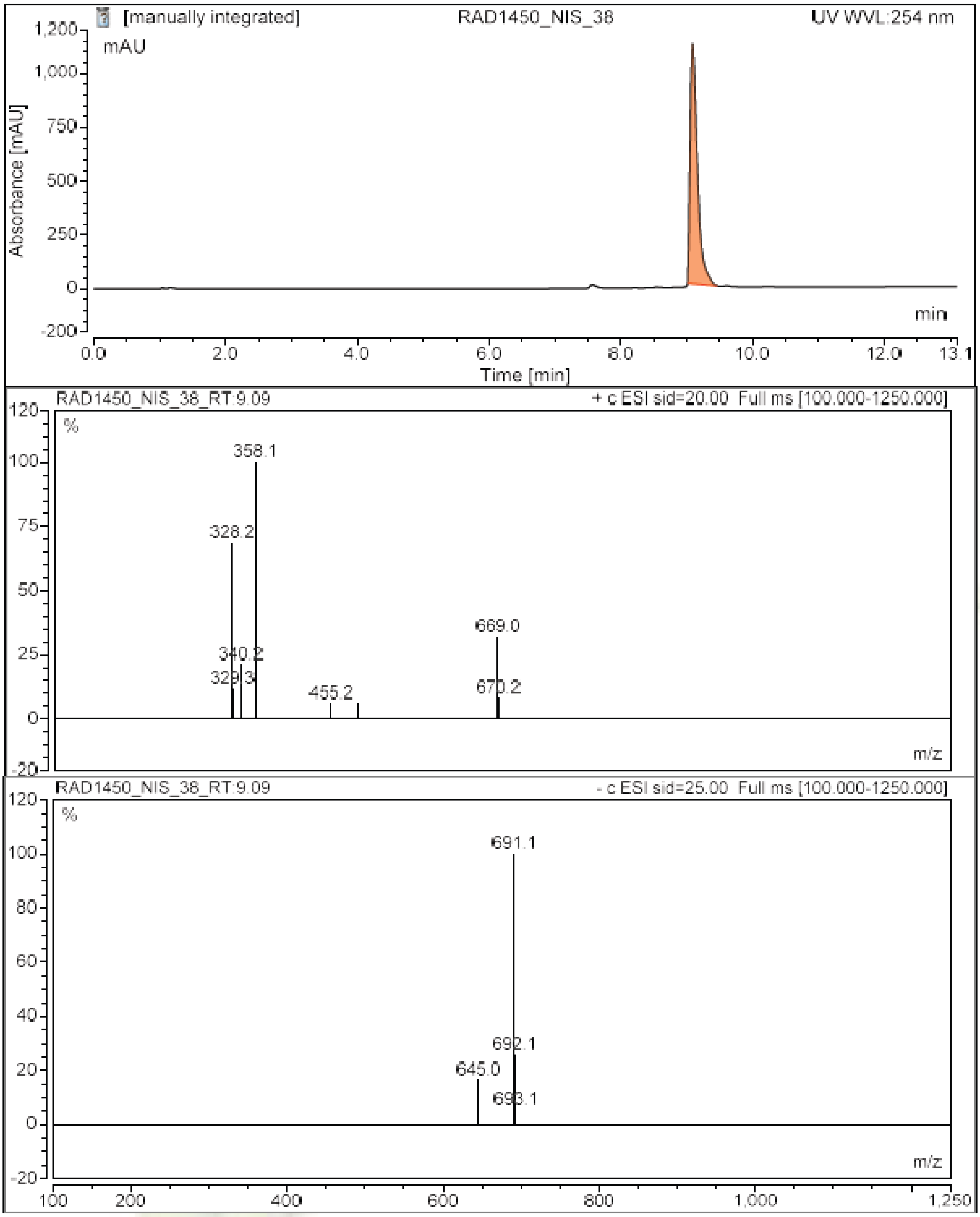

Figure S113 HRESIMS data of compound **14**

High Resolution Mass Spectrum

Analysis Info

Analysis Name	D:\Data\Kanchana\20240830\RAD1466000001.d		
Method	DirectInfusion_2018_pos.m	Acquisition Date	8/30/2024 11:06:50 AM
Sample Name	RAD1466	Instrument	maXis II ETD 1823391.22321
Comment			

+MS, 0.0min #1

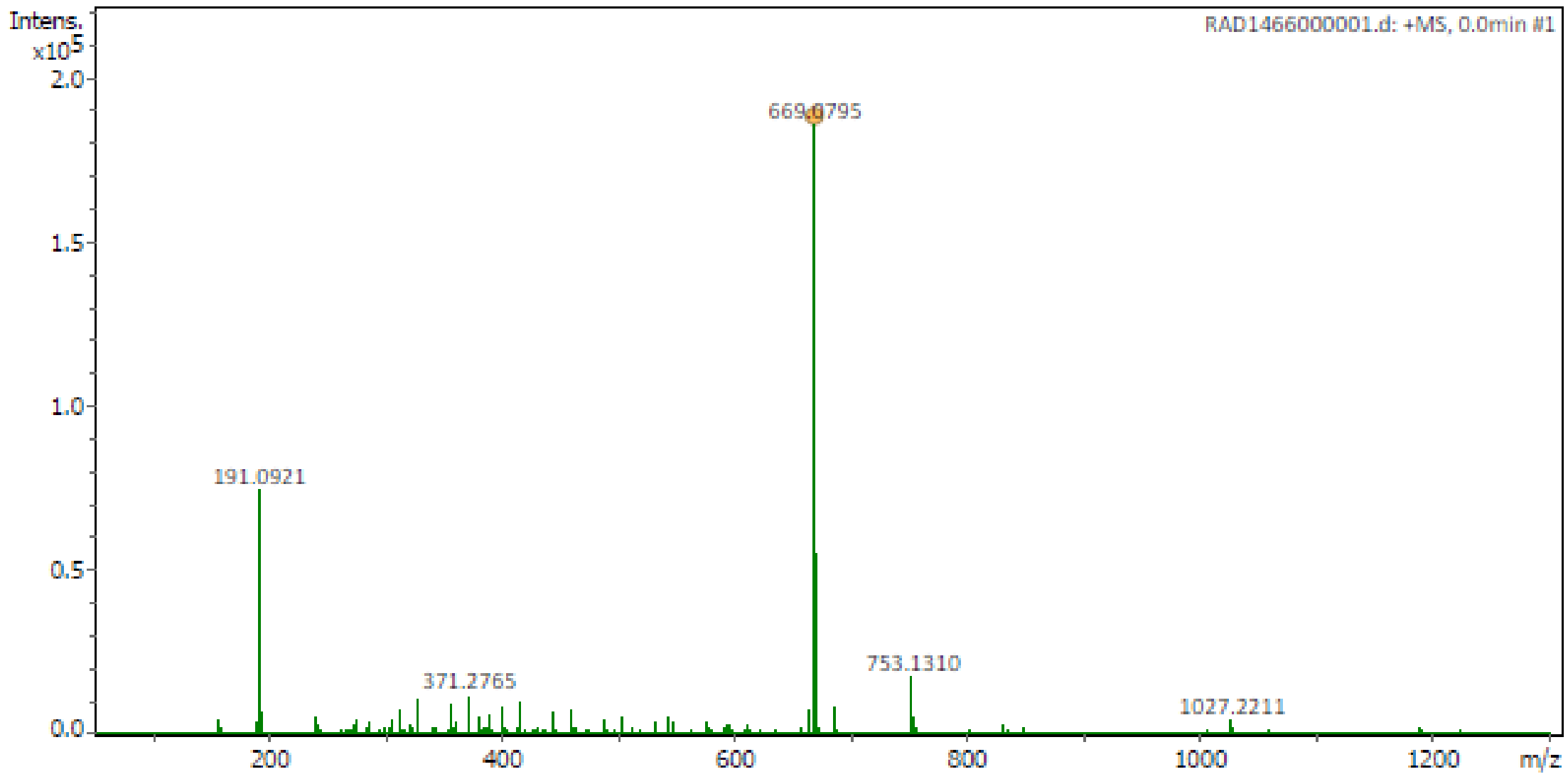

Meas. m/z	#	Ion Formula	m/z	err [ppm]	mSigma	# mSigma	Score	rdb	e⁻ Conf	N-Rule
669.0795	1	C26H31INaO11	669.0803	1.3	3.3	1	100.00	14.0	even	ok

Figure S114 LC-MS data of compound **15**

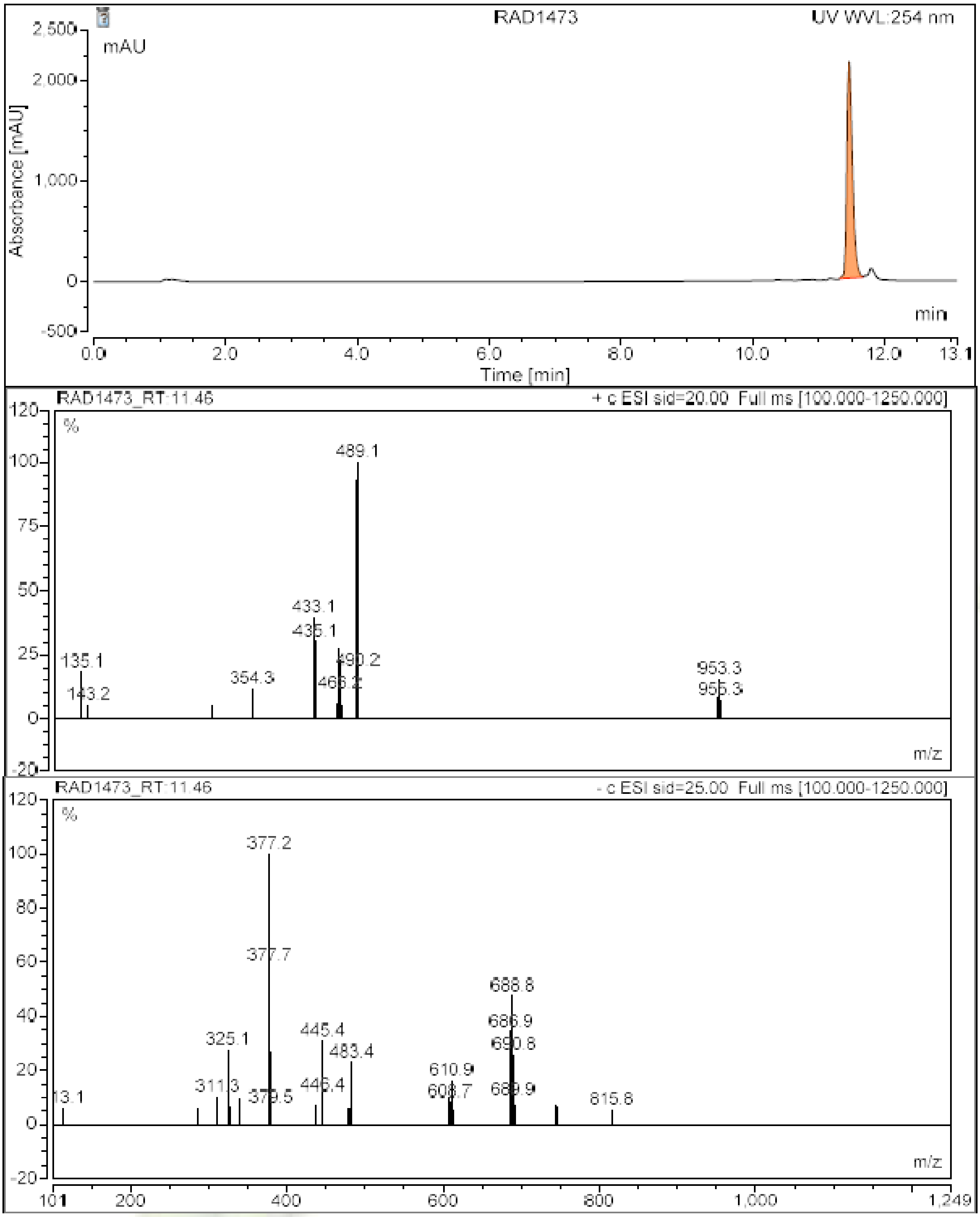

Figure S115 HRESIMS data of compound **15**

High Resolution Mass Spectrum

Analysis Info

Analysis Name	D:\Data\Kanchana\20240830\RAD1474000001.d		
Method	DirectInfusion_2018_pos.m	Acquisition Date	8/30/2024 11:50:35 AM
Sample Name	RAD1474	Instrument	maXis II ETD 1823391.22321
Comment			

+MS, 0.0min #1

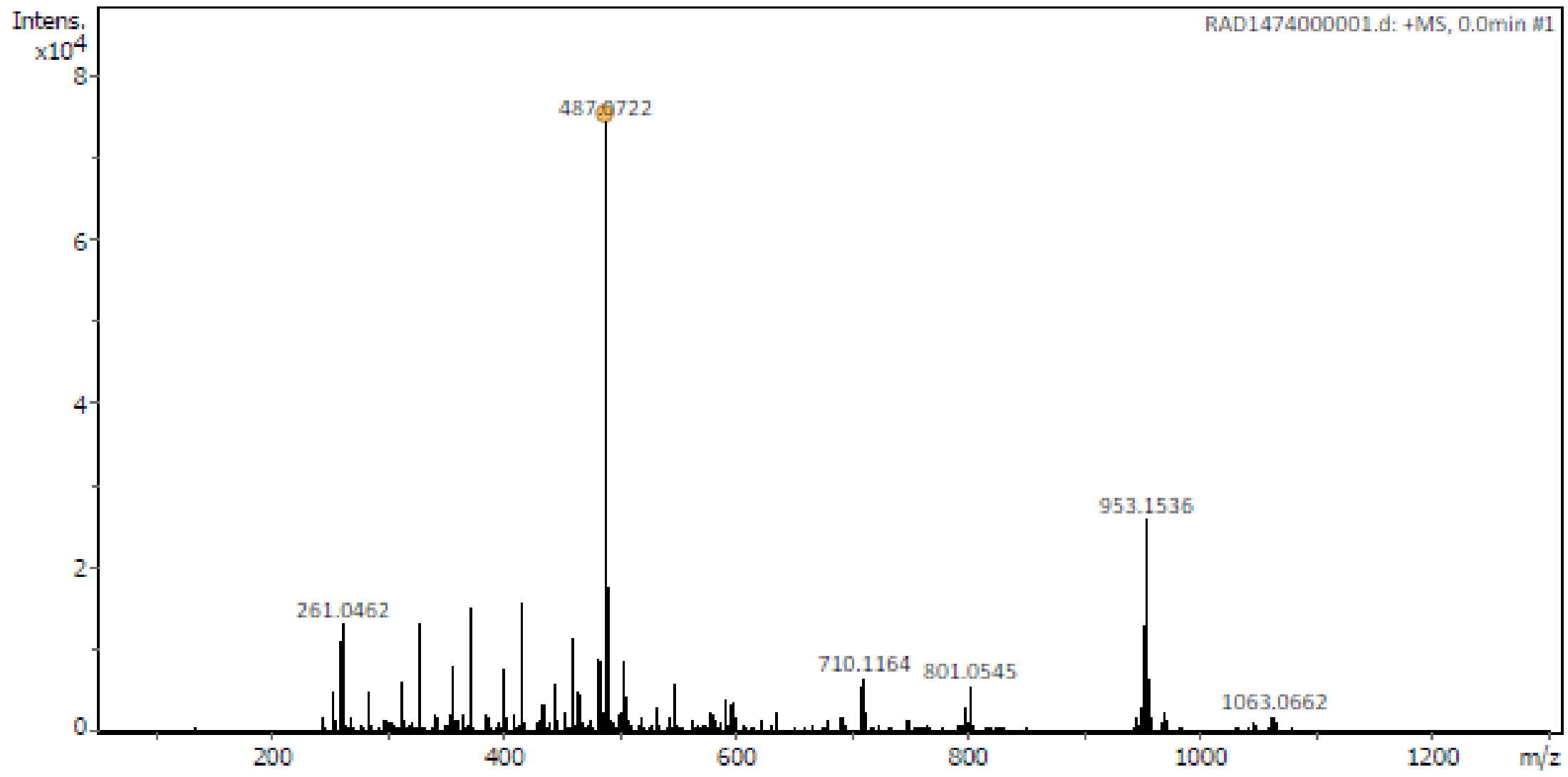

Meas. m/z	#	Ion Formula	m/z	err [ppm]	mSigma	# mSigma	Score	rdb	e⁻ Conf	N-Rule
487.0722	1	C22H25BrNaO6	487.0727	1.0	7.3	1	100.00	12.0	even	ok

Figure S116 LC-MS data of compound **16**

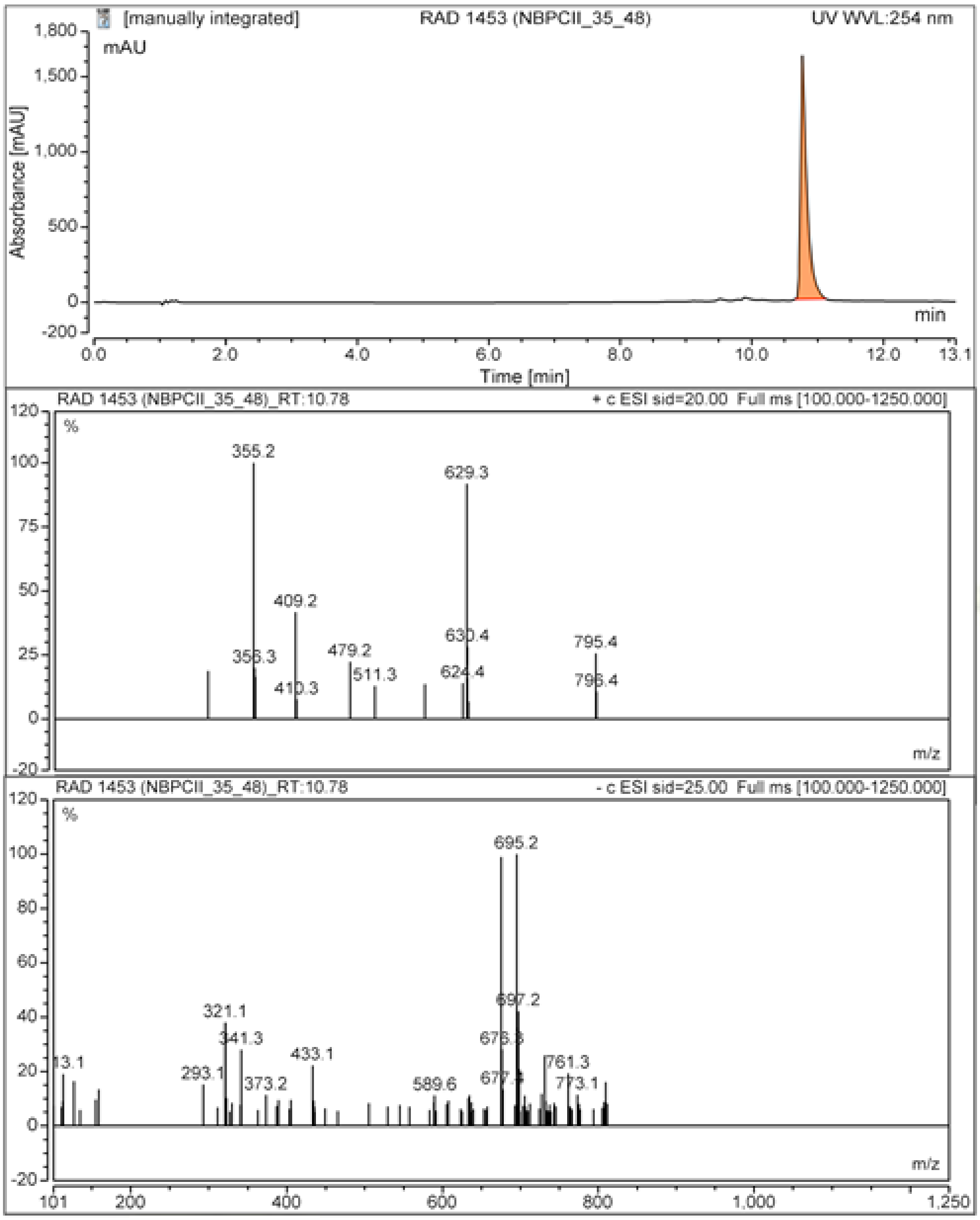

Figure S117 LC-MS data of compound **17**

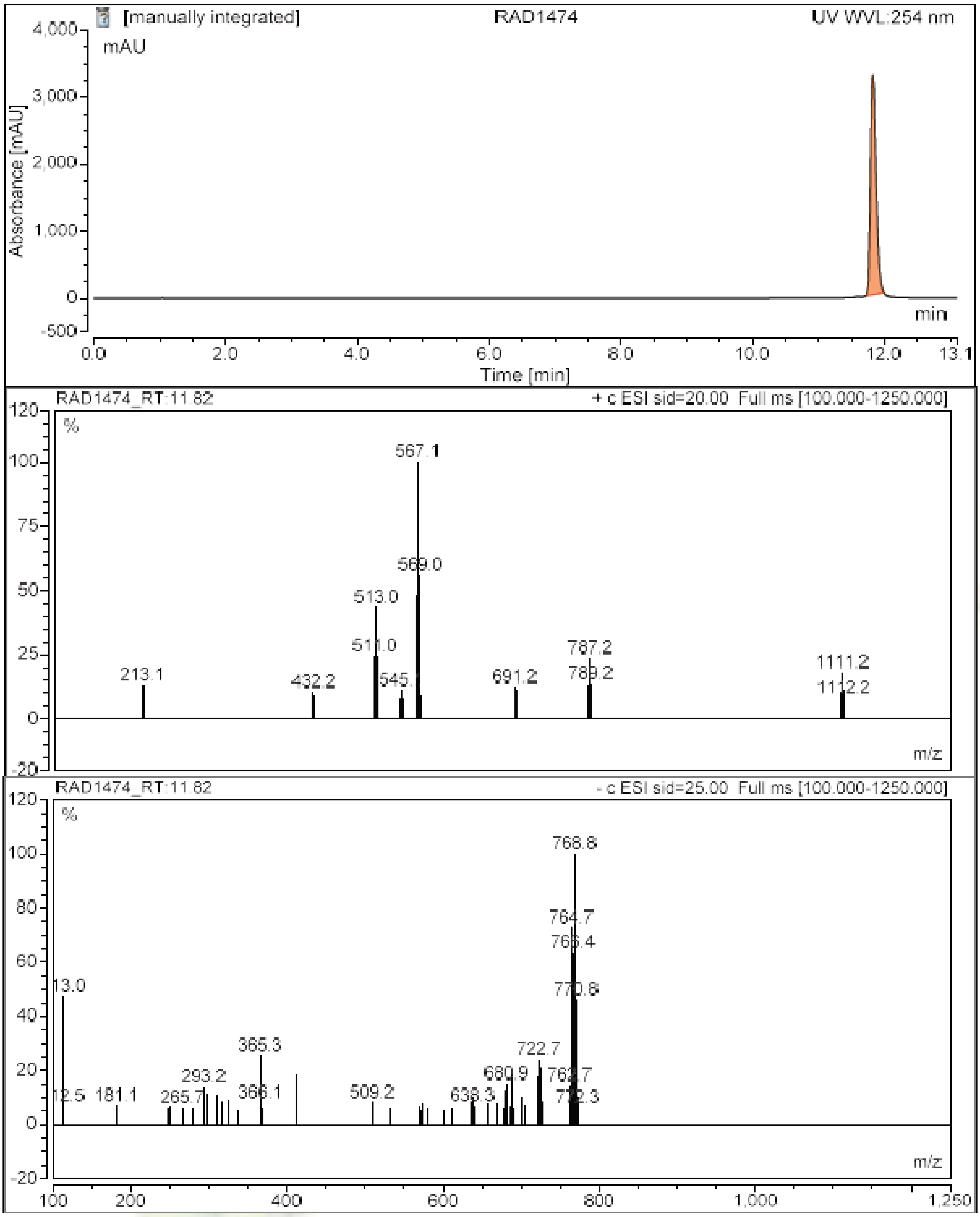

Figure S118 HRESIMS data of compound **17**

High Resolution Mass Spectrum

Analysis Info

Analysis Name	D:\Data\Kanchana\20240830\RAD1474_000001.d		
Method	DirectInfusion_2018_pos.m	Acquisition Date	8/30/2024 12:04:49 PM
Sample Name	RAD1474_	Instrument	maXis II ETD 1823391.22321
Comment			

+MS, 0.0min #2

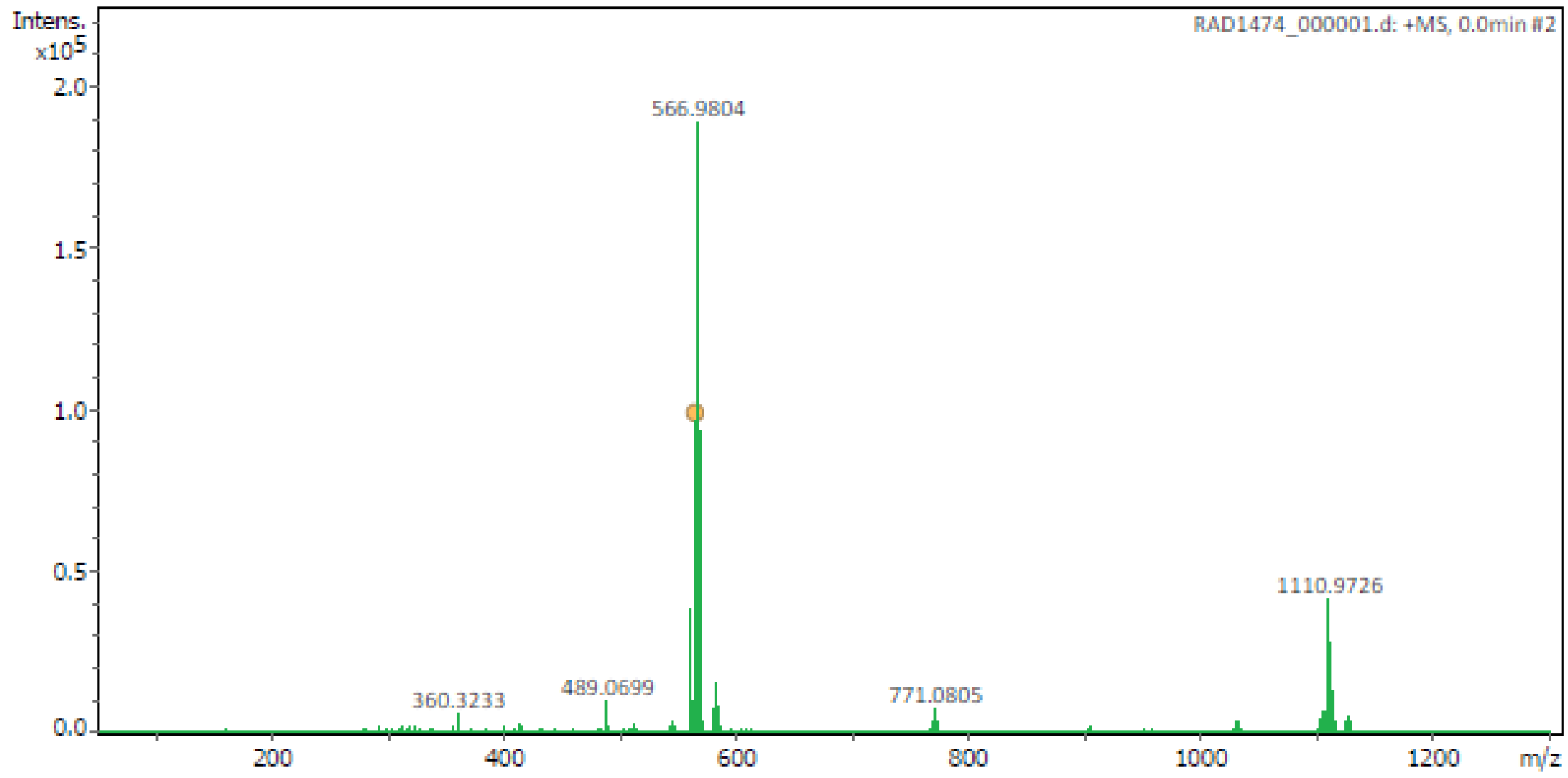

Meas. m/z	#	Ion Formula	m/z	err [ppm]	mSigma	# mSigma	Score	rdb	e⁻ Conf	N-Rule
564.9823	1	C22H24Br2NaO6	564.9832	1.6	8.7	1	100.00	14.0	even	ok

RAD1474_000001.d
Bruker Compass DataAnalysis 5.2 printed: 8/30/2024 12:09:45 PM by: demo Page 1 of 1

Figure S119 LC-MS data of compound **18**

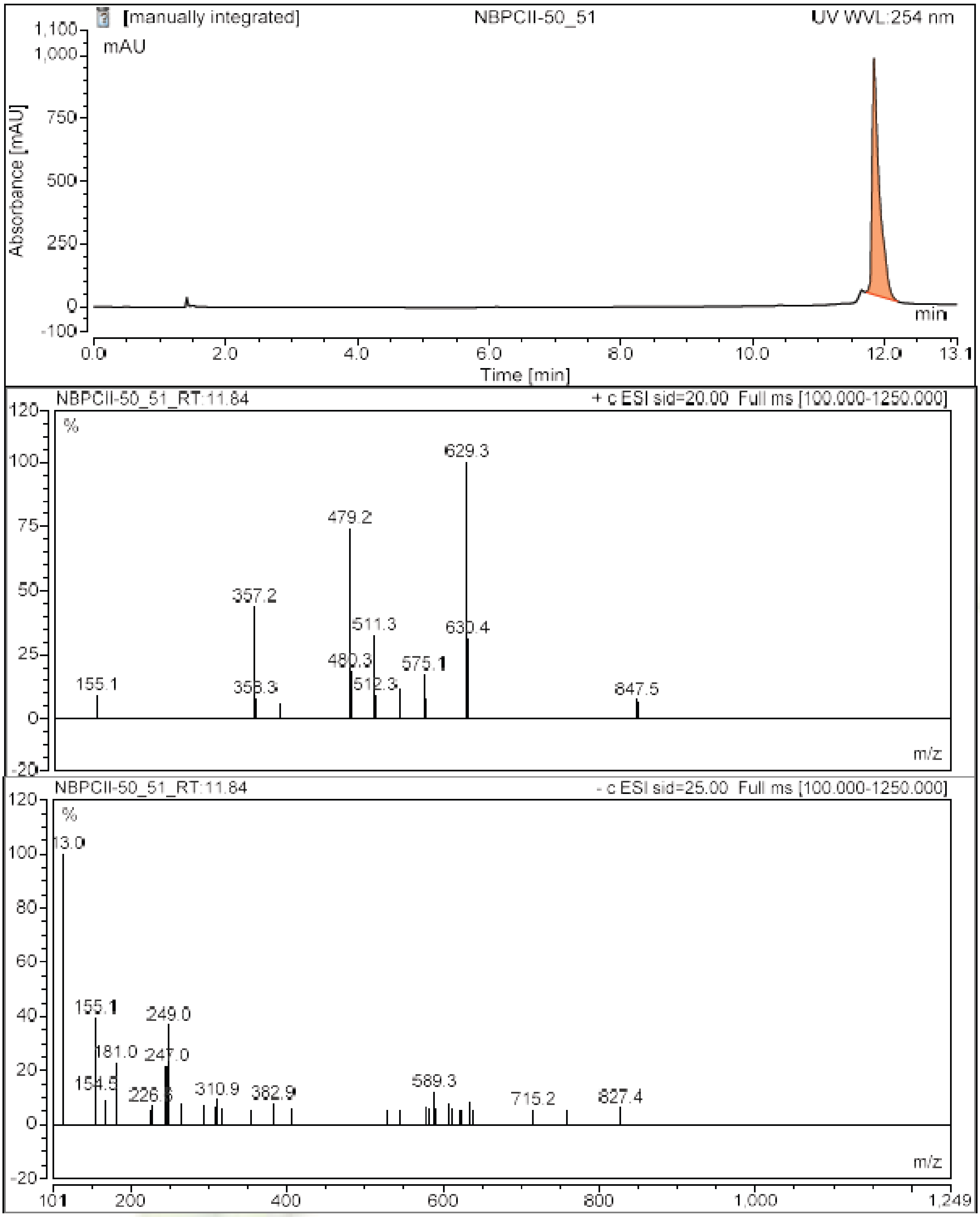

Figure S120 HRESIMS data of compound **18**

High Resolution Mass Spectrum

Analysis Info

Analysis Name D:\Data\Kanchana\20240830\RAD1470000001.d
Method DirectInfusion_2018_pos.m
Sample Name RAD1470
Comment

Acquisition Date 8/30/2024 11:38:57 AM
Instrument maXis II ETD 1823391.22321

+MS, 0.0min #1-2

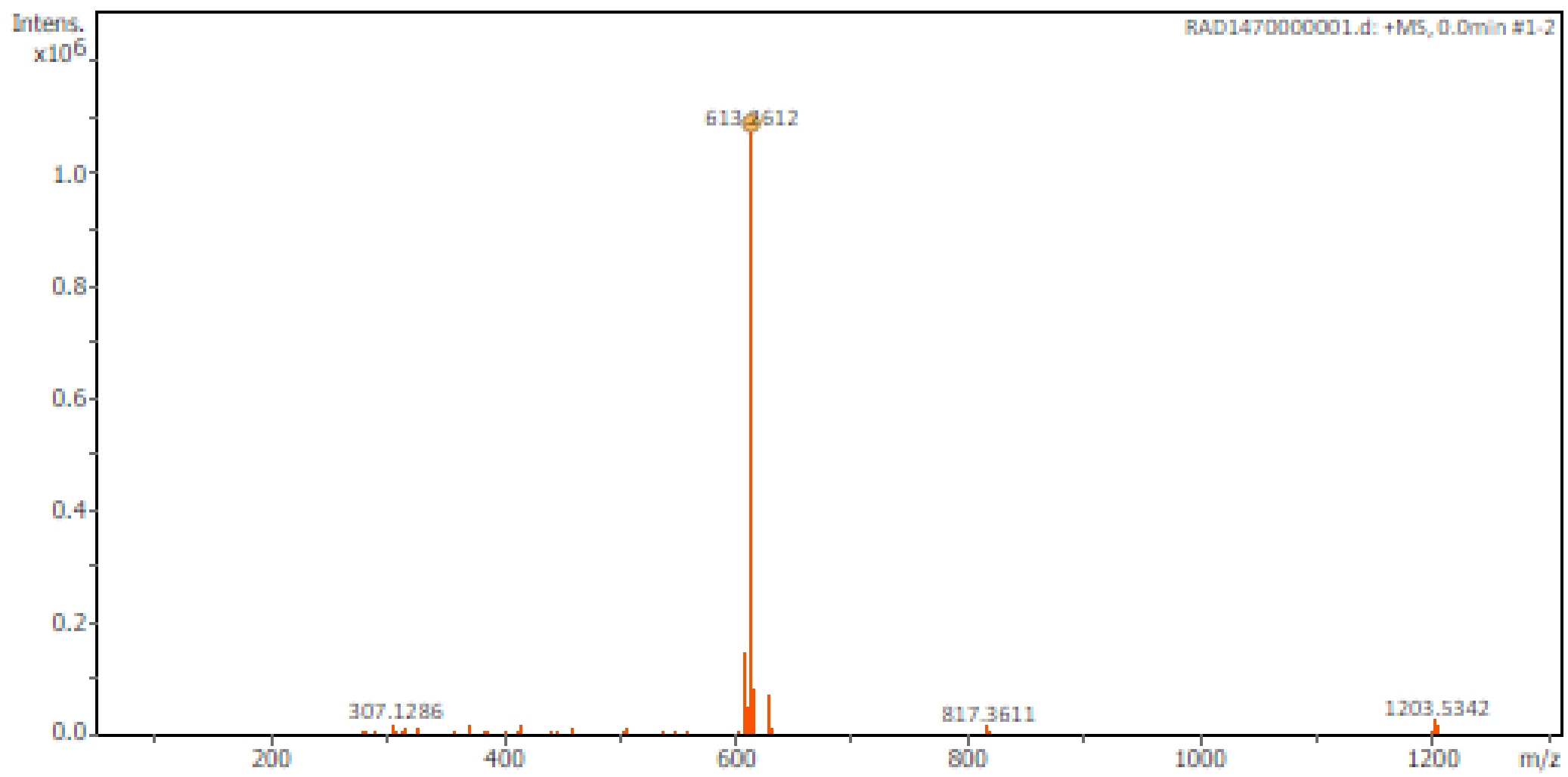

Meas. m/z	#	Ion Formula	m/z	err [ppm]	mSigma	# mSigma	Score	rdb	e¯ Conf	N-Rule
613.2612	1	C31H42NaO11	613.2619	1.1	4.0	1	100.00	11.0	even	ok

RAD1470000001.d
Bruker Compass DataAnalysis 5.2 printed: 8/30/2024 11:41:22 AM by: demo Page 1 of 1
